# Effects of a natural nutritional supplement on immune cell infiltration and immune gene expression in exercise-induced injury

**DOI:** 10.3389/fnut.2022.987545

**Published:** 2022-09-16

**Authors:** Feng Jiang, Rongfeng Yang, Diya Xue, Rong Li, Meiling Tan, Zhicong Zeng, Luhua Xu, Linling Liu, Yinzhi Song, Fengxia Lin

**Affiliations:** ^1^Department of Cardiology, Shenzhen Bao’an Chinese Medicine Hospital, Guangzhou University of Chinese Medicine, Shenzhen, China; ^2^Department of Cardiology, The Eighth Affiliated Hospital, Sun Yat-sen University, Shenzhen, China; ^3^Department of Obstetrics, Shenzhen Bao’an Chinese Medicine Hospital, Guangzhou University of Chinese Medicine, Shenzhen, China; ^4^Wenhua Community Health Service Center, Shenzhen Luohu Hospital Group, Shenzhen, China

**Keywords:** sport injury, nutritional supplement, immune infiltration, inflammation, echinacoside

## Abstract

Inflammatory immune response plays a key role in exercise-induced injury and healing; however, the relevant regulatory mechanisms of immune infiltration in exercise-induced injuries remain less studied. In the present study, a highly efficient system for screening immunity-related biomarkers and immunomodulatory ability of natural nutritional supplements was developed by integrating intelligent data acquisition, data mining, network pharmacology, and computer-assisted target fishing. The findings demonstrated that resting natural killer cells showed a higher rate of infiltration after exercise, whereas naive B cells and activated dendritic cells showed higher rate of infiltration before exercise. Four key genes, namely *PRF1*, *GZMB*, *CCL4*, and *FASLG*, were associated with exercise-induced injuries and inflammatory immune response. In total, 26 natural compounds including echinacoside, eugenol, tocopherol, and casuariin were predicted by using the HERB databases. Molecular docking analysis showed that GZMB, FASLG, and CCL4 bound to echinacoside. *In vivo* experiments in mice showed that after 30 min swimming, natural killer (NK) cells showed high infiltration rates, and the key genes (*GZMB*, *PRF1*, *FASLG*, and *CCL4*) were highly expressed; however, echinocandin significantly reduced the level of NK cells and decreased the expression of the four key genes post exercise. This natural nutritional supplement may act to protect against inflammatory injury after exercise by suppressing specific immune infiltration.

## Introduction

Exercise and physical activity are effective in the prevention and treatment of a wide range of chronic diseases, and there is considerable evidence for a positive association between long-term exercise and health benefits (1). Physical activity lowers diabetic blood sugar levels (2) and blood pressure (3), reduces the risk of acute cardiovascular disease (4, 5) malignancies such as breast and colon cancer (6), prevents memory and cognitive impairment (7), and enhances immunity to pathogens (8). The Physical Activity Guidelines for Americans (9) recommend that children and adolescents should engage in moderate-to-vigorous physical activity for 60 min or more per day, and adults should engage in at least 150 min of moderate-intensity aerobic activity or at least 75 min of vigorous aerobic activity per week. However, injuries are an inevitable part of exercise and participation in sport; they affect continuation of the sport activity, and the inflammatory response caused by the injury is detrimental to health and may affect athletes’ ability and performance. Thus, the prevention and treatment of sports-related injuries is particularly important. Inflammation is prevalent in the body after exercise; under normal circumstances, this short-term immune response facilitates the removal of necrotic damaged tissue to achieve renewal and repair (10, 11). Inflammation is also one of the main causes of pain and discomfort from post-exercise injuries, and non-steroidal anti-inflammatory drugs (NSAIDs) are used in certain high-intensity competitive sports (12). Delayed-onset muscle soreness is a common negative manifestation of post-exercise discomfort in the body, which was thought to be the result of a combination of factors such as connective tissue damage, muscle damage, lactic acid accumulation, and inflammatory response (13). Chronic inflammation caused by frequent sports-related injuries may place a greater burden on the body than the benefits of exercise. A survey of college athletes showed that 94% had used NSAIDs and 13.9% had overdosed on NSAIDs (14). However, NSAIDs have side effects such as gastrointestinal bleeding, kidney damage, and even inhibition of cartilage proliferation (15). Nutritional therapy is an important strategy to repair sports injuries; it is more widely available than conventional medical care and results in significant cost savings (16). In addition, dietary supplements that relieve pain and discomfort after sports injuries and promote injury repair have fewer side effects than, for example, NSAIDs, and are more effective in improving physical fitness (17). Natural ingredients, such as curcumin, quercetin, and resveratrol, are a great treasure trove and have been found to be effective for sports injuries (18–20). Our previous studies have found that *Rhodiola rosea* as a natural supplement has a positive effect on exercise capacity and performance, reduces post-exercise pain and skeletal muscle injury, and enhances antioxidant capacity (21). Therefore, we aimed to identify more natural nutritional supplements that are effective in the prevention of and recovery from sports-related injuries. We hope to find some key genes from which to predict some potential natural ingredients that exert anti-inflammatory effects in sports injuries, and use experiments to validate the anti-inflammatory effects of these candidate ingredients, and eventually convert these candidates into natural dietary supplements for use in the anti-inflammatory response to sports injuries.

## Materials and methods

### Gene expression profiles before and after exercise

Gene expression profiles of individuals before and after exercise was obtained by searching the GEO database; gene IDs were collected and then converted into gene symbols.

### Analysis of immune cell infiltration and differentially expressed genes

The CIBERSORT deconvolution method (perm = 1000) was used to analyze immune cell infiltration. The gene expression profiles were screened for differentially expressed genes (DEGs) after normalization using the R limma package based on the cut-off criteria | logFC| ≥ 1 and adjP ≤ 0.05 (22).

### Immune-related differentially expressed genes

In addition to immune cell infiltration analysis, we also studied the differential expression of immune-related genes before and after exercise. Immune-related genes were downloaded from the ImmPort database; subsequently, they were compared with DEGs to obtain a list of immune-related differentially expressed genes (ImmDEGs) (23).

### Protein–protein interaction, hub genes, and enrichment analyses

We used the STRING database for protein–protein interaction (PPI) analysis of DEGs with confidence level ≥ 0.04 and filtered the top five genes, which were considered to be the hub genes according to Cytoscape’s Cytohubba plugin (Degree algorithm). DEGs were also subjected to gene ontology (GO) enrichment analyses using the R package clusterProfiler (cutoff: *P* ≤ 0.05 and *q* ≤ 0.05) (24). Furthermore, to examine the overall gene enrichment more comprehensively, we performed a gene set enrichment analysis (GSEA) of all genes after sorting by logFC descending order using R soft (pvalueCutoff = 0.05, pAdjustMethod = “BH”) (25).

### Prediction of key genes and nutrients and molecular docking for validation

Key genes were defined as intersecting genes between ImmDEGs and hub genes. On the basis of the identified key genes, we used the HERB database to back-predict the target nutrients (26). Proteins encoded by key genes were downloaded from the PDB database and subjected to molecular docking analysis using AutoDock software to validate nutrients (Table 1).

**TABLE 1:** All relevant software and websites used in this study.

Name	Entrance
GEO database	https://www.ncbi.nlm.nih.gov/geo/
R soft and main plug-in package	Version: R 4.1.1; Package: limma, clusterprofiler
ImmPort database	https://www.immport.org/home
String database	https://cn.string-db.org/
Cytoscape	Version: Cytoscape_v3.9.0; Plug-in: Degree
HERB database	http://herb.ac.cn/
PubChem database	https://pubchem.ncbi.nlm.nih.gov/
ChemOffice	Chem3D 19.0
Uniprot database	https://www.uniprot.org/
PDB database	https://www.rcsb.org/
Autodock vina	Autodock vina 1.1.2

### Materials and experimental animals and treatment

According to the predicted results, we selected echinacoside for *in vitro* validation. Echinacoside was purchased from Chengdu HerbSubstance Co., Ltd. (Chengdu, China) and the percentage purity of echinacoside was 99.85%. Echinacoside was dissolved in 25 mg/mL dimethyl sulfoxide (DMSO) and diluted to 10mg/mL with phosphate-buffered saline. The final concentration of DMSO was less than 10%; the solution was then divided into 1.5 mL aliquots and stored at −80 °C until further use. Specific pathogen-free male C57BL/6 mice (body weight: 20 ± 2 g, age: 56–62 days, *n* = 12) were purchased from the Guangdong Medical Laboratory Animal Center (Guangdong, China). The mice were maintained under controlled temperature (22 ± 1 °C), humidity (50%), and lighting (12:12 h light/dark) conditions. After 7 days of habituation, the mice were randomly divided into four groups (*n* = 3 per group; Figure 3D). In the normal control group (NC group), mice were gavaged with distilled water (10 mL/kg) once a day for 1 week. In the model group, mice were gavaged with distilled water (10 mL/kg) once a day for 1 week and subjected to passive swimming activity for 30 min on the last day. In the long-term echinacoside group (LE group), mice were gavaged with echinacoside (100 mg/kg) (27) once a day for 1 week and subjected to passive swimming for 30 min on the last day. In the short-term echinacoside group (SE group), mice were gavaged with distilled water (10 mL/kg) once a day for 6 days; on the seventh day, the mice were gavaged with echinacoside (100mg/kg) (27) and, after 30 min, subjected to passive swimming for 30 min. Peripheral blood and muscle tissues were harvested immediately after the swimming session. All animal experiments were approved by the Animal Care and Use Committee of Affiliated Hospital of Guangzhou University of Chinese Medicine (approval number:20200331016), and experiments were performed in accordance with the Guide for the Care and Use of Laboratory Animals published by the US National Institutes of Health.

### Flow cytometry analysis

Flow cytometry was used to detect the percentage of natural killer (NK) cells and dendritic cell (DC) subsets in the peripheral blood of mice. Peripheral blood was collected into tubes containing ethylenediaminetetraacetic acid. Peripheral blood mononuclear cells (PBMCs) were obtained from whole blood by density gradient centrifugation. PBMCs (1 × 10^6^) were incubated with fluorescein isothiocyanate-conjugated rat anti-mouse antibodies against CD45/PE, NK1.1/APC, CD11C/PE-CY5, and CD3 for 60 min in the dark. The antibodies were purchased from Tonbo (Beijing, China). The cell samples were detected on Novocyte D2060R (Agilent, CA, USA), and the percentage of CD3^–^NK1.1^+^ NK cell subset and CD11C^+^NK1.1^–^ DC subset was analyzed using NovoExpress 1.4.1.

### Quantitative reverse transcription PCR

Total RNA was extracted from muscle tissues using TRIzol reagent. cDNA was synthesized using the EvoM-MLV kits. Quantitative reverse transcription-PCR (RT-qPCR) was performed using 2X SYBR Green qPCR Master Mix (K1070-500, APExBIO, US) on a CFX96 Real-Time PCR Detection System (Bio-Rad Laboratories) following the manufacturer’s protocol and analyzed using the 2^–ΔΔCt^ method. The following optimized thermal conditions were used: 95°C for 30 s, 95°C for 5 s, and 40 cycles at 60°C for 5 s. The levels of mRNA were normalized to endogenous GAPDH, and the expression of target genes was analyzed using the 2^–ΔΔCt^ method. The experiments were repeated three times independently. The primer sequences used in this study are listed in Table 2.

**TABLE 2:** Primers used for quantitative reverse transcription-PCR.

Target	Primer	Sequence (5′-3′)
GZMB	FP	CCTGCTACTGCTGACCTTGT
	RP	GGGATGACTTGCTGGGTCTT
FASLG	FP	CAGCCCATGAATTACCCATGT
	RP	ATTTGTGTTGTGGTCCTTCTTCT
CCL4	FP	AAGCCAGCTGTGGTATTCCTGA
	RP	ATCTGAACGTGAGGAGCAAGG
PRF1	FP	CTGCCACTCGGTCAGAATG
	RP	CGGAGGGTAGTCACATCCAT

### Statistical analysis

Data were expressed as mean ± standard error of the mean (SEM). GraphPad Prism 8.0 software was used to perform statistical analysis and construct histograms. The differences between groups were evaluated by *t*-tests or one-way analysis of variance (ANOVA) with appropriate *post hoc* tests; and *P* < 0.05 was considered to indicate statistically significant differences.

## Results

### Gene expression profiles

We downloaded a gene expression matrix for GSE14642 from the GEO database. This data matrix contains data on gene expression levels in peripheral blood of 20 young women before and after 30 min cycle ergometry exercise (28). The work rate was calculated to 50% of the work rate between the anaerobic threshold and the peak oxygen uptake Individually.

### Immune cell infiltration and differentially expressed genes

**FIGURE 1:**
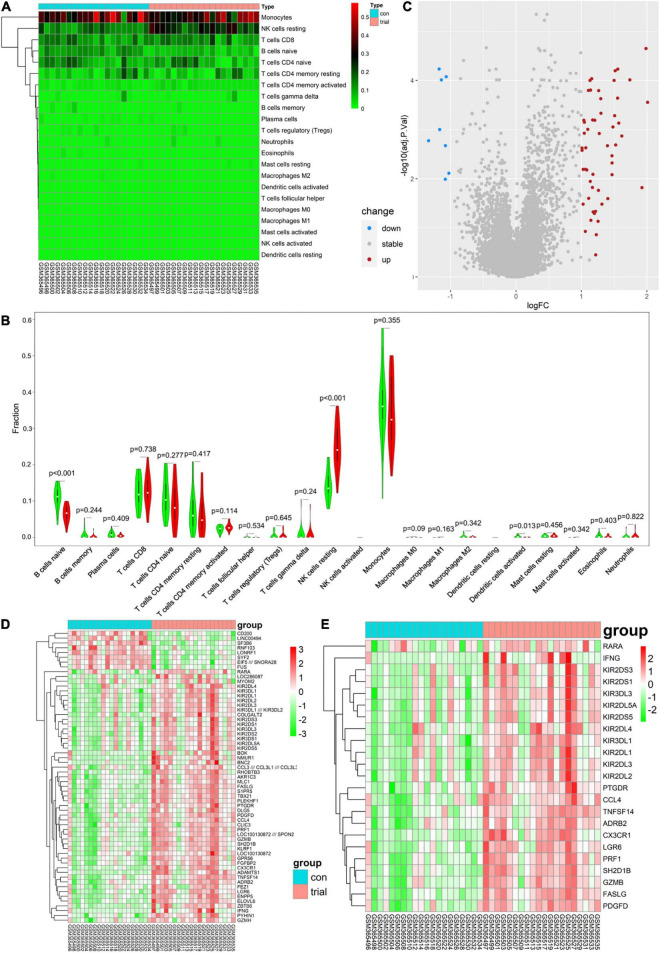
**(A)** Each column represents a sample, and each row represents a type of immune cells. Color transition from green to red represents an increase in immune cell infiltration level. Con group = before exercise; trial group = after exercise. **(B)** Green and red violin columns represent immune cell infiltration levels before and after exercise, respectively. The vertical axis represents the ratio of immune cell infiltration responsible for total immune cell infiltration. *P* value, obtained using the Wilcoxon test, represents the statistical significance of the difference in the immune cell infiltration levels before and after exercise. **(C)** Upregulated and downregulated differentially expressed genes (DEGs) are highlighted in red and blue, respectively. Criteria: |logFC| ≥ 1 and adjP ≤ 0.05. **(D)** Expression levels of 61 DEGs are shown; the darker the red color, the higher the expression level, and the darker the green color, the lower the expression level. Con group = before exercise; trial group = after exercise. **(E)** Expression levels of 23 immune-related differentially expressed genes are shown; the darker the red color, the higher the expression level, and the darker the green color, the lower the expression level. Con group = pre-exercise; trial group = post-exercise.

There were marked differences in the immune cell infiltration in these women before and after exercise (Figure 1A). Subsequently, Wilcoxon tests showed that resting NK cells were more highly infiltrated after exercise (*P* = 0.001), whereas naive B cells and activated DCs were more highly infiltrated before exercise (*P* = 0.001 and *P* = 0.013, respectively; Figure 1B). Moreover, 61 DEGs were identified: 8 genes were downregulated, and 53 were upregulated (Figures 1C,D).

### Immune-related differentially expressed genes

Analysis of immune-related gene expression levels identified 23 ImmDEGs: all of them (e.g., *PRF1*) were highly expressed after exercise (Figure 1E).

### Protein–protein interaction network construction, hub gene selection, and enrichment analysis

**FIGURE 2:**
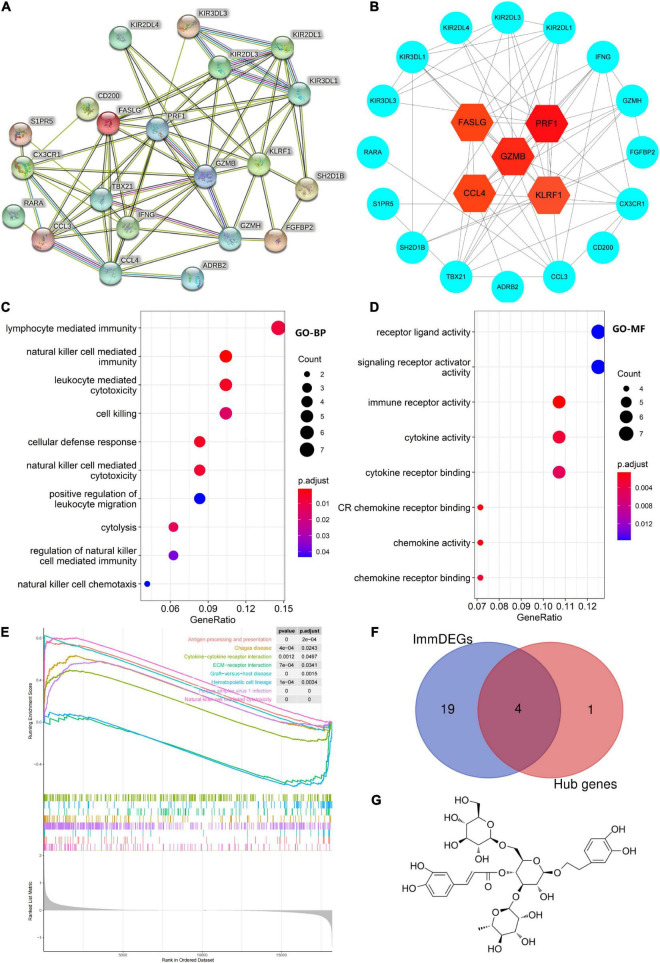
**(A)** Protein–protein interaction network of differentially expressed genes (DEGs). **(B)** Hub gene map, the darker the red color of the node, the stronger the protein interaction. **(C,D)** Top 10 biological processes and all molecular functions observed to be enriched in gene ontology enrichment analysis. The horizontal axis represents the gene ratio, that is, the ratio of the number of DEGs to number of total genes. Dot size is proportional to the gene ratio, and dot color transition from blue to red indicates that the adjusted P value is getting smaller. **(E)** Gene set enrichment analysis results, with each colored line representing an enriched pathway. **(F)** Intersection of immune-related differentially expressed genes (ImmDEGs) and hub genes; the genes common between the two groups were *GZMB*, *FASLG*, *CCL4*, and *PRF1*. **(G)** The two-dimensional structure of echinacoside.

Five hub genes (*PRF1*, *GZMB*, *KLRF1*, *CCL4*, and *FASLG*) were identified on the basis of PPI analysis (Figures 2A,B). GO enrichment analysis revealed that DEGs were enriched in 52 biological processes (GO-BP) mainly associated with, for example, NK cell-mediated immunity and cellular defense response. Similarly, DEGs were enriched in eight molecular functions (GO-MF), with the major categories being immune receptor activity and chemokine activity (Figures 2C,D). However, cellular components were not significantly enriched. GSEA enrichment analysis showed that eight pathways were enriched; six pathways including NK cell-mediated cytotoxicity were upregulated after exercise, and two pathways including ECM-receptor interaction were downregulated after exercise (Figure 2E and Table 3).

**FIGURE 3:**
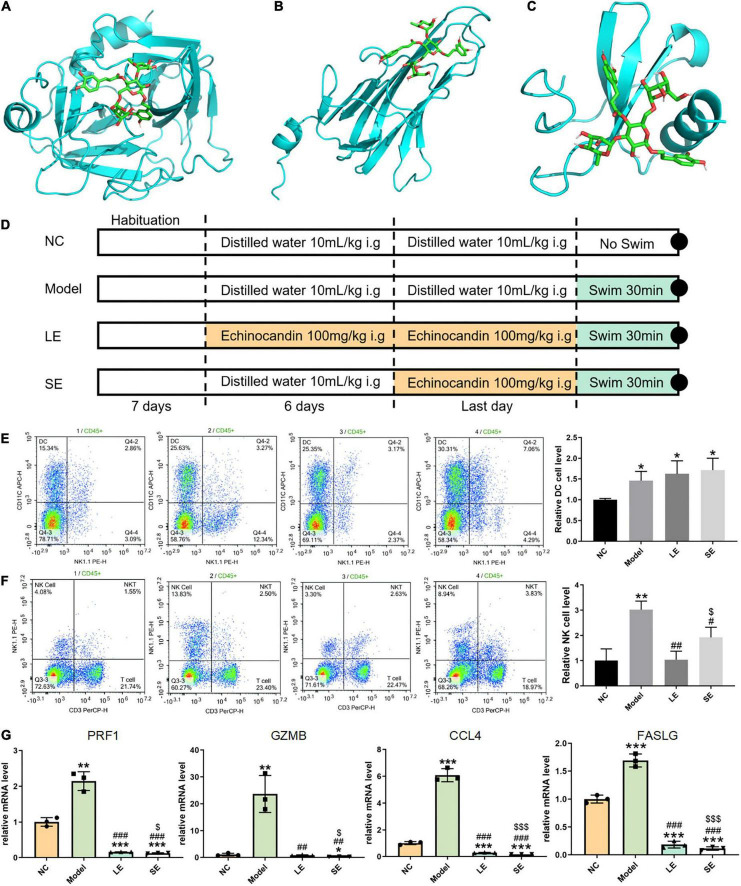
**(A–C)** Lowest-free-energy docking models of echinacoside with GZMB, FASLG, and CCL4, respectively. **(D)** Depiction of the control and experimental groups. **(E,F)** Results of flow cytometry analysis of dendritic cells (DCs) and natural killer (NK) cells, respectively. **(G)** Quantitative reverse transcription-PCR results for key gene expression levels averaged over three independent experiments. Data are shown as mean ± standard error of the mean. *p < 0.05, **p < 0.01, ***p < 0.001 vs. NC; ^#^*p* < 0.05, ^##^*p* < 0.01, ^###^*p* < 0.001 vs. Model; ^$^*p* < 0.05, ^$$^*p* < 0.01, ^$$$^*p* < 0.001 vs. LE.

**TABLE 3:** Gene set enrichment analysis results.

Pathway	Enrichment score	NES	*P*-value	Adjusted *P*	q-value
Natural killer cell mediated cytotoxicity	0.8020	2.1665	1.00E-10	3.33E-08	3.09E-08
Antigen processing and presentation	0.7687	1.9303	1.94E-06	0.0002	0.0002
Graft-versus-host disease	0.8231	1.868	1.79E-05	0.0015	0.0014
Herpes simplex virus 1 infection	0.5788	1.7565	5.57E-10	9.27E-08	8.61E-08
Chagas disease	0.6336	1.6734	0.0004	0.0242	0.0226
Cytokine-cytokine receptor interaction	0.4958	1.4536	0.0012	0.0497	0.0462
ECM-receptor interaction	–0.5803	–1.6812	0.0007	0.0341	0.0317
Hematopoietic cell lineage	–0.6125	–1.7857	5.09E-05	0.0034	0.0031

### Prediction of key genes and nutrients and molecular docking

Four common genes (*PRF1*, *GZMB*, *CCL4*, and *FASLG*) were obtained by intersecting the sets of ImmDEGs and hub genes, which were considered as key genes (Figure 2F). The top five natural Chinese herbs with the lowest P value predicted on the basis of key genes in the HERB database were selected (Table 4). The 26 candidate (Table 5) ingredients were identified by the intersection of the related target ingredients directly predicted based on key genes by the HERB database and the active ingredients of above-mentioned Chinese herbs. However, *PRF1* failed to predict natural Chinese medicine by the database. Of these 26 candidates, echinacoside (Figure 2G) was the most interesting to us, as this compound is the active ingredient in our predicted herb, Herba Cistanches, which have been used in China for thousands of years and are still used to this day to enhance physical functions. Based on this fact, we consider echinacoside to be the highest priority research candidate. Molecular docking of selected compound to key genes showed that GZMB, FASLG, and CCL4 bind tightly to echinacoside with their lowest binding free energies of −8.0kcal/mol, −7.2kcal/mol, −6.9kcal/mol, respectively (Figures 3A–C). However, we were unable to analyze the molecular docking of PRF1 with echinacoside because its 3D structure was not accessible.

**TABLE 4:** Top five herbs with the lowest *P* value predicted on the basis of key genes.

Key gene	GZMB	FASLG	CCL4
Natural herbs	digenea simplex	rhizoma Zingiberis	millettia reticulata
	herba Cistanches	fagopyrum esculentum	rhamnus davurica
	mangifera indica	fructus Choerospondiatis	pericarpium papaveris
	eucalyptus viminalis	fructus Leonuri	cassia mimosoides
	folium camelliae sinensis	radix Platycodi	verbascum thapsus

**TABLE 5:** Candidate ingredients.

Key gene	GZMB		FASLG	CCL4
Candidate ingredients	Echinacoside	Kainic acid	6-shogaol	Rottlerin
	α-tocopherol	Lysine acid	Acacetin	Morphine
	βetaine	Tea polyphenols	Adeninenucleoside	
	Carotene	TellimagrandinII	Arsenic	
	Casuariin	Tocopherol	Gallic acid	
	Ethylparaben	Vanilloid	Geraniin	
	Vanilloid		Morphine	
	Soleucine		Progesterone	
	Eugenol		Ursolic acid	
	D-Chiro-Inositol		α-tocopherol	
	Hydroxybenzoic acid		Tocopherol	

### Immune cell infiltration after echinacoside intervention detected by flow cytometry

The frequency of infiltrated immune cells in each group were detected by flow cytometry (Figures 3E,F). The percentage of infiltrating NK cells significantly increased after swimming. LE and SE groups both reduced the percentage of infiltrating NK cells with model group. Long-term supplementation with echinacoside restricted the infiltration of NK cells more significantly than short-term supplementation with echinacoside. However, in contrast, the percentage of DCs was significantly increased after swimming. Long-term or short-term supplementation with echinacoside did not significantly decrease the percentage of DCs after swimming.

### Expression of key genes after echinacoside intervention detected by quantitative reverse transcription PCR

The mRNA levels of key genes were determined by quantitative reverse transcription PCR, which showed that the mRNA levels of all key genes were significantly increased after swimming (Figure 3G). Both long-term or short-term supplementation with echinacoside significantly reduced the expression level of key genes. *GZMB*, *PRF1*, and *CCL4* were markedly downregulated in the SE group compared with the LE group.

## Discussion

Our analysis of immune cell infiltration in the peripheral blood before and 30 min after exercise showed that NK cells showed a higher rate of infiltration post exercise, whereas naive B cells and activated DCs showed a low rate of infiltration (see Figure 1B). DCs, the most effective antigen-presenting cells, play an important role in immune homeostasis, bridging the gap between innate and adaptive immunity (29). They rapidly perform uptake and presentation functions when foreign antigens are present, activating T cells to produce an immune clearance response and inducing immune tolerance in the presence of self-antigens (30). Naive B cells are immature B cells that play an immunomodulatory role in inflammation by secreting cytokines and other pro-inflammatory indicators (31). Both of these cells are adaptive immune cells. NK cells are innate immune cells that determine the inflammatory microenvironment and are important in the removal of foreign infectious agents and own necrotic tissue (32). Therefore, the immune infiltration results do not signify an increase in immunity after 30 min of exercise but a sterile inflammation resulting from exercise-induced injury. We found that 53 genes were upregulated and 8 genes were downregulated post exercise (see Figures 1C,D). Notably, a PPI analysis of these differentially expressed genes showed that four of the top five genes (*PRF1*, *GZMB*, *CCL4*, and *FASLG*) were immune-related genes (see Figure 2F). GMZB, PRF1, and FASLG are key cytotoxic proteins in the secretory lysosomes in NK cells and typically produce cytotoxic effects in the inflammation zone (33). Thus, the findings suggest that the women developed immune dysregulation after 30 min of exercise, which was primarily associated with abnormally high levels of NK cells.

PRF1 is a potent component of NK cells; *PRF1* expression levels are positively correlated with the inflammatory toxic effects of NK cells, and PRF1 targeting regulates (enhances or decreases) the activity and function of NK cells (34–37). Thus, the high expression of *PRF1* after exercise is consistent with the high rate of NK cell infiltration. GZMB is a component of NK cell endolytic granules and is known for its apoptosis-promoting function (38, 39). It is an important mediator of the inflammatory response, and some studies have found that silencing *GZMB* reduces tissue damage caused by inflammation, for example, in a rat model of rheumatoid arthritis, *GZMB* silencing significantly reduced the degree of swelling in the ankle joint and reduced joint soft tissue damage (40, 41). FASLG, a member of the tumor necrosis factor family, is a transmembrane protein that forms an apoptotic cascade with FAS that is important for cell proliferation homeostasis (42, 43). This factor is widely involved in the inflammatory response and is highly expressed in inflammatory injury responses such as neurological injury, acute lung injury, kidney injury, and traumatic brain injury, and its expression is significantly associated with the extent of acute burns (44–47). Similar to *PRF1*, the high *GZMB* and *FASLG* expression was consistent with a high infiltration rate of NK cells, suggesting an inflammatory response following exercise. CCL4 is also an inflammatory chemokine that mediates the inflammatory immune response by recruiting lymphocytes, NK cells, and eosinophils, among others (48, 49). Several previous studies have found that the expression of this chemokine is significantly elevated in the inflammatory immune response following exercise (50, 51). It has also been found that CCL4 is a key mediator of neuroinflammatory pain following nerve injury (52). Overall, the high expression of these key genes reflects the fact that inflammatory damage does occur after acute exercise.

Notably, we predicted 26 natural immunomodulatory compounds on the basis of these immune infiltration findings. Echinacoside is a key natural component of the traditional Chinese medicinal herb *Herba Cistanches*. This compound remains little studied in the field of exercise, but it is widely used in traditional Chinese medicine because it is believed to play a key role in regulating human body functions. Interestingly, the substance is homologous to food and medicine; therefore, we intended to investigate whether it regulates inflammation after sports-related injury and further examine whether it is a potential sports supplement. Therefore, we selected this natural ingredient, echinacoside, as the optimal candidate compound and as the experimentally verified ingredient. Firstly, we verified the tightness of binding between echinacoside and GZMB using molecular docking and we found a minimum binding free energy of −8.0kcal/mol between them, which indicating a very tight binding. Furthermore, although echinacoside are not among the other key genetically predicted natural ingredients, we wanted to explore whether this component could bind tightly to key genes other than GZMB, such as FASLG. Surprisingly, we found that the binding free energy of echinacoside to FASLG and CCL4 was also very low, at −7.2kcal/mol and −6.9kcal/mol, respectively, suggesting that this natural ingredient also binds tightly to FASLG and CCL4. Finally, Experiments in mice were used to verify the modulatory effect of echinacoside on immune imbalance after sports-related injury. Remarkably, the flow cytometry results showed that mice gavaged with echinacoside had significantly lower levels of NK cells in their peripheral blood; the LE group showed the lowest NK cell levels, equivalent to those in the NC group, followed by the SE group, whereas the Model group, which was not supplemented with echinacoside, showed the highest levels of NK cells in the peripheral blood (see Figure 3F). However, for DCs, there was no statistical difference in the DC levels between the Model, LE, and SE groups, which all showed significant higher DC levels than those in the NC group. On the basis of this finding, we analyzed the factors affecting the expression levels of DCs in peripheral blood. DCs maintain overall immune homeostasis through their own active or passive regulation during an inflammatory response occurring after exercise; for example, in tissue damage inflammation, vimentin prevents autoimmune damage by inhibiting DCs (53). The mode of energy supply is another important factor affecting DC expression. Hypoxia or changes in the nutritional status affect DC metabolism, aerobic glycolysis significantly promotes DC activation, and inhibition of glycolytic efficiency directly prevents DC activation (54, 55). It is difficult to identify which factor most influences the function of DCs, but these factors together lead to the dynamic regulation of DCs.

Furthermore, we examined the expression levels of key genes (*GZMB*, *PRF1*, *FASLG*, and *CCL4*) in each mouse group by RT-qPCR and found that the expression levels of these genes in the LE and SE groups were similar to those in the NC group, significantly lower than those in the Model group, and the lowest in the SE group (see Figure 3G). Overall, the flow cytometry results for NK cells were consistent with the expression levels of key genes. However, unexpectedly, the expression of key genes was lower in the SE group than in the LE group. We then reviewed the pharmacokinetics of echinacoside and found that this natural active ingredient is extremely rapidly absorbed in the gastrointestinal tract, with a peak blood concentration time of 15 min and a short half-life of 74 min (56). The changes in the expression levels of these key genes intervened by echinacoside are relatively consistent with the pharmacokinetics of echinacoside. This may therefore explain the lowest expression of key genes in the SE group. Therefore, our preliminary findings suggest that echinacoside administration for a longer period is more effective in regulating peripheral blood NK cell levels after exercise and that this natural supplement is relatively more effective in reducing inflammation when administered 30 min before exercise.

In summary, echinacoside effectively modulates the inflammatory immune response following exercise, primarily by regulating NK cell levels. We report, for the first time to our knowledge, that echinacoside, a promising natural compound, may be used as a dietary supplement to reduce inflammatory damage due to excessive NK cell infiltration following exercise by reducing the elevated NK cell levels and lowering the levels of NK cell-associated cytotoxic proteins such as GZMB, PRF1, FASLG, and CCL4 in the peripheral blood.

**FIGURE 4:**
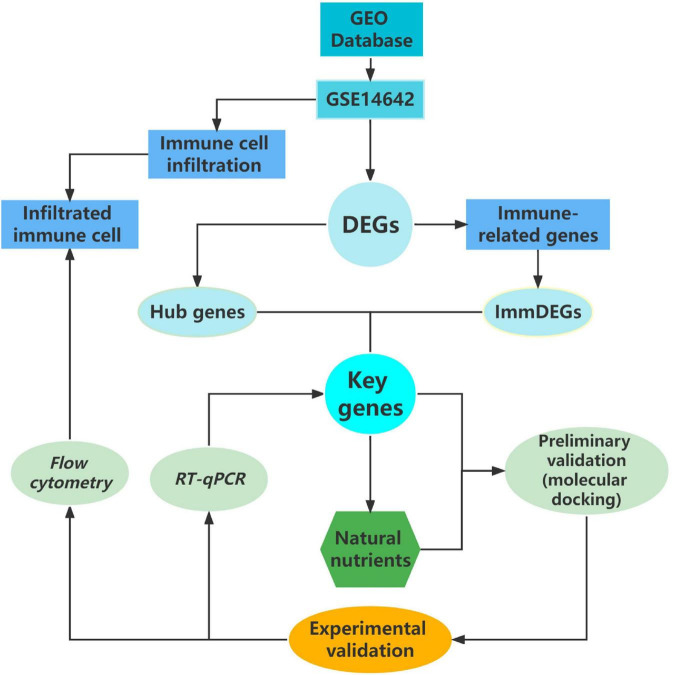
Flow chart of research methodology.

In this study, our approach has pioneered a very novel screening model for natural nutritional candidates, a systematic model that allows for rapid screening of a number of potential target natural compounds, greatly improving research efficiency (see Figure 4). The results of our study, echinacoside, which can bring preventive and therapeutic effects to patients with sports injuries, are of great clinical application. Particularly in the context of the apparent underdevelopment of anti-inflammatory dietary supplements for the prevention and mitigation of sports injuries, both our research model and echinacoside show strong potential in this area.

## Data availability statement

The original contributions presented in this study are included in the article/Supplementary material, further inquiries can be directed to the corresponding author.

## Ethics statement

The animal study was reviewed and approved by Animal Care and Use Committee of Affiliated Hospital of Guangzhou University of Chinese Medicine.

## Author contributions

FL and RY designed and planned the study and identified, and selected original studies. FJ and DX drafted the manuscript. FL and YS revised the final manuscript. LL and RL performed most experiments with some help from FL and RY. FL performed all the bioinformatics analyses. LX and MT contributed to data analysis. FL and ZZ provided advice during the study and manuscript preparation. All authors read and approved the final manuscript.

## References

[1] Chaput JP Willumsen J Bull F Chou R Ekelund U Firth J 2020 WHO guidelines on physical activity and sedentary behaviour for children and adolescents aged 5-17 years: summary of the evidence. *Int J Behav Nutr Phys Act.* (2020) 17:141. 10.1186/s12966-020-01037-z 33239009 PMC7691077

[2] Smith AD Crippa A Woodcock J Brage S. Physical activity and incident type 2 diabetes mellitus: a systematic review and dose–response meta-analysis of prospective cohort studies. *Diabetologia.* (2016) 59:2527–45. 10.1007/s00125-016-4079-0 27747395 PMC6207340

[3] Börjesson M Onerup A Lundqvist S Dahlöf B. Physical activity and exercise lower blood pressure in individuals with hypertension: narrative review of 27 RCTs. *Br J Sports Med.* (2016) 50:356–61. 10.1136/bjsports-2015-095786 26787705

[4] Jeong SW Kim SH Kang SH Kim HJ Yoon CH Youn TJ Mortality reduction with physical activity in patients with and without cardiovascular disease. *Eur Heart J.* (2019) 40:3547–55. 10.1093/eurheartj/ehz564 31504416 PMC6855138

[5] Fiuza-Luces C Santos-Lozano A Joyner M Carrera-Bastos P Picazo O Zugaza JL Exercise benefits in cardiovascular disease: beyond attenuation of traditional risk factors. *Nat Rev Cardiol.* (2018) 15:731–43. 10.1038/s41569-018-0065-1 30115967

[6] Schmitz KH Campbell AM Stuiver MM Pinto BM Schwartz AL Morris GS Exercise is medicine in oncology: engaging clinicians to help patients move through cancer. *CA Cancer J Clin.* (2019) 69:468–84. 10.3322/caac.21579 31617590 PMC7896280

[7] Valenzuela PL Castillo-García A Morales JS de la Villa P Hampel H Emanuele E Exercise benefits on Alzheimer’s disease: state-of-the-science. *Ageing Res Rev.* (2020) 62:101108. 10.1016/j.arr.2020.101108 32561386

[8] Chastin SFM Abaraogu U Bourgois JG Dall PM Darnborough J Duncan E Effects of regular physical activity on the immune system, vaccination and risk of community-acquired infectious disease in the general population: systematic review and meta-analysis. *Sports Med.* (2021) 51:1673–86. 10.1007/s40279-021-01466-1 33877614 PMC8056368

[9] Piercy KL Troiano RP Ballard RM Carlson SA Fulton JE Galuska DA The physical activity guidelines for Americans. *JAMA.* (2018) 320:2020–8. 10.1001/jama.2018.14854 30418471 PMC9582631

[10] Mohr M Draganidis D Chatzinikolaou A Barbero-Álvarez JC Castagna C Douroudos I Muscle damage, inflammatory, immune and performance responses to three football games in 1 week in competitive male players. *Eur J Appl Physiol.* (2016) 116:179–93. 10.1007/s00421-015-3245-2 26377004

[11] Lee EC Fragala MS Kavouras SA Queen RM Pryor JL Casa DJ. Biomarkers in sports and exercise: tracking health, performance, and recovery in athletes. *J Strength Cond Res.* (2017) 31:2920–37. 10.1519/jsc.0000000000002122 28737585 PMC5640004

[12] Matava MJ. Injectable nonsteroidal anti-inflammatory drugs in sport. *Clin J Sport Med.* (2018) 28:443–50. 10.1097/jsm.0000000000000602 29771750

[13] Cheung K Hume P Maxwell L. Delayed onset muscle soreness : treatment strategies and performance factors. *Sports Med.* (2003) 33:145–64. 10.2165/00007256-200333020-00005 12617692

[14] O’Connor S McCaffrey N Whyte E Moran K Lacey P. Nonsteroidal anti-inflammatory drug use, knowledge, and behaviors around their use and misuse in Irish collegiate student-athletes. *Phys Sports Med.* (2019) 47:318–22. 10.1080/00913847.2018.1553468 30479177

[15] Bedi A Trinh TQ Olszewski AM Maerz T Ramme AJ. Nonbiologic injections in sports medicine. *JBJS Rev.* (2020) 8:e0052. 10.2106/jbjs.Rvw.19.00052 32224626

[16] Smith-Ryan AE Hirsch KR Saylor HE Gould LM Blue MN. Nutritional considerations and strategies to facilitate injury recovery and rehabilitation. *J Athl Train.* (2020) 55:918–30. 10.4085/1062-6050-550-19 32991705 PMC7534941

[17] Rawson ES Miles MP Larson-Meyer DE. Dietary supplements for health, adaptation, and recovery in athletes. *Int J Sport Nutr Exerc Metab.* (2018) 28:188–99. 10.1123/ijsnem.2017-0340 29345167

[18] Fernández-Lázaro D Mielgo-Ayuso J Seco Calvo J Córdova Martínez A Caballero García A Fernandez-Lazaro CI. Modulation of exercise-induced muscle damage, inflammation, and oxidative markers by curcumin supplementation in a physically active population: a systematic review. *Nutrients.* (2020) 12:501. 10.3390/nu12020501 32075287 PMC7071279

[19] Huang CC Lee MC Ho CS Hsu YJ Ho CC Kan NW. Protective and recovery effects of resveratrol supplementation on exercise performance and muscle damage following acute plyometric exercise. *Nutrients.* (2021) 13:3217. 10.3390/nu13093217 34579095 PMC8469037

[20] Borghi SM Pinho-Ribeiro FA Fattori V Bussmann AJ Vignoli JA Camilios-Neto D Quercetin inhibits peripheral and spinal cord nociceptive mechanisms to reduce intense acute swimming-induced muscle pain in mice. *PLoS One.* (2016) 11:e0162267. 10.1371/journal.pone.0162267 27583449 PMC5008838

[21] Lu Y Deng B Xu L Liu H Song Y Lin F. Effects of *Rhodiola rosea* supplementation on exercise and sport: a systematic review. *Front Nutr.* (2022) 9:856287. 10.3389/fnut.2022.856287 35464040 PMC9021834

[22] Shen S Kong J Qiu Y Yang X Wang W Yan L. Identification of core genes and outcomes in hepatocellular carcinoma by bioinformatics analysis. *J Cell Biochem.* (2019) 120:10069–81. 10.1002/jcb.28290 30525236

[23] Liu Y Zhang X Zhang J Tan J Li J Song Z. Development and validation of a combined ferroptosis and immune prognostic classifier for hepatocellular carcinoma. *Front Cell Dev Biol.* (2020) 8:596679. 10.3389/fcell.2020.596679 33425905 PMC7785857

[24] Sun Q Li X Xu M Zhang L Zuo H Xin Y Differential expression and bioinformatics analysis of circRNA in non-small cell lung cancer. *Front Genet.* (2020) 11:586814. 10.3389/fgene.2020.586814 33329727 PMC7732606

[25] Subramanian A Tamayo P Mootha VK Mukherjee S Ebert BL Gillette MA Gene set enrichment analysis: a knowledge-based approach for interpreting genome-wide expression profiles. *Proc Natl Acad Sci U.S.A.* (2005) 102:15545–50. 10.1073/pnas.0506580102 16199517 PMC1239896

[26] Fang S Dong L Liu L Guo J Zhao L Zhang J HERB: a high-throughput experiment- and reference-guided database of traditional Chinese medicine. *Nucleic Acids Res.* (2021) 49:D1197–206. 10.1093/nar/gkaa1063 33264402 PMC7779036

[27] Liu J Yang L Dong Y Zhang B Ma X. Echinacoside, an inestimable natural product in treatment of neurological and other disorders. *Molecules.* (2018) 23:1213. 10.3390/molecules23051213 29783690 PMC6100060

[28] Radom-Aizik S Zaldivar F Jr Leu SY Cooper DM. A brief bout of exercise alters gene expression and distinct gene pathways in peripheral blood mononuclear cells of early- and late-pubertal females. *J Appl Physiol.* (2009) 107:168–75. 10.1152/japplphysiol.00121.2009 19407257 PMC2711785

[29] Balan S Saxena M Bhardwaj N. Dendritic cell subsets and locations. *Int Rev Cell Mol Biol.* (2019) 348:1–68. 10.1016/bs.ircmb.2019.07.004 31810551

[30] Waisman A Lukas D Clausen BE Yogev N. Dendritic cells as gatekeepers of tolerance. *Semin Immunopathol.* (2017) 39:153–63. 10.1007/s00281-016-0583-z 27456849

[31] Frasca D Diaz A Romero M Garcia D Blomberg B. B cell immunosenescence. *Annu Rev Cell Dev Biol.* (2020) 36:551–74. 10.1146/annurev-cellbio-011620-034148 33021823 PMC8060858

[32] Li Y Wang F Imani S Tao L Deng Y Cai Y. Natural killer cells: friend or foe in metabolic diseases? *Front Immunol.* (2021) 12:614429. 10.3389/fimmu.2021.614429 33717101 PMC7943437

[33] Lettau M Kabelitz D Janssen O. Lysosome-related effector vesicles in T lymphocytes and NK cells. *Scand J Immunol.* (2015) 82:235–43. 10.1111/sji.12337 26118957

[34] Romero CA Sánchez IP Gutierrez-Hincapié S Álvarez-Álvarez JA Pereañez JA Ochoa R A novel pathogenic variant in PRF1 associated with hemophagocytic lymphohistiocytosis. *J Clin Immunol.* (2015) 35:501–11. 10.1007/s10875-015-0169-x 25975970

[35] Huang Q Ding J Gong M Wei M Zhao Q Yang J. Effect of miR-30e regulating NK cell activities on immune tolerance of maternal-fetal interface by targeting PRF1. *Biomed Pharmacother.* (2019) 109:1478–87. 10.1016/j.biopha.2018.09.172 30551399

[36] Lu CC Wu TS Hsu YJ Chang CJ Lin CS Chia JH NK cells kill mycobacteria directly by releasing perforin and granulysin. *J Leukoc Biol.* (2014) 96:1119–29. 10.1189/jlb.4A0713-363RR 25139289

[37] Chang CJ Chen YY Lu CC Lin CS Martel J Tsai SH *Ganoderma lucidum* stimulates NK cell cytotoxicity by inducing NKG2D/NCR activation and secretion of perforin and granulysin. *Innate Immun.* (2014) 20:301–11. 10.1177/1753425913491789 23803412

[38] Oboshi W Watanabe T Hayashi K Nakamura T Yukimasa N. QPY/RAH haplotypes of the GZMB gene are associated with natural killer cell cytotoxicity. *Immunogenetics.* (2018) 70:29–36. 10.1007/s00251-017-1014-6 28653095

[39] Turner CT Lim D Granville DJ. Granzyme B in skin inflammation and disease. *Matrix Biol.* (2019) 75-76:126–40. 10.1016/j.matbio.2017.12.005 29247692

[40] Velotti F Barchetta I Cimini FA Cavallo MG. Granzyme B in inflammatory diseases: apoptosis, inflammation, extracellular matrix remodeling, epithelial-to-mesenchymal transition and fibrosis. *Front Immunol.* (2020) 11:587581. 10.3389/fimmu.2020.587581 33262766 PMC7686573

[41] Bao CX Chen HX Mou XJ Zhu XK Zhao Q Wang XG. GZMB gene silencing confers protection against synovial tissue hyperplasia and articular cartilage tissue injury in rheumatoid arthritis through the MAPK signaling pathway. *Biomed Pharmacother.* (2018) 103:346–54. 10.1016/j.biopha.2018.04.023 29669300

[42] Ji W Xin Y Zhang L Liu X. ALG2 influences T cell apoptosis by regulating FASLG intracellular transportation. *Biochem J.* (2020) 477:3105–21. 10.1042/bcj20200028 32766719

[43] Magerus A Bercher-Brayer C Rieux-Laucat F. The genetic landscape of the FAS pathway deficiencies. *Biomed J.* (2021) 44:388–99. 10.1016/j.bj.2021.06.005 34171534 PMC8514852

[44] Li Y Sun Y Cai M Zhang H Gao N Huang H FAS Ligand Gene (FASLG) plays an important role in nerve degeneration and regeneration after rat sciatic nerve injury. *Front Mol Neurosci.* (2018) 11:210. 10.3389/fnmol.2018.00210 29970988 PMC6018423

[45] Jiang H Wang S Hou L Huang JA Su B. Resveratrol inhibits cell apoptosis by suppressing long noncoding RNA (lncRNA) XLOC_014869 during lipopolysaccharide-induced acute lung injury in rats. *J Thorac Dis.* (2021) 13:6409–26. 10.21037/jtd-21-1113 34992821 PMC8662516

[46] Ortiz A Lorz C Egido J. The FAS ligand/FAS system in renal injury. *Nephrol Dial Transplant.* (1999) 14:1831–4. 10.1093/ndt/14.8.1831 10462254

[47] Beer R Franz G Schöpf M Reindl M Zelger B Schmutzhard E Expression of FAS and FAS ligand after experimental traumatic brain injury in the rat. *J Cereb Blood Flow Metab.* (2000) 20:669–77. 10.1097/00004647-200004000-00004 10779011

[48] Mukaida N Sasaki SI Baba T. CCL4 signaling in the tumor microenvironment. *Adv Exp Med Biol.* (2020) 1231:23–32. 10.1007/978-3-030-36667-4_3 32060843

[49] Kobayashi Y Konno Y Kanda A Yamada Y Yasuba H Sakata Y Critical role of CCL4 in eosinophil recruitment into the airway. *Clin Exp Allergy.* (2019) 49:853–60. 10.1111/cea.13382 30854716

[50] Muders K Pilat C Deuster V Frech T Krüger K Pons-Kühnemann J Effects of Traumeel (Tr14) on recovery and inflammatory immune response after repeated bouts of exercise: a double-blind RCT. *Eur J Appl Physiol.* (2017) 117:591–605. 10.1007/s00421-017-3554-8 28224232

[51] Peake JM Roberts LA Figueiredo VC Egner I Krog S Aas SN The effects of cold water immersion and active recovery on inflammation and cell stress responses in human skeletal muscle after resistance exercise. *J Physiol.* (2017) 595:695–711. 10.1113/jp272881 27704555 PMC5285720

[52] Saika F Kiguchi N Kobayashi Y Fukazawa Y Kishioka S. CC-chemokine ligand 4/macrophage inflammatory protein-1β participates in the induction of neuropathic pain after peripheral nerve injury. *Eur J Pain.* (2012) 16:1271–80. 10.1002/j.1532-2149.2012.00146.x 22528550

[53] Yu MB Guerra J Firek A Langridge WHR. Extracellular vimentin modulates human dendritic cell activation. *Mol Immunol.* (2018) 104:37–46. 10.1016/j.molimm.2018.09.017 30399492 PMC6497527

[54] O’Neill LA Pearce EJ. Immunometabolism governs dendritic cell and macrophage function. *J Exp Med.* (2016) 213:15–23. 10.1084/jem.20151570 26694970 PMC4710204

[55] Kelly B O’Neill LA. Metabolic reprogramming in macrophages and dendritic cells in innate immunity. *Cell Res.* (2015) 25:771–84. 10.1038/cr.2015.68 26045163 PMC4493277

[56] Jia C Shi H Wu X Li Y Chen J Tu P. Determination of echinacoside in rat serum by reversed-phase high-performance liquid chromatography with ultraviolet detection and its application to pharmacokinetics and bioavailability. *J Chromatogr B Analyt Technol Biomed Life Sci.* (2006) 844:308–13. 10.1016/j.jchromb.2006.07.040 16931184

