# Supplementary material for: Effects of a natural nutritional supplement on immune cell infiltration and immune gene expression in exercise-induced injury
Source: Front Nutr. 2022 Sep 16;9:987545. doi: 10.3389/fnut.2022.987545 (PMC9523794; doi:10.3389/fnut.2022.987545)
Supplement: Supplementary file 1 [file Data_Sheet_1.docx]

logFC AveExpr t P.Value adj.P.Val B change

TAGAP 0.473356693 10.74774624 8.412163753 2.88E-10 4.50E-06 13.34438717 stable

PPM1B 0.308510093 11.39635841 8.317494925 3.83E-10 4.50E-06 13.06184221 stable

SLC38A2 0.431257412 12.64643727 8.092670525 7.57E-10 5.94E-06 12.38685129 stable

ZCCHC10 -0.455402921 8.486094986 -7.578077283 3.68E-09 2.17E-05 10.8222695 stable

FASLG 1.985010435 6.072325873 7.451868523 5.45E-09 2.25E-05 10.43476154 up

RPRD1B 0.341492548 7.932662049 7.434768896 5.75E-09 2.25E-05 10.38215506 stable

RSBN1 0.434936208 10.43898497 7.322009429 8.17E-09 2.47E-05 10.03465783 stable

ZBTB11 0.367113024 10.6144486 7.312453049 8.41E-09 2.47E-05 10.00516101 stable

SMAD7 0.988915296 8.699985569 7.24159676 1.05E-08 2.74E-05 9.786237628 stable

ATP2B4 0.531839025 8.241848021 7.17293464 1.30E-08 3.06E-05 9.573738928 stable

ZBTB24 -0.800379337 7.151136067 -7.061816697 1.84E-08 3.94E-05 9.229147792 stable

ZBTB21 0.59379726 8.056455758 7.006701332 2.19E-08 4.30E-05 9.057926206 stable

CCDC117 0.497076337 10.2766222 6.902034148 3.05E-08 5.52E-05 8.732256916 stable

SYF2 -1.175612179 7.588060794 -6.858971433 3.49E-08 5.87E-05 8.598086569 down

S1PR5 1.555326318 8.698067083 6.805550164 4.13E-08 5.90E-05 8.431504915 up

ATF4 -0.306629032 12.22673661 -6.799084686 4.22E-08 5.90E-05 8.411333938 stable

KBTBD2 0.269262244 9.473394142 6.795725803 4.27E-08 5.90E-05 8.400854084 stable

LOC101929889 /// SPN 0.537678023 10.19818399 6.755699296 4.84E-08 6.22E-05 8.275927275 stable

ZBED5-AS1 -0.408655521 9.06639282 -6.744064575 5.02E-08 6.22E-05 8.239599785 stable

SH2D1B 1.514674336 8.466348674 6.721030403 5.40E-08 6.35E-05 8.167660966 up

YES1 0.915718535 7.877328959 6.653739422 6.68E-08 7.19E-05 7.95736854 stable

ENPP4 0.827974881 9.446521322 6.651736526 6.73E-08 7.19E-05 7.951106338 stable

SF3B6 -1.067937146 4.42290602 -6.587787295 8.24E-08 8.42E-05 7.751081983 down

BTG1 -0.848093077 8.721735308 -6.541647689 9.53E-08 9.22E-05 7.606670993 stable

TBX21 1.156876794 10.33261728 6.532920132 9.80E-08 9.22E-05 7.579346787 up

CASP10 0.38404481 8.521849908 6.503288176 1.08E-07 9.74E-05 7.486557095 stable

MED14 0.243480659 9.845559486 6.464353362 1.22E-07 9.76E-05 7.364596405 stable

ENPP5 1.738855658 5.594954901 6.461171837 1.23E-07 9.76E-05 7.354628599 up

LONRF1 -1.141521475 7.594032837 -6.460862375 1.23E-07 9.76E-05 7.353659033 down

TFRC -0.256748228 10.69173638 -6.442155417 1.31E-07 9.76E-05 7.295043994 stable

NCBP2-AS2 -0.298600057 9.869060927 -6.438172451 1.32E-07 9.76E-05 7.282562879 stable

ZNF644 0.423502619 8.424224898 6.437244693 1.33E-07 9.76E-05 7.279655577 stable

ELOVL6 1.498986391 4.10406869 6.416803327 1.42E-07 0.000100044 7.215593305 up

PLEKHF1 1.130812392 8.814547813 6.410392024 1.45E-07 0.000100044 7.195498505 up

FEM1B 0.508655105 8.58552422 6.399435806 1.50E-07 0.000100628 7.161156469 stable

TGFBR1 0.681251415 7.490877186 6.385811178 1.56E-07 0.000101493 7.118446596 stable

LOC285812 0.767184839 9.879791772 6.379256053 1.60E-07 0.000101493 7.097896447 stable

ABHD17B 0.362875157 8.556020946 6.295672106 2.08E-07 0.000128905 6.835791253 stable

LPCAT1 0.562386233 11.30342411 6.264573744 2.30E-07 0.000138661 6.738243711 stable

ANKRD46 0.630353807 8.978821064 6.247053719 2.43E-07 0.000142945 6.68328264 stable

ZFAND2A -0.446134307 9.235681193 -6.223185049 2.62E-07 0.000150465 6.608400447 stable

NKG7 0.911249938 13.75266391 6.210896964 2.73E-07 0.000152741 6.569847551 stable

ZBTB6 1.138644207 4.779878082 6.193901059 2.88E-07 0.000157482 6.516522378 up

PRF1 1.106748062 12.8502094 6.179281265 3.02E-07 0.000159832 6.470650872 up

NUP133 0.336874964 9.308406985 6.174966811 3.06E-07 0.000159832 6.457113497 stable

CX3CR1 1.262942463 4.913564853 6.165147067 3.15E-07 0.000161323 6.426301969 up

PRR5L 0.990465383 8.920306417 6.144037314 3.37E-07 0.000168111 6.360064511 stable

EOMES 0.96433684 10.47348306 6.138836365 3.43E-07 0.000168111 6.343744982 stable

FUT11 0.730536136 10.03239843 6.074186417 4.22E-07 0.000201711 6.140886821 stable

NUFIP2 0.275861083 10.32996966 6.068806856 4.29E-07 0.000201711 6.124007307 stable

EIF1 -0.274870296 14.16947562 -6.038024469 4.73E-07 0.00021813 6.02742399 stable

LGR6 1.55141977 7.478343467 6.015980525 5.07E-07 0.000229499 5.958262641 up

LOC100130872 /// SPON2 1.29462882 11.46038142 6.002100207 5.30E-07 0.00023535 5.91471635 up

ERCC6 0.593748839 5.685874699 5.973743086 5.80E-07 0.000252832 5.825759011 stable

ADAMTS1 2.010094203 5.148709927 5.936435913 6.54E-07 0.000279566 5.708741457 up

LOC102724017 -0.438536229 7.254568287 -5.921098848 6.86E-07 0.000288325 5.660641705 stable

MATK 0.700120104 8.969951575 5.869233172 8.10E-07 0.000334166 5.498015272 stable

NIPBL 0.188816744 10.24388098 5.859741997 8.35E-07 0.000338486 5.468261711 stable

PHF13 0.482517678 9.020501956 5.850441855 8.60E-07 0.000342756 5.439109109 stable

RAB8B 0.438976681 10.84529329 5.839430538 8.90E-07 0.000349077 5.404595376 stable

ACBD5 0.413518894 7.456137296 5.784003432 1.06E-06 0.000409661 5.230915858 stable

IL4R -0.497311879 11.10011174 -5.765286309 1.13E-06 0.000423926 5.172287193 stable

GZMA 0.861276898 12.95348336 5.763129003 1.14E-06 0.000423926 5.165530485 stable

IGIP 0.435513612 8.376524685 5.747455899 1.19E-06 0.000438661 5.116446896 stable

ARHGEF3 0.414528876 12.24579099 5.739836874 1.22E-06 0.000442521 5.092589434 stable

TGFBR3 0.94633232 9.965292643 5.729281703 1.26E-06 0.000450714 5.059541471 stable

TRIM13 -0.673392847 7.771525325 -5.714878715 1.32E-06 0.000464825 5.014452786 stable

ARNTL 0.345960557 10.02192012 5.708677899 1.35E-06 0.000465505 4.995043522 stable

RHOBTB3 1.297957777 4.828947162 5.705181981 1.37E-06 0.000465505 4.984101558 up

P2RX5 -0.668525826 10.63783497 -5.691214679 1.43E-06 0.000479721 4.940389823 stable

CST7 0.996169027 11.87137929 5.668815015 1.53E-06 0.000507923 4.870305204 stable

CHST12 0.563906275 9.728309058 5.657958378 1.59E-06 0.000516248 4.836344458 stable

MBD5 0.499993114 5.500555004 5.654980296 1.60E-06 0.000516248 4.827029605 stable

CHST2 0.712261496 11.11396118 5.645084179 1.65E-06 0.000520331 4.796079376 stable

SLK 0.361713659 9.415542089 5.638467694 1.69E-06 0.000520331 4.775388738 stable

RNF145 0.277688463 11.18951197 5.636259003 1.70E-06 0.000520331 4.768482319 stable

GPR56 1.506798716 8.784165986 5.635744393 1.70E-06 0.000520331 4.766873205 up

IL2RB 0.807421866 12.43485778 5.617600204 1.80E-06 0.000544192 4.710146827 stable

FBXO38 0.283900648 8.479076566 5.594346454 1.94E-06 0.000577792 4.637469445 stable

C19orf12 0.444141254 9.565480573 5.590811912 1.97E-06 0.000577792 4.626424972 stable

NMUR1 1.171102405 5.767418393 5.556465422 2.19E-06 0.000636535 4.51913609 up

MLC1 1.036688649 8.556370445 5.545135727 2.27E-06 0.000645468 4.483759453 up

GZMB 1.203763397 12.18068124 5.543814588 2.28E-06 0.000645468 4.479634709 up

KLF3 0.196881608 10.46784541 5.540645856 2.31E-06 0.000645468 4.469741976 stable

IRF2BPL 0.351359366 11.94549487 5.527051599 2.41E-06 0.000666049 4.4273075 stable

KLRF1 1.044965637 11.44813144 5.50369094 2.59E-06 0.000709049 4.354412806 up

ZNF322 0.583584621 8.057543309 5.489589558 2.71E-06 0.000733023 4.310426974 stable

KIR2DL3 1.569307666 7.421726492 5.484164809 2.76E-06 0.000737294 4.293509116 up

STK39 0.494204792 9.727533667 5.472176931 2.87E-06 0.000757312 4.256129895 stable

PMS2P5 -0.619411571 8.264015309 -5.465815443 2.92E-06 0.000764187 4.236298028 stable

RAP1GAP2 0.400574011 11.11669991 5.45158394 3.06E-06 0.000784662 4.19194119 stable

RSRC2 0.200899992 10.37616711 5.448462301 3.09E-06 0.000784662 4.182213463 stable

ADAM28 -0.700530409 9.865124332 -5.447168231 3.10E-06 0.000784662 4.178181048 stable

AK024119 /// CTC-378H22.2 -0.478750196 6.98030501 -5.43029018 3.27E-06 0.000819058 4.125598338 stable

PXN 0.248985546 10.97830401 5.425115753 3.33E-06 0.000823076 4.109481651 stable

RNF219 0.376988288 8.84562387 5.422117977 3.36E-06 0.000823076 4.100145397 stable

HSPA8 /// SNORD14C /// SNORD14D 0.182353854 15.0508105 5.394780537 3.66E-06 0.000882256 4.015035744 stable

PDGFD 1.48034368 7.704278293 5.393746925 3.68E-06 0.000882256 4.011818873 up

FAM78A /// LOC101927137 0.161957406 11.52949688 5.388198132 3.74E-06 0.000888859 3.994550947 stable

PIK3CG 0.415641865 9.407639963 5.376374727 3.88E-06 0.000912944 3.957764071 stable

CLDND2 0.89683668 4.425145363 5.372943164 3.93E-06 0.000912944 3.947089237 stable

APMAP 0.462032617 11.80930175 5.370362104 3.96E-06 0.000912944 3.939060725 stable

PPAPDC1B -0.55331336 8.916695435 -5.351877895 4.20E-06 0.000958669 3.881579864 stable

ZNFX1 0.238426702 10.34085476 5.331426638 4.48E-06 0.000984107 3.818013694 stable

NCR1 0.946325775 4.158901488 5.331196953 4.48E-06 0.000984107 3.817299987 stable

TMEM170A 0.223359714 10.28700744 5.328284394 4.52E-06 0.000984107 3.808250053 stable

SLC5A3 0.594168317 6.054918389 5.327634224 4.53E-06 0.000984107 3.806229933 stable

PPM1D 0.64169841 8.927709253 5.326423419 4.55E-06 0.000984107 3.802467977 stable

ZNF217 0.500201344 10.73487901 5.323059384 4.60E-06 0.000984107 3.792016598 stable

BCL10 -0.798997295 7.515591756 -5.322878861 4.60E-06 0.000984107 3.791455776 stable

TNFSF14 1.096705953 6.706311296 5.317808298 4.68E-06 0.000991042 3.775704411 up

EIF5 /// SNORA28 -1.16770914 7.856268215 -5.313175481 4.75E-06 0.00099672 3.761314755 down

CD247 0.604198837 12.37789152 5.293926067 5.04E-06 0.00105003 3.701545053 stable

PHF23 0.547994797 9.65191128 5.286744077 5.16E-06 0.001064633 3.679253047 stable

GPR174 0.55385219 7.939012085 5.284027341 5.21E-06 0.001064633 3.670821808 stable

ZKSCAN8 0.465542382 6.176819939 5.279794171 5.28E-06 0.001069701 3.657685697 stable

INSIG2 0.243002674 9.619573296 5.272141173 5.40E-06 0.001086576 3.633941413 stable

ZC3H10 0.760673656 6.553872832 5.263288706 5.56E-06 0.001107994 3.606482168 stable

NPC1 0.56517379 9.929986294 5.251558232 5.77E-06 0.00114025 3.570106646 stable

PTGDR 1.043987312 9.009367176 5.235466852 6.07E-06 0.001185221 3.520228824 up

AK6 /// TAF9 -0.231179709 10.89034145 -5.234068981 6.10E-06 0.001185221 3.51589704 stable

GOLGA4 0.236123489 10.25265249 5.226425868 6.25E-06 0.001197381 3.492215483 stable

FGFBP2 1.271145098 11.82211695 5.225661143 6.26E-06 0.001197381 3.489846351 up

PKIG -0.811459162 8.942852513 -5.222136645 6.33E-06 0.001201042 3.47892811 stable

ITPRIPL1 0.707205054 4.623390667 5.202523234 6.74E-06 0.001267679 3.41819127 stable

RAB9A 0.404991113 10.42336978 5.196460297 6.87E-06 0.001281959 3.399423774 stable

SETX 0.265572696 11.08072509 5.191205268 6.98E-06 0.001284805 3.383160058 stable

KLRD1 0.969469827 10.83170817 5.188866121 7.03E-06 0.001284805 3.375921548 stable

SEMA4C 0.498618537 8.12144794 5.18831543 7.05E-06 0.001284805 3.37421751 stable

DNAJB4 0.579346023 5.223751349 5.177582923 7.29E-06 0.00131393 3.341013312 stable

B3GNT2 0.432292588 9.341960053 5.176355376 7.32E-06 0.00131393 3.337216275 stable

KIR3DL1 /// KIR3DL2 1.616465348 7.351013053 5.166002989 7.56E-06 0.001347335 3.305200459 up

PPM1L 0.599441112 8.843527835 5.162321071 7.65E-06 0.00135285 3.293816405 stable

CASP3 0.331136623 9.765964041 5.153457574 7.87E-06 0.001380879 3.266417285 stable

TM2D3 0.329264505 10.15479371 5.141522547 8.17E-06 0.001423296 3.229536357 stable

WDR89 0.448607475 6.577608199 5.13264261 8.40E-06 0.001453 3.202105892 stable

LASP1 0.158544067 13.3205217 5.126474142 8.57E-06 0.001470755 3.183056237 stable

GPR65 0.483933692 11.24655337 5.110226731 9.02E-06 0.001536911 3.132900253 stable

NAPEPLD 0.486343126 7.560446161 5.098103077 9.37E-06 0.001585339 3.095493315 stable

PAFAH2 0.53214337 8.497568412 5.095647222 9.44E-06 0.001586254 3.087917892 stable

FADS3 -0.449195719 8.008837899 -5.092099568 9.55E-06 0.001592725 3.07697586 stable

ZNF225 0.470807643 4.750239773 5.081345374 9.88E-06 0.001636055 3.043815393 stable

FUS -1.335925748 7.650079826 -5.07013784 1.02E-05 0.001683034 3.00927112 down

LOC102724112 /// WIBG -0.276021287 8.335176241 -5.055493712 1.07E-05 0.001750276 2.964156391 stable

CMC1 0.50531099 9.786518949 5.050771501 1.09E-05 0.001759466 2.94961386 stable

IGHD -0.905028011 11.16390367 -5.049452622 1.09E-05 0.001759466 2.945552706 stable

WASF1 -0.500268566 6.087237481 -5.037948338 1.13E-05 0.00180341 2.910136979 stable

DERL1 0.226392274 10.5813037 5.037299374 1.13E-05 0.00180341 2.908139627 stable

PTPN4 0.643967652 10.68595666 5.035053879 1.14E-05 0.001804017 2.901228934 stable

XIAP 0.427736346 7.410568484 5.027118121 1.17E-05 0.001837324 2.876810883 stable

MYBL1 0.750168724 11.40996204 5.018441282 1.20E-05 0.001863998 2.850121323 stable

RASSF5 0.190520672 11.91772689 5.017914174 1.21E-05 0.001863998 2.848500258 stable

C16orf72 0.384924588 10.10108313 5.016246811 1.21E-05 0.001863998 2.84337269 stable

PARP6 -0.169274613 9.693231758 -4.991191616 1.31E-05 0.00200376 2.766363163 stable

CCDC59 -0.256228729 10.06944783 -4.983119997 1.35E-05 0.002042003 2.741571105 stable

ZNF227 0.283257536 8.21905037 4.980032679 1.36E-05 0.002048586 2.732090572 stable

KIR3DL1 1.522702061 7.429700238 4.978017764 1.37E-05 0.002048586 2.725903833 up

ARL4C 0.462691463 12.32704944 4.97355536 1.39E-05 0.002061784 2.712204009 stable

SNIP1 0.500324939 7.579357036 4.971946981 1.39E-05 0.002061784 2.70726683 stable

RAB33A 0.635191695 7.255453737 4.969678408 1.40E-05 0.002063557 2.700303644 stable

GFI1 0.712591096 7.991391087 4.958225283 1.46E-05 0.002121399 2.665159508 stable

RNF103 -1.080157522 6.460614073 -4.956925473 1.46E-05 0.002121399 2.661172103 down

CCL4 1.398127305 10.66325435 4.953397031 1.48E-05 0.002131882 2.650349074 up

SH2D2A 0.886300163 8.96438204 4.947960932 1.50E-05 0.002152212 2.633677782 stable

TKTL1 0.96378461 8.279935322 4.946491551 1.51E-05 0.002152212 2.629172197 stable

TIPARP 0.359264866 10.06372445 4.933124212 1.57E-05 0.002230934 2.588196941 stable

NCK1 0.189710264 10.35225126 4.923934375 1.62E-05 0.002282463 2.560041058 stable

PRPF38B 0.182173913 10.21867309 4.917748507 1.65E-05 0.002313342 2.541095226 stable

COLGALT2 1.067118873 5.815949086 4.91176116 1.68E-05 0.002343253 2.522762415 up

MFAP1 0.617716751 9.292288683 4.905650706 1.72E-05 0.002374541 2.504057758 stable

ADRB2 1.009594474 9.056297863 4.903030297 1.73E-05 0.002380133 2.496038031 up

SELPLG 0.219862937 12.533728 4.899861297 1.75E-05 0.002385958 2.486340633 stable

GIMAP4 0.313794564 12.9404982 4.898541982 1.75E-05 0.002385958 2.482303833 stable

GIMAP6 0.332687346 12.449206 4.894837406 1.78E-05 0.002393142 2.470969988 stable

SFT2D3 /// WDR33 0.313845157 8.049685387 4.893916511 1.78E-05 0.002393142 2.468152883 stable

SLC20A1 0.340373564 11.66577644 4.868105455 1.93E-05 0.002572673 2.389243386 stable

ERMP1 0.511629489 8.981947375 4.867202228 1.94E-05 0.002572673 2.386483769 stable

MED17 0.364957421 10.12851871 4.86413161 1.95E-05 0.002582938 2.377103037 stable

FAM179A 0.612647754 6.508526815 4.859799361 1.98E-05 0.002603587 2.363870347 stable

PTGER4 0.706253675 8.872816748 4.8539031 2.02E-05 0.002637359 2.345864834 stable

PYHIN1 1.009143501 7.375491794 4.851497717 2.03E-05 0.00264261 2.338520937 up

LYSMD3 0.496604671 9.760433791 4.848937155 2.05E-05 0.002649237 2.330704195 stable

ZNF184 0.537661063 8.097535729 4.846819645 2.06E-05 0.002652279 2.324240704 stable

EFHD2 0.367431195 12.71042217 4.84401507 2.08E-05 0.002661117 2.315681037 stable

PEX13 0.404250604 4.795107772 4.842054179 2.09E-05 0.002663022 2.309697013 stable

IFFO2 0.308515905 8.580361783 4.836368693 2.13E-05 0.002696241 2.292349922 stable

ARL13B 0.264866007 7.834063226 4.830955567 2.17E-05 0.002709115 2.275838314 stable

RGS3 0.477839585 9.568566952 4.830551373 2.17E-05 0.002709115 2.274605579 stable

TARDBP 0.184183186 11.19239412 4.829730458 2.18E-05 0.002709115 2.272101981 stable

MIB2 -0.409864911 7.451385508 -4.827088007 2.19E-05 0.002717219 2.264043815 stable

GNLY 0.830289328 13.81324996 4.822769445 2.22E-05 0.002726507 2.250876605 stable

HEG1 0.509710007 8.056303518 4.822647936 2.23E-05 0.002726507 2.250506169 stable

IGF2R 0.397508612 10.23036415 4.814532248 2.28E-05 0.002782078 2.225769374 stable

LOC100505501 0.60328769 7.703873625 4.80627409 2.34E-05 0.0028401 2.200608629 stable

AB074162 /// MIR181A2HG 0.937269456 4.18588109 4.797872429 2.40E-05 0.002900685 2.175021409 stable

CLASRP -0.266905539 10.72212042 -4.793188215 2.44E-05 0.002928419 2.160760394 stable

STAT4 0.480824018 10.96839093 4.788342556 2.48E-05 0.002957979 2.146011455 stable

RAB22A 0.240426875 9.707790454 4.785524112 2.50E-05 0.002969055 2.137434523 stable

ATAD2 0.328956238 7.372570061 4.767884023 2.64E-05 0.003121346 2.08378159 stable

FBXO30 0.583586833 9.024759088 4.763863791 2.67E-05 0.003144938 2.071560825 stable

BZRAP1 0.664170355 7.768876796 4.745300418 2.83E-05 0.003300259 2.015165278 stable

HS3ST3B1 0.745185831 6.578146126 4.745214578 2.83E-05 0.003300259 2.014904626 stable

ALG11 /// UTP14C 0.347028136 9.302563529 4.743067988 2.85E-05 0.003306049 2.008386914 stable

KIR3DL3 1.465655078 7.582929372 4.732306024 2.95E-05 0.003402048 1.975721617 up

YPEL5 -0.281537518 13.29119041 -4.714396202 3.12E-05 0.003579659 1.921403167 stable

BBS12 0.865018338 4.762859107 4.70746378 3.19E-05 0.003623095 1.900392302 stable

CEP78 0.50118585 8.845496985 4.707402672 3.19E-05 0.003623095 1.90020713 stable

STK10 0.190429329 11.98925079 4.705618658 3.21E-05 0.003625752 1.894801446 stable

GNPTAB 0.458131714 11.3583131 4.691851418 3.35E-05 0.003766322 1.853103933 stable

PHF8 0.130487803 9.291575219 4.685319283 3.42E-05 0.003825312 1.833331054 stable

SPRED1 0.763234106 5.468454805 4.673835767 3.54E-05 0.003945548 1.798588097 stable

PDLIM1 -0.516310966 11.62576713 -4.665029178 3.64E-05 0.004035881 1.771959613 stable

KCTD10 0.492092012 9.354915165 4.658110174 3.72E-05 0.004104199 1.75104815 stable

GNGT2 0.734916422 7.391095085 4.655816518 3.74E-05 0.004114222 1.744117843 stable

PUS10 0.46363695 5.741386362 4.649056627 3.82E-05 0.004181947 1.723698172 stable

FBRS -0.21339198 9.139460982 -4.646429483 3.85E-05 0.004196673 1.715764523 stable

ZNF267 0.563073881 7.738538548 4.644886003 3.87E-05 0.004197395 1.711103977 stable

WDR7 0.199122217 9.437090358 4.630012176 4.06E-05 0.004371859 1.666214268 stable

APOBEC3F /// APOBEC3G 0.368937955 10.71853054 4.628803989 4.07E-05 0.004371859 1.66256967 stable

TFDP2 0.55845096 8.254084584 4.625782132 4.11E-05 0.004377106 1.65345514 stable

ZNF41 0.345263435 6.755429599 4.625485984 4.11E-05 0.004377106 1.652561986 stable

ASUN 0.186421005 10.17839073 4.619023611 4.20E-05 0.004430139 1.633076083 stable

C9orf69 0.314546409 9.527595291 4.617675412 4.21E-05 0.004430139 1.629011839 stable

CKAP5 0.186428877 9.614524324 4.617253107 4.22E-05 0.004430139 1.627738838 stable

GIMAP7 0.547664686 12.02525328 4.608981611 4.33E-05 0.004506032 1.602811734 stable

FCHSD2 -0.363899002 7.508793005 -4.608904608 4.33E-05 0.004506032 1.602579736 stable

MIR7111 /// RPL10A -0.377317991 4.736344236 -4.607210964 4.35E-05 0.004509787 1.59747732 stable

CSE1L 0.414877508 8.534998187 4.604402606 4.39E-05 0.004529246 1.58901779 stable

MSMO1 0.394738156 9.021897404 4.593402122 4.54E-05 0.004663022 1.55589546 stable

SNRPA -0.175947864 10.80675272 -4.592184572 4.56E-05 0.004663022 1.552230816 stable

ZNF649 0.639285477 6.388609078 4.587739183 4.62E-05 0.004707176 1.538853214 stable

MAP4K2 -0.251361258 8.684965779 -4.582963686 4.69E-05 0.00475668 1.524486342 stable

MCCC1 -0.19077767 8.980533851 -4.579440972 4.74E-05 0.00475794 1.51389117 stable

AKR1C3 1.467388885 8.939277233 4.578944399 4.75E-05 0.00475794 1.512397829 up

PRPF4B 0.143426351 10.95993488 4.578726346 4.75E-05 0.00475794 1.511742092 stable

NFKBIA -0.53331452 12.67461376 -4.577011573 4.78E-05 0.004762984 1.506585694 stable

RAB11A -0.226183581 12.9330416 -4.56669087 4.93E-05 0.004896746 1.475562685 stable

GPR132 0.485481774 7.177008014 4.535024528 5.44E-05 0.005361955 1.380504626 stable

CACNA2D2 0.719231933 7.342102314 4.534600305 5.45E-05 0.005361955 1.37923249 stable

ANKRD12 0.210329266 10.41553355 4.532671572 5.48E-05 0.005371505 1.373449163 stable

PIK3R3 0.913231254 4.044094851 4.53109501 5.51E-05 0.005374531 1.36872237 stable

RNF34 0.268663264 10.39930179 4.529800904 5.53E-05 0.005374531 1.364842792 stable

FEM1C 0.665392687 7.744890149 4.523721191 5.63E-05 0.005444311 1.346620946 stable

RNF4 0.208293245 11.7197739 4.522954317 5.65E-05 0.005444311 1.344323025 stable

GYLTL1B -0.415659554 7.382520385 -4.521279042 5.68E-05 0.00545019 1.339303509 stable

HSPE1-MOB4 /// MOB4 0.185537684 10.91447907 4.519841858 5.70E-05 0.005452158 1.334997812 stable

NCOA2 0.287059713 8.255545446 4.501410476 6.04E-05 0.005747662 1.279815324 stable

SRPK2 0.232838911 10.58047655 4.497862654 6.10E-05 0.005766167 1.269201193 stable

RPL36A -0.479040989 7.621699695 -4.497750892 6.10E-05 0.005766167 1.268866871 stable

LOC101928625 /// MED21 0.568790616 8.291990109 4.492605865 6.20E-05 0.005834875 1.253478977 stable

TRAF6 0.23574075 8.313532729 4.48344761 6.38E-05 0.005977902 1.226101538 stable

ZNF180 0.495140369 6.565199114 4.476610765 6.51E-05 0.006080842 1.205674859 stable

EXOSC3 0.348501777 6.187981228 4.473120168 6.59E-05 0.006122233 1.195249589 stable

ZBTB41 0.283733874 8.724890224 4.468100539 6.69E-05 0.006193058 1.180262022 stable

CIITA -0.434988021 9.236017354 -4.460629229 6.84E-05 0.006312195 1.157963931 stable

EED 0.185859171 10.51462252 4.456696093 6.93E-05 0.006364052 1.146230181 stable

ZNF564 0.328619364 7.786556998 4.452994097 7.01E-05 0.006390263 1.135188955 stable

MSANTD4 0.558801092 6.861821499 4.452828226 7.01E-05 0.006390263 1.134694311 stable

LINC00926 -0.843350673 10.06720423 -4.450700634 7.06E-05 0.006407362 1.128350129 stable

FEZ1 1.198251439 6.498797828 3.379924801 0.001667647 0.005107388 -1.905963764 up

BNC2 1.062550078 3.350146822 4.448691604 7.10E-05 0.006422256 1.122360357 up

EMC3-AS1 -0.304086024 7.100697452 -4.444755394 7.19E-05 0.006450797 1.110627312 stable

MAT2B 0.134195465 13.06804434 4.439319649 7.31E-05 0.006511706 1.094429845 stable

C1orf21 0.481199189 5.733927192 4.4392217 7.31E-05 0.006511706 1.094138034 stable

SBDS /// SBDSP1 -0.405832188 10.49748356 -4.435393856 7.40E-05 0.00656386 1.082735644 stable

LOC100130872 1.026588309 7.092308045 4.445448007 7.17E-05 0.006450797 1.112691614 up

MBD4 -0.216993339 12.03409884 -4.424455222 7.65E-05 0.006737215 1.050168855 stable

UNC50 0.161707591 10.51725826 4.420516406 7.74E-05 0.006793711 1.038448393 stable

GZMM 0.697210053 10.81186733 4.418217395 7.80E-05 0.006816375 1.031608931 stable

LAS1L -0.149998167 9.134787118 -4.413420908 7.91E-05 0.006891805 1.01734326 stable

FCRL1 -0.880038198 9.542659724 -4.409146878 8.02E-05 0.006953824 1.004635679 stable

C2orf88 -0.62214629 10.66225761 -4.408093888 8.04E-05 0.006953824 1.00150553 stable

ARF1 -0.511571898 8.503385446 -4.400487529 8.23E-05 0.007091829 0.978901828 stable

TSEN54 0.416497366 10.05288254 4.396566941 8.33E-05 0.007151367 0.967256019 stable

UTRN 0.281734147 11.13520747 4.387572167 8.56E-05 0.007324461 0.940550506 stable

WTAP -0.853037302 8.930542887 -4.379378048 8.78E-05 0.007477313 0.916237724 stable

TAF5 0.399029741 7.940553175 4.37846115 8.81E-05 0.007477313 0.913518121 stable

C16orf80 0.253578992 10.12970044 4.369664867 9.05E-05 0.007639443 0.887437102 stable

ZNF443 0.493132761 7.721087075 4.36863815 9.07E-05 0.007639443 0.88439401 stable

STARD3NL 0.290978568 10.85978196 4.367932461 9.09E-05 0.007639443 0.882302552 stable

KIR2DL2 1.081825027 8.44118278 4.432677386 7.46E-05 0.006593967 1.074645709 up

HMGA1P4 /// HMGA1P4 -0.214490253 5.529110997 -4.362983581 9.23E-05 0.007700971 0.867638654 stable

LOC101928963 -0.316894232 6.375890516 -4.352814659 9.52E-05 0.007916116 0.837524743 stable

STAM 0.182253416 8.67876229 4.349555669 9.62E-05 0.007967211 0.827878643 stable

GALNT3 0.499430855 8.801309858 4.347786658 9.67E-05 0.0079762 0.82264366 stable

C9orf40 0.62577568 5.348548138 4.346890358 9.70E-05 0.0079762 0.81999154 stable

GZMH 1.144283822 11.08631825 3.190175656 0.002819166 0.007046716 -2.400561032 up

BOK 1.254552485 3.994486324 3.165356812 0.003016562 0.007366883 -2.464047891 up

RAB21 0.128850981 10.90845674 4.333236861 0.000101116 0.008229552 0.779614053 stable

GTF2E1 0.240626515 9.353077714 4.327645699 0.000102856 0.008342355 0.763091701 stable

ZNF805 0.377015983 4.518754474 4.321175629 0.000104907 0.008479463 0.743981147 stable

FBXL3 0.217162528 11.23595527 4.314393453 0.0001071 0.008627066 0.723959198 stable

MAML1 0.141308657 10.35592697 4.310593513 0.000108348 0.008681271 0.712745936 stable

RBM14-RBM4 /// RBM4 0.152075333 11.78523456 4.310100838 0.000108511 0.008681271 0.711292347 stable

CD160 0.964918151 9.345217885 4.304083515 0.00011052 0.008766684 0.693543438 stable

MSN 0.1195952 14.39371719 4.303559536 0.000110697 0.008766684 0.691998294 stable

TES 0.229856298 11.30212679 4.303558402 0.000110697 0.008766684 0.691994948 stable

ZNF780A /// ZNF780B 0.367259832 7.515076962 4.291367829 0.000114885 0.009067854 0.656064944 stable

ZNF830 0.371066378 9.987899652 4.28894986 0.000115734 0.009071467 0.648942515 stable

PAM 0.498818211 9.927314399 4.288144419 0.000116019 0.009071467 0.646570299 stable

ZNF570 0.546164712 3.669512274 4.287947421 0.000116088 0.009071467 0.645990117 stable

NRF1 0.202303975 8.761029664 4.28520945 0.00011706 0.009117108 0.637927421 stable

SMNDC1 0.203065992 11.00490525 4.283336221 0.000117729 0.009138981 0.632412228 stable

SNORA72 -0.559427687 7.592550847 -4.28065843 0.000118693 0.009183459 0.624529689 stable

MIER3 0.362243128 3.407205752 4.276916729 0.000120052 0.009258161 0.61351823 stable

TRIM32 0.526237219 7.617201236 4.268319765 0.000123233 0.009472391 0.588230997 stable

LYPLAL1 -0.213963652 10.75793436 -4.263770519 0.000124949 0.009569866 0.574857019 stable

TRAPPC6B 0.154774437 8.955696265 4.262809628 0.000125314 0.009569866 0.572032814 stable

LBR 0.176753744 12.73301087 4.26158185 0.000125783 0.009574562 0.568424513 stable

NUP153 0.242726883 10.97643698 4.258448846 0.000126986 0.009634984 0.55921863 stable

KIAA1407 -0.394041535 6.950707325 -4.254659012 0.000128457 0.009715238 0.548085953 stable

KIF21A 0.753209062 7.474336636 4.248564963 0.000130857 0.009865031 0.530191992 stable

NDUFV2-AS1 -0.444277408 6.003181818 -4.24275774 0.000133185 0.010008445 0.513148736 stable

ZNF200 0.436276882 9.006554977 4.237389375 0.000135373 0.010121724 0.497400864 stable

UBQLN1 0.348324272 8.525398332 4.236951173 0.000135553 0.010121724 0.496115734 stable

CD200 -1.026101521 5.726553189 -4.36326059 9.23E-05 0.007700971 0.868459307 down

LOC101928954 -0.180253916 6.355474083 -4.231730039 0.000137718 0.010218465 0.48080718 stable

USP38 0.280850853 9.779044367 4.22909795 0.000138821 0.010267984 0.473092371 stable

LLGL2 0.276719664 6.086832015 4.219849257 0.00014277 0.010502155 0.44599763 stable

RUNX3 0.415537073 11.66855306 4.219593187 0.00014288 0.010502155 0.445247757 stable

IZUMO4 -0.219033454 6.530357092 -4.215045996 0.000144863 0.010614717 0.431934579 stable

LRRC8A 0.268983965 10.12963699 4.212226222 0.000146106 0.010672547 0.423681516 stable

TRIM11 0.251219024 9.402297588 4.210958773 0.000146668 0.010680436 0.419972535 stable

BD495725 0.439148228 7.54420587 4.19932145 0.000151929 0.011006304 0.38593683 stable

ZNF780A 0.490186944 7.922281499 4.198995221 0.000152079 0.011006304 0.384983203 stable

YPEL1 0.663486811 8.56036995 4.197130965 0.000152939 0.011025773 0.379534167 stable

TP53RK 0.452765851 8.704060492 4.195331658 0.000153775 0.011025773 0.374275808 stable

MSL2 0.258197388 10.60278739 4.195228747 0.000153822 0.011025773 0.373975084 stable

ZXDA 0.504062673 5.396117282 4.19436934 0.000154223 0.011025773 0.371463838 stable

SPIN4 0.433093593 8.148603856 4.189337932 0.000156589 0.01116097 0.356765512 stable

BATF 0.289871692 8.737891428 4.18554001 0.000158398 0.011248982 0.34567489 stable

BRD2 0.189709266 10.9593309 4.184743015 0.00015878 0.011248982 0.34334799 stable

CD37 -0.217072764 13.43090632 -4.18041155 0.000160873 0.011306708 0.330704748 stable

DLG5 1.176274404 6.447534305 4.340940112 9.88E-05 0.008094225 0.802389617 up

AP1G1 0.227748258 9.679788676 4.18007482 0.000161037 0.011306708 0.329722058 stable

SEPSECS 0.298771577 6.584236663 4.172973442 0.00016453 0.011517615 0.30900475 stable

MINPP1 0.41973867 8.529697499 4.166677417 0.00016769 0.011650044 0.290647901 stable

ZNF580 -0.232494707 10.25324678 -4.166275349 0.000167893 0.011650044 0.289475972 stable

GTF3C1 0.314562662 9.330668966 4.166246281 0.000167908 0.011650044 0.289391247 stable

REM2 -0.439120855 5.9173927 -4.163759211 0.000169174 0.011703347 0.282143012 stable

CASP8AP2 0.238750604 4.068026209 4.161792696 0.000170181 0.011738518 0.276413007 stable

SUCLA2 0.310358213 9.302748528 4.157669968 0.000172312 0.011850767 0.264403549 stable

TMEM2 0.406816331 9.706535054 4.155486515 0.000173452 0.011888098 0.258044981 stable

GSKIP 0.301501609 10.16432758 4.154695703 0.000173866 0.011888098 0.25574232 stable

TM9SF3 0.11573851 11.2646881 4.151369416 0.00017562 0.011973206 0.246058739 stable

SNHG19 -0.331316966 9.053388499 -4.146510936 0.000178213 0.01211485 0.231919843 stable

ZFYVE28 0.540585468 5.831204158 4.141038133 0.000181178 0.012280934 0.216000657 stable

ZBTB8OS -0.167616447 10.486234 -4.13650816 0.000183669 0.012370236 0.202829975 stable

SNAPC4 -0.229439453 7.485081172 -4.136018933 0.00018394 0.012370236 0.2014079 stable

GTF2B 0.285947164 10.46990946 4.135778312 0.000184073 0.012370236 0.200708489 stable

RIPK1 0.227549133 9.041079779 4.131615365 0.000186397 0.01249069 0.188610551 stable

CD300A 0.435039777 12.29574407 4.130125753 0.000187235 0.012511223 0.184282715 stable

PDIK1L 0.380359119 7.888148814 4.128663891 0.000188061 0.012530837 0.180036081 stable

POU6F1 -0.403755325 5.916703413 -4.126349855 0.000189376 0.012582827 0.173315094 stable

MICAL3 -0.526561492 7.548622574 -4.124577348 0.00019039 0.012607571 0.168167921 stable

C6orf141 0.013882423 2.88081638 4.12344708 0.000191039 0.012607571 0.164886182 stable

CNOT4 0.269605224 9.11931731 4.122894873 0.000191357 0.012607571 0.163282972 stable

GAPVD1 0.098557353 11.14146498 4.121881725 0.000191941 0.012610761 0.160341734 stable

DTHD1 0.716332867 3.265917695 4.118368822 0.000193982 0.012709318 0.150145681 stable

CNNM2 -0.26059916 5.26826204 -4.115207094 0.000195836 0.012753701 0.140971744 stable

PFN1 0.108775373 14.60223509 4.115206275 0.000195837 0.012753701 0.140969367 stable

FBXO34 0.204937212 10.17954115 4.114445035 0.000196286 0.012753701 0.13876099 stable

SACM1L 0.325539416 10.68241491 4.111666466 0.000197934 0.012793708 0.130701618 stable

TOX 0.513233414 9.0329224 4.111572722 0.000197989 0.012793708 0.130429747 stable

ZNF555 0.549689234 6.760356271 4.106468113 0.000201053 0.012956053 0.115629172 stable

SMYD5 -0.289517519 7.274019677 -4.105536585 0.000201617 0.012956897 0.112929015 stable

NGRN 0.262901491 10.93246032 4.103428807 0.000202898 0.012996621 0.106820214 stable

GIMAP1-GIMAP5 /// GIMAP5 0.567260506 6.462223458 4.102036187 0.00020375 0.012996621 0.102784763 stable

SFXN3 -0.662768213 8.386071374 -4.101803205 0.000203892 0.012996621 0.102109693 stable

MAP3K14 -0.160853695 8.320433234 -4.099219629 0.000205482 0.013062542 0.094624726 stable

MAPK1 0.364692125 10.12613877 4.098049397 0.000206206 0.013073231 0.091235006 stable

ABI3 0.434874846 10.10606296 4.095948148 0.000207512 0.013120675 0.085149423 stable

TRIP12 0.090001633 12.10540321 4.089420664 0.000211621 0.013301852 0.066252425 stable

NUP98 0.211544414 9.466924096 4.089043306 0.000211861 0.013301852 0.065160336 stable

RARRES3 0.338247998 12.12644875 4.088573193 0.000212161 0.013301852 0.063799865 stable

PPP3CB-AS1 -0.170742056 8.522602297 -4.087814019 0.000212645 0.013301852 0.061603004 stable

STK38L 0.493555973 8.252637555 4.086937724 0.000213205 0.013301852 0.059067419 stable

MFSD6 0.380735206 7.515477416 4.082335263 0.000216172 0.013451251 0.045753545 stable

DDX50 -0.46121095 6.117841912 -4.078897337 0.000218414 0.013535516 0.03581223 stable

ARL5B 0.255214994 8.680263685 4.078496263 0.000218677 0.013535516 0.034652674 stable

DUSP7 0.253249397 9.391323673 4.074217799 0.000221502 0.013674408 0.022285867 stable

SH2D1A 0.510306651 10.85247304 4.070565154 0.000223942 0.013774213 0.011732004 stable

ABCB1 0.570566892 8.482011842 4.070047756 0.00022429 0.013774213 0.010237348 stable

HIPK2 0.32898257 7.886492055 4.06912263 0.000224913 0.01377651 0.007565035 stable

NOL7 -0.142700321 11.48247347 -4.065372349 0.000227456 0.013876184 -0.003265549 stable

TLE4 0.141419162 11.27044257 4.064985661 0.00022772 0.013876184 -0.004382056 stable

RAC2 0.170873848 13.22354492 4.063185895 0.000228952 0.013915198 -0.009578079 stable

TMEM41A 0.26263561 7.984943573 4.059610763 0.000231418 0.014028846 -0.019896985 stable

CD22 -0.746863968 10.11119356 -4.057082382 0.000233178 0.014099191 -0.027192486 stable

CDC34 0.224351435 9.221731364 4.055247429 0.000234464 0.014135078 -0.032486013 stable

PVRIG 0.315790986 11.08300286 4.054521952 0.000234974 0.014135078 -0.034578631 stable

ZFX /// ZFY 0.460935978 7.79581024 4.049359504 0.000238635 0.014318692 -0.049465259 stable

SENP1 0.31329954 7.333614078 4.045224493 0.000241607 0.014444056 -0.061383693 stable

FBXO8 0.424986585 8.861650258 4.044747753 0.000241952 0.014444056 -0.062757499 stable

SNORA76C /// SNORD104 -0.335197399 10.57117867 -4.042913391 0.000243284 0.014486806 -0.068042921 stable

LOC284513 -0.346708652 6.843441946 -4.036420633 0.000248057 0.014733681 -0.086743072 stable

FAM8A1 0.214617632 10.48223224 4.029905457 0.000252937 0.014985716 -0.105495697 stable

SYTL2 0.624506766 7.698477422 4.028842905 0.000253742 0.014995623 -0.108552885 stable

DUSP11 0.239440726 10.24748297 4.02688967 0.000255228 0.01502767 -0.114171914 stable

KIR2DS2 1.467753482 8.036848733 4.333776921 0.000100949 0.008229552 0.781210361 up

CD82 -0.259479025 9.478101847 -4.025039894 0.000256642 0.015031398 -0.119492304 stable

BFAR 0.164455189 9.610027406 4.024320757 0.000257195 0.015031398 -0.121560444 stable

FANCF 0.453928468 9.034917483 4.023868665 0.000257542 0.015031398 -0.122860524 stable

APC 0.23386965 9.680693948 4.0227389 0.000258413 0.015039855 -0.126109128 stable

HIST1H2BJ -0.58101006 5.654503627 -4.022023667 0.000258966 0.015039855 -0.128165568 stable

HCP5 0.496877943 9.555292423 4.019621109 0.000260832 0.015110887 -0.13507233 stable

LINC00494 -1.081405826 4.980227777 -4.232662422 0.000137329 0.010218465 0.483540463 down

PI4K2A 0.300807685 7.950218873 4.016505372 0.00026327 0.015177407 -0.144026822 stable

FADD 0.522013075 8.675202971 4.013936079 0.000265298 0.01525691 -0.151408748 stable

PRKCH 0.387465373 11.38405918 4.012686195 0.00026629 0.015276603 -0.15499914 stable

ANKRD17 0.119432305 10.11845981 4.010740269 0.000267842 0.015328224 -0.160588069 stable

RNF165 0.618349594 3.946633479 4.003342979 0.00027382 0.015589488 -0.181823907 stable

ZNF600 0.494044132 10.45995325 4.002959077 0.000274134 0.015589488 -0.182925562 stable

MED23 0.248272582 10.31032769 4.002639798 0.000274395 0.015589488 -0.183841739 stable

ZNF567 0.388836853 7.425466775 3.998003817 0.000278216 0.015768466 -0.197141434 stable

ANKRD27 0.195965706 10.25747668 3.99686519 0.000279162 0.015784064 -0.200406965 stable

MFAP3 0.661025242 7.985556146 3.989917241 0.000285004 0.016075754 -0.220325113 stable

METRNL 0.607336734 10.46821486 3.988553853 0.000286165 0.016102587 -0.22423196 stable

C1orf228 -0.334971554 9.893018012 -3.980905854 0.000292759 0.016434351 -0.246137468 stable

IL18RAP 0.848629015 10.27513313 3.979462951 0.00029402 0.016449835 -0.250268312 stable

GIMAP8 0.450073449 11.6189911 3.978990476 0.000294434 0.016449835 -0.251620811 stable

RBM8A -0.554251019 7.489165162 -3.975676859 0.000297353 0.016573553 -0.261104464 stable

TIGD1 -0.372667471 8.084421443 -3.973785416 0.000299032 0.016627723 -0.266516356 stable

BANK1 -0.707106758 10.38433773 -3.969264671 0.000303082 0.016776065 -0.279447023 stable

MAN1A2 0.273096752 6.379117206 3.968494883 0.000303777 0.016776065 -0.281648237 stable

ZNF436 0.475263283 6.942532067 3.968425648 0.000303839 0.016776065 -0.281846208 stable

ZSWIM3 0.503114988 6.570167261 3.964813831 0.000307121 0.016917561 -0.292171782 stable

FCER2 -0.841884673 8.166227834 -3.962565999 0.000309181 0.016991237 -0.298595984 stable

HIST1H3A /// HIST1H3B /// HIST1H3C /// HIST1H3D /// HIST1H3E /// HIST1H3F /// HIST1H3G /// HIST1H3H /// HIST1H3I /// HIST1H3J -0.622985532 11.96543713 -3.960681762 0.000310918 0.017031794 -0.303979881 stable

CCDC142 0.369909435 5.796711525 3.959832344 0.000311704 0.017031794 -0.306406603 stable

RNF19A 0.27360057 7.947794701 3.959414858 0.000312091 0.017031794 -0.307599248 stable

PAWR -0.591946923 8.389603562 -3.957632371 0.00031375 0.017082644 -0.312690753 stable

ZNF174 0.352707813 5.687799766 3.956279034 0.000315014 0.017111885 -0.31655579 stable

KIR2DL1 1.13458115 7.535748035 4.180213855 0.000160969 0.011306708 0.330127805 up

CCDC186 0.242170205 7.496626408 3.948236937 0.000322632 0.017445094 -0.339512084 stable

CTBP2 0.555710547 11.0566657 3.939838981 0.000330777 0.017844512 -0.363463289 stable

B4GALT6 0.534388256 3.182333529 3.938592608 0.000332003 0.017869656 -0.367016153 stable

APTX 0.266293364 8.29343444 3.930324037 0.000340248 0.018271606 -0.390574235 stable

DEF8 -0.383904899 8.320362233 -3.927781593 0.000342822 0.018367941 -0.397813738 stable

AKAP13 0.206404906 10.20283791 3.92471971 0.000345948 0.018463976 -0.406529693 stable

PANK4 0.244453236 9.123349409 3.924175127 0.000346507 0.018463976 -0.408079602 stable

KLHL26 0.239783264 7.468281171 3.923725119 0.00034697 0.018463976 -0.409360276 stable

ZFX 0.272317658 8.991056153 3.913924806 0.000357192 0.01896504 -0.437235436 stable

HCST 0.192097868 12.23941906 3.911999774 0.000359234 0.01901815 -0.442707345 stable

AGBL5 -0.442027657 6.717979556 -3.911459203 0.000359809 0.01901815 -0.444243714 stable

OST4 -0.295249977 14.34670821 -3.910388263 0.000360952 0.01902296 -0.447287185 stable

S1PR1 0.41939668 9.918301472 3.909717716 0.000361669 0.01902296 -0.449192612 stable

SLAMF7 0.586024492 9.411498489 3.908376342 0.000363108 0.01902296 -0.453003844 stable

PEX12 0.692395813 6.412071661 3.908351084 0.000363135 0.01902296 -0.453075601 stable

PLAC8 0.206166177 13.17276834 3.904433033 0.000367371 0.019202062 -0.464204683 stable

PPP1R3D 0.74470151 8.000718824 3.902075297 0.000369943 0.019293609 -0.470899446 stable

IL10RA 0.24825384 12.51922403 3.899537901 0.00037273 0.019395965 -0.47810241 stable

TPP2 -0.60574023 4.589375383 -3.891807904 0.000381347 0.019800098 -0.500033333 stable

SNHG11 /// SNORA60 /// SNORA71E -0.266629081 6.646359273 -3.891070161 0.00038218 0.019800098 -0.502125418 stable

GPS2 -0.142920172 10.63631838 -3.889955055 0.000383441 0.0198218 -0.505287301 stable

NAB2 0.404892601 7.568345739 3.885390953 0.000388647 0.020046866 -0.518224742 stable

KIR2DS4 0.966443662 7.252420067 3.882497782 0.000391983 0.020094665 -0.526422351 stable

GADD45A -0.453123948 9.799704749 -3.882341378 0.000392164 0.020094665 -0.526865435 stable

BCL2L2 /// BCL2L2-PABPN1 /// PABPN1 -0.132860086 12.7227652 -3.881843822 0.000392741 0.020094665 -0.528274936 stable

CD244 0.400678399 9.559656558 3.881627812 0.000392991 0.020094665 -0.528886837 stable

DUSP10 0.47658148 8.089916858 3.880879566 0.000393861 0.02009543 -0.531006305 stable

ACRBP -0.482750761 12.6366395 -3.879343426 0.000395651 0.020122256 -0.535356999 stable

PLEKHM2 -0.200571674 8.733676849 -3.8789617 0.000396097 0.020122256 -0.536438016 stable

CA2 -0.560270938 12.48963625 -3.877485764 0.000397827 0.020166572 -0.540617316 stable

PEBP1 -0.201903622 10.76238272 -3.869640187 0.000407145 0.020594556 -0.562821458 stable

CSRNP2 0.302039374 7.680466151 3.864372866 0.00041352 0.020872114 -0.57771777 stable

ZRSR2 -0.15261693 10.30331282 -3.861771676 0.000416704 0.020987766 -0.585070834 stable

SLC9A3R1 0.257593127 11.46621278 3.858594648 0.000420624 0.021139965 -0.59404874 stable

AUTS2 0.723454832 10.31816392 3.857160271 0.000422406 0.021183715 -0.598101061 stable

MRFAP1L1 0.251160044 11.97959066 3.856446043 0.000423296 0.021183715 -0.600118611 stable

CHMP3 /// RNF103 /// RNF103-CHMP3 -0.129033657 10.50742586 -3.853270581 0.000427275 0.021333551 -0.609086672 stable

SPINT2 -0.240552594 10.58629557 -3.85215416 0.000428682 0.021333551 -0.61223887 stable

ATAT1 -0.224003193 4.073673604 -3.851893851 0.000429011 0.021333551 -0.612973791 stable

RP4-635E18.8 -0.412044589 10.0007664 -3.849594715 0.000431926 0.021433183 -0.619463908 stable

HIST1H2BD -0.571541416 10.27737958 -3.84581887 0.000436755 0.021627166 -0.63011886 stable

INO80B /// INO80B-WBP1 /// WBP1 -0.15252381 9.151211068 -3.844780203 0.000438092 0.021647818 -0.633049038 stable

SIGLEC17P 0.978932813 7.367562472 3.840738928 0.000443334 0.021860899 -0.644446537 stable

CDCA7L -0.538732025 10.28954996 -3.839637144 0.000444773 0.021886 -0.647552953 stable

CITED4 -0.354028531 7.328935764 -3.835739097 0.000449903 0.022092189 -0.6585401 stable

CDK17 0.267448491 8.656440077 3.833288964 0.000453156 0.022205589 -0.665443588 stable

COTL1 -0.611630539 7.188072423 -3.831982469 0.0004549 0.022244714 -0.669123964 stable

SMEK2 -0.49768675 5.982280487 -3.830870365 0.00045639 0.022271262 -0.672256307 stable

BC034636 /// CTB-113P19.4 0.020304197 3.013779092 3.826604501 0.000462148 0.022464225 -0.684267761 stable

ASTE1 0.447870086 8.303451878 3.825987821 0.000462987 0.022464225 -0.686003664 stable

ZNF468 0.361673916 9.089691917 3.824648076 0.000464813 0.022464225 -0.689774505 stable

UBAP2L -0.168866869 9.497747066 -3.824510608 0.000465001 0.022464225 -0.69016139 stable

HENMT1 0.365170469 9.243799861 3.824423639 0.00046512 0.022464225 -0.690406148 stable

HIST1H2BH -0.684420575 9.711721972 -3.821973994 0.000468479 0.022580107 -0.697299209 stable

MAFK 0.172529438 5.154713861 3.820777673 0.000470128 0.022613254 -0.700664826 stable

NAA30 0.452897213 8.092877627 3.819841634 0.000471422 0.022629231 -0.703297862 stable

CPNE5 -0.591016222 10.30647413 -3.818887817 0.000472745 0.02264649 -0.705980608 stable

GIMAP2 0.225046117 12.57414544 3.817227412 0.000475055 0.022667596 -0.710650027 stable

C12orf42 -0.288154412 3.522854431 -3.817186213 0.000475113 0.022667596 -0.710765876 stable

PROX2 0.011315528 2.629205543 3.816127486 0.000476592 0.022692139 -0.713742756 stable

HAVCR2 0.613403878 8.021828206 3.81455209 0.000478801 0.022751274 -0.718171699 stable

TOMM40 -0.135801157 8.980555214 -3.812659488 0.000481468 0.022831889 -0.723491329 stable

GIMAP1 0.26308424 9.864122379 3.811058953 0.000483735 0.02289323 -0.727989112 stable

SVIP -0.375465825 9.642963317 -3.80942946 0.000486054 0.022956764 -0.732567402 stable

EPC2 0.196025167 9.445710691 3.801277372 0.000497815 0.023465154 -0.755458647 stable

SMKR1 0.700859024 5.142353295 3.800170631 0.000499433 0.023494331 -0.7585647 stable

SMARCAD1 0.175010853 9.631848004 3.798115926 0.00050245 0.023556622 -0.764330124 stable

ABHD17A 0.284387907 12.42826728 3.797905436 0.00050276 0.023556622 -0.764920672 stable

ZNF319 0.227577266 7.782617372 3.792600097 0.000510637 0.023873577 -0.779800374 stable

HIST1H2BC /// HIST1H2BE /// HIST1H2BF /// HIST1H2BG /// HIST1H2BI -0.663753684 9.173815106 -3.791987135 0.000511555 0.023873577 -0.781518925 stable

ZNF764 0.330287933 6.131832814 3.786287296 0.000520166 0.024226595 -0.797493424 stable

PHAX 0.25759833 7.641762244 3.785622296 0.000521179 0.024226595 -0.79935646 stable

RP11-796E2.4 -0.393044199 7.057912015 -3.781009919 0.000528263 0.02446267 -0.812274217 stable

DCP2 0.151439732 11.52240852 3.780170891 0.000529562 0.02446267 -0.814623291 stable

AMMECR1L 0.273228778 9.148816507 3.779643273 0.00053038 0.02446267 -0.816100371 stable

KCNH8 -0.890566334 3.706496917 -3.779618597 0.000530418 0.02446267 -0.816169448 stable

LOC100506473 /// RP11-332H14.2 -0.376657713 5.584863331 -3.777203321 0.000534179 0.024587926 -0.822929862 stable

SNX5 0.256792504 10.61240422 3.772973317 0.000540829 0.024845381 -0.834765 stable

RARA 1.059365619 7.025030534 2.881212285 0.006430959 0.011835883 -3.16933427 up

PPP1R10 0.318758106 6.886366926 3.768985621 0.000547171 0.025038919 -0.845916645 stable

BHLHE40 0.517433968 8.319316457 3.768054896 0.000548661 0.025054309 -0.848518655 stable

LOC101930306 /// RRN3 /// RRN3P1 /// RRN3P2 -0.557240237 7.60328569 -3.767446681 0.000549637 0.025054309 -0.850218873 stable

NCAM1 0.807666558 4.89270892 3.766433052 0.000551268 0.025068154 -0.853052111 stable

ABCC1 -0.156099371 10.32865512 -3.765933864 0.000552073 0.025068154 -0.854447287 stable

TIGIT 0.55742592 8.508895805 3.765251208 0.000553175 0.025069811 -0.856355098 stable

LOC286087 1.226090366 5.177232296 2.772986989 0.008501776 0.013819645 -3.426843302 up

HIST1H2BK -0.409043527 12.33021738 -3.761297805 0.000559601 0.025263676 -0.867400514 stable

MRTO4 -0.225213715 8.338336786 -3.759904521 0.000561883 0.025318095 -0.871291943 stable

ABCB4 -0.535862273 2.787476749 -3.758690631 0.000563878 0.025359423 -0.874681789 stable

SF3A2 -0.18646582 9.466565827 -3.757317977 0.000566143 0.025412678 -0.878514385 stable

NOTCH1 0.151873451 9.736919288 3.750396208 0.000577697 0.025881915 -0.897830897 stable

TTC32 -0.281854829 10.25432427 -3.748536431 0.00058084 0.025973257 -0.903018162 stable

TOMM70A 0.184746174 9.662418924 3.747732451 0.000582204 0.025984844 -0.905260245 stable

HYLS1 0.625424096 8.143958065 3.746676162 0.000584 0.026015662 -0.908205615 stable

RASGEF1A 0.655945092 7.591741782 3.741941831 0.000592119 0.026327451 -0.921402166 stable

ZNF276 0.300864419 9.255359001 3.741105736 0.000593564 0.026341906 -0.923731909 stable

DTX4 -0.430335821 8.393950295 -3.740226771 0.000595086 0.02635975 -0.92618085 stable

MON1B 0.217853678 9.181439428 3.737187727 0.000600381 0.026544272 -0.934646067 stable

USP6NL /// USP6NL-IT1 -0.345210737 7.253678859 -3.735232852 0.00060381 0.026569645 -0.940089662 stable

LOC644656 -0.403609642 4.11527382 -3.734952081 0.000604304 0.026569645 -0.940871394 stable

FSD1 0.325930846 3.651903492 3.734929898 0.000604343 0.026569645 -0.940933156 stable

SMARCB1 -0.210750346 9.603785896 -3.732039497 0.000609453 0.026744308 -0.948979116 stable

HSPA13 0.345030075 9.457426366 3.731174045 0.000610991 0.026761876 -0.951387697 stable

EIF5 0.141785538 11.37607828 3.729765807 0.000613502 0.026821903 -0.955306312 stable

ASH1L-AS1 -0.311180277 7.828311301 -3.728348792 0.000616039 0.02688283 -0.95924866 stable

PTGER2 0.355685357 10.76874004 3.723279473 0.000625196 0.027231925 -0.973346545 stable

LOC155060 /// ZNF783 -0.151264798 5.673840133 -3.718725173 0.000633535 0.027544152 -0.98600452 stable

ARHGEF9 0.324962674 7.182693399 3.716370602 0.000637889 0.027682256 -0.99254585 stable

TCEANC 0.439273602 5.247109999 3.715606241 0.000639308 0.027692761 -0.994668936 stable

PPP1CC -0.088526511 12.8809117 -3.713765049 0.00064274 0.027790232 -0.999782181 stable

LYRM9 -0.585908992 6.631791051 -3.710572586 0.000648732 0.027980116 -1.008645283 stable

NANP 0.83363598 6.473895268 3.709827923 0.000650138 0.027980116 -1.010712144 stable

PTPN22 0.226124974 10.53616208 3.709530365 0.0006507 0.027980116 -1.011537982 stable

ZKSCAN4 0.462361687 7.734781514 3.704943231 0.000659431 0.028259442 -1.024265108 stable

ACAP3 -0.229694339 8.818099824 -3.704855305 0.000659599 0.028259442 -1.024508989 stable

CHIC2 0.235680592 10.47256446 3.701269604 0.000666504 0.028440846 -1.034452343 stable

IMPA1 0.275901295 10.07332447 3.700794035 0.000667425 0.028440846 -1.035770781 stable

ACTN4 0.190558482 10.96089948 3.700577439 0.000667845 0.028440846 -1.036371234 stable

CUTC -0.143958973 9.438278543 -3.699526096 0.000669887 0.028440846 -1.039285552 stable

ZNRD1 0.239709891 9.967546657 3.698521498 0.000671843 0.028440846 -1.042069927 stable

CCND2 0.439548782 7.703032008 3.697918954 0.000673019 0.028440846 -1.043739786 stable

SEC22A 0.184287855 7.171817166 3.697846176 0.000673161 0.028440846 -1.043941472 stable

FLJ12120 0.944329642 5.423694264 3.697401637 0.000674031 0.028440846 -1.045173348 stable

APOBEC3G 0.341815388 12.16603871 3.696422857 0.000675948 0.028440846 -1.047885437 stable

C1orf174 0.220093697 10.12024681 3.69630418 0.000676181 0.028440846 -1.048214253 stable

BLK -0.938193463 8.345884393 -3.695818825 0.000677134 0.028440846 -1.049558971 stable

SPRED3 -0.18550437 8.198836299 -3.694663897 0.000679407 0.02848546 -1.052758462 stable

XCL1 /// XCL2 0.930152858 9.680039607 3.690918995 0.000686829 0.028745401 -1.063129692 stable

COG8 /// PDF -0.153796513 9.054019214 -3.687552795 0.000693568 0.028975851 -1.072447887 stable

LCMT2 0.531052192 7.256436929 3.68556855 0.000697569 0.029027808 -1.077938715 stable

RBM15 0.156362844 9.482954793 3.685289893 0.000698133 0.029027808 -1.078709707 stable

TBC1D22B 0.359424691 4.82953927 3.684576233 0.000699579 0.029027808 -1.080684142 stable

GCC1 0.32411098 8.545444489 3.684493017 0.000699748 0.029027808 -1.080914358 stable

KLHL21 0.207582501 7.700398959 3.682918609 0.000702948 0.029078142 -1.085269479 stable

RPL17 /// RPL17-C18orf32 /// SNORD58A /// SNORD58B /// SNORD58C -0.629709225 8.708228065 -3.682680528 0.000703434 0.029078142 -1.08592798 stable

HIST1H2BE -0.500748704 9.750881443 -3.67981992 0.000709289 0.029268755 -1.093838479 stable

MXI1 -0.183675318 10.92302899 -3.67637939 0.000716394 0.029510165 -1.10334877 stable

ZFYVE20 -0.413804775 6.499897906 -3.674914688 0.00071944 0.029583807 -1.107396205 stable

G2E3 0.160125835 8.045594382 3.674278031 0.000720767 0.029586676 -1.10915525 stable

TDG 0.169886691 10.72486716 3.671891412 0.000725765 0.029739938 -1.115748042 stable

HVCN1 -0.281437752 11.10157663 -3.666596142 0.000736974 0.030042568 -1.130368381 stable

LOC100505564 -0.619053228 5.647850163 -3.666572259 0.000737025 0.030042568 -1.130434298 stable

SSBP1 -0.572429598 6.27896695 -3.665914061 0.00073843 0.030042568 -1.132250886 stable

GATA3 0.441730352 8.239136776 3.665885056 0.000738492 0.030042568 -1.132330933 stable

SLC15A4 0.296284796 10.5475483 3.665396444 0.000739537 0.030042568 -1.133679366 stable

MRFAP1 0.109891187 13.79456924 3.664594276 0.000741255 0.030060453 -1.135892941 stable

RLIM 0.292990399 7.896096511 3.663395874 0.000743829 0.030112926 -1.139199485 stable

JAKMIP2 0.618392931 6.505054062 3.661958142 0.000746929 0.030186455 -1.143165689 stable

IVNS1ABP 0.284527908 10.66035945 3.661151771 0.000748673 0.030205036 -1.14538986 stable

SYT11 0.460385969 7.593283838 3.660343317 0.000750425 0.030223893 -1.147619541 stable

VPS37A 0.256883327 9.048510297 3.659035757 0.000753268 0.030260923 -1.151225234 stable

MIR6516 /// SCARNA16 /// SNHG20 -0.386755037 5.520571962 -3.658737474 0.000753918 0.030260923 -1.152047684 stable

B4GALT4 0.335517907 7.650339321 3.655891978 0.000760145 0.03043101 -1.159891906 stable

H2BFS -0.513049729 11.20549271 -3.65561993 0.000760743 0.03043101 -1.16064171 stable

ZNF526 0.45195624 6.61787435 3.654566279 0.000763063 0.030458723 -1.163545479 stable

THAP2 0.320728575 3.160177594 3.654130047 0.000764026 0.030458723 -1.164747578 stable

CLIC4 -0.362546986 8.848540947 -3.651972977 0.000768803 0.030597303 -1.170690669 stable

SPRTN 0.370781049 5.883196038 3.65090751 0.000771173 0.030639789 -1.173625587 stable

CUL3 0.265542742 9.027744376 3.64799318 0.000777692 0.030846695 -1.181651247 stable

ZNF260 0.301263029 8.305624134 3.644165802 0.000786334 0.031136976 -1.192186618 stable

MKLN1 0.148332575 8.415275669 3.640185668 0.000795419 0.031443796 -1.203136798 stable

CRNKL1 0.531717905 8.672245187 3.63313613 0.00081176 0.032035916 -1.222517328 stable

C22orf46 -0.162252278 6.069955108 -3.630037548 0.000819044 0.032269246 -1.231030155 stable

DEXI 0.242701468 8.520542766 3.626223384 0.000828097 0.032557722 -1.241504073 stable

TERF2IP 0.151836747 11.41370162 3.625231518 0.000830467 0.032557722 -1.244226917 stable

COX20 -0.311802672 9.091417443 -3.625209981 0.000830519 0.032557722 -1.244286036 stable

NDEL1 0.183032632 10.69910623 3.623395349 0.000834872 0.032649952 -1.24926656 stable

IRF1 0.299161008 11.67485275 3.623072881 0.000835648 0.032649952 -1.250151494 stable

KIR2DL5A 1.167178313 7.575160238 4.026451834 0.000255562 0.01502767 -0.115431321 up

EPHB6 -0.304895092 8.591178652 -3.620907299 0.000840876 0.032688363 -1.256093403 stable

C11orf80 -0.243642223 6.214255763 -3.620826887 0.000841071 0.032688363 -1.256314004 stable

ADAM19 -0.383643193 9.558694402 -3.620365226 0.00084219 0.032688363 -1.257580469 stable

KIR3DS1 1.924340796 6.738243937 4.017696878 0.000262335 0.01516065 -0.140602819 up

ZNF823 0.402993205 5.944467323 3.617700256 0.000848677 0.032831795 -1.264889681 stable

WDR61 -0.497537194 7.192912166 -3.616134041 0.000852512 0.032925998 -1.269184111 stable

CD19 -0.799647746 9.727732867 -3.614326977 0.000856957 0.033043431 -1.274137799 stable

ELF1 0.142954927 11.79849209 3.613483974 0.000859039 0.033048482 -1.2764483 stable

STRIP1 0.137351004 8.856494983 3.61313636 0.000859898 0.033048482 -1.277400964 stable

LOC101926967 -0.369378252 7.012761082 -3.612026525 0.000862649 0.0331001 -1.280442253 stable

PDZD4 0.562596617 7.615305865 3.608416987 0.000871652 0.033391101 -1.290330328 stable

ZNF230 0.218274505 3.283061815 3.602489828 0.000886634 0.033909775 -1.306556832 stable

TRAPPC1 -0.21031911 12.16158329 -3.601364306 0.000889506 0.033964417 -1.30963664 stable

YRDC -0.280906058 8.802434607 -3.599510563 0.000894257 0.034090481 -1.314708072 stable

TARBP1 -0.25520331 9.638165212 -3.598774346 0.000896151 0.034107389 -1.316721843 stable

MRC2 -0.143165355 8.140623893 -3.59616659 0.000902889 0.034308338 -1.323853191 stable

MSH6 0.16126788 9.635803648 3.595498188 0.000904624 0.034318822 -1.325680638 stable

FAM179B 0.313067355 8.259072738 3.592579282 0.000912239 0.034551959 -1.33365912 stable

BC022568 /// CTC-265F19.1 -0.742424209 6.029543861 -3.590155121 0.000918609 0.034737314 -1.340282843 stable

SEMA4B -0.380846156 9.471226903 -3.588848202 0.000922062 0.034761613 -1.34385292 stable

ZNF581 -0.197281082 9.563199596 -3.588792992 0.000922208 0.034761613 -1.344003722 stable

FANCM 0.240803672 3.306687071 3.588150068 0.000923911 0.03477009 -1.345759733 stable

EIF4A1 /// SNORA48 /// SNORA67 /// SNORD10 -0.571134555 10.73227625 -3.586944964 0.000927111 0.034834802 -1.349050799 stable

CTSW 0.623692678 11.83345003 3.585489105 0.000930992 0.034924825 -1.353025931 stable

ARL6 0.324705798 3.445144676 3.584803232 0.000932826 0.03493789 -1.354898385 stable

APOBEC3C 0.19499266 10.87078646 3.581942274 0.000940512 0.035119729 -1.362706981 stable

MED6 -0.585920166 8.762297235 -3.581884828 0.000940667 0.035119729 -1.362863741 stable

HSPH1 0.238809238 10.41479775 3.576959125 0.000954046 0.035562768 -1.376300414 stable

BCDIN3D 0.550975794 6.450999962 3.575285667 0.000958632 0.035677201 -1.380863297 stable

IGFBP7 0.71526716 9.836154437 3.574003982 0.00096216 0.035751906 -1.384357245 stable

KCMF1 -0.101464264 11.46759619 -3.573447442 0.000963695 0.035752482 -1.385874211 stable

XRN1 0.294824396 8.011512772 3.566331119 0.000983538 0.03643119 -1.405260854 stable

CNEP1R1 0.215375336 10.15141882 3.565383772 0.000986209 0.036472691 -1.407840208 stable

IL12RB2 0.886344374 6.708345094 3.559389744 0.001003272 0.036990963 -1.424152284 stable

RP11-140I16.3 0.313770316 5.425576144 3.559356043 0.001003369 0.036990963 -1.424243958 stable

TRDV3 0.654330848 12.11543937 3.558260357 0.001006518 0.037049015 -1.427224244 stable

DCAF17 0.220898324 7.601302716 3.556890545 0.00101047 0.03709656 -1.430949511 stable

CDC5L 0.170101703 10.07132061 3.556719361 0.001010964 0.03709656 -1.431415005 stable

FKBP3 -0.134088643 10.90199919 -3.556083483 0.001012805 0.037106194 -1.433144018 stable

CASD1 0.221118369 9.715284816 3.555524274 0.001014425 0.037107779 -1.434664433 stable

SLA 0.266775349 11.64325904 3.554298208 0.001017988 0.037180274 -1.437997523 stable

IL1A -0.436350742 7.812903141 -3.552953809 0.001021908 0.037251981 -1.441651641 stable

KIAA0125 -0.909643783 7.461346123 -3.552539636 0.001023119 0.037251981 -1.442777235 stable

TBC1D20 0.143760852 9.727611097 3.551324695 0.001026678 0.037323798 -1.446078692 stable

ELF2 0.169745193 9.750153905 3.549655819 0.001031587 0.037444367 -1.450612735 stable

CHMP3 /// RNF103-CHMP3 -0.155814273 10.38571448 -3.548134125 0.001036082 0.037549588 -1.454745976 stable

RUNDC1 0.323678557 9.201194161 3.547342862 0.001038427 0.037576673 -1.456894861 stable

HIST1H2AC -0.44839662 13.24110208 -3.546801648 0.001040034 0.037577007 -1.458364532 stable

FNIP1 -0.306329134 3.484048511 -3.544851674 0.001045843 0.037728941 -1.463658766 stable

HOOK2 -0.09662359 6.899642401 -3.538579344 0.001064741 0.038351874 -1.48067835 stable

CDK14 -0.371551848 8.958068158 -3.537470649 0.001068115 0.038414591 -1.483685144 stable

TECPR1 -0.193413777 10.53639853 -3.532919797 0.001082074 0.0388572 -1.496022106 stable

GABBR1 -0.452922106 9.682493117 -3.531838498 0.001085416 0.038917806 -1.498952232 stable

ASAP1 -0.24134529 10.55368338 -3.529166262 0.001093719 0.039155806 -1.506191555 stable

C8orf76 0.277924126 7.029420169 3.526903764 0.001100796 0.039349281 -1.51231869 stable

TCL1A -0.721735318 10.95528371 -3.525207839 0.00110613 0.039479941 -1.516910164 stable

QSOX2 -0.306460626 9.247323012 -3.521491127 0.001117906 0.039803825 -1.526968698 stable

UBR1 0.23382696 10.29187671 3.52118171 0.001118892 0.039803825 -1.527805832 stable

CD97 0.203914564 11.88776708 3.520746041 0.001120281 0.039803825 -1.528984474 stable

TMEM68 0.384221841 6.553083527 3.518905892 0.001126168 0.039952633 -1.533961934 stable

ITGAL 0.238261433 13.11024723 3.515344381 0.001137646 0.040299053 -1.543591772 stable

PTP4A1 -0.223229174 9.854744073 -3.513329119 0.00114419 0.040421993 -1.54903856 stable

STRAP -0.577381574 2.906174696 -3.51321759 0.001144554 0.040421993 -1.549339951 stable

CRIP3 -0.3773295 5.002149737 -3.511147218 0.001151317 0.040599884 -1.554933935 stable

NUP35 0.216005777 9.199166543 3.507148799 0.001164487 0.041002838 -1.565732578 stable

GPR18 0.617848911 10.07522202 3.506436261 0.001166849 0.041024596 -1.567656289 stable

FCF1 -0.258206503 8.423511202 -3.505274172 0.001170711 0.041098954 -1.570793268 stable

HCFC2 0.178577183 8.262173087 3.504429453 0.001173526 0.041136385 -1.573073192 stable

RNF111 0.10622806 10.19544891 3.503098219 0.001177976 0.041230918 -1.576665666 stable

LOC100287497 -0.519637549 7.741919243 -3.501320951 0.001183942 0.04133383 -1.581460721 stable

SLC50A1 -0.238909019 9.045572026 -3.501009312 0.001184991 0.04133383 -1.582301389 stable

SNX3 -0.126758242 12.70138743 -3.500653994 0.001186188 0.04133383 -1.583259841 stable

TOMM20 -0.134911657 11.86969814 -3.496976358 0.001198648 0.041671026 -1.593177127 stable

PPP1R18 0.178868983 12.67068042 3.496753155 0.001199408 0.041671026 -1.593778855 stable

ARFGEF1 0.109477564 9.441734321 3.492695509 0.00121331 0.042091829 -1.604714313 stable

RHOC 0.33815373 9.785167888 3.489182699 0.001225469 0.042344776 -1.614176141 stable

LNX2 0.304912569 7.919178633 3.488865555 0.001226573 0.042344776 -1.615030132 stable

NHLRC3 0.222397779 9.73362673 3.488526548 0.001227754 0.042344776 -1.615942953 stable

CHORDC1 0.199902235 10.42032106 3.488205477 0.001228873 0.042344776 -1.616807434 stable

NT5C3A -0.391403787 12.21807506 -3.487855212 0.001230095 0.042344776 -1.617750475 stable

PDGFC -0.405944726 9.098519491 -3.487480672 0.001231403 0.042344776 -1.618758819 stable

EXO5 0.193811032 5.168403364 3.482419164 0.001249211 0.042763861 -1.632380017 stable

RMDN3 0.280049753 9.02722966 3.481842769 0.001251255 0.042763861 -1.633930526 stable

EFR3A 0.175634923 10.9189863 3.481671226 0.001251864 0.042763861 -1.634391952 stable

NCOA1 0.130703589 10.15492021 3.481661719 0.001251897 0.042763861 -1.634417523 stable

DPY19L4 -0.283340206 6.007156745 -3.481441171 0.001252681 0.042763861 -1.635010749 stable

CISH 0.431414643 9.999725278 3.480186661 0.001257144 0.042839417 -1.638384729 stable

M6PR 0.164234597 11.53281859 3.479796279 0.001258536 0.042839417 -1.639434528 stable

PLEKHA3 0.165980797 8.943583924 3.479143985 0.001260866 0.042856688 -1.64118851 stable

RP1-39G22.7 -0.211080149 7.502883349 -3.477589542 0.001266434 0.042983825 -1.645367634 stable

PIGA -0.210911108 8.590926594 -3.472668048 0.001284218 0.043524634 -1.658592692 stable

BTBD10 0.345532326 8.49726964 3.471643578 0.00128795 0.043588312 -1.661344428 stable

EXOC5 0.110816419 9.894250721 3.470028817 0.001293854 0.04372519 -1.665680835 stable

IGHV5-78 -0.346420727 4.271232005 -3.468629243 0.001298991 0.043835836 -1.669438514 stable

DENND2D 0.339455819 12.03398327 3.46640374 0.001307201 0.044049691 -1.675412082 stable

LGALS12 -0.395823904 7.981570624 -3.465589775 0.001310217 0.044088132 -1.677596383 stable

CAPS -0.251508345 9.213853489 -3.464322506 0.001314924 0.044183331 -1.680996607 stable

GNG11 -0.444609309 13.22108305 -3.463005786 0.001319833 0.044223744 -1.684528826 stable

VANGL1 0.557454145 5.761402312 3.462991159 0.001319887 0.044223744 -1.68456806 stable

RP11-410L14.2 -0.275533331 7.057753152 -3.461647693 0.001324914 0.044297187 -1.688171299 stable

LAMC1 -0.366471007 6.76380144 -3.461399099 0.001325846 0.044297187 -1.688837958 stable

NCK2 -0.313614109 12.28401503 -3.457610126 0.001340131 0.044710938 -1.698995845 stable

EBLN3 -0.1824115 10.73068384 -3.456864285 0.00134296 0.044724132 -1.700994691 stable

ARCN1 0.138243294 12.15574905 3.45633708 0.001344963 0.044724132 -1.70240746 stable

NRD1 -0.104180015 11.55067557 -3.455958655 0.001346403 0.044724132 -1.703421467 stable

LOC101929177 -0.171206486 7.558034415 -3.455504692 0.001348132 0.044724132 -1.704637806 stable

CNOT6 0.154755728 10.3176373 3.454681277 0.001351274 0.044765218 -1.706843834 stable

TCRDV2 /// TRDC /// TRDC /// YME1L1 0.579600076 11.86167145 3.451848311 0.001362136 0.04496116 -1.714431588 stable

HIST1H1C -0.390522015 8.363920463 -3.451675424 0.001362802 0.04496116 -1.714894539 stable

RASGRP3 -0.590839208 7.828176934 -3.451643985 0.001362923 0.04496116 -1.714978725 stable

LINC-PINT -0.349990883 9.14943029 -3.450906435 0.001365766 0.044991854 -1.716953566 stable

CDC42EP2 0.923564668 7.488960951 3.449752543 0.001370226 0.045075638 -1.72004275 stable

PIP4K2C 0.223947965 9.196910005 3.448699853 0.001374307 0.045122747 -1.722860524 stable

WDR47 0.261789715 9.415423329 3.448139424 0.001376484 0.045122747 -1.724360461 stable

MAP3K7CL -0.468095885 12.78584704 -3.44790048 0.001377413 0.045122747 -1.724999932 stable

VPRBP -0.425128769 6.856025023 -3.444879771 0.001389213 0.04538896 -1.733082087 stable

SRP54 0.145036412 10.18666432 3.444832385 0.001389399 0.04538896 -1.733208842 stable

APOL6 0.306145921 9.454036737 3.442224603 0.001399665 0.045641479 -1.740183132 stable

PUM2 0.145067205 12.20431593 3.441884383 0.00140101 0.045641479 -1.741092814 stable

CLIC3 1.258797277 9.753985487 3.953870194 0.000317277 0.017195108 -0.32343391 up

SP3 0.23212973 11.39212318 3.438119891 0.001415971 0.045943894 -1.751155203 stable

TNFRSF1A 0.39957667 11.21241618 3.438074521 0.001416153 0.045943894 -1.751276439 stable

ANP32A -0.135829424 11.16500815 -3.431956622 0.001440797 0.046679058 -1.767616924 stable

KLHL9 0.241102914 8.538277118 3.431336808 0.001443317 0.046696368 -1.769271547 stable

ADK -0.318292652 10.22464075 -3.430516655 0.001446657 0.046720269 -1.771460744 stable

SMARCA5 0.108969838 10.57552633 3.43001065 0.001448722 0.046720269 -1.77281126 stable

SBK1 0.36362956 9.189587586 3.429694208 0.001450015 0.046720269 -1.773655784 stable

NUP50 0.161035395 11.28167008 3.428680323 0.001454164 0.046734514 -1.776361375 stable

PGRMC1 -0.442008299 11.86492271 -3.428615171 0.001454431 0.046734514 -1.776535219 stable

GOLGA8N 0.317187517 12.62719036 3.425225933 0.001468385 0.047090959 -1.785576365 stable

VEZF1 0.155885817 10.81702943 3.424949751 0.001469528 0.047090959 -1.786312899 stable

LINC01215 -0.387540815 9.971782549 -3.423842777 0.001474117 0.047173737 -1.789264718 stable

ZNF708 0.296133493 8.047284884 3.420619438 0.001487557 0.047430832 -1.797857076 stable

KIR2DS5 1.110463832 8.03647432 3.771440117 0.000543259 0.02490836 -0.83905325 up

ZNF383 0.31095453 6.970886099 3.420465855 0.0014882 0.047430832 -1.798266371 stable

TBXA2R -0.389549887 7.559843501 -3.41912324 0.001493835 0.047546003 -1.801843986 stable

LOC102724483 /// PIGL -0.210103143 6.727416844 -3.416051313 0.001506805 0.047894006 -1.810026832 stable

TSPAN33 -0.490754148 10.8461519 -3.415548403 0.001508939 0.047897097 -1.811366088 stable

STK38 0.225680286 11.3205944 3.413942185 0.001515772 0.047995654 -1.81564276 stable

B3GAT1 0.8748091 5.585728869 3.413230662 0.001518809 0.047995654 -1.817536901 stable

ZNF250 0.306234347 3.378603827 3.413073807 0.001519479 0.047995654 -1.817954434 stable

TFIP11 0.274780976 8.889210253 3.41290385 0.001520206 0.047995654 -1.818406834 stable

APOL1 0.316639069 8.308461852 3.406647238 0.001547183 0.048744355 -1.835052609 stable

MED11 0.218517067 8.902406524 3.406142745 0.001549379 0.048744355 -1.836394108 stable

SSPN 0.009345151 2.702403166 3.405968551 0.001550137 0.048744355 -1.836857282 stable

LOC100505549 -0.20131224 8.556975556 -3.404374858 0.001557095 0.048897757 -1.841094266 stable

ARHGEF2 0.250261279 7.805713895 3.402633033 0.001564733 0.049072101 -1.84572386 stable

LOC100506639 /// ZNF131 -0.381742872 8.288847391 -3.401076301 0.001571589 0.049169333 -1.849860422 stable

FBXO3 0.239033145 9.284805273 3.400980141 0.001572014 0.049169333 -1.850115907 stable

LSM1 -0.126641128 10.84321787 -3.400274635 0.001575132 0.049201421 -1.851990223 stable

KIAA1429 0.136782325 9.687785668 3.399423059 0.001578903 0.049253806 -1.854252326 stable

HSPA1A /// HSPA1B /// HSPA1L 0.462216507 11.48157446 3.397886305 0.00158573 0.049401261 -1.858333751 stable

RP11-111M22.3 -0.313981743 5.672306146 -3.392971094 0.001607756 0.049991322 -1.871381292 stable

ODC1 -0.366796511 12.1645178 -3.392650186 0.001609204 0.049991322 -1.872232797 stable

MLH3 -0.641148839 11.70473229 -3.39224242 0.001611046 0.049991322 -1.87331471 stable

LDOC1L 0.339601094 9.464616992 3.391388329 0.001614911 0.049997732 -1.875580622 stable

TCEAL4 -0.219752021 9.376835094 -3.391257392 0.001615504 0.049997732 -1.875927971 stable

SNHG7 /// SNORA17 /// SNORA43 -0.46846774 7.091013413 -3.389046606 0.001625553 0.050242604 -1.88179166 stable

NPRL2 0.218065581 10.41025138 3.387506597 0.001632587 0.050393815 -1.885875029 stable

AASDHPPT 0.23747895 10.70362989 3.383231063 0.001652271 0.050913424 -1.897206467 stable

STK17B -0.378930094 11.74857381 -3.382902363 0.001653794 0.050913424 -1.8980773 stable

MLYCD 0.402954076 8.338171391 3.382444931 0.001655915 0.050913424 -1.899289113 stable

VCPIP1 0.247826204 7.426279841 3.381962963 0.001658152 0.05091567 -1.900565828 stable

MAP3K4 0.139118153 9.590970481 3.379958962 0.001667488 0.051073876 -1.905873305 stable

KIR2DS3 1.402594039 3.875814711 3.761845464 0.000558707 0.025263676 -0.865870726 up

BIRC2 0.144467482 12.01556041 3.379106223 0.001671476 0.051124546 -1.908131217 stable

PRSS30P 0.744264845 4.358757506 3.378082338 0.001676276 0.051204775 -1.910841893 stable

MOB3A 0.168758463 12.08414307 3.376370441 0.00168433 0.051384091 -1.915373047 stable

TRAF3 -0.175808303 8.178929793 -3.372908752 0.001700731 0.051747949 -1.924531869 stable

LOC102724200 /// TRAPPC10 0.172119217 9.894550902 3.372814326 0.001701181 0.051747949 -1.924781628 stable

RP11-196G18.24 -0.436195336 6.363660351 -3.372462349 0.001702858 0.051747949 -1.925712578 stable

CTPS1 0.328750045 7.487314818 3.371512495 0.00170739 0.051818732 -1.928224604 stable

LOC100270804 -0.552778155 5.940241704 -3.369654557 0.001716289 0.052011752 -1.93313708 stable

ZNF615 0.431404819 7.209362376 3.369262421 0.001718172 0.052011752 -1.934173717 stable

TRAK1 -0.261991775 7.697300359 -3.366483584 0.001731578 0.052350201 -1.941517896 stable

LOC389765 0.202504976 7.699899658 3.365829575 0.001734748 0.052378704 -1.943245896 stable

AUP1 -0.100419248 11.23840188 -3.36240793 0.001751422 0.05279684 -1.952283483 stable

CAPRIN1 0.151016847 12.01067338 3.362068139 0.001753086 0.05279684 -1.953180702 stable

HEXIM1 0.205136518 10.09396547 3.358692188 0.001769702 0.053229098 -1.962092217 stable

UQCRH /// UQCRHL -0.098598267 13.19977196 -3.355006053 0.001788016 0.053701093 -1.971816968 stable

ZNF20 /// ZNF625-ZNF20 0.523081853 6.176150074 3.354199225 0.001792049 0.053701093 -1.973944763 stable

LRCH4 -0.166289315 7.938648373 -3.353838453 0.001793855 0.053701093 -1.974896113 stable

CMTR2 0.28172547 9.480337531 3.353448403 0.001795809 0.053701093 -1.975924608 stable

GNG2 0.486373196 9.373360549 3.353248942 0.00179681 0.053701093 -1.976450526 stable

POU2F2 -0.295485273 7.136878684 -3.352089593 0.001802634 0.053806804 -1.979507044 stable

OXA1L -0.115160781 12.14120114 -3.350237296 0.001811978 0.053903827 -1.984389261 stable

HIST1H2AD /// HIST1H3D -0.32474772 3.426206321 -3.349497901 0.00181572 0.053903827 -1.98633772 stable

FCGR3A /// FCGR3B 0.81756317 12.65107403 3.349338542 0.001816528 0.053903827 -1.986757632 stable

FAM209B -0.270691158 6.310791203 -3.348902715 0.001818738 0.053903827 -1.987905989 stable

CHPT1 -0.737649126 8.17387858 -3.348517057 0.001820697 0.053903827 -1.988922085 stable

KEAP1 0.342622286 9.43811194 3.34830436 0.001821777 0.053903827 -1.989482454 stable

ZNF566 0.206657404 5.287554849 3.347910745 0.001823779 0.053903827 -1.990519412 stable

ZNF740 0.521954726 6.26083989 3.347824376 0.001824219 0.053903827 -1.990746938 stable

NGLY1 -0.373144589 8.309865053 -3.346214533 0.001832429 0.054078504 -1.994987241 stable

SMG8 0.520279733 8.862444568 3.343458085 0.00184657 0.054427529 -2.002245094 stable

CLTC 0.141047271 13.03450621 3.341847064 0.001854882 0.054604118 -2.006485471 stable

ISOC1 0.240431356 9.028795978 3.34124514 0.001857997 0.0546215 -2.008069511 stable

ARHGEF5 -0.236662716 5.791050772 -3.34083605 0.001860117 0.0546215 -2.009145995 stable

CTSC 0.266621849 12.48319167 3.339726077 0.001865881 0.054722427 -2.012066428 stable

RP11-1094M14.11 -0.218977803 8.439360537 -3.338943136 0.001869957 0.054773662 -2.014126092 stable

KIAA1279 0.353170892 8.106380031 3.338033409 0.001874703 0.054789059 -2.016518958 stable

RP11-50B3.4 -0.256400916 8.496759842 -3.337949567 0.001875141 0.054789059 -2.016739471 stable

MATN1-AS1 -0.349446907 7.387587488 -3.336350246 0.001883515 0.054951231 -2.020945256 stable

NKIRAS1 0.365220933 6.007411981 3.335998049 0.001885364 0.054951231 -2.021871293 stable

MAP3K7 0.224239654 7.74821521 3.334290258 0.001894354 0.055144913 -2.026360847 stable

MPP1 -0.29859547 11.63190735 -3.33224934 0.00190515 0.055355583 -2.031724489 stable

VPS26A 0.15323883 11.35288912 3.331260316 0.001910404 0.055355583 -2.034323052 stable

UNG 0.256888967 8.422900319 3.330964404 0.001911978 0.055355583 -2.035100449 stable

MEX3C -0.265236033 4.115886266 -3.330902533 0.001912307 0.055355583 -2.035262987 stable

LYL1 -0.237952885 10.39421959 -3.330705275 0.001913358 0.055355583 -2.035781179 stable

HGS -0.130528003 10.51896941 -3.329725483 0.001918583 0.055438573 -2.038354832 stable

FAM209A /// FAM209B -0.30209373 4.620329885 -3.329148715 0.001921666 0.055459509 -2.039869651 stable

MAGEE1 0.356798878 7.522026698 3.328467771 0.001925311 0.055496618 -2.041657893 stable

RP11-874J12.4 -0.638898788 7.766165634 -3.327957926 0.001928045 0.055507391 -2.042996676 stable

CIRH1A -0.130208467 9.924778856 -3.326460568 0.001936094 0.055581415 -2.046927885 stable

ARPC2 0.100308009 13.38880607 3.326376596 0.001936547 0.055581415 -2.047148317 stable

ACTB /// ACTG1 0.070004473 14.97187873 3.326161694 0.001937705 0.055581415 -2.047712438 stable

MEGF6 -0.451234306 4.587867438 -3.323512388 0.001952038 0.055924335 -2.054665271 stable

CARM1 -0.125836217 9.437096862 -3.322556009 0.001957236 0.056005059 -2.057174441 stable

AGPAT4 0.553888801 6.126217449 3.321393686 0.001963572 0.056105523 -2.060223392 stable

ZNF222 0.340335635 6.903391266 3.320847252 0.001966558 0.056105523 -2.061656568 stable

HEATR9 0.317433844 3.048425966 3.320601246 0.001967903 0.056105523 -2.062301746 stable

SEPHS2 0.238064169 10.61026921 3.319796097 0.001972313 0.056163169 -2.064413151 stable

BPGM 0.407151045 8.016748708 3.316033319 0.001993047 0.056684947 -2.074276835 stable

NOXA1 -0.210394557 7.397964737 -3.314975321 0.001998914 0.056783153 -2.07704914 stable

KIFAP3 0.279911715 9.487153803 3.314009728 0.002004283 0.05680761 -2.079578887 stable

GGA2 -0.190558132 9.400685932 -3.313951845 0.002004605 0.05680761 -2.07973052 stable

GCLM 0.273105984 9.94480624 3.311781831 0.002016723 0.057072709 -2.085414173 stable

RNF14 0.206195606 9.150525979 3.311408895 0.002018813 0.057072709 -2.086390751 stable

SGOL2 0.379944729 5.511320186 3.30894201 0.002032687 0.057395958 -2.092849057 stable

SEL1L3 -0.394327592 10.18054572 -3.307956603 0.002038255 0.057484157 -2.095428108 stable

GRAMD3 0.312189003 8.045037001 3.305992272 0.002049396 0.057729165 -2.100567972 stable

TMEM184B 0.244919682 9.538119379 3.304777799 0.002056314 0.057803093 -2.103744911 stable

LEPROTL1 0.241702926 11.76567585 3.304668808 0.002056936 0.057803093 -2.104029989 stable

PIK3R4 0.157026 9.921118678 3.303154207 0.002065597 0.057977206 -2.107991053 stable

RPE /// RPEL1 0.332688215 7.318689 3.300730154 0.00207953 0.058298724 -2.114328465 stable

PMS2P1 /// PMS2P5 -0.379298262 5.049117973 -3.29859945 0.002091851 0.05857433 -2.119896815 stable

GALNT10 0.21159308 8.239394644 3.295811113 0.002108081 0.058958579 -2.127180789 stable

VPS26B 0.117998213 9.341521474 3.29406957 0.002118278 0.059173414 -2.131728484 stable

BTK -0.236939241 11.0254654 -3.293346348 0.002122526 0.059221759 -2.13361664 stable

FAT4 0.049664419 2.996624834 3.291719814 0.002132111 0.059418697 -2.137862283 stable

PSMC6 0.156848538 11.8975146 3.288831474 0.002149232 0.059824965 -2.145398656 stable

SLFN5 0.43171407 9.048329317 3.286630537 0.002162367 0.060119425 -2.151138948 stable

RSBN1L 0.164472186 9.935139468 3.284259597 0.002176602 0.060234124 -2.157320225 stable

ZNF638 -0.650622206 6.640468815 -3.284124286 0.002177417 0.060234124 -2.157672919 stable

SLC17A9 -0.243138086 7.067850467 -3.284038891 0.002177931 0.060234124 -2.157895501 stable

DUS1L -0.125633159 9.872317375 -3.283841517 0.002179121 0.060234124 -2.158409943 stable

PDGFRB 0.745160331 5.061326575 3.283812333 0.002179297 0.060234124 -2.158486008 stable

GPR183 0.388753684 10.64279229 3.283029748 0.00218402 0.060293829 -2.160525583 stable

LOC157562 0.250216696 7.241606372 3.28184362 0.002191198 0.060351893 -2.163616352 stable

LMBR1 0.248242714 6.886476107 3.281834198 0.002191255 0.060351893 -2.163640902 stable

ARHGAP26 0.248094297 9.328585686 3.281282332 0.002194603 0.060373392 -2.165078719 stable

SMOX -0.468875307 7.958695842 -3.276887431 0.002221434 0.06104014 -2.176524246 stable

NEXN -0.550104151 9.02300968 -3.276245727 0.002225378 0.061077154 -2.178194698 stable

MED20 0.48275619 6.081084145 3.275520489 0.002229843 0.061128375 -2.180082385 stable

YARS2 0.146824082 8.582524575 3.274772626 0.002234457 0.061154541 -2.182028713 stable

H1FX -0.274239449 12.00965124 -3.274523136 0.002235998 0.061154541 -2.182677961 stable

RPF2 -0.182284421 9.321311415 -3.272541916 0.002248272 0.061418809 -2.187832699 stable

ZNRF2 0.20508381 8.2746853 3.272007977 0.00225159 0.061438113 -2.189221599 stable

PARD3B 0.012187276 2.844997236 3.269113719 0.002269661 0.061859434 -2.196748038 stable

HARBI1 0.10188495 2.684913753 3.266861211 0.00228382 0.062173298 -2.202603032 stable

LOC102725022 -0.150248575 8.333325577 -3.264893404 0.002296258 0.062439643 -2.207716137 stable

RIC8B 0.19006066 8.139909409 3.263517876 0.002304991 0.062604729 -2.211289246 stable

MGC70870 0.271344865 6.135091268 3.262585528 0.002310928 0.062636272 -2.213710656 stable

ZNF2 0.495293397 5.992792235 3.262499245 0.002311478 0.062636272 -2.213934723 stable

CGRRF1 -0.166121234 9.043055219 -3.261454451 0.00231815 0.062744782 -2.216647665 stable

SNORD50A /// SNORD50B -0.417593297 8.880307338 -3.260457713 0.002324533 0.062845208 -2.219235364 stable

ZNF24 -0.297745402 7.16145607 -3.257353072 0.002344519 0.063312783 -2.227292679 stable

LOC153577 0.359634238 8.493770489 3.254832017 0.002360869 0.063681188 -2.23383225 stable

CHSY1 0.166368673 10.8461914 3.254050131 0.002365962 0.063717009 -2.235859866 stable

ATP6V1G1 -0.142760336 12.55932891 -3.253796672 0.002367615 0.063717009 -2.236517086 stable

SLC1A7 0.341950878 2.822615279 3.25180109 0.002380669 0.063937175 -2.241690629 stable

LY6G5C -0.498914994 7.246268837 -3.251715179 0.002381232 0.063937175 -2.241913315 stable

MYL5 -0.186560659 6.642509945 -3.2508612 0.002386841 0.063940347 -2.24412667 stable

DNAJA1 0.203948671 11.40707187 3.250278366 0.002390676 0.063940347 -2.24563708 stable

CEP19 0.260279931 4.928975771 3.250223128 0.002391039 0.063940347 -2.245780221 stable

MIR22 /// MIR22HG -0.367368759 9.174467153 -3.24966 0.002394751 0.063940347 -2.247239404 stable

TRPV2 0.190807827 9.988966803 3.249630922 0.002394943 0.063940347 -2.247314748 stable

SIGLEC7 0.453071066 8.83313292 3.243653545 0.002434679 0.064853151 -2.262794462 stable

MGC16142 0.319377453 4.361998025 3.243213745 0.002437628 0.064853151 -2.26393278 stable

NUP54 -0.477339815 3.702306106 -3.242689781 0.002441145 0.064853151 -2.265288825 stable

FOSL2 0.362639638 7.768297791 3.242538259 0.002442163 0.064853151 -2.265680946 stable

ZFAS1 -0.152647826 13.23707785 -3.242158167 0.002444718 0.064853151 -2.266664539 stable

YTHDF3 0.246496437 10.60741816 3.241902316 0.00244644 0.064853151 -2.267326585 stable

CTD-2537I9.5 0.00672627 2.366358229 3.241606237 0.002448433 0.064853151 -2.268092692 stable

PEX11A 0.39870687 6.026325397 3.240903134 0.002453174 0.064905622 -2.269911813 stable

RAB35 0.286917427 7.536965829 3.240083449 0.002458711 0.064979039 -2.272032281 stable

ATG2B 0.344304552 6.060310739 3.236977921 0.002479799 0.065462793 -2.280063302 stable

SNAP23 -0.207539866 12.67349142 -3.236117141 0.002485674 0.065544331 -2.282288538 stable

RING1 -0.126220034 9.147710822 -3.234500559 0.002496744 0.065762508 -2.286466716 stable

RBX1 -0.193698415 12.58017337 -3.233541349 0.002503335 0.065842471 -2.288945306 stable

ADAM8 0.313321649 9.179031072 3.233244356 0.002505379 0.065842471 -2.289712646 stable

KLHL15 0.195798217 7.865615147 3.231821398 0.002515194 0.06602664 -2.29338859 stable

GFI1B -0.480794771 8.238253111 -3.230629226 0.002523445 0.066169394 -2.296467632 stable

GPX4 -0.135930304 12.85288706 -3.229679094 0.002530039 0.066268434 -2.29892109 stable

WNK1 0.101578869 3.404587587 3.22898395 0.002534874 0.066321222 -2.300715848 stable

ZNF608 -0.314871844 5.383454842 -3.227409864 0.002545855 0.066499286 -2.304779091 stable

NRBP1 0.155695933 10.00948441 3.227198295 0.002547335 0.066499286 -2.305325133 stable

SH2B2 -0.265356702 9.858201438 -3.226551401 0.002551863 0.066543646 -2.306994593 stable

C2orf44 0.413719523 5.720316818 3.225890358 0.002556498 0.066551862 -2.308700368 stable

HIST1H2AG /// HIST1H2AH /// HIST1H2AI /// HIST1H2AK /// HIST1H2AL /// HIST1H2AM -0.537121326 8.625487575 -3.2256432 0.002558233 0.066551862 -2.309338091 stable

ERP27 -0.279792432 8.740548043 -3.225296908 0.002560666 0.066551862 -2.310231554 stable

PPIAL4B /// PPIAL4C /// PPIAL4G 0.303568303 4.918452842 3.223009105 0.002576796 0.066897138 -2.316132911 stable

SFMBT2 0.229051908 9.186466328 3.222338122 0.002581544 0.066946529 -2.317863247 stable

INF2 -0.193810317 7.929125811 -3.221027592 0.002590843 0.067113681 -2.321242259 stable

WAPAL 0.118210097 10.45683066 3.218654482 0.002607763 0.067477654 -2.327358976 stable

LOC101927151 0.289312821 4.320878875 3.2179254 0.002612982 0.067538403 -2.329237673 stable

FKBPL 0.335844823 5.333658908 3.217035834 0.002619363 0.067629029 -2.331529577 stable

CAB39L 0.141382674 4.689069252 3.21574318 0.002628663 0.06777788 -2.334859358 stable

DNAJB2 -0.190802656 8.769196851 -3.215317581 0.002631731 0.06777788 -2.335955503 stable

TLR3 0.415145652 3.280938095 3.215034643 0.002633773 0.06777788 -2.336684173 stable

SGSM2 -0.236636246 8.340159463 -3.210749541 0.002664883 0.068503501 -2.347715392 stable

ARMCX3 -0.202252714 9.926885603 -3.209157502 0.002676529 0.068727777 -2.351811655 stable

ERLEC1 0.19375719 10.35487639 3.208100601 0.002684288 0.06878935 -2.35453038 stable

CPOX 0.18086234 9.08424255 3.208034136 0.002684776 0.06878935 -2.354701334 stable

RGS18 -0.28988689 14.13722581 -3.206567931 0.002695577 0.068920579 -2.358472036 stable

PRKRA -0.121523858 10.40483332 -3.205865905 0.002700762 0.068920579 -2.360277115 stable

BC010186 0.407072028 4.518692054 3.205069207 0.002706659 0.068920579 -2.362325343 stable

SYNPO -0.333061935 5.239559472 -3.205055515 0.00270676 0.068920579 -2.362360541 stable

SCN1B -0.584071984 6.830236494 -3.204741907 0.002709085 0.068920579 -2.363166715 stable

NME8 0.625978388 6.47362675 3.204084248 0.002713966 0.068920579 -2.364857168 stable

GPRIN3 0.133787862 4.088251501 3.203805342 0.002716038 0.068920579 -2.365574008 stable

PRPSAP1 0.401783969 5.722830929 3.203761918 0.002716361 0.068920579 -2.365685615 stable

C20orf197 0.306662846 4.283428914 3.203454995 0.002718644 0.068920579 -2.366474417 stable

ZMYM3 -0.132106597 7.67713901 -3.202908453 0.002722713 0.068920579 -2.367878944 stable

WHAMM 0.180490636 9.400731763 3.202249839 0.002727624 0.068920579 -2.369571293 stable

ACSL3 0.301906239 8.704722355 3.201967738 0.002729731 0.068920579 -2.370296108 stable

ZNF134 0.298359635 7.884769159 3.201742321 0.002731415 0.068920579 -2.370875255 stable

RBM7 0.258741489 9.400388924 3.20163067 0.002732249 0.068920579 -2.371162103 stable

IFT57 -0.352703129 9.580701343 -3.201416511 0.002733851 0.068920579 -2.371712294 stable

CDYL2 0.247489881 4.005608841 3.198235327 0.002757743 0.069448475 -2.379882489 stable

KIAA0430 0.146925386 8.843873331 3.196377991 0.002771784 0.069718527 -2.384650494 stable

BLNK -0.569718605 10.03397699 -3.196029251 0.002774428 0.069718527 -2.385545573 stable

SH3KBP1 0.094350623 11.35758467 3.195643055 0.002777359 0.069718527 -2.386536723 stable

CTA-29F11.1 -0.420162525 8.02730483 -3.194087868 0.002789191 0.069905636 -2.390527314 stable

SLFN11 0.344373872 9.188435911 3.193882456 0.002790757 0.069905636 -2.391054313 stable

USP28 0.467355368 7.605480779 3.191815714 0.002806563 0.070226767 -2.396355613 stable

CCL3 /// CCL3L1 /// CCL3L3 1.025917925 8.09660849 3.621227759 0.000840101 0.032688363 -1.255214236 up

SESTD1 -0.307596874 9.085924081 -3.185974515 0.002851695 0.071204582 -2.411327844 stable

JMJD8 -0.12183851 8.538039934 -3.185049405 0.002858906 0.071308929 -2.413697637 stable

DDX51 -0.251291652 8.174981001 -3.183693098 0.002869509 0.071468281 -2.417171281 stable

ADO 0.272872337 7.383798767 3.183074451 0.002874357 0.071468281 -2.418755415 stable

TSHZ1 0.236267324 10.17270083 3.183067744 0.00287441 0.071468281 -2.418772586 stable

CD72 -0.569235338 9.396920633 -3.181662319 0.002885454 0.071624813 -2.42237071 stable

RIOK2 0.314377471 9.169103247 3.181491911 0.002886796 0.071624813 -2.42280692 stable

SLAIN2 0.126534926 4.526914725 3.179912071 0.002899264 0.071858364 -2.426850345 stable

CD96 0.540876069 6.684166639 3.178558275 0.002909989 0.07204826 -2.430314308 stable

DAB2 0.306404205 2.567607823 3.177719388 0.002916653 0.072137334 -2.432460342 stable

PROCR 0.334899741 4.322249716 3.176419118 0.002927012 0.07231749 -2.435786029 stable

RP11-173B14.4 -0.190664569 5.280579053 -3.175298203 0.00293597 0.072462694 -2.438652345 stable

DNAJC30 -0.231151366 8.002666084 -3.174346574 0.002943595 0.07249619 -2.441085316 stable

ZRANB1 0.284150267 6.767988625 3.174320894 0.002943801 0.07249619 -2.441150965 stable

CEP290 -0.185807143 8.199746451 -3.17397551 0.002946574 0.07249619 -2.442033876 stable

CD47 0.158446291 12.98151574 3.171765876 0.002964369 0.072857803 -2.447681085 stable

PGBD2 0.284780854 7.9868029 3.171083961 0.002969881 0.07291709 -2.449423408 stable

DLG3 0.294363126 3.695726317 3.170124143 0.002977656 0.07297599 -2.451875415 stable

PCYT1B -0.105545943 2.601016001 -3.170021953 0.002978485 0.07297599 -2.452136448 stable

SESN2 0.312686081 7.768668764 3.167796132 0.002996595 0.073343299 -2.457820884 stable

RAB39B 0.284894819 6.706827405 3.167012584 0.003002995 0.073423538 -2.459821402 stable

KIR2DS1 1.313167793 7.324754804 3.619230705 0.000844946 0.032741297 -1.26069244 up

STAP1 -0.498202143 8.663730161 -3.165024506 0.003019291 0.073668832 -2.464895973 stable

PBX3 -0.141572384 9.186194341 -3.164569857 0.00302303 0.073683616 -2.466056204 stable

PDCL 0.341181973 7.397293432 3.162878747 0.003036975 0.073917117 -2.470370935 stable

TAL1 -0.580439858 7.719399264 -3.162646406 0.003038895 0.073917117 -2.470963628 stable

SYPL1 -0.192309611 11.76023036 -3.160851108 0.003053774 0.074202299 -2.475542515 stable

ND6 -0.51570719 10.51924701 -3.160188969 0.003059279 0.074259351 -2.477230906 stable

PF4 -0.336059067 14.49545863 -3.158769528 0.003071113 0.074469731 -2.480849653 stable

FAM199X 0.198008436 9.927466114 3.156805644 0.003087556 0.074788475 -2.485854842 stable

SWI5 -0.353523064 7.298895177 -3.156441172 0.003090617 0.074788475 -2.48678354 stable

CIRBP -0.193264236 13.14786184 -3.155565073 0.003097986 0.074851355 -2.489015642 stable

RAB38 0.29752075 2.775326195 3.155317977 0.003100067 0.074851355 -2.48964512 stable

PCNX 0.186843731 8.387315395 3.154652606 0.003105679 0.074851355 -2.491340014 stable

CDYL 0.178383355 9.2790692 3.15462113 0.003105945 0.074851355 -2.491420186 stable

DHX15 0.078624432 12.68839069 3.153025823 0.003119439 0.075041846 -2.495483021 stable

TRANK1 0.30228271 10.04592885 3.152932525 0.00312023 0.075041846 -2.495720588 stable

CD7 0.382258548 11.81232053 3.151835801 0.003129541 0.075188905 -2.498512913 stable

SNORA21 -0.722621024 5.823004577 -3.151278282 0.003134285 0.075226031 -2.49993217 stable

RBM12B 0.239421081 7.24804277 3.150014045 0.003145066 0.075407855 -2.50314995 stable

PAQR8 0.293869136 10.20830708 3.149635135 0.003148305 0.075408628 -2.504114218 stable

FCHO1 -0.148292323 8.544262181 -3.146213208 0.003177693 0.076035103 -2.512819404 stable

RGCC 0.345468186 11.03848529 3.143998092 0.003196854 0.076415868 -2.518451564 stable

FAM98A 0.262902255 9.547042265 3.142258472 0.00321198 0.076699468 -2.522873088 stable

ALG2 0.271481872 10.2039495 3.141335625 0.003220031 0.076813741 -2.525218067 stable

HDAC3 -0.104566348 9.844464021 -3.139048726 0.003240065 0.077139156 -2.531027392 stable

ITPRIP 0.272570164 9.29154411 3.139029782 0.003240232 0.077139156 -2.531075504 stable

ZNF518A 0.213373148 8.64085674 3.137807552 0.003250988 0.077288183 -2.534179255 stable

ATP8B4 0.544544907 7.531088678 3.13757213 0.003253063 0.077288183 -2.534777009 stable

ANKRD40 0.233172888 6.887291021 3.137179241 0.00325653 0.077292477 -2.535774521 stable

KLF2 0.19267406 14.19369186 3.136493871 0.003262586 0.077298117 -2.537514445 stable

SRP72 -0.12467226 12.11677435 -3.136408589 0.00326334 0.077298117 -2.537730931 stable

AL832909 /// LINC00984 /// LINC00984 /// LINC00984 -0.27954861 9.677008394 -3.134530082 0.003279997 0.077614508 -2.542498596 stable

CTDSPL2 0.240647113 8.362535723 3.133373065 0.003290297 0.077779975 -2.545434276 stable

TTC17 -0.156306882 9.924840869 -3.132501954 0.003298072 0.077885483 -2.547644107 stable

ARID5A 0.184268515 9.168754379 3.131049023 0.003311078 0.078065505 -2.551329089 stable

BACE2 -0.611165752 5.65317628 -3.130909111 0.003312333 0.078065505 -2.551683886 stable

GOPC 0.199189688 9.4248845 3.129738795 0.003322848 0.078212228 -2.554651278 stable

MORC2 0.231624523 10.16943247 3.129476533 0.003325208 0.078212228 -2.555316164 stable

IGLV4-3 /// IGLV4-3 -0.21231519 5.98408205 -3.127095245 0.003346716 0.07863743 -2.561351702 stable

RPL18A /// SNORA68 -0.452150176 4.618211949 -3.126735959 0.003349973 0.07863743 -2.562262101 stable

JHDM1D-AS1 -0.223942754 7.02709213 -3.125960151 0.003357014 0.078724165 -2.564227719 stable

AF289551 0.306853759 7.000362225 3.123569426 0.003378803 0.079156199 -2.570283136 stable

CASP6 -0.287296703 8.298860765 -3.123071355 0.003383359 0.079184067 -2.571544342 stable

ZGLP1 -0.276997517 6.601107578 -3.120215407 0.003409596 0.079718797 -2.578773827 stable

ZNF449 0.357192875 6.76699774 3.119295254 0.00341809 0.079838035 -2.581102244 stable

TRIP4 0.165646268 9.673496663 3.11884154 0.003422286 0.079856735 -2.582250204 stable

EBF1 -0.780936132 8.245030796 -3.117994933 0.003430128 0.079960397 -2.584391971 stable

IER3 0.678166586 9.872829848 3.1175012 0.003434709 0.079987917 -2.585640872 stable

XBP1 0.260485874 10.46568827 3.116939093 0.003439932 0.080023039 -2.58706258 stable

ZNF831 0.471598147 8.36298142 3.116606918 0.003443022 0.080023039 -2.58790266 stable

KAT2A -0.192598763 9.007349575 -3.114619511 0.003461563 0.080374563 -2.592927764 stable

IP6K2 0.204012311 9.61684346 3.114118899 0.003466249 0.08040398 -2.594193246 stable

ELL3 -0.207273686 6.0598215 -3.113678519 0.003470375 0.080420391 -2.595306371 stable

MS4A1 -0.573296934 12.76989003 -3.111212065 0.003493573 0.080867189 -2.601538958 stable

FAM217B 0.233226131 9.946703026 3.110898567 0.003496532 0.080867189 -2.60233094 stable

ACTN1 -0.234838591 12.5410615 -3.109840292 0.003506538 0.08094076 -2.605004077 stable

ZMIZ2 -0.176950562 8.069497596 -3.109834252 0.003506596 0.08094076 -2.605019333 stable

MAPKAPK5 -0.159413874 9.439586554 -3.10673935 0.003536015 0.081539807 -2.612833746 stable

DRAM2 -0.157584526 9.940069154 -3.105218038 0.003550561 0.081795055 -2.616673253 stable

TUBB 0.180466082 11.5614632 3.104789148 0.003554672 0.081804629 -2.617755488 stable

SRSF5 -0.198177317 13.25051826 -3.104449357 0.003557933 0.081804629 -2.618612833 stable

ZNF185 -0.421209554 10.70677855 -3.102110149 0.003580454 0.082242055 -2.624513494 stable

ARMC1 0.10655824 10.12298771 3.101581563 0.003585562 0.082279028 -2.625846485 stable

AFF3 -0.867391648 4.993598469 -3.098959588 0.003611 0.082782005 -2.632456594 stable

ELMOD2 0.301728849 7.431145563 3.096412908 0.003635871 0.083271008 -2.638873681 stable

POP1 0.345620145 5.53095651 3.094483054 0.003654826 0.083502236 -2.643734393 stable

CTD-2124B8.2 0.184532801 5.042713385 3.094299919 0.003656629 0.083502236 -2.644195559 stable

ZBTB25 -0.321729453 8.987386156 -3.094048747 0.003659104 0.083502236 -2.644828031 stable

R3HDM1 -0.113191703 10.19979993 -3.093635246 0.003663182 0.083502236 -2.645869189 stable

LOC100129447 -0.19021544 3.760388594 -3.093580969 0.003663718 0.083502236 -2.646005849 stable

SH3BGRL -0.176309292 12.41641278 -3.092947042 0.003669979 0.083563962 -2.647601841 stable

SPATA5 0.252038953 5.73228076 3.091116548 0.003688115 0.083876948 -2.652209248 stable

SASH3 0.195756462 12.35273661 3.090840512 0.003690857 0.083876948 -2.652903897 stable

MZF1 -0.473095563 6.053877067 -3.087476572 0.00372443 0.084558211 -2.661366313 stable

TNXA /// TNXB -0.309454805 2.958536044 -3.084291654 0.003756481 0.085203659 -2.669373273 stable

NFS1 0.317388051 6.852713672 3.083278852 0.003766728 0.085353767 -2.671918442 stable

PDP2 0.280748531 6.238316624 3.079126675 0.003809014 0.086228881 -2.682347593 stable

SYNJ1 0.256720085 7.414859573 3.078167609 0.003818844 0.0863683 -2.684755304 stable

IGF2BP2 -0.321898293 8.386989975 -3.077499245 0.003825709 0.086440445 -2.686432951 stable

SAMD9 0.274270513 9.737580577 3.076226202 0.003838817 0.086653376 -2.689627777 stable

KPNA3 0.108036008 10.29830392 3.075716843 0.003844074 0.086688837 -2.690905841 stable

GTF3C6 -0.113368551 12.00341986 -3.073743065 0.003864507 0.087066161 -2.695857165 stable

RAB30 -0.419573523 4.704726887 -3.072761528 0.003874707 0.087212415 -2.698318687 stable

CMKLR1 0.620002625 6.691359363 3.072202229 0.00388053 0.087259982 -2.699721098 stable

RSF1 0.199922969 6.755555816 3.071634756 0.003886447 0.087309559 -2.701143846 stable

CENPC 0.201976986 8.285514249 3.070965661 0.003893434 0.087383068 -2.702821175 stable

RALGPS2 -0.756292757 8.522457117 -3.069617738 0.003907546 0.087616193 -2.706199561 stable

GPR114 0.402472201 4.42150143 3.06746901 0.003930142 0.08803892 -2.711583204 stable

SLCO4C1 0.173016473 2.701418656 3.065523994 0.003950702 0.088415278 -2.716454481 stable

SRRM1 -0.124657715 10.20098956 -3.06361388 0.003970992 0.088784879 -2.721236527 stable

ENKUR -0.719254972 5.624877736 -3.062371323 0.003984243 0.088977672 -2.724346347 stable

BCL7A -0.518082551 8.175245248 -3.062096443 0.00398718 0.088977672 -2.725034203 stable

ZNF684 0.22535662 5.206369545 3.060312185 0.004006295 0.089319493 -2.729498193 stable

RP11-216L13.19 -0.298006827 4.903495978 -3.058784454 0.00402273 0.089582169 -2.733319128 stable

ITGAV 0.216621362 9.060557932 3.058509577 0.004025694 0.089582169 -2.734006488 stable

TRMT1 -0.112553708 9.64995809 -3.058105626 0.004030054 0.089594413 -2.735016544 stable

RBM4 -0.282448394 9.19900901 -3.056307159 0.004049516 0.089942087 -2.739512519 stable

HIST1H2AE -0.683349142 8.590962644 -3.05503692 0.004063316 0.090163445 -2.742687012 stable

KPNA1 0.092449588 9.815283227 3.052669242 0.004089156 0.090651314 -2.74860201 stable

LRRC74 -0.103656929 3.118662093 -3.051157623 0.004105735 0.090933133 -2.752376923 stable

SLC35F2 -0.375947041 5.456811705 -3.050652358 0.00411129 0.090970516 -2.753638448 stable

EZH1 -0.155644711 9.714865147 -3.048277347 0.004137499 0.091406612 -2.759566582 stable

MTX3 -0.265226029 8.641761804 -3.048162439 0.004138771 0.091406612 -2.759853325 stable

AKT3 0.268769303 9.152700121 3.046925848 0.004152484 0.091623426 -2.762938729 stable

SIPA1L3 -0.205059321 8.926969736 -3.046397011 0.004158361 0.091667114 -2.764257991 stable

PTCRA -0.375630866 8.3907059 -3.045895377 0.004163943 0.091704223 -2.765509262 stable

TPRKB -0.118923693 9.588750164 -3.044931022 0.004174695 0.091854995 -2.767914389 stable

WDR5B 0.563101206 6.852029345 3.044040683 0.004184644 0.091987857 -2.770134506 stable

ZNF74 0.278348802 6.312591407 3.043522078 0.004190449 0.091993773 -2.771427496 stable

MAP1LC3B -0.138160462 11.47641834 -3.043318069 0.004192735 0.091993773 -2.771936097 stable

RNF44 0.118924203 12.60720424 3.041671612 0.004211226 0.092253576 -2.776040003 stable

EIF2S2 0.157555136 7.279634194 3.041565526 0.00421242 0.092253576 -2.776304383 stable

SNX2 -0.213022208 12.52265582 -3.040344227 0.00422619 0.092469037 -2.779347617 stable

ZMYM6 /// ZMYM6NB 0.178359555 10.01018551 3.039729072 0.004233142 0.092471754 -2.780880177 stable

BAHD1 0.230813816 9.059246842 3.039539974 0.004235281 0.092471754 -2.781351246 stable

GAB3 0.243338565 9.553040248 3.039290125 0.004238109 0.092471754 -2.781973626 stable

AGPAT1 -0.233602881 10.36540516 -3.038619032 0.004245713 0.09250141 -2.783645182 stable

PPP1CA 0.126982862 12.67343613 3.038361727 0.004248632 0.09250141 -2.78428602 stable

PITPNC1 0.244079636 8.97912098 3.038129705 0.004251266 0.09250141 -2.784863857 stable

YTHDF2 0.127974158 11.37825383 3.036435775 0.004270541 0.092657202 -2.789081676 stable

ZNF606 0.295963476 5.89741801 3.036366635 0.00427133 0.092657202 -2.789253803 stable

AMT -0.208448951 8.513543747 -3.036227746 0.004272914 0.092657202 -2.789599563 stable

PHTF1 0.200626906 3.567314451 3.035820868 0.004277559 0.092657202 -2.790612415 stable

SCFD2 -0.338001698 9.825928146 -3.035771518 0.004278123 0.092657202 -2.790735258 stable

PELP1 -0.157532751 8.944321314 -3.034860308 0.004288543 0.092797439 -2.793003238 stable

STRBP -0.393084657 8.793591716 -3.034182648 0.004296308 0.092856468 -2.794689648 stable

DHTKD1 -0.202020309 7.821033774 -3.033933458 0.004299166 0.092856468 -2.795309718 stable

ZFAND1 -0.191338098 10.18769454 -3.031085137 0.004331969 0.093479132 -2.802395113 stable

CPSF6 -0.137435031 10.85542538 -3.029513747 0.004350167 0.093772603 -2.806302307 stable

TSPAN14 0.285556622 10.75369954 3.029222945 0.004353543 0.093772603 -2.80702524 stable

LAMTOR1 -0.18538007 12.32917613 -3.028012362 0.004367622 0.09398978 -2.810034281 stable

MMP24-AS1 -0.145956269 7.969446706 -3.025315459 0.00439914 0.094581515 -2.816735087 stable

E2F6 0.560439814 6.663644625 3.02487037 0.004404363 0.094607316 -2.817840618 stable

PSMC4 0.162860871 10.56562373 3.021927356 0.00443904 0.095115029 -2.82514808 stable

BMP6 -0.524317354 8.799036398 -3.021882079 0.004439576 0.095115029 -2.825260468 stable

FAM83G -0.105376614 7.158947665 -3.021835221 0.00444013 0.095115029 -2.825376779 stable

OSBPL10 -0.298130667 4.838792669 -3.021002764 0.004449988 0.095239459 -2.827442927 stable

FAM129C -0.661436018 6.111203213 -3.019471561 0.004468174 0.095541743 -2.83124244 stable

LINC00672 0.441411428 6.869869048 3.018795325 0.004476228 0.095554889 -2.832920071 stable

NGFRAP1 -0.409205008 12.36928837 -3.018737772 0.004476914 0.095554889 -2.833062839 stable

CLIP4 0.208198547 8.428303039 3.018198467 0.004483347 0.095605454 -2.834400585 stable

LIPE -0.161851618 7.095790035 -3.017823852 0.004487822 0.095614178 -2.83532973 stable

MTCH1 -0.09716931 12.40693919 -3.016680238 0.004501506 0.095818939 -2.838165758 stable

DPH1 /// OVCA2 -0.126845825 9.807918381 -3.016232167 0.004506879 0.095844278 -2.839276743 stable

LCN10 -0.602675242 7.421098374 -3.015901568 0.004510846 0.095844278 -2.84009639 stable

WDR34 -0.316137696 6.691619303 -3.014614417 0.004526326 0.096086375 -2.843287077 stable

GLTSCR2 -0.170066669 7.983742974 -3.013835096 0.004535722 0.096199023 -2.845218508 stable

DAXX 0.158632701 10.1729278 3.013406299 0.0045409 0.096222077 -2.846281087 stable

PDE12 0.16770785 8.531634462 3.01289289 0.004547107 0.096266876 -2.847553217 stable

RBFA -0.115463604 7.782205836 -3.011622835 0.004562496 0.096505809 -2.850699593 stable

PRDM10 0.184769288 5.780569142 3.010807543 0.0045724 0.096554805 -2.852718932 stable

FRYL 0.147216717 9.886880126 3.010756397 0.004573022 0.096554805 -2.8528456 stable

BUD13 0.255124172 8.747146754 3.009249275 0.004591387 0.096839587 -2.856577555 stable

GNG8 -0.669875976 6.791315314 -3.008974349 0.004594744 0.096839587 -2.857258207 stable

AKIP1 /// NUAK2 0.316597192 10.20722439 3.00792445 0.004607587 0.097023331 -2.859857151 stable

FCRL2 -0.652517461 9.730018237 -3.006998081 0.004618947 0.097175545 -2.862149842 stable

CREM 0.509360614 8.589118682 3.005238084 0.004640603 0.097481169 -2.866504498 stable

GXYLT1 0.115890631 9.224433612 3.005063398 0.004642757 0.097481169 -2.86693663 stable

KB-431C1.4 -0.276325829 7.910247125 -3.004635696 0.004648036 0.097481169 -2.86799459 stable

RGS2 0.400246241 14.39482276 3.004178273 0.004653689 0.097481169 -2.869125967 stable

RPL22L1 -0.172200607 10.94069572 -3.004137224 0.004654196 0.097481169 -2.869227492 stable

USP37 0.116171749 7.503458978 3.002962708 0.004668741 0.097698803 -2.872131993 stable

PHKB -0.144813347 10.92964154 -3.00013613 0.004703919 0.098347447 -2.87911906 stable

TSPAN13 -0.461841002 10.51341697 -2.999596127 0.004710668 0.098355635 -2.880453438 stable

SHQ1 0.130036129 10.10789402 2.999435763 0.004712674 0.098355635 -2.880849678 stable

SOCS4 0.600036362 5.137864031 2.99828201 0.00472713 0.098569871 -2.883700078 stable

ACBD6 -0.099066464 8.792011998 -2.99764279 0.004735157 0.098649793 -2.88527901 stable

HMOX2 0.177826054 10.07201537 2.997053471 0.004742568 0.098716766 -2.886734494 stable

CD164 0.104987338 13.14170233 2.995436098 0.004762965 0.099053678 -2.890728132 stable

UBOX5 -0.285152363 6.914327117 -2.9949504 0.004769107 0.099093782 -2.891927164 stable

AK291611 /// TRGV7 /// TRGV7 0.436711689 5.821254717 2.993317258 0.004789812 0.099436151 -2.895957979 stable

CNTRL -0.142075595 9.82815424 -2.991212461 0.004816621 0.099829603 -2.901150889 stable

ZNF668 0.357427527 6.075478235 2.99083968 0.004821384 0.099829603 -2.902070371 stable

LOC283070 -0.182889866 10.15575915 -2.990830809 0.004821497 0.099829603 -2.902092251 stable

LIM2 0.232432932 2.79416313 2.989428721 0.004839451 0.100113211 -2.905549921 stable

ARHGAP32 -0.350608209 6.115231945 -2.988990449 0.004845076 0.100141499 -2.90663053 stable

PSMG4 -0.204064848 10.23888531 -2.988120667 0.004856257 0.100249519 -2.908774788 stable

RP11-248J18.3 -0.21313466 8.603069347 -2.987921084 0.004858826 0.100249519 -2.909266762 stable

SMC2 0.290314079 7.478074797 2.987156944 0.004868674 0.100336024 -2.911150183 stable

PSMD10 0.12622889 10.18124458 2.986934058 0.00487155 0.100336024 -2.911699487 stable

CCDC127 0.214020639 8.750384672 2.985991162 0.004883735 0.10049898 -2.91402298 stable

LOC389906 -0.288211416 7.989308876 -2.985259704 0.004893207 0.100605878 -2.915825131 stable

IFITM2 0.228530195 13.82425935 2.984551704 0.004902392 0.100659248 -2.917569224 stable

IPO9 -0.231423897 7.02637614 -2.983911421 0.004910712 0.100659248 -2.919146282 stable

CTC-338M12.4 -0.102572168 7.820249638 -2.983547457 0.004915447 0.100659248 -2.920042654 stable

CLCN4 -0.620159842 3.369026034 -2.983147243 0.004920659 0.100659248 -2.921028226 stable

FKBP1B -0.419565394 8.52695906 -2.983108493 0.004921164 0.100659248 -2.921123649 stable

FAM122A 0.30407366 8.382101575 2.98228994 0.004931842 0.100659248 -2.923139151 stable

HIST2H2BE -0.615846976 10.64955663 -2.98188674 0.004937109 0.100659248 -2.924131816 stable

RFWD3 0.18026532 7.6607822 2.981882869 0.00493716 0.100659248 -2.924141346 stable

ARL6IP1 0.173180312 11.79742717 2.981342678 0.004944225 0.100659248 -2.925471145 stable

CD79A -0.612934263 12.05001209 -2.980792834 0.004951427 0.100659248 -2.926824552 stable

LOC101927181 0.33726051 4.768100701 2.98049264 0.004955362 0.100659248 -2.927563394 stable

PRO2852 -0.486544126 9.238656197 -2.980465104 0.004955724 0.100659248 -2.927631164 stable

TIMM9 -0.15902842 8.601000383 -2.980417055 0.004956354 0.100659248 -2.927749418 stable

LOC101928631 /// ZNF77 -0.08094873 3.592312691 -2.979887423 0.004963306 0.100659248 -2.929052826 stable

CYB561A3 -0.231053823 11.13272026 -2.979840116 0.004963928 0.100659248 -2.92916924 stable

OGFOD1 -0.176414044 8.256649712 -2.979571555 0.004967457 0.100659248 -2.929830098 stable

NFIA 0.028675073 3.314656857 2.979488028 0.004968555 0.100659248 -2.930035629 stable

HSPA1A /// HSPA1B 0.339360525 12.57304214 2.97879593 0.004977663 0.100741678 -2.9317385 stable

NAP1L2 0.504200699 6.279059366 2.978408036 0.004982775 0.100741678 -2.932692786 stable

RHOB 0.651153091 9.345792448 2.978203425 0.004985473 0.100741678 -2.933196133 stable

PARG 0.113399054 8.707532948 2.977811311 0.004990648 0.100759682 -2.934160681 stable

RLF 0.166668102 8.248682203 2.977007301 0.005001274 0.100887628 -2.936138187 stable

CDK5RAP3 -0.184638639 11.47474372 -2.976304949 0.005010575 0.100988625 -2.937865389 stable

TACC3 -0.156047295 10.5343969 -2.971670963 0.005072344 0.102146069 -2.949254772 stable

LOC102724517 /// NLK -0.281238905 5.259211974 -2.968552036 0.00511432 0.102903277 -2.956914211 stable

MOGS 0.14332566 8.840009282 2.968090382 0.005120561 0.102940787 -2.958047512 stable

LOC101929336 /// RIF1 0.133043706 9.153202205 2.966737449 0.005138892 0.103221073 -2.961368149 stable

IL18R1 0.495651795 8.529551051 2.964731608 0.005166182 0.103680692 -2.966289539 stable

CALM1 /// CALM2 /// CALM3 -0.246865321 11.54372309 -2.964160069 0.005173983 0.103748726 -2.967691446 stable

CD83 -0.596798691 9.245856771 -2.963111083 0.005188329 0.103875186 -2.970264024 stable

ALKBH1 0.286306991 9.448361858 2.963053187 0.005189122 0.103875186 -2.970405993 stable

TMEM181 0.266769565 10.00450247 2.961428779 0.005211415 0.104165607 -2.974388586 stable

GLRX2 0.30563054 8.762293754 2.961157893 0.005215141 0.104165607 -2.975052591 stable

HIST1H2BC -0.641840695 8.137362008 -2.961028934 0.005216916 0.104165607 -2.975368687 stable

ANK1 -0.359355941 4.66831943 -2.960308425 0.005226843 0.104194776 -2.977134584 stable

PDZK1 0.005515969 2.325683532 2.960279853 0.005227237 0.104194776 -2.977204606 stable

SDE2 0.402071017 6.045313817 2.959749125 0.005234561 0.104252412 -2.978505191 stable

SEC24C 0.157640111 10.68977118 2.959243945 0.005241541 0.104303113 -2.97974303 stable

TP53INP1 -0.205966276 11.44701424 -2.95614283 0.005284581 0.105070699 -2.987338778 stable

DARS -0.169078199 11.22381478 -2.954521476 0.005307216 0.105431611 -2.991308067 stable

EXOC8 0.227159532 8.947157759 2.953150539 0.005326426 0.105723931 -2.994663236 stable

HSP90AA1 0.129303933 14.22185261 2.952446762 0.005336312 0.105830863 -2.996385247 stable

PLA2G4A 0.719550145 7.219370802 2.948456054 0.0053927 0.106791118 -3.00614489 stable

ELP4 0.147356869 7.890586636 2.948377795 0.005393812 0.106791118 -3.006336195 stable

CISD3 0.178387936 8.688403652 2.947427617 0.005407322 0.106968571 -3.00865868 stable

NDFIP2 0.492611914 6.431867636 2.945101247 0.005440535 0.107477977 -3.014342948 stable

PRDX6 -0.206283696 12.66766004 -2.944984147 0.005442212 0.107477977 -3.014628997 stable

SIAH1 0.172500684 6.39666188 2.944166271 0.005453937 0.107506867 -3.01662668 stable

NDST1 -0.296422399 4.692248157 -2.943923823 0.005457418 0.107506867 -3.017218797 stable

DIEXF 0.272187608 7.810413913 2.943660247 0.005461204 0.107506867 -3.017862481 stable

C21orf91 0.245939635 8.571128707 2.943607784 0.005461958 0.107506867 -3.017990598 stable

HYDIN2 0.005813447 2.624365563 2.942240459 0.00548164 0.10780406 -3.021329142 stable

PATZ1 0.129474965 9.239510065 2.940696814 0.00550394 0.107889906 -3.025097027 stable

EBP 0.188950784 8.659381469 2.940617221 0.005505093 0.107889906 -3.025291271 stable

LOC339988 0.568406888 7.03325348 2.940604952 0.00550527 0.107889906 -3.025321213 stable

SPTY2D1 0.213367548 9.664972635 2.940541593 0.005506188 0.107889906 -3.025475835 stable

NOP58 -0.165076966 11.28324198 -2.940351547 0.00550894 0.107889906 -3.025939616 stable

CKS2 0.594820559 8.298339477 2.939326341 0.00552381 0.108036301 -3.028441169 stable

GABARAPL2 -0.101799556 12.78552017 -2.939203035 0.005525601 0.108036301 -3.028742003 stable

PNRC2 0.124663403 12.98587794 2.937412613 0.005551669 0.108455823 -3.03310928 stable

SNX29 -0.303409834 6.57725847 -2.936474734 0.00556537 0.108538205 -3.035396327 stable

RPS24 0.536724164 7.618522923 2.93620319 0.005569343 0.108538205 -3.036058409 stable

SLC30A1 0.442304627 10.39384033 2.936082614 0.005571108 0.108538205 -3.036352386 stable

L3MBTL1 0.15078129 3.205936436 2.93561249 0.005577994 0.108538205 -3.037498526 stable

PPP1R8 0.138446618 10.75584483 2.935546699 0.005578959 0.108538205 -3.037658913 stable

DNAJC1 0.286426125 8.946487249 2.93269874 0.005620854 0.10926289 -3.04459953 stable

LINC00938 -0.185048433 10.2803506 -2.931070987 0.005644931 0.109625508 -3.048564531 stable

WDR26 0.083607486 12.36834606 2.930598071 0.005651944 0.109625508 -3.049716233 stable

METRN 0.295959831 5.609709541 2.930493896 0.00565349 0.109625508 -3.049969917 stable

PDSS2 -0.174494153 8.279915491 -2.928237245 0.005687077 0.110185952 -3.055463843 stable

CD2BP2 0.126659599 9.103070687 2.927729826 0.005694655 0.110227305 -3.05669881 stable

OK/SW-CL.58 -0.16005511 2.927670854 -2.927466986 0.005698584 0.110227305 -3.057338463 stable

LOC100506530 0.013243148 2.705687543 2.926655088 0.005710737 0.110371607 -3.059314084 stable

SDPR -0.452980862 12.73801328 -2.925370825 0.00573001 0.110653168 -3.062438416 stable

RANBP6 0.169871274 10.12642618 2.924991298 0.005735717 0.110672516 -3.063361557 stable

GMPR -0.513970516 8.431014724 -2.924262385 0.005746693 0.110793413 -3.065134313 stable

ZBTB5 0.216593942 7.810028253 2.923390113 0.005759853 0.110956194 -3.067255356 stable

EIF1B -0.104199176 12.00282343 -2.921167581 0.005793514 0.11148114 -3.072657917 stable

SEPT5-GP1BB -0.145897073 6.978803109 -2.920965528 0.005796583 0.11148114 -3.073148941 stable

TNFRSF9 0.354516735 4.025462464 2.920635432 0.005801601 0.11148648 -3.073951084 stable

SETD3 0.165718077 10.41430598 2.920028202 0.005810841 0.111554759 -3.075426525 stable

STAG3 -0.499051185 6.261385381 -2.91965274 0.005816562 0.111554759 -3.076338717 stable

CALHM2 0.515290461 9.565599247 2.919467764 0.005819382 0.111554759 -3.076788094 stable

DDX27 -0.112231964 10.08295018 -2.91902282 0.005826171 0.111593954 -3.077868959 stable

LINC00961 -0.081888002 2.919257742 -2.915644032 0.005877969 0.112494465 -3.086073331 stable

DUSP22 -0.166574497 11.08319368 -2.914974963 0.005888276 0.112600122 -3.08769725 stable

DET1 -0.260598581 6.453845242 -2.910888816 0.005951597 0.113718537 -3.097609708 stable

DERL3 -0.415679742 7.438215962 -2.909307635 0.005976271 0.114097289 -3.101443069 stable

ADIPOR1 -0.208637131 11.5295121 -2.908223499 0.005993243 0.114253416 -3.104070645 stable

RNF20 0.113454437 11.11707605 2.908164802 0.005994163 0.114253416 -3.10421289 stable

TRG-AS1 0.363079191 10.55211854 2.906279554 0.006023789 0.114632884 -3.108780541 stable

SWAP70 -0.36372326 11.08670107 -2.905814839 0.006031113 0.114632884 -3.109906181 stable

CDK2AP2 0.241878041 10.74934444 2.90557962 0.006034823 0.114632884 -3.110475886 stable

TAZ -0.098671392 8.700057546 -2.905404189 0.006037592 0.114632884 -3.110900766 stable

BTBD1 0.094254812 12.03560751 2.905058942 0.006043044 0.114632884 -3.111736878 stable

SLC37A1 -0.167528864 7.8962585 -2.905041869 0.006043313 0.114632884 -3.111778225 stable

SGK494 -0.280655558 7.39508985 -2.904524133 0.006051498 0.11469564 -3.113031944 stable

MMP23A /// MMP23B 0.402857966 3.139472026 2.903915963 0.006061126 0.11478562 -3.114504472 stable

DPM2 -0.122814801 8.508903698 -2.903593668 0.006066234 0.11478993 -3.115284746 stable

ORAI2 -0.252245298 9.353420631 -2.903224064 0.006072096 0.114808503 -3.116179487 stable

LZTS3 -0.166564527 5.588758977 -2.902744029 0.006079719 0.114860288 -3.117341453 stable

MANEA 0.462337325 5.221741588 2.901886399 0.006093359 0.115025592 -3.119417113 stable

NFATC2 0.291923315 5.472083272 2.899872679 0.006125498 0.115539569 -3.124289233 stable

TOB2 0.339200509 7.856596078 2.898459484 0.006148148 0.115675891 -3.127707116 stable

SH2D3A -0.28213161 7.804723943 -2.898323119 0.006150338 0.115675891 -3.128036865 stable

ATAD2B 0.200629929 7.174582383 2.898234922 0.006151754 0.115675891 -3.128250134 stable

PDCD10 -0.124193804 12.46161551 -2.89819488 0.006152397 0.115675891 -3.128346956 stable

KIAA1432 0.020602427 2.958714751 2.897812974 0.006158536 0.115698817 -3.129270379 stable

NR3C1 0.121384147 12.34692819 2.896802423 0.006174806 0.11590828 -3.131713444 stable

ZMYND12 -0.516780927 4.242182954 -2.896508782 0.006179541 0.11590828 -3.132423236 stable

RP5-935K16.1 0.185361714 7.022773669 2.895045319 0.006203191 0.116259172 -3.135960047 stable

SKP1 -0.110687 10.63690639 -2.894703435 0.006208728 0.116270303 -3.136786126 stable

ATF7IP 0.132882743 6.232090229 2.894140419 0.006217857 0.116348622 -3.138146382 stable

CSTF1 0.299769776 7.053824651 2.891904943 0.006254227 0.116864758 -3.143545659 stable

HSD11B1L -0.050448588 3.294885611 -2.891834449 0.006255377 0.116864758 -3.143715876 stable

CAPN3 -0.273366757 8.280801284 -2.890834962 0.006271706 0.117076815 -3.146129004 stable

HGD -0.599259997 8.491899395 -2.889938155 0.00628639 0.117257879 -3.148293769 stable

COPZ2 0.444277689 4.616796146 2.889127584 0.006299691 0.117270445 -3.150250001 stable

TMEM91 -0.336683053 9.406864441 -2.889043985 0.006301064 0.117270445 -3.150451739 stable

CDKN1A -0.338840253 10.23059536 -2.888985705 0.006302021 0.117270445 -3.150592377 stable

PAXIP1-AS1 -0.251430244 9.203656991 -2.888245053 0.006314201 0.117322747 -3.152379502 stable

ZFP30 0.142848343 4.136504818 2.8878044 0.006321459 0.117322747 -3.153442618 stable

BRD8 0.121890165 10.12855343 2.887726263 0.006322746 0.117322747 -3.15363112 stable

KRTAP3-3 0.006908254 2.431838859 2.887425173 0.00632771 0.117322747 -3.154357454 stable

YAE1D1 0.371303753 7.481319137 2.887031936 0.006334199 0.117322747 -3.155306008 stable

LOC101928419 -0.655194926 7.311439116 -2.886793846 0.006338131 0.117322747 -3.15588028 stable

LOC100287210 0.007061712 2.481485191 2.886695959 0.006339748 0.117322747 -3.156116375 stable

EFNA4 0.261141003 6.743827862 2.885728405 0.006355753 0.117526469 -3.158449748 stable

POLE3 -0.110976914 10.75810463 -2.884583884 0.006374734 0.11778486 -3.16120925 stable

PPARA 0.173072073 5.047822209 2.884097467 0.006382817 0.117841638 -3.162381815 stable

RTP4 0.627480513 8.210656381 2.88341946 0.0063941 0.117957355 -3.164016019 stable

RBM28 0.109104049 8.559923843 2.883024607 0.006400679 0.117986192 -3.164967619 stable

SEPP1 0.099148858 3.205533311 2.882353629 0.006411874 0.118099996 -3.166584496 stable

MYOM2 1.218022496 9.313589642 2.089627899 0.043278318 0.035493351 -4.887994365 up

RAD51L3-RFFL /// RFFL 0.241579174 7.669442223 2.880445713 0.006443807 0.118502564 -3.171180735 stable

ZNF701 0.191405676 4.193873356 2.880113103 0.006449389 0.118512558 -3.171981805 stable

FOXO1 -0.319420097 9.360886393 -2.879344496 0.006462305 0.118657206 -3.173832716 stable

FYTTD1 0.08632292 11.88685286 2.878324359 0.006479486 0.11880452 -3.176288856 stable

GLYATL1 -0.193298072 4.473161259 -2.878221189 0.006481226 0.11880452 -3.176537223 stable

ITCH 0.215949297 7.117485769 2.877968975 0.006485481 0.11880452 -3.177144367 stable

PRR5 0.318661383 8.675189676 2.876955516 0.006502607 0.119025532 -3.179583679 stable

TOE1 -0.132629113 8.447178602 -2.876350667 0.006512847 0.119120282 -3.181039237 stable

TMEM194A 0.178747869 6.962912582 2.875742938 0.006523152 0.119216053 -3.182501528 stable

PPP1R14A -0.617789824 7.38585353 -2.875143484 0.006533331 0.119309386 -3.183943713 stable

DRG1 -0.123277126 10.61265689 -2.874756778 0.006539906 0.119336795 -3.184873959 stable

VPREB3 -0.830530456 7.8285272 -2.874147788 0.006550272 0.119433296 -3.186338761 stable

PHF11 -0.088573401 12.10305514 -2.873426597 0.006562568 0.119486313 -3.188073179 stable

GIN1 0.20193825 8.160041704 2.873381377 0.00656334 0.119486313 -3.188181922 stable

FBXO45 0.388823558 8.723308549 2.872801781 0.006573238 0.119573967 -3.189575597 stable

LINC00921 /// TIGD7 /// ZNF263 -0.223614032 3.068983454 -2.87228226 0.006582123 0.119643052 -3.190824662 stable

ZNF330 0.146665227 11.09345348 2.870865029 0.006606416 0.119991902 -3.194231322 stable

ZNF776 0.205096666 9.821357899 2.870340292 0.006615432 0.120062946 -3.195492381 stable

ZUFSP 0.19116732 8.457796844 2.870033598 0.006620707 0.120066036 -3.196229364 stable

RTKN 0.032672899 2.685849096 2.869384341 0.006631887 0.120176121 -3.197789357 stable

DANCR -0.23195506 9.952366643 -2.86871475 0.006643435 0.120276188 -3.199397969 stable

NPHP3 -0.239998402 3.495351301 -2.868302486 0.006650554 0.120276188 -3.200388262 stable

CCDC71L 0.240121662 8.240683973 2.868175462 0.00665275 0.120276188 -3.200693366 stable

PIGV 0.25279412 8.987213632 2.867788533 0.00665944 0.120304678 -3.201622693 stable

NEO1 0.396020162 3.840038309 2.865766472 0.006694507 0.120845354 -3.206477966 stable

AIMP1 -0.172513721 10.30393565 -2.864749524 0.006712208 0.121071966 -3.208918975 stable

APPBP2 0.099357791 9.896796027 2.863726758 0.006730054 0.121272414 -3.211373383 stable

CCDC23 -0.247491248 8.585812275 -2.863327753 0.006737029 0.121272414 -3.212330753 stable

SLFN13 0.415708082 6.597359043 2.863227134 0.006738789 0.121272414 -3.212572163 stable

UBXN7-AS1 -0.270555229 3.509917491 -2.862387427 0.006753492 0.121444096 -3.214586621 stable

UBAP2 -0.125344636 8.924856643 -2.861791931 0.006763937 0.121539005 -3.216014984 stable

IFIH1 -0.439075854 6.521622352 -2.861361053 0.006771504 0.121582095 -3.217048373 stable

RP11-16P6.1 -0.241555023 3.313175396 -2.86096703 0.006778431 0.121613631 -3.217993285 stable

GTF3C5 -0.106909336 6.280792298 -2.859844863 0.006798194 0.121794787 -3.220683903 stable

ADCY3 -0.150035272 7.050172899 -2.859805746 0.006798884 0.121794787 -3.220777681 stable

DHX8 0.171387904 9.598547579 2.85894583 0.006814067 0.121973871 -3.222839019 stable

SH2D3C 0.210240539 10.39101731 2.857543493 0.006838894 0.122184281 -3.226199756 stable

HELZ 0.110001314 10.74169318 2.85752075 0.006839298 0.122184281 -3.226254252 stable

NFIC -0.18455133 8.133619015 -2.857401923 0.006841405 0.122184281 -3.226538972 stable

CBLN3 -0.707968552 4.537014276 -2.856884057 0.006850598 0.122196908 -3.227779743 stable

GRASP -0.253301633 7.938246334 -2.856776855 0.006852503 0.122196908 -3.228036571 stable

PAK6 0.310970389 2.730392389 2.85589754 0.006868143 0.122383018 -3.230142961 stable

LOC100289283 -0.073236774 3.830029309 -2.855570732 0.006873964 0.122394023 -3.23092572 stable

DHRS7 0.17847105 11.70261876 2.854209909 0.006898253 0.122733588 -3.234184484 stable

PPP2R2A 0.085217523 10.30645175 2.852457461 0.00692965 0.123199009 -3.238379592 stable

POLL -0.111050259 8.250194983 -2.85209975 0.006936075 0.123220102 -3.239235694 stable

ZC3H12D -0.370011482 9.503883902 -2.851791013 0.006941625 0.123225626 -3.239974534 stable

TMEM133 0.170947758 3.223811251 2.850868895 0.006958226 0.123261359 -3.242180944 stable

GRB14 -0.753434763 6.300174787 -2.850825069 0.006959016 0.123261359 -3.242285799 stable

C5orf45 -0.276524398 8.409174135 -2.850581126 0.006963414 0.123261359 -3.242869414 stable

LZIC 0.146217064 8.139751281 2.85025526 0.006969293 0.123261359 -3.243648973 stable

GNA12 -0.170790184 8.23713114 -2.850118802 0.006971757 0.123261359 -3.243975398 stable

CREBRF -0.180008403 8.630218728 -2.84917766 0.006988769 0.123261359 -3.246226467 stable

TFAP2E 0.209514399 4.951488929 2.849031991 0.006991406 0.123261359 -3.246574842 stable

USP4 -0.065078445 10.53416702 -2.848993403 0.006992104 0.123261359 -3.246667125 stable

VAMP7 -0.111956965 11.74438073 -2.848874472 0.006994258 0.123261359 -3.246951545 stable

FERMT3 -0.182370548 12.11627806 -2.848775949 0.006996042 0.123261359 -3.247187153 stable

ZNF687 0.406660527 6.737567295 2.848079586 0.007008667 0.123391365 -3.248852284 stable

MIR4657 /// PURB 0.117122489 9.740587149 2.845666218 0.007052586 0.124071701 -3.254621039 stable

RAB28 0.179261102 7.62544406 2.845337136 0.007058594 0.124084595 -3.255407409 stable

AK023627 /// TCTE3 -0.687353142 3.599843347 -2.844329529 0.007077021 0.124315613 -3.257814805 stable

KIAA1377 0.47543442 4.057199307 2.843975809 0.0070835 0.124336572 -3.258659788 stable

B3GALT4 0.198091251 8.792526058 2.84207097 0.007118488 0.124720609 -3.263208984 stable

CCDC88C 0.247090554 9.379232257 2.842003263 0.007119734 0.124720609 -3.263370648 stable

FCGR3B 0.725494611 7.570456595 2.841918978 0.007121286 0.124720609 -3.263571889 stable

AMN1 -0.261039313 7.940871904 -2.841121051 0.007135996 0.124885236 -3.265476869 stable

GPATCH11 0.242493188 5.422788674 2.839133724 0.007172754 0.125435208 -3.270219916 stable

POLD1 -0.132578525 8.466876187 -2.837935958 0.007194994 0.125667593 -3.273077517 stable

CCL28 -0.349715188 6.976812001 -2.837842695 0.007196728 0.125667593 -3.273299987 stable

ESCO1 0.170929209 9.716624024 2.836779462 0.007216528 0.125919854 -3.275835911 stable

WDR11-AS1 -0.683432477 6.630558835 -2.83627876 0.00722587 0.125989394 -3.277029922 stable

AP001462.6 -0.32542964 3.741602189 -2.83593502 0.00723229 0.126007922 -3.277849551 stable

C8orf44 /// C8orf44-SGK3 /// SGK3 -0.342799888 5.704408492 -2.835625399 0.007238077 0.126015408 -3.278587771 stable

ORMDL1 -0.107399883 11.97057423 -2.834499407 0.00725916 0.126247863 -3.281271989 stable

FAM231D /// LINC00869 /// LOC388692 0.36731052 6.342380678 2.83433921 0.007262164 0.126247863 -3.281653822 stable

LOC155060 -0.299087127 6.682330704 -2.833098579 0.007285468 0.126507257 -3.284610414 stable

ATXN2 -0.11139433 9.115933876 -2.832972416 0.007287842 0.126507257 -3.28491103 stable

LCN2 -0.619244859 10.24563494 -2.832600759 0.007294839 0.126535331 -3.285796549 stable

CEP76 0.38769828 6.930710451 2.832021842 0.00730575 0.126568317 -3.287175736 stable

GNG7 -0.482992144 7.903311755 -2.831928941 0.007307503 0.126568317 -3.287397044 stable

RAB18 0.135046258 10.62367825 2.831596195 0.007313783 0.126583873 -3.288189664 stable

LINC00869 /// LOC100996741 0.401719883 9.402557986 2.831166992 0.00732189 0.126631018 -3.289211962 stable

LINC00324 0.373028868 8.839685779 2.830879174 0.007327332 0.126632019 -3.289897445 stable

RHEBL1 0.287774757 3.893907139 2.83050973 0.007334322 0.126656217 -3.290777265 stable

EGFL7 -0.540535782 4.455526676 -2.830236208 0.007339502 0.126656217 -3.291428603 stable

ZNF616 0.189524449 4.201766939 2.82841361 0.007374102 0.127146691 -3.295767698 stable

ZNF609 0.201406737 7.56958992 2.827880654 0.007384248 0.127146691 -3.297036172 stable

GUCD1 -0.178600707 10.33908741 -2.82770145 0.007387663 0.127146691 -3.297462656 stable

MEIS3 0.020354045 3.132030653 2.827602608 0.007389547 0.127146691 -3.29769788 stable

KRT10 -0.155863926 9.332231101 -2.827276269 0.00739577 0.12716075 -3.298474463 stable

C1GALT1C1 0.231901739 8.879187579 2.826207174 0.007416192 0.127326005 -3.301018158 stable

NPAT 0.207300503 10.43648399 2.82620634 0.007416208 0.127326005 -3.301020144 stable

LOC284630 /// TTC34 -0.158014515 3.751083786 -2.824189842 0.00745487 0.127896432 -3.305816281 stable

KDM6A 0.130490728 8.751629041 2.822691575 0.007483718 0.12829777 -3.309378378 stable

2-Mar -0.077581084 3.757754697 -2.821967274 0.007497702 0.128443873 -3.311099946 stable

DBNDD1 -0.159496342 3.155698763 -2.821209615 0.007512355 0.128601233 -3.312900492 stable

MARS2 0.322827445 7.392949799 2.820362605 0.007528767 0.128788465 -3.314912999 stable

P2RX5-TAX1BP3 /// TAX1BP3 -0.24932446 11.28519203 -2.819643198 0.007542734 0.128933607 -3.316622012 stable

SMIM20 -0.171208237 8.954571935 -2.818828183 0.007558585 0.12911074 -3.318557802 stable

PHLDA1 0.11960207 3.080709012 2.817315146 0.007588096 0.129520753 -3.322150533 stable

FBXO41 /// LOC101927826 -0.167704953 4.755912097 -2.816739048 0.00759936 0.12961896 -3.323518153 stable

CCDC102A 0.347390733 3.773009279 2.816306275 0.007607832 0.129669435 -3.324545409 stable

ZNF432 0.178538956 8.326836933 2.814050308 0.007652139 0.130330162 -3.32989863 stable

AK057657 /// RP11-473I1.9 -0.105129259 11.42748922 -2.81296651 0.007673509 0.130599578 -3.332469389 stable

SUN2 0.136672843 12.60450945 2.812357189 0.007685549 0.1307099 -3.333914409 stable

ADSL -0.098497565 10.54405235 -2.809232155 0.007747571 0.131669529 -3.341322264 stable

KIAA1958 -0.157951038 4.295207021 -2.808342953 0.007765304 0.131854816 -3.343429117 stable

GLE1 0.170960113 8.925746161 2.808123558 0.007769686 0.131854816 -3.343948879 stable

DPP10-AS1 0.010561612 2.802932195 2.807646589 0.007779218 0.131921406 -3.345078759 stable

NFE2L2 0.229955828 5.854590325 2.806985282 0.007792453 0.132050637 -3.346645102 stable

FAM219B 0.123873578 8.104093632 2.806598132 0.007800211 0.132086935 -3.347561977 stable

KLRC1 /// KLRC2 0.702814266 11.5077492 2.806043105 0.007811345 0.132180317 -3.348876283 stable

LOC642776 0.009930044 2.657800069 2.80398113 0.007852839 0.132786935 -3.353757552 stable

SPRED2 0.538710006 3.168453754 2.803607411 0.007860382 0.132818989 -3.354641996 stable

CARD11 0.294372284 7.684181879 2.803138849 0.007869848 0.132883479 -3.355750785 stable

SIGLEC8 -0.020741641 2.791776519 -2.801056269 0.00791205 0.133500227 -3.360677456 stable

KLRB1 0.474239184 12.76703899 2.800414439 0.007925098 0.133593787 -3.36219532 stable

HIST2H2AA3 /// HIST2H2AA4 -0.375619281 12.42559338 -2.800224965 0.007928954 0.133593787 -3.362643363 stable

C2CD2L 0.23044511 8.372980925 2.799450947 0.007944724 0.133679412 -3.36447345 stable

WBP11 -0.084405208 10.77526589 -2.799241364 0.007948999 0.133679412 -3.364968932 stable

BAZ2B 0.21497753 9.856474337 2.799139067 0.007951086 0.133679412 -3.365210765 stable

POU5F2 0.007659326 2.689133463 2.798367597 0.007966845 0.133848683 -3.367034362 stable

AP3M1 0.172891717 10.02412983 2.797470319 0.00798521 0.134014048 -3.369154926 stable

LHPP -0.208462616 8.102292843 -2.797084997 0.007993108 0.134014048 -3.370065432 stable

NMRK1 0.366139975 4.501678791 2.79705223 0.00799378 0.134014048 -3.370142856 stable

SNAI3 0.358072011 7.319850576 2.796352394 0.008008146 0.13415926 -3.371796324 stable

PPP1R12C -0.135648348 9.799456419 -2.795548853 0.00802467 0.134340398 -3.373694474 stable

ZAP70 0.317390694 11.77476283 2.794886009 0.008038324 0.13438314 -3.375259996 stable

ZMYM4 0.122934175 9.570904131 2.794870228 0.00803865 0.13438314 -3.375297265 stable

PPP3CB 0.074797596 11.47713308 2.793841609 0.008059883 0.134642399 -3.377726186 stable

ORC3 -0.122105883 8.922409158 -2.792766372 0.008082134 0.134719984 -3.380264559 stable

LOC101928623 /// LOC401320 -0.196828116 7.396687072 -2.792299254 0.008091818 0.134719984 -3.381367109 stable

SRCRB4D -0.166917493 6.468064204 -2.79219137 0.008094056 0.134719984 -3.381621732 stable

INPP4A 0.166125095 10.9411757 2.79184006 0.008101349 0.134719984 -3.382450835 stable

ZNF22 0.200654065 10.49548028 2.791838171 0.008101388 0.134719984 -3.382455293 stable

FAM109A -0.172354815 7.47085483 -2.791753277 0.008103151 0.134719984 -3.382655635 stable

CHMP5 0.300335293 9.745506229 2.791682532 0.00810462 0.134719984 -3.382822586 stable

FRG1 /// FRG1B /// LOC100134091 /// LOC100289097 /// LOC101930278 /// LOC101930531 /// LOC102724923 0.388817052 8.529688231 2.791369853 0.008111119 0.134732782 -3.383560433 stable

CYP4Z1 0.005866358 2.48960555 2.789794589 0.008143929 0.135149335 -3.387276848 stable

ZNF638 /// ZNF638-IT1 0.177352501 9.505528699 2.789614535 0.008147687 0.135149335 -3.387701549 stable

EGF -0.452758289 7.778532716 -2.789016257 0.008160186 0.135261273 -3.389112603 stable

SUV420H1 0.121799804 10.54590832 2.78863373 0.008168187 0.135298548 -3.390014698 stable

LSR -0.315119375 7.386779149 -2.786621515 0.008210395 0.135901979 -3.394758658 stable

NRBF2 0.392526546 9.711446779 2.786333687 0.008216449 0.135906545 -3.39543705 stable

TNFAIP1 0.295542971 7.463727307 2.785979343 0.008223908 0.135912309 -3.396272153 stable

RP11-53O19.3 0.194492817 8.863576055 2.785768251 0.008228355 0.135912309 -3.396769612 stable

MTMR6 0.11958099 10.82847022 2.784774722 0.008249312 0.135997967 -3.399110624 stable

ZNF706 -0.089327745 12.45056967 -2.784774277 0.008249321 0.135997967 -3.399111674 stable

SCMH1 -0.165902419 7.327806189 -2.784700174 0.008250886 0.135997967 -3.399286256 stable

SPATA5L1 -0.268283524 5.449188867 -2.783589286 0.008274382 0.136289737 -3.401903097 stable

EXOSC6 -0.252257681 7.968425591 -2.782920457 0.008288558 0.136313369 -3.403478275 stable

KLRC4-KLRK1 /// KLRK1 0.341925544 12.13217339 2.782913267 0.008288711 0.136313369 -3.403495208 stable

GCH1 0.269412527 12.66639717 2.782701539 0.008293203 0.136313369 -3.403993801 stable

SEPN1 -0.172726843 9.164016104 -2.780540978 0.008339175 0.136973282 -3.409080224 stable

RPUSD2 0.226198195 7.818876653 2.779753926 0.008355981 0.13715354 -3.41093246 stable

ASMTL-AS1 -0.01497319 2.817934176 -2.77942462 0.008363022 0.137161598 -3.41170734 stable

KIF21B 0.190921916 10.16369505 2.779185642 0.008368135 0.137161598 -3.412269634 stable

ZNF350 0.1551942 7.520301955 2.778675529 0.008379058 0.137175282 -3.413469778 stable

ZNF394 -0.278220925 10.51546086 -2.778602022 0.008380633 0.137175282 -3.413642707 stable

LOC100996740 -0.23391373 11.7466358 -2.777444584 0.008405473 0.137486178 -3.416365222 stable

ACTR6 0.147016652 9.502524241 2.776690579 0.008421691 0.137655727 -3.418138382 stable

RP11-429B14.4 0.009848904 2.744724817 2.776094417 0.008434534 0.13776992 -3.419540122 stable

SNN -0.238445445 12.42761328 -2.774888182 0.008460577 0.137851397 -3.422375702 stable

PHYKPL -0.187265633 10.66366491 -2.774674846 0.008465191 0.137851397 -3.42287712 stable

ESAM -0.618267023 8.152255212 -2.774581162 0.008467218 0.137851397 -3.423097303 stable

TOPORS 0.156237674 10.07638933 2.774553149 0.008467824 0.137851397 -3.423163139 stable

EIF2B1 -0.082530465 10.89025884 -2.774506805 0.008468827 0.137851397 -3.423272059 stable

SLC25A12 0.196407973 7.734782086 2.773497837 0.008490687 0.138111657 -3.425643063 stable

IFNG 1.215885196 3.468861783 3.439071802 0.001412174 0.045941542 -1.748611318 up

PRMT5 -0.198123443 7.51111258 -2.77231697 0.008516339 0.138337576 -3.428417288 stable

CFL1 0.053405457 14.45894106 2.77201694 0.008522868 0.138348087 -3.429122027 stable

SNCA -0.482742749 11.16067287 -2.770862738 0.008548028 0.138660804 -3.431832654 stable

ELOVL2-AS1 0.007311241 2.689070027 2.768381801 0.008602343 0.139445692 -3.437656556 stable

LINC01089 -0.304212453 9.443818856 -2.766771148 0.008637776 0.139923636 -3.44143564 stable

LOC102724162 -0.30603588 2.930383795 -2.766185272 0.008650698 0.140036522 -3.442809921 stable

LOC220729 /// SDHA /// SDHAP1 /// SDHAP2 -0.335876727 7.823324613 -2.76588331 0.008657365 0.140048064 -3.443518151 stable

CCDC19 -0.136459933 2.999200468 -2.765202375 0.008672418 0.140075548 -3.445115052 stable

MRPL10 0.203413468 9.938333962 2.765096098 0.008674769 0.140075548 -3.445364264 stable

TSC22D1 -0.334690971 12.53075559 -2.764998448 0.00867693 0.140075548 -3.445593241 stable

CYB5D2 -0.264953266 7.885256021 -2.764462202 0.008688807 0.140171075 -3.446850576 stable

MCUR1 -0.399044957 11.09056046 -2.763933992 0.00870052 0.140235817 -3.448088909 stable

HLA-DMA -0.243318273 12.517156 -2.763487206 0.00871044 0.140235817 -3.449136228 stable

ZDHHC23 -0.42053636 7.076328507 -2.763475196 0.008710707 0.140235817 -3.449164381 stable

FLJ10038 -0.24424133 8.346497862 -2.761945981 0.008744738 0.140679857 -3.452748179 stable

MATR3 -0.325915367 5.010733892 -2.761698814 0.00875025 0.140679857 -3.453327305 stable

KCTD7 /// RABGEF1 0.186543796 6.762770933 2.761324603 0.008758601 0.14071794 -3.454204036 stable

RP11-251G23.5 0.137008701 3.218860195 2.760216296 0.008783379 0.140915922 -3.456800196 stable

RASSF1 0.209365397 10.09536448 2.759726662 0.008794347 0.140915922 -3.45794692 stable

ATG4D 0.225324993 6.603933787 2.759705358 0.008794824 0.140915922 -3.457996811 stable

C9orf37 -0.095827669 6.862133826 -2.75970248 0.008794889 0.140915922 -3.458003551 stable

KCTD6 0.525867494 6.499478599 2.758641917 0.008818689 0.141201077 -3.460486914 stable

THBS3 -0.145704239 6.186395445 -2.757087289 0.008853685 0.141664979 -3.464126003 stable

SLC17A5 0.509479626 7.377286462 2.756133146 0.008875227 0.14191313 -3.466358791 stable

NAT1 0.468558622 8.377101845 2.753490731 0.008935141 0.142733926 -3.472539606 stable

DHFRL1 0.31458259 6.81754116 2.753334428 0.008938696 0.142733926 -3.472905086 stable

DDX21 -0.111804043 11.24207792 -2.751638932 0.008977351 0.143212107 -3.476868744 stable

COL7A1 -0.187713242 6.439109804 -2.751487128 0.00898082 0.143212107 -3.477223545 stable

HIST1H4I -0.156122022 7.087861632 -2.748834154 0.009041637 0.144004623 -3.483422044 stable

PARP3 -0.179826555 7.187152496 -2.748785183 0.009042763 0.144004623 -3.483536425 stable

CYP4F12 -0.317608689 5.111951034 -2.747997413 0.009060897 0.144195784 -3.485376204 stable

GNAQ -0.181463188 12.48380383 -2.747505134 0.009072247 0.144278781 -3.486525704 stable

RP11-378J18.8 -0.313037265 4.787737368 -2.746720272 0.009090369 0.144469302 -3.488358123 stable

ZNF45 0.182418484 7.275846338 2.745393751 0.009121074 0.144793583 -3.491454353 stable

UBFD1 0.252529674 7.036446987 2.745307 0.009123085 0.144793583 -3.491656802 stable

HIP1R -0.312999055 8.444124887 -2.743914123 0.009155436 0.14520905 -3.494906765 stable

GNG3 -0.030233276 3.07995814 -2.74340731 0.009167234 0.145279369 -3.496089024 stable

MSANTD2 0.201354435 7.936813787 2.743193167 0.009172223 0.145279369 -3.496588518 stable

IL5RA 0.00631137 2.310745784 2.742923594 0.009178507 0.14528107 -3.497217266 stable

LELP1 -0.031417242 3.652201747 -2.742359391 0.009191672 0.145391613 -3.498533072 stable

RPL27A /// SNORA45A 0.033208413 15.84381696 2.741036268 0.009222615 0.145783017 -3.501618082 stable

CIDEC -0.207007385 5.291519832 -2.737611458 0.009303157 0.146957389 -3.509598763 stable

CCDC53 -0.108550659 10.53360483 -2.736780227 0.009322803 0.147168894 -3.511534733 stable

ZBTB49 0.182770776 8.239004228 2.735349471 0.009356709 0.147423718 -3.514866093 stable

HIST1H2BA 0.017078092 2.944528819 2.735323658 0.009357322 0.147423718 -3.514926185 stable

DTX1 -0.305265976 7.212885414 -2.735279108 0.00935838 0.147423718 -3.515029894 stable

ST3GAL4-AS1 0.452992734 5.197972947 2.735041753 0.009364017 0.147423718 -3.515582428 stable

FBXO5 0.245119801 7.77970784 2.733595647 0.009398428 0.147833373 -3.518948083 stable

LOC102724809 0.008009747 2.531496687 2.733306276 0.009405327 0.147833373 -3.519621419 stable

ICAM2 0.137860437 11.61657812 2.733156836 0.009408892 0.147833373 -3.519969133 stable

MIR1204 /// PVT1 -0.249928182 9.065683995 -2.730735815 0.009466824 0.148620648 -3.525600505 stable

UPF1 0.172085548 7.433161852 2.730535343 0.009471636 0.148620648 -3.52606666 stable

RMI1 0.339228839 8.986452782 2.729488897 0.00949679 0.148915999 -3.528499567 stable

LMNTD2 -0.191478667 6.254581213 -2.729187542 0.009504045 0.14893048 -3.529200079 stable

AKAP5 0.373945257 3.511463839 2.727456028 0.009545832 0.149485699 -3.533224036 stable

ZNF765 0.236541417 8.948941218 2.726881789 0.009559728 0.149533809 -3.534558159 stable

BC017988 /// RP11-308N19.1 0.438880241 3.170564679 2.726803687 0.009561619 0.149533809 -3.5347396 stable

ALG6 0.119277707 9.630146278 2.726306335 0.009573672 0.149622815 -3.535894918 stable

SNX29P1 /// SNX29P2 -0.49245501 5.489293317 -2.725718396 0.009587938 0.149746272 -3.537260482 stable

NDST2 /// ZSWIM8-AS1 -0.363556298 7.304539966 -2.724289697 0.009622686 0.150189252 -3.540577987 stable

RBM25 -0.317650834 8.756471893 -2.723853502 0.009633318 0.150255493 -3.541590619 stable

LOC101926963 -0.22245743 5.832328311 -2.723387727 0.009644684 0.150296469 -3.5426718 stable

F13A1 -0.427891705 12.90871622 -2.723222216 0.009648725 0.150296469 -3.543055963 stable

TRADD -0.100900519 10.44456402 -2.722687402 0.009661796 0.150400459 -3.544297196 stable

RTN4R 0.338830103 3.202014997 2.720915041 0.009705227 0.15092162 -3.548409436 stable

FAM210B -0.13686665 10.24865186 -2.720797743 0.009708108 0.15092162 -3.548681529 stable

TRIM23 0.28348107 5.562576245 2.719436365 0.009741601 0.151319014 -3.551838883 stable

IQGAP2 0.162984788 11.27671677 2.719236063 0.009746537 0.151319014 -3.55230334 stable

CCR4 0.173044859 4.127827648 2.718778603 0.009757821 0.151394268 -3.553364007 stable

GRPEL2 0.241103338 9.00399392 2.71709414 0.009799474 0.1519403 -3.557268554 stable

LINC00528 -0.240981965 6.772737153 -2.716555889 0.009812819 0.151983804 -3.558515863 stable

WNT9A 0.008778489 2.427264543 2.716459777 0.009815203 0.151983804 -3.55873857 stable

CRLF3 0.103627514 12.53565323 2.716181366 0.009822114 0.151990751 -3.559383661 stable

ZNF211 0.180552512 8.639652612 2.715626568 0.009835898 0.152092889 -3.56066902 stable

TPD52 -0.355722308 10.27356501 -2.715395408 0.009841647 0.152092889 -3.56120452 stable

TSHB 0.021878704 2.504865789 2.714793131 0.009856639 0.152224566 -3.562599597 stable

OSBPL5 0.694564496 7.059792722 2.714184364 0.009871815 0.152272398 -3.564009497 stable

KDM1A -0.092703353 9.272402779 -2.714149511 0.009872684 0.152272398 -3.564090208 stable

PDDC1 -0.111082036 9.011387664 -2.713452511 0.009890087 0.152396272 -3.565704177 stable

LANCL3 0.124924102 3.003109335 2.713308993 0.009893674 0.152396272 -3.566036469 stable

GNAS -0.080336959 14.92268713 -2.712901336 0.009903869 0.152453473 -3.566980274 stable

MIR29B2 /// MIR29C 0.182706664 8.542522617 2.711170533 0.009947263 0.153021305 -3.570986342 stable

BRD4 -0.132022088 11.43777673 -2.709338249 0.009993393 0.153497114 -3.575225406 stable

IPO7 0.07873295 10.92583822 2.708813428 0.010006642 0.153497114 -3.576439245 stable

CTC-523E23.1 0.571926815 5.42324951 2.708798635 0.010007016 0.153497114 -3.576473455 stable

ZC3H3 -0.154860442 8.413494573 -2.708498544 0.0100146 0.153497114 -3.57716745 stable

CORO2B -0.422114942 2.811186494 -2.708441251 0.010016048 0.153497114 -3.57729994 stable

ETFB -0.14933774 9.745258154 -2.70838979 0.010017349 0.153497114 -3.577418943 stable

UBR5 0.127193381 9.977254576 2.707628006 0.010036629 0.15367651 -3.579180373 stable

RP11-1191J2.5 -0.232819097 6.023985809 -2.707411132 0.010042124 0.15367651 -3.579681776 stable

SLC33A1 0.167256477 8.90539756 2.706695815 0.010060268 0.153854072 -3.581335367 stable

HCN2 0.117957439 4.086879434 2.705601215 0.010088091 0.154179334 -3.583865167 stable

LOC100129973 -0.450942572 3.976209177 -2.705064488 0.01010176 0.154287988 -3.585105377 stable

F8A1 /// F8A2 /// F8A3 -0.103082104 11.38499381 -2.703960131 0.010129939 0.154495097 -3.587656681 stable

LOC283357 0.237116876 9.312722235 2.703659145 0.010137631 0.154495097 -3.588351901 stable

C9orf16 -0.182856921 11.17170516 -2.703398275 0.010144303 0.154495097 -3.588954419 stable

ZNF420 0.430055246 6.799030656 2.703301997 0.010146766 0.154495097 -3.589176777 stable

STOM 0.232153811 11.26519955 2.703247449 0.010148162 0.154495097 -3.589302756 stable

DDX60L 0.259417543 3.14323146 2.702255573 0.010173575 0.154781798 -3.59159319 stable

GPKOW 0.148609756 8.987702802 2.701973982 0.0101808 0.154791601 -3.592243336 stable

OTUD3 -0.165815821 8.099220084 -2.70068213 0.010214009 0.155138781 -3.595225406 stable

ESD -0.127018409 11.76822397 -2.700572712 0.010216826 0.155138781 -3.59547794 stable

CTSA -0.262096672 12.67420668 -2.700070461 0.010229768 0.155235073 -3.596637025 stable

ID1 -0.655580779 3.12054891 -2.699017469 0.010256949 0.155463377 -3.599066628 stable

ZNF18 0.154782036 8.386563833 2.698975574 0.010258032 0.155463377 -3.599163282 stable

ZFYVE26 0.224164077 8.485934237 2.69712925 0.010305857 0.156087616 -3.603421764 stable

TNN 0.009350511 2.787517505 2.696369628 0.010325594 0.156285899 -3.605173231 stable

ARSG 0.19509713 8.131981286 2.694858835 0.010364951 0.15678071 -3.608655679 stable

FAM114A2 0.095642231 8.389016012 2.694569777 0.010372497 0.156794018 -3.609321821 stable

LOC100134445 -0.318489082 9.435870902 -2.69301882 0.010413072 0.157306265 -3.612895207 stable

FHL3 0.273335856 6.73535126 2.692024478 0.010439162 0.157599182 -3.615185423 stable

HDC -0.814010717 7.738515329 -2.690966401 0.010466991 0.157917954 -3.617621803 stable

OTULIN 0.234903754 7.131862176 2.689294693 0.010511099 0.158481768 -3.621469828 stable

DGCR2 0.177400434 8.161081248 2.688673483 0.010527533 0.158627875 -3.622899346 stable

FBXL14 0.0486378 2.414811172 2.687938617 0.010547005 0.158819533 -3.624590117 stable

EXOC2 0.17622516 8.858121373 2.687196511 0.010566703 0.159011352 -3.626297225 stable

MIR1292 /// NOP56 /// SNORD110 /// SNORD57 /// SNORD86 -0.119727081 8.618356755 -2.686949578 0.010573265 0.159011352 -3.626865188 stable

ST6GALNAC6 0.180265538 6.85110058 2.685670982 0.010607301 0.159421298 -3.629805476 stable

FUZ -0.177277862 7.507674314 -2.685140476 0.010621453 0.159532055 -3.63102516 stable

BAGE2 /// BAGE3 /// BAGE5 /// KMT2C 0.180472044 4.19096872 2.682649683 0.010688132 0.160416022 -3.636749529 stable

GORASP2 0.123041028 10.62526296 2.682383381 0.010695284 0.160416022 -3.637361332 stable

YWHAH -0.273032333 11.17300145 -2.682179313 0.010700767 0.160416022 -3.637830131 stable

ZNF304 0.301695001 7.757124225 2.681836374 0.010709988 0.160451987 -3.638617899 stable

BZW2 -0.12910677 10.69661602 -2.681369903 0.010722542 0.160536638 -3.639689322 stable

NOL11 0.129649559 10.77849461 2.68111942 0.010729289 0.160536638 -3.640264597 stable

PPP2R3C 0.068755167 4.001761436 2.679870715 0.010762981 0.160938384 -3.643131899 stable

KIAA0247 0.107006999 11.6365483 2.679288517 0.010778723 0.161038113 -3.644468442 stable

KBTBD6 0.255659008 4.144417841 2.679117789 0.010783344 0.161038113 -3.644860344 stable

USO1 0.129176837 10.35417688 2.678239486 0.010807142 0.16129111 -3.646876187 stable

RP11-672L10.6 -0.463675991 4.512633735 -2.677902891 0.010816275 0.161325055 -3.647648605 stable

LOC101927752 -0.176675544 8.450429793 -2.677097053 0.01083817 0.161549173 -3.649497574 stable

CCT6P1 /// CCT6P3 -0.237448355 9.018227673 -2.676205975 0.010862428 0.161808216 -3.651541678 stable

UBE2Q2 0.16441674 10.64493326 2.675644256 0.010877746 0.161933835 -3.652830005 stable

BCKDHB -0.217930857 7.910480618 -2.675178529 0.010890461 0.162020578 -3.653898031 stable

VGLL4 0.167750838 9.496791217 2.673254369 0.01094314 0.162701382 -3.658309244 stable

LRIF1 0.236843417 9.482989006 2.672218854 0.010971587 0.163021281 -3.660682304 stable

MGC24103 0.586341134 3.109832484 2.671930567 0.010979518 0.163036144 -3.661342851 stable

LANCL1 0.164849312 7.894855406 2.670723684 0.011012781 0.163426897 -3.664107629 stable

SCAMP1 0.170481075 8.711591663 2.670461708 0.011020014 0.163431116 -3.664707663 stable

PAX5 -0.228279577 3.435339646 -2.669416012 0.011048927 0.163515476 -3.667102334 stable

ZNF318 -0.132103717 9.22871867 -2.669406558 0.011049189 0.163515476 -3.66712398 stable

KCTD5 0.258471388 9.811030258 2.669314652 0.011051734 0.163515476 -3.667334417 stable

NAV3 0.008428243 2.809735253 2.669250505 0.01105351 0.163515476 -3.667481291 stable

TEFM 0.339574334 7.679329963 2.668709866 0.011068492 0.163634187 -3.668719064 stable

IGH /// IGHA1 /// IGHA2 /// IGHD /// IGHG1 /// IGHG3 /// IGHG4 /// IGHM /// IGHV3-23 /// IGHV4-31 -0.565564304 10.63316337 -2.6676392 0.011098216 0.163970569 -3.671169807 stable

AC079767.4 -0.652137152 7.202821594 -2.664398397 0.01118864 0.165051952 -3.678583854 stable

CLK4 0.331571231 6.769842073 2.664305647 0.011191237 0.165051952 -3.678795948 stable

LOC441155 /// ZC3H11A 0.219363902 6.239964899 2.664261986 0.01119246 0.165051952 -3.678895789 stable

UBB 0.075013352 14.71992329 2.663795565 0.011205534 0.165141209 -3.67996228 stable

BLOC1S1-RDH5 /// RDH5 -0.175122811 6.404745596 -2.658314026 0.011360241 0.16731636 -3.692486458 stable

SPAG1 0.286841035 5.979650271 2.657535926 0.01138236 0.167537233 -3.694262815 stable

ZNF182 0.143791748 8.440325753 2.657170466 0.011392763 0.167574003 -3.695097016 stable

TSPYL2 -0.254210087 9.219646257 -2.656947741 0.011399107 0.167574003 -3.695605372 stable

LETMD1 -0.119805877 10.05177194 -2.656056965 0.011424513 0.167725428 -3.697638213 stable

ZNF611 0.093599368 3.574927062 2.655903323 0.0114289 0.167725428 -3.697988793 stable

PKM -0.150145988 13.55306247 -2.655836796 0.011430801 0.167725428 -3.698140591 stable

TBPL1 0.101394241 10.06667949 2.655490515 0.011440696 0.167765966 -3.698930668 stable

PINLYP -0.172531548 5.925960616 -2.655228907 0.011448177 0.167771072 -3.699527508 stable

RAB14 0.09741828 11.17603255 2.654083145 0.011480994 0.168106346 -3.702141002 stable

MRPS12 -0.174782353 3.562194354 -2.653845068 0.011487824 0.168106346 -3.702683961 stable

ZCCHC6 0.187222532 10.38662821 2.653682296 0.011492496 0.168106346 -3.70305516 stable

NAB1 0.202358695 6.638203169 2.652541312 0.011525293 0.168481307 -3.705656713 stable

CCZ1B -0.331756947 9.2584274 -2.651937752 0.011542677 0.168630628 -3.707032577 stable

TMEM55A -0.171383681 9.027720148 -2.65051962 0.011583618 0.169123697 -3.710264473 stable

ZNF257 0.238578473 3.659765372 2.649949823 0.011600105 0.169259352 -3.711562692 stable

VLDLR 0.011712649 2.631017878 2.649561998 0.01161134 0.169318238 -3.7124462 stable

ZNF574 0.36096907 7.3262829 2.649107367 0.011624522 0.169377084 -3.713481784 stable

ACTR8 0.225831868 5.975474205 2.64892625 0.011629777 0.169377084 -3.713894309 stable

ARL4A -0.308455955 8.846462421 -2.648595015 0.011639394 0.169412248 -3.714648702 stable

MINK1 -0.255778226 8.13297424 -2.64792804 0.011658781 0.169549138 -3.716167553 stable

SAMD3 0.586826275 3.768197853 2.647775615 0.011663216 0.169549138 -3.716514622 stable

PDE4A 0.385184535 8.107379546 2.644870831 0.011748027 0.170676558 -3.723126116 stable

CHCHD10 -0.148152992 11.56196552 -2.644001034 0.011773533 0.170941524 -3.72510486 stable

GPR68 0.116196654 2.736831112 2.643360121 0.01179236 0.171109249 -3.726562616 stable

HOXA1 0.550944855 3.703570266 2.642383483 0.011821102 0.171389289 -3.728783511 stable

ACO1 0.183622617 7.295048541 2.642209365 0.011826233 0.171389289 -3.7291794 stable

MTA1 -0.173628374 8.409621903 -2.641547672 0.01184575 0.171566437 -3.730683713 stable

SLC26A2 0.182414489 8.968906939 2.640491032 0.011876979 0.171912878 -3.733085369 stable

IFT20 -0.117742238 10.82611056 -2.640128046 0.011887725 0.17196259 -3.733910254 stable

LOC100129361 -0.134250013 10.86763601 -2.639857902 0.011895728 0.171972592 -3.734524102 stable

NAP1L3 0.455004391 5.995319645 2.639527449 0.011905524 0.172008492 -3.735274935 stable

KANSL2 0.118278374 9.655515838 2.634361123 0.012059649 0.174128299 -3.747005032 stable

C6orf226 -0.171718953 7.343219159 -2.633563958 0.012083593 0.174366993 -3.748813572 stable

GSTK1 -0.091224406 12.27291418 -2.631196234 0.012154971 0.175289438 -3.75418302 stable

SENCR -0.264962259 6.889546783 -2.630827407 0.012166125 0.17534278 -3.755019133 stable

RPP38 0.282627634 8.809517477 2.630120016 0.012187543 0.175543906 -3.756622528 stable

ANAPC2 -0.147412664 8.776507243 -2.62871286 0.012230252 0.17605126 -3.759811138 stable

TRMT1L 0.184867648 9.024132994 2.627751926 0.012259497 0.176099207 -3.761987933 stable

ZNF445 0.174518677 7.645616005 2.627509496 0.012266885 0.176099207 -3.762537021 stable

PARD3 -0.226779295 6.459126626 -2.62749503 0.012267326 0.176099207 -3.762569782 stable

RLTPR -0.289008061 8.009547028 -2.627298829 0.012273309 0.176099207 -3.763014138 stable

FKTN -0.202839816 8.144245562 -2.627174643 0.012277097 0.176099207 -3.763295381 stable

SLC30A6 0.12185538 8.846189536 2.627128503 0.012278504 0.176099207 -3.76339987 stable

ALAS1 0.251519108 9.343510736 2.62661306 0.01229424 0.17621744 -3.764567079 stable

TTC21A -0.367040964 6.459279104 -2.625940354 0.012314805 0.176234309 -3.766090165 stable

EEF1E1 -0.101904199 9.849361812 -2.625916268 0.012315542 0.176234309 -3.766144693 stable

CLSTN3 0.316004469 7.258412315 2.62583936 0.012317895 0.176234309 -3.766318805 stable

ITPKB -0.561612171 8.658335946 -2.62536986 0.01233227 0.176332713 -3.767381619 stable

JAK1 0.16770703 12.88826799 2.624488432 0.012359298 0.176611816 -3.769376566 stable

ZNF14 0.348088306 7.852614218 2.623736533 0.012382398 0.176834472 -3.771077982 stable

C1orf186 /// LOC101929219 -0.268713574 5.753956885 -2.623110663 0.012401656 0.177002029 -3.772493957 stable

FOXN2 0.248625614 6.828662862 2.622642532 0.012416078 0.177100409 -3.773552909 stable

FARSA 0.128457982 8.998642072 2.622121672 0.012432143 0.177158229 -3.774730982 stable

TCEA3 -0.329062874 7.651737124 -2.622022779 0.012435196 0.177158229 -3.774954638 stable

TSEN15 0.175206766 9.516982168 2.621778202 0.012442747 0.177158513 -3.775507748 stable

SEPHS1 0.120371235 8.930027905 2.621076145 0.012464449 0.177294223 -3.777095249 stable

MIR4746 /// UBXN6 -0.134308292 8.891166141 -2.62088491 0.012470366 0.177294223 -3.777527622 stable

UBE2L3 0.175049273 8.427230929 2.620738683 0.012474892 0.177294223 -3.777858218 stable

HIPK1 0.149688823 3.571933293 2.618804955 0.012534891 0.178039361 -3.78222887 stable

SH3BP5L 0.219350049 7.788622594 2.618336171 0.012549477 0.178138951 -3.783288086 stable

PREP 0.133526803 9.253055156 2.617832499 0.012565165 0.17816058 -3.784425987 stable

ZNF416 0.486968907 4.957821924 2.617800904 0.01256615 0.17816058 -3.784497362 stable

ZFP1 0.215529461 7.833058562 2.616034276 0.012621322 0.178745143 -3.788487301 stable

TRUB1 0.180064644 8.924949099 2.615994112 0.012622579 0.178745143 -3.788577991 stable

ALS2CL -0.146581103 7.718501605 -2.614669267 0.012664105 0.179225272 -3.791568899 stable

IFITM1 0.168241905 13.27675353 2.613277847 0.012707852 0.179707194 -3.794708967 stable

ITPK1-AS1 0.281549676 3.361927716 2.6131005 0.012713438 0.179707194 -3.795109108 stable

CITED2 0.257806772 10.65887072 2.611530373 0.012762991 0.180299282 -3.798650905 stable

HMGA1 -0.140304197 11.37370657 -2.61123259 0.012772409 0.180324025 -3.799322458 stable

RABGGTB /// SNORD45A /// SNORD45B /// SNORD45C -0.19196829 11.38835097 -2.609964349 0.012812591 0.180782815 -3.802181968 stable

DDX23 0.136969361 9.560661251 2.608979176 0.012843885 0.181115716 -3.804402574 stable

CUL4A 0.091593996 10.72608632 2.608535327 0.012858007 0.181206214 -3.805402829 stable

ZNF557 0.715329215 4.999165029 2.607002892 0.012906873 0.181594056 -3.808855405 stable

YARS 0.133027968 10.42281689 2.606915241 0.012909673 0.181594056 -3.80905284 stable

ARFRP1 -0.127814072 8.644642685 -2.606724419 0.012915771 0.181594056 -3.809482651 stable

RAB27A 0.192781771 11.29047751 2.60670447 0.012916409 0.181594056 -3.809527584 stable

TBC1D9B -0.118758212 8.539354845 -2.60614869 0.012934186 0.181735358 -3.810779308 stable

MTIF2 -0.078673243 9.593512168 -2.604409761 0.012989952 0.182409949 -3.814694501 stable

LDB2 0.689525805 3.557205002 2.602941077 0.013037224 0.182834527 -3.817999818 stable

SPRY2 0.342747405 3.556905954 2.602766056 0.013042868 0.182834527 -3.818393619 stable

CIZ1 -0.105670377 10.14734118 -2.60274622 0.013043507 0.182834527 -3.818438251 stable

MCOLN2 0.463697737 6.03941044 2.602324887 0.013057104 0.182916102 -3.819386182 stable

DCUN1D1 0.145251593 9.370916309 2.601712653 0.013076884 0.183084154 -3.820763419 stable

ZNF358 -0.152977979 6.610906612 -2.601014386 0.013099477 0.183291368 -3.822333915 stable

N4BP2L2-IT2 -0.254639423 8.102772054 -2.600142605 0.013127734 0.183577548 -3.824294252 stable

MYEOV -0.268575573 4.665637939 -2.598851041 0.013169701 0.184054988 -3.82719769 stable

POLD4 -0.129172371 10.55532947 -2.598394032 0.013184581 0.184153515 -3.828224806 stable

MMD -0.338784749 13.30513798 -2.597548635 0.013212146 0.184359214 -3.830124481 stable

LOC101929715 -0.063000885 2.880831237 -2.597461681 0.013214984 0.184359214 -3.830319848 stable

IL21R 0.202882571 7.652827955 2.596082712 0.013260069 0.184807701 -3.833417501 stable

BZW1 0.118129905 11.70724066 2.595878935 0.013266743 0.184807701 -3.833875157 stable

DGKK 0.783656518 2.99830253 2.595698371 0.01327266 0.184807701 -3.834280661 stable

WDR36 0.141516433 9.3919149 2.595518387 0.01327856 0.184807701 -3.834684841 stable

AK128734 /// JAK1 0.702993595 5.409797025 2.595039753 0.013294262 0.184916818 -3.83575959 stable

SMIM3 -0.270530206 9.995594997 -2.593604191 0.013341459 0.185463628 -3.838982239 stable

KDM5A 0.171968055 10.57919027 2.592696928 0.013371366 0.185700733 -3.84101828 stable

ADAMTS7 -0.049031579 2.692967628 -2.592352208 0.013382746 0.185700733 -3.841791754 stable

PABPN1 -0.189741332 9.548442656 -2.591677545 0.013405043 0.185700733 -3.84330534 stable

PHRF1 0.11516685 8.260460646 2.591671451 0.013405245 0.185700733 -3.843319011 stable

KIAA1683 -0.390146182 4.599879646 -2.591287082 0.013417963 0.185700733 -3.844181206 stable

HDAC6 -0.091975472 9.518623263 -2.591237747 0.013419597 0.185700733 -3.844291866 stable

CTSK -0.298965261 7.432416523 -2.591176208 0.013421634 0.185700733 -3.844429895 stable

TREML2 -0.186734255 6.246552539 -2.591174938 0.013421676 0.185700733 -3.844432744 stable

DUS4L -0.207111479 7.889072634 -2.590408949 0.013447062 0.185942588 -3.846150649 stable

HLA-G 0.042252118 13.49584151 2.588784512 0.013501043 0.186562088 -3.849792639 stable

RP11-21L23.2 0.015707479 3.140221555 2.588516512 0.013509968 0.186562088 -3.850393341 stable

TUBB2A /// TUBB2B -0.198466339 5.932178647 -2.588345734 0.013515658 0.186562088 -3.850776104 stable

RNF6 0.160910116 11.70910233 2.587071975 0.013558168 0.187039097 -3.853630411 stable

TROAP -0.079920028 3.299480976 -2.58658101 0.013574585 0.187155816 -3.854730329 stable

IFITM1 /// IFITM2 0.167959401 13.40632726 2.585380163 0.013614818 0.187600553 -3.857419991 stable

TMEM39A 0.379272646 7.383460081 2.58293057 0.013697229 0.188533615 -3.862903875 stable

CUL5 0.064491845 10.7958323 2.582468957 0.01371281 0.188533615 -3.863936877 stable

NCOR1 0.104426686 10.81262721 2.582436692 0.013713899 0.188533615 -3.864009075 stable

PSIP1 -0.399699132 6.13635706 -2.582416061 0.013714596 0.188533615 -3.864055239 stable

EMC3 -0.277678938 5.307528032 -2.581522912 0.013744793 0.188720717 -3.866053536 stable

RP11-309G3.3 0.264996751 3.912667727 2.581441422 0.013747551 0.188720717 -3.866235835 stable

UMPS 0.23951003 8.078614326 2.580957993 0.013763923 0.188720717 -3.867317214 stable

UBE2E3 -0.107656803 12.25282591 -2.580839533 0.013767938 0.188720717 -3.867582174 stable

RAB32 -0.228903133 10.78725909 -2.580828147 0.013768324 0.188720717 -3.867607639 stable

TBC1D2B 0.17432468 9.642226572 2.579479699 0.013814101 0.189237899 -3.870623114 stable

KRBA1 -0.121979241 7.783852152 -2.578449191 0.013849179 0.189515349 -3.872926843 stable

PDCD1LG2 0.006020602 2.292637018 2.57841133 0.013850469 0.189515349 -3.87301147 stable

CSNK1D 0.145901671 11.14764781 2.576838402 0.013904173 0.190079033 -3.876526512 stable

USP24 0.115842615 10.36556741 2.576721797 0.013908161 0.190079033 -3.876787032 stable

TLR10 -0.474590275 8.917449956 -2.576495396 0.013915909 0.190079033 -3.877292832 stable

SMARCC2 -0.622069143 6.764858795 -2.576086228 0.013929921 0.190099044 -3.878206872 stable

NFKBIZ -0.406883241 11.21285675 -2.575980665 0.013933538 0.190099044 -3.878442672 stable

KLHL6 0.247244005 7.05393764 2.575479676 0.013950716 0.190223072 -3.879561657 stable

HMG20B -0.110196374 8.531327212 -2.574806293 0.013973836 0.190297861 -3.881065456 stable

TMEM168 0.121092171 9.899635409 2.574736963 0.013976219 0.190297861 -3.881220267 stable

LOC100130938 -0.546779138 7.626092465 -2.574613186 0.013980473 0.190297861 -3.881496649 stable

DEDD2 0.155633601 11.69486116 2.572696366 0.014046506 0.191086104 -3.885775542 stable

LOC202181 -0.278402615 7.537931001 -2.572147752 0.014065458 0.19123332 -3.886999793 stable

TMPRSS11D -0.069460157 2.886178923 -2.571416285 0.014090763 0.191466693 -3.888631799 stable

KIFC2 -0.304314518 8.129310773 -2.570875824 0.014109488 0.191610425 -3.889837436 stable

TIRAP 0.279614297 6.827883447 2.570190048 0.014133279 0.191822765 -3.891366974 stable

KIF3C -0.303372336 5.983511227 -2.568968604 0.014175745 0.192288177 -3.894090543 stable

AL832909 /// LINC00984 -0.267047356 10.57418707 -2.568209404 0.014202199 0.192535981 -3.895782943 stable

RP11-271C24.3 -0.357294064 9.869049098 -2.564632578 0.014327443 0.193906966 -3.903751608 stable

SDHC 0.134530813 10.04888863 2.564439036 0.014334248 0.193906966 -3.904182568 stable

LINC01289 0.010240966 2.83827404 2.564210918 0.014342274 0.193906966 -3.90469049 stable

SLC18B1 -0.193016284 9.76446241 -2.564173588 0.014343587 0.193906966 -3.904773605 stable

IGBP1 -0.098176215 10.96201843 -2.564146282 0.014344548 0.193906966 -3.904834401 stable

IGFALS -0.126586835 4.127652618 -2.563905386 0.014353029 0.193910162 -3.905370729 stable

RTN2 -0.375107533 6.691127258 -2.563345098 0.014372771 0.194065414 -3.906618012 stable

LOC102723692 -0.161088262 2.731175026 -2.56266642 0.014396718 0.194277231 -3.908128589 stable

RPS15A 0.028346722 15.60184878 2.562417852 0.014405498 0.194284247 -3.908681772 stable

ITGA5 0.171713297 10.1876545 2.561932612 0.014422652 0.194404125 -3.909761554 stable

SAV1 -0.279636246 9.366149522 -2.561439634 0.014440098 0.19452781 -3.910858407 stable

PIR 0.012511672 2.879031413 2.560652588 0.014467992 0.19479201 -3.912609241 stable

PRX -0.179812983 6.127550864 -2.560231973 0.014482919 0.194881432 -3.913544768 stable

HIST1H2AJ -0.21425522 7.804889579 -2.559721644 0.014501049 0.195013824 -3.914679691 stable

CDC14B -0.557597611 7.848285866 -2.559164401 0.01452087 0.195168784 -3.915918762 stable

CHMP6 -0.132851737 8.632382492 -2.558202769 0.014555132 0.195517565 -3.918056575 stable

STAB2 -0.009267348 2.170967338 -2.557684213 0.014573638 0.195654423 -3.919209147 stable

LINC00473 0.004806871 2.554120333 2.556673162 0.014609783 0.196027789 -3.921455887 stable

NIPA1 0.267050286 5.196469734 2.555875443 0.01463836 0.196291469 -3.923228122 stable

SMIM5 -0.52793892 8.283637869 -2.555636294 0.014646936 0.196291469 -3.923759346 stable

TARDBPP1 /// TARDBPP1 0.007541168 2.81388683 2.555426306 0.014654471 0.196291469 -3.924225765 stable

MAP1B -0.329884231 5.005024635 -2.554370766 0.0146924 0.196687505 -3.926569886 stable

NFATC1 -0.193742771 7.634834415 -2.553950747 0.014707518 0.196777888 -3.927502466 stable

GPX1 -0.197735701 13.94448388 -2.55298864 0.0147422 0.197129787 -3.929638248 stable

RP1-151F17.2 0.24489091 4.205883467 2.552174686 0.014771601 0.197410693 -3.9314447 stable

LOC101928222 0.012025498 2.925669057 2.551549731 0.014794211 0.197530091 -3.932831417 stable

WASL 0.249936185 5.720478243 2.551463554 0.014797331 0.197530091 -3.933022619 stable

ARHGDIB -0.124427824 8.207820172 -2.550369567 0.014836994 0.197947209 -3.935449437 stable

SRM -0.147865079 9.924120277 -2.549707209 0.014861055 0.198155825 -3.936918405 stable

CCDC47 0.179181889 9.894163902 2.549148211 0.014881389 0.198206702 -3.938157932 stable

IGH /// IGHA2 /// IGHD /// IGHG1 -0.327514315 6.824936194 -2.548958369 0.014888301 0.198206702 -3.938578846 stable

SRPR 0.084577722 10.43969483 2.548856617 0.014892007 0.198206702 -3.93880444 stable

TMEM242 -0.217746578 2.89507742 -2.548543288 0.014903423 0.198206702 -3.939499075 stable

GPCPD1 -0.146917955 12.0078948 -2.548445034 0.014907005 0.198206702 -3.939716889 stable

EXTL2 0.362989065 6.221791012 2.547566096 0.014939079 0.198499481 -3.941665081 stable

MAD2L1BP 0.281686719 8.219355167 2.547379329 0.014945903 0.198499481 -3.942078994 stable

TRMT6 -0.125516841 9.013667763 -2.545285469 0.0150226 0.199329335 -3.946717936 stable

EPB41L4A-AS1 -0.187576908 11.45334813 -2.545210962 0.015025335 0.199329335 -3.946882955 stable

LIN52 0.218216954 7.933238602 2.544377388 0.015055974 0.199524343 -3.948728944 stable

LOC441124 -0.234306955 8.246276079 -2.544349479 0.015057001 0.199524343 -3.948790742 stable

LINC01146 0.045176677 3.406770593 2.543955009 0.01507152 0.199537102 -3.949664157 stable

CNOT1 0.099046351 8.486147877 2.543785724 0.015077755 0.199537102 -3.95003895 stable

CWC25 0.135182807 8.673126111 2.543632157 0.015083413 0.199537102 -3.950378928 stable

SPARC -0.456036825 13.10011674 -2.543033688 0.015105482 0.199716725 -3.951703724 stable

BTD 0.235406924 6.352981781 2.540757001 0.015189707 0.200717476 -3.95674147 stable

ZBTB2 0.160595733 9.347883732 2.540413535 0.015202451 0.200773076 -3.957501198 stable

RC3H1 -0.27058665 6.256552731 -2.539807756 0.015224951 0.200887001 -3.958840967 stable

KLRC4 0.524013146 8.839621578 2.539655835 0.015230598 0.200887001 -3.959176928 stable

TMEM33 0.265395332 9.283488852 2.539491767 0.0152367 0.200887001 -3.959539735 stable

FAXDC2 -0.544653945 8.601390416 -2.539239439 0.015246087 0.200892596 -3.960097679 stable

FAH -0.323930924 7.352436036 -2.539021334 0.015254206 0.200892596 -3.960579919 stable

ASCC3 0.129210084 9.447447215 2.538061726 0.015289973 0.201082762 -3.9627013 stable

ETNK1 0.348691577 7.82700232 2.538056815 0.015290156 0.201082762 -3.962712156 stable

GGACT 0.274280063 5.874959595 2.537945964 0.015294293 0.201082762 -3.962957174 stable

LPIN2 0.179281687 10.59820084 2.535827149 0.015373559 0.20199917 -3.967639016 stable

TGDS 0.119787592 8.973704378 2.535624209 0.015381171 0.20199917 -3.968087295 stable

RNF149 0.229005673 12.59556774 2.534337037 0.01542953 0.202521185 -3.970929973 stable

MID1IP1 0.292724091 10.14676182 2.532240922 0.015508578 0.203358637 -3.975556976 stable

NABP1 0.196587507 3.685432378 2.532184321 0.015510718 0.203358637 -3.975681881 stable

NUP88 -0.155117533 10.86865115 -2.531745483 0.015527316 0.203358637 -3.976650219 stable

SETD4 0.230092274 6.25390166 2.531729626 0.015527916 0.203358637 -3.976685205 stable

IQGAP1 0.131343397 13.42481881 2.531035252 0.015554214 0.20357338 -3.978217157 stable

RP1-193H18.2 0.262093579 5.92555883 2.530839802 0.015561623 0.20357338 -3.978648311 stable

ZC3H7A -0.072053639 10.07947252 -2.530061065 0.015591177 0.203846623 -3.980365937 stable

IL12RB1 0.232789522 10.31229447 2.529325607 0.015619136 0.204098716 -3.981987757 stable

PRORSD1P -0.221925767 4.728993638 -2.528734246 0.015641649 0.204240621 -3.983291573 stable

TUBB1 -0.391418992 12.92225497 -2.528584324 0.015647362 0.204240621 -3.983622081 stable

AGAP1 0.946733598 4.678332322 2.528244802 0.015660306 0.204296201 -3.984370521 stable

ZNF628 -0.220976876 6.939479993 -2.527135476 0.015702665 0.204735251 -3.986815406 stable

TNFSF4 -0.365777177 9.262921587 -2.526806524 0.015715247 0.204785769 -3.987540247 stable

NAA40 0.301022943 7.780969653 2.526563184 0.015724559 0.204793665 -3.988076402 stable

BTLA -0.294357688 8.714965911 -2.525727323 0.015756587 0.204964113 -3.989917783 stable

LINC00896 -0.044142382 3.503454726 -2.525686187 0.015758164 0.204964113 -3.990008394 stable

CLDN5 -0.315580472 8.840357921 -2.525539565 0.015763789 0.204964113 -3.99033135 stable

ANKRD39 0.235994801 6.384258666 2.523945794 0.015825048 0.205535236 -3.993840998 stable

BBS1 -0.115652632 8.385588364 -2.523500433 0.015842204 0.205535236 -3.994821446 stable

AATF -0.084481922 9.829821377 -2.523499961 0.015842223 0.205535236 -3.994822484 stable

OSTM1 0.157852095 10.03557355 2.523488417 0.015842668 0.205535236 -3.994847895 stable

RP11-496I2.2 0.012241811 2.829937017 2.522866508 0.015866655 0.205581012 -3.996216797 stable

OSER1 -0.13636583 9.54934885 -2.522609715 0.015876569 0.205581012 -3.99678196 stable

TNFAIP8L2 0.311989862 10.37492577 2.522298427 0.015888595 0.205581012 -3.997467004 stable

ZNF419 0.271077418 6.937000092 2.52229691 0.015888653 0.205581012 -3.997470342 stable

HNRNPK -0.106490185 13.02186247 -2.522264717 0.015889897 0.205581012 -3.997541186 stable

HBA1 /// HBA2 -0.255300602 15.43399752 -2.5208391 0.015945083 0.20606124 -4.000677704 stable

RCOR3 -0.142538271 10.16887455 -2.520629698 0.015953203 0.20606124 -4.001138306 stable

LOC100996782 /// MED18 0.301635102 3.492302701 2.520627266 0.015953298 0.20606124 -4.001143656 stable

GRAMD1A -0.17020963 8.993423235 -2.518353159 0.016041732 0.20708978 -4.006144024 stable

ZNF75A -0.166638682 9.389319916 -2.517572134 0.016072207 0.207288213 -4.007860616 stable

KLHL7 0.153710099 8.041671156 2.517507566 0.016074729 0.207288213 -4.008002512 stable

C12orf73 -0.216178411 6.781256287 -2.516762098 0.01610387 0.20755021 -4.00964057 stable

GINS4 -0.102590966 2.506392796 -2.516326539 0.016120919 0.207570023 -4.010597488 stable

C14orf119 0.239623726 10.51505406 2.516271943 0.016123057 0.207570023 -4.010717426 stable

DDX52 -0.418468539 3.562830327 -2.515950051 0.016135669 0.207618749 -4.011424532 stable

BOD1 0.120653806 9.882844766 2.513410174 0.016235498 0.208778521 -4.017001648 stable

LIMS1 /// LIMS3L -0.377079237 12.0835684 -2.513205774 0.016243557 0.208778521 -4.017450298 stable

HMGXB4 -0.102810177 9.205165989 -2.512904401 0.016255445 0.208817211 -4.018111753 stable

SSBP3 0.192060101 9.302338249 2.51252603 0.016270381 0.208843313 -4.018942124 stable

DBT 0.240498498 6.538905 2.512387842 0.01627584 0.208843313 -4.019245367 stable

BYSL 0.349154141 6.16475653 2.512178447 0.016284114 0.208843313 -4.019704847 stable

ITPR1 -0.135043239 10.56841461 -2.511936984 0.01629366 0.208851862 -4.02023466 stable

C5orf51 0.15823825 9.826546755 2.511643132 0.016305284 0.208887023 -4.020879375 stable

SLC35A2 0.296506151 6.372972809 2.5100133 0.016369893 0.209498788 -4.024454267 stable

PAN2 -0.163302017 9.23841607 -2.509989185 0.01637085 0.209498788 -4.024507149 stable

IFRD2 -0.137527923 8.738859161 -2.509703107 0.016382216 0.209530231 -4.025134458 stable

RP11-65L19.4 -0.080387647 3.581654736 -2.508421631 0.016433214 0.209907427 -4.027943844 stable

PLEC -0.161811542 9.374862489 -2.508416713 0.01643341 0.209907427 -4.027954622 stable

ADI1 -0.200819351 10.81858384 -2.507921937 0.01645314 0.209907427 -4.029039046 stable

THUMPD1 -0.167795306 11.20539631 -2.507891879 0.016454339 0.209907427 -4.029104919 stable

NPR1 -0.135839496 6.742938296 -2.507842031 0.016456328 0.209907427 -4.029214165 stable

CDKN2AIPNL -0.297514405 3.174005583 -2.507129301 0.016484792 0.210045996 -4.030775982 stable

BLOC1S3 0.254498356 8.190825442 2.50712279 0.016485052 0.210045996 -4.030790248 stable

PTCH1 0.310954589 3.460278011 2.50605615 0.016527734 0.210475814 -4.033126993 stable

SNX24 0.199020083 5.08576002 2.502996659 0.01665072 0.211840682 -4.039825622 stable

GSTCD 0.198355472 4.001239031 2.502942029 0.016652924 0.211840682 -4.039945178 stable

KDELC2 0.23686777 7.481703551 2.502638799 0.01666516 0.211881745 -4.040608758 stable

PRR7 -0.290234267 6.225233617 -2.501795725 0.016699223 0.21219647 -4.04245341 stable

WRAP73 0.093640194 8.601317162 2.501579816 0.016707957 0.21219647 -4.042925749 stable

REPIN1 -0.112831238 10.42387599 -2.500862012 0.016737023 0.212337163 -4.04449586 stable

CCNL1 -0.209939342 12.26286579 -2.500860358 0.01673709 0.212337163 -4.044499479 stable

ARRDC4 0.300817724 9.546722155 2.500563123 0.01674914 0.212373962 -4.045149549 stable

IL13 -0.042907294 3.443849571 -2.50034347 0.016758049 0.212373962 -4.045629908 stable

SETD1A -0.154290687 7.53983229 -2.499956168 0.016773769 0.212458712 -4.046476826 stable

ARMCX6 -0.152180431 10.77508283 -2.499599131 0.016788273 0.212527969 -4.047257477 stable

BC043356 0.630174442 4.249150769 2.49871987 0.016824039 0.212777048 -4.049179618 stable

PREX1 0.144000509 11.49618886 2.498565908 0.016830309 0.212777048 -4.049516141 stable

HIAT1 0.11785825 10.77075293 2.498280015 0.016841957 0.212777048 -4.050140996 stable

LIAS -0.1633436 7.938327209 -2.498226628 0.016844134 0.212777048 -4.050257674 stable

RNF146 0.251684588 9.102263652 2.497674352 0.016866659 0.212893945 -4.051464576 stable

ZNF808 0.198442212 5.856893888 2.497470689 0.016874972 0.212893945 -4.051909598 stable

LOC101927451 -0.368442584 6.483424128 -2.497334319 0.016880541 0.212893945 -4.052207563 stable

NEDD1 0.174673716 8.111766631 2.496866398 0.016899662 0.21302087 -4.053229873 stable

VPS4B 0.132754385 11.10947453 2.496474985 0.016915671 0.213108464 -4.054084921 stable

IGH /// IGHA1 /// IGHA2 /// IGHD /// IGHG1 /// IGHG3 /// IGHG4 /// IGHM /// IGHV4-31 -0.506776681 10.15594302 -2.496081102 0.016931796 0.213197411 -4.054945269 stable

GPSM3 -0.087099057 12.32385643 -2.495347032 0.016961884 0.213461994 -4.056548417 stable

RP11-359E8.5 0.00628087 2.380568698 2.494533628 0.01699528 0.213767909 -4.058324425 stable

BRF2 0.376185374 7.105492818 2.49351056 0.017037371 0.214182788 -4.060557631 stable

JUN -0.511372629 9.734784097 -2.491737945 0.017110523 0.214987509 -4.064425422 stable

ABHD15 0.216483834 8.606139754 2.490450982 0.017163813 0.215432226 -4.067232292 stable

SORL1 0.138763874 12.86130409 2.490256242 0.01717189 0.215432226 -4.067656929 stable

MED7 0.187195794 8.658988041 2.490176846 0.017175184 0.215432226 -4.06783005 stable

RP11-152L20.3 -0.526117575 3.135230352 -2.489819723 0.017190007 0.215432226 -4.068608688 stable

RP9 /// RP9P -0.132644976 8.894237544 -2.489778634 0.017191713 0.215432226 -4.068698271 stable

GHR 0.008970545 2.811689127 2.489058847 0.017221628 0.215692179 -4.070267367 stable

DEFA6 0.007983909 2.76121104 2.488456038 0.017246717 0.215891454 -4.071581206 stable

LOC284930 0.005750686 2.492131895 2.487948913 0.01726785 0.215956438 -4.072686322 stable

TMEM14B /// TMEM14C -0.107719937 12.14019464 -2.487890843 0.017270272 0.215956438 -4.072812856 stable

SLC7A6OS 0.157876833 7.741718713 2.487478326 0.017287482 0.216056778 -4.073711669 stable

C1orf64 -0.022082458 2.77921188 -2.486945417 0.017309737 0.216220039 -4.074872639 stable

C19orf60 -0.108968389 8.525705385 -2.486371567 0.017333732 0.216362034 -4.076122601 stable

C5orf24 0.211175342 5.642551767 2.486233676 0.017339502 0.216362034 -4.076422923 stable

ACTR2 0.137036309 12.58566084 2.485790892 0.017358043 0.216478543 -4.077387215 stable

DNER 0.010903281 2.783865622 2.484823872 0.017398598 0.216869329 -4.079492755 stable

LINC01339 -0.128272586 3.463821181 -2.484457473 0.017413987 0.216914966 -4.080290377 stable

ZNF549 0.164691514 3.340579216 2.484297633 0.017420704 0.216914966 -4.080638311 stable

MED28 0.136004094 10.35588646 2.484054004 0.017430946 0.216927667 -4.081168601 stable

CES4A -0.080631292 2.958643832 -2.483676595 0.017446825 0.217010449 -4.081990009 stable

H2AFJ -0.394245862 7.000316928 -2.48331808 0.01746192 0.217083413 -4.082770209 stable

MTHFD1 -0.136249528 8.803664342 -2.482304529 0.01750466 0.217483468 -4.084975461 stable

CRELD1 0.134292269 7.568948124 2.482116663 0.017512593 0.217483468 -4.085384143 stable

WFDC5 0.00646538 2.720204052 2.48170569 0.017529957 0.217584232 -4.08627809 stable

EBAG9 -0.101153793 10.79899698 -2.481266045 0.01754855 0.217700133 -4.087234287 stable

ARHGAP30 0.06276 12.9888287 2.480988256 0.017560308 0.217731153 -4.087838394 stable

RNF169 0.093038918 9.886545709 2.480124673 0.017596905 0.217959917 -4.089716118 stable

GTF2IRD2B -0.190794262 4.663569081 -2.480115565 0.017597291 0.217959917 -4.089735919 stable

LOC100506459 0.24819649 6.061478094 2.479681604 0.017615708 0.218059889 -4.090679317 stable

PLEKHG3 0.197915747 4.570014025 2.479374628 0.017628746 0.218059889 -4.091346587 stable

CYB5B 0.139530218 8.706549043 2.479080149 0.017641262 0.218059889 -4.091986638 stable

EXD2 0.205599349 4.936991539 2.478878164 0.017649852 0.218059889 -4.092425619 stable

C20orf196 0.439329236 4.434402901 2.478834321 0.017651717 0.218059889 -4.092520901 stable

AHNAK 0.132888409 13.36004483 2.478606122 0.017661426 0.218065307 -4.093016818 stable

ACIN1 -0.115883634 9.935823791 -2.478219952 0.017677869 0.218153804 -4.093855959 stable

AMACR /// C1QTNF3-AMACR 0.213488056 5.819627141 2.477801016 0.017695722 0.218259609 -4.094766192 stable

SERPINH1 0.198776817 3.684004681 2.477228809 0.017720133 0.21844615 -4.09600926 stable

LOC100506472 -0.088920068 6.448449294 -2.476203605 0.017763947 0.218769037 -4.098235906 stable

DOLK 0.507998797 6.266778266 2.476180689 0.017764928 0.218769037 -4.09828567 stable

ABLIM3 -0.482296361 8.390864921 -2.475728703 0.017784276 0.218809685 -4.099267123 stable

ILF3-AS1 -0.375217105 8.708619518 -2.475668991 0.017786834 0.218809685 -4.099396773 stable

LOC100505515 -0.019942137 2.254791148 -2.475152896 0.017808953 0.21896727 -4.100517255 stable

NAP1L1 -0.199754493 8.126423367 -2.474765132 0.017825589 0.219057302 -4.101359008 stable

FAM212A -0.193321965 6.514515922 -2.47454351 0.017835103 0.219059771 -4.101840061 stable

FCRLA -0.577402679 9.959653558 -2.472832556 0.017908711 0.219833025 -4.105552802 stable

SERTAD1 0.390976262 8.568433668 2.472646042 0.017916751 0.219833025 -4.105957421 stable

ARHGEF19 -0.205249222 5.820864066 -2.47236311 0.017928955 0.219868066 -4.106571166 stable

EXTL3 0.216705559 5.418182991 2.471985038 0.017945274 0.219953515 -4.107391212 stable

CNTNAP2 -0.452933153 3.344003811 -2.470196897 0.018022641 0.220766981 -4.111268495 stable

VIPR1 -0.256453642 9.423571256 -2.470017631 0.018030414 0.220766981 -4.111657091 stable

N4BP3 -0.213179934 4.191905357 -2.469180779 0.01806674 0.221096669 -4.11347087 stable

CYB5R3 -0.195741581 10.03535538 -2.468842158 0.018081458 0.221161714 -4.114204665 stable

PHF1 -0.215401397 8.644663516 -2.468175136 0.018110482 0.221338076 -4.115649894 stable

C7orf63 -0.018593166 3.16269993 -2.468078327 0.018114697 0.221338076 -4.115859625 stable

ETV3 -0.481505375 6.348024298 -2.467402137 0.018144169 0.221583075 -4.117324388 stable

TSPAN5 0.268338401 7.353106197 2.467042109 0.018159879 0.221618957 -4.118104161 stable

AHCTF1 /// AHCTF1P1 0.254406521 7.920739215 2.466903009 0.018165952 0.221618957 -4.118405412 stable

DMRTC1 /// DMRTC1B -0.367475622 4.900307397 -2.466081313 0.018201863 0.221903619 -4.120184716 stable

IST1 0.266617976 4.357916738 2.465769717 0.018215498 0.221903619 -4.120859336 stable

PRKG2 -0.059990124 3.383877969 -2.465721975 0.018217588 0.221903619 -4.120962695 stable

ALDH3A1 -0.07668014 4.075839198 -2.465225047 0.018239354 0.222053754 -4.122038422 stable

CCDC84 -0.256626777 9.683925374 -2.464794968 0.018258212 0.222135053 -4.12296931 stable

SIPA1 0.122305754 10.70701837 2.464488978 0.018271639 0.222135053 -4.123631542 stable

CDADC1 0.131901732 3.714860774 2.464426887 0.018274365 0.222135053 -4.123765915 stable

SSB -0.119679607 10.63126703 -2.463277396 0.018324893 0.222588291 -4.126253094 stable

EREG 0.268064281 2.836798278 2.463003567 0.018336948 0.222588291 -4.12684546 stable

PMAIP1 -0.256901957 10.58537569 -2.462933341 0.018340041 0.222588291 -4.12699737 stable

STAG1 0.104616854 9.257238195 2.459807552 0.018478189 0.22414929 -4.133755762 stable

LRRC8D -0.106292606 11.69244803 -2.457848961 0.018565232 0.225015826 -4.137987328 stable

GAL3ST1 0.005901071 2.502483101 2.457264041 0.018591299 0.225015826 -4.139250583 stable

SERPINB9P1 -0.407834539 6.722607475 -2.457043318 0.018601144 0.225015826 -4.139727221 stable

PTPMT1 -0.143989484 8.446790195 -2.45699013 0.018603517 0.225015826 -4.139842073 stable

ZNF92 0.177536568 5.173897702 2.456945575 0.018605506 0.225015826 -4.139938282 stable

KCTD9 0.148562645 8.429782341 2.456911579 0.018607023 0.225015826 -4.140011691 stable

GPD1 -0.117573132 3.498021285 -2.455948429 0.018650051 0.225420275 -4.142091114 stable

GGTA1P -0.449473849 10.32982541 -2.455206536 0.018683257 0.225705642 -4.143692446 stable

STRADB -0.295576409 9.250925787 -2.454549808 0.018712695 0.225945231 -4.145109658 stable

NTAN1 -0.099971007 10.28157771 -2.454078621 0.018733843 0.226084515 -4.146126306 stable

LOC101929740 /// ZNF37A 0.175306183 5.548601542 2.453429249 0.018763023 0.226223904 -4.147527177 stable

TBC1D19 0.118405775 2.449384705 2.453156173 0.018775306 0.226223904 -4.148116195 stable

HIST1H2AM -0.515219356 4.27692664 -2.453092513 0.018778171 0.226223904 -4.148253503 stable

ZNF76 -0.151217242 8.466344964 -2.452966004 0.018783865 0.226223904 -4.148526358 stable

REPS2 -0.240226482 6.572005956 -2.449330988 0.018948138 0.228085543 -4.15636201 stable

PFKM -0.176102103 7.854666993 -2.448495888 0.018986061 0.228425142 -4.158160957 stable

MIR6836 /// SNX8 -0.23019059 6.343850478 -2.447415461 0.019035228 0.228899592 -4.160487718 stable

KLHL23 /// PHOSPHO2 /// PHOSPHO2-KLHL23 0.066011244 3.527571923 2.446634286 0.019070849 0.22921075 -4.162169556 stable

MIB1 0.135052557 9.973293284 2.44589645 0.019104549 0.229498517 -4.163757727 stable

ISCA1 -0.101069262 11.0261455 -2.445221696 0.019135415 0.229751966 -4.165209809 stable

SRP19 /// ZRSR1 0.201107203 3.909449196 2.444732782 0.019157809 0.229754986 -4.166261778 stable

CR2 -0.487512058 8.087789493 -2.444567314 0.019165393 0.229754986 -4.166617772 stable

SUPT7L 0.10137287 8.502300158 2.444450339 0.019170756 0.229754986 -4.166869426 stable

DDX20 0.162134994 9.395866737 2.444363484 0.019174739 0.229754986 -4.167056276 stable

SLC15A2 -0.356301296 8.552252615 -2.443692989 0.019205513 0.229923298 -4.168498534 stable

MAPK7 0.221820686 7.956053487 2.443386566 0.019219591 0.229923298 -4.169157565 stable

H19 /// MIR675 0.011398478 2.595404761 2.443171173 0.019229493 0.229923298 -4.169620779 stable

TCF12 0.108351207 9.62014664 2.443125019 0.019231616 0.229923298 -4.169720032 stable

RELA -0.055632815 10.84902276 -2.442993556 0.019237662 0.229923298 -4.170002732 stable

NIF3L1 0.149079658 9.539293745 2.442049123 0.019281151 0.230298744 -4.172033327 stable

CCR5 0.398884403 9.942626854 2.441886279 0.019288658 0.230298744 -4.172383393 stable

KCNAB3 -0.06791859 2.935125655 -2.441058401 0.019326866 0.230637861 -4.174162827 stable

TTC16 0.241685009 6.877433611 2.440243204 0.019364557 0.230923622 -4.175914576 stable

MEST -0.405496049 7.779494804 -2.440115911 0.019370448 0.230923622 -4.176188073 stable

RP3-388M5.9 0.005820076 2.268270463 2.439207364 0.019412544 0.230950731 -4.178139839 stable

TST -0.318550916 8.744881383 -2.439122412 0.019416485 0.230950731 -4.178322308 stable

FAM63A -0.288548227 9.173204458 -2.439103408 0.019417366 0.230950731 -4.178363125 stable

TCTA -0.111666582 9.318252335 -2.439044769 0.019420087 0.230950731 -4.178489073 stable

C3orf62 /// MIR4271 /// USP4 -0.076121515 10.00072718 -2.43900749 0.019421816 0.230950731 -4.178569143 stable

BAZ1B 0.091214001 10.15977112 2.437504105 0.019491687 0.231664458 -4.181797397 stable

TARS 0.114664272 10.06382721 2.436882749 0.019520631 0.231762746 -4.183131223 stable

CLIP2 -0.256025832 7.700701998 -2.436783153 0.019525274 0.231762746 -4.183344997 stable

C19orf26 0.030529463 2.902344479 2.436692163 0.019529517 0.231762746 -4.183540292 stable

RBM14 0.11942603 9.178363736 2.436103122 0.019557003 0.231971895 -4.184804449 stable

ADA 0.287937342 9.950502278 2.435791719 0.019571548 0.232027411 -4.185472668 stable

ZNF550 0.179554381 5.907493753 2.435372184 0.019591159 0.232028071 -4.186372825 stable

CHD9 0.1354907 9.436860496 2.435368469 0.019591333 0.232028071 -4.186380796 stable

SCAF8 0.098734194 11.50007913 2.435058588 0.01960583 0.232082906 -4.187045603 stable

RP11-326I11.3 -0.241149859 5.647396084 -2.434675163 0.019623781 0.232109172 -4.187868102 stable

DCBLD2 0.008233608 2.726392379 2.434444376 0.019634594 0.232109172 -4.188363127 stable

TAGLN2 -0.168158017 13.37415658 -2.434285477 0.019642041 0.232109172 -4.188703938 stable

ZNF395 -0.192744388 9.948245075 -2.434086447 0.019651373 0.232109172 -4.189130797 stable

ATXN2L -0.209394921 8.725502503 -2.433958158 0.01965739 0.232109172 -4.189405924 stable

GLYAT 0.00607978 2.312904285 2.433416746 0.019682803 0.23225115 -4.190566918 stable

HLA-DOA -0.405064691 7.688425518 -2.433061979 0.019699471 0.23225115 -4.191327569 stable

ID2 0.221884967 13.48055774 2.43281382 0.019711137 0.23225115 -4.191859597 stable

PSMD6-AS2 -0.318368749 7.492417004 -2.432672365 0.019717791 0.23225115 -4.192162844 stable

PAPSS1 -0.144582982 10.75795331 -2.43249342 0.01972621 0.23225115 -4.192546444 stable

VCP -0.108949258 9.89559247 -2.432426697 0.01972935 0.23225115 -4.19268947 stable

MGLL -0.438581924 9.425455013 -2.432154571 0.019742162 0.23225115 -4.193272765 stable

CCDC106 -0.110687549 5.961589144 -2.432021958 0.019748408 0.23225115 -4.193557001 stable

LINC00629 0.026732489 3.23231443 2.431437413 0.019775961 0.232458965 -4.194809749 stable

PEG10 -0.36104201 3.327524979 -2.430762417 0.019807822 0.232552614 -4.196256071 stable

IL2RA -0.344490846 7.225925123 -2.430721737 0.019809744 0.232552614 -4.196343227 stable

MEF2C -0.255653107 8.880081091 -2.430435577 0.019823266 0.232552614 -4.19695629 stable

ZNF512 -0.15929399 10.20044346 -2.430431122 0.019823477 0.232552614 -4.196965833 stable

KIAA0226L -0.431276742 9.394892953 -2.429611932 0.019862234 0.232891124 -4.198720549 stable

TBL1XR1 0.150936336 10.59694123 2.428951809 0.019893515 0.232916438 -4.200134227 stable

CENPL 0.365058376 5.264122469 2.428848683 0.019898406 0.232916438 -4.200355049 stable

HNRNPUL2 /// HNRNPUL2-BSCL2 0.137327158 6.173392587 2.428837428 0.01989894 0.232916438 -4.200379149 stable

ZNF189 0.176620796 9.074382143 2.428730703 0.019904002 0.232916438 -4.200607671 stable

PRKAR2B -0.351378227 12.75428421 -2.428054772 0.019936094 0.233175971 -4.202054814 stable

HLCS 0.236231948 3.946146891 2.427342037 0.019969985 0.233366057 -4.203580432 stable

IGHA1 /// IGHA2 /// IGHG1 /// IGHG3 /// IGHM /// IGHV3-23 /// IGHV4-31 -0.471516986 12.18549194 -2.427199883 0.01997675 0.233366057 -4.203884676 stable

ALOX12 -0.479646933 9.797948874 -2.427087273 0.019982111 0.233366057 -4.204125679 stable

BAG6 -0.06343157 11.81255193 -2.42666765 0.0200021 0.233483565 -4.205023664 stable

METTL22 -0.220265669 8.394492523 -2.426192299 0.020024764 0.233493081 -4.206040769 stable

FBRSL1 -0.112040121 9.865819006 -2.426101551 0.020029094 0.233493081 -4.206234926 stable

BTN3A1 0.167673096 10.54443048 2.426026066 0.020032696 0.233493081 -4.206396423 stable

ENO1-AS1 -0.078322196 2.547120174 -2.425619567 0.020052103 0.233533675 -4.207266046 stable

CD40 -0.363787639 8.397359688 -2.425537233 0.020056036 0.233533675 -4.207442172 stable

ANAPC5 -0.102244152 12.13330389 -2.424855166 0.020088644 0.233718716 -4.20890104 stable

MGARP 0.008293498 2.514140477 2.424789186 0.020091801 0.233718716 -4.209042149 stable

NRGN -0.302091409 14.55689319 -2.424143844 0.020122701 0.233802783 -4.210422165 stable

TMEM102 0.098884813 5.391977125 2.424069786 0.02012625 0.233802783 -4.210580514 stable

NCKIPSD -0.173706846 4.875248647 -2.423832164 0.020137641 0.233802783 -4.211088572 stable

ACTR3 0.109394775 13.5096697 2.423601133 0.020148721 0.233802783 -4.211582501 stable

LOC100507291 -0.111062807 2.654015021 -2.423600983 0.020148728 0.233802783 -4.211582823 stable

DPH7 -0.09677448 8.39892758 -2.423269618 0.02016463 0.233871929 -4.212291199 stable

RPL9 0.036646106 15.54404683 2.422333943 0.020209594 0.233892564 -4.214291061 stable

GEMIN5 0.194591866 8.102131358 2.422308216 0.020210832 0.233892564 -4.214346041 stable

PPIEL -0.087846978 5.786354034 -2.422234334 0.020214386 0.233892564 -4.214503926 stable

CXorf58 0.005282931 2.306764893 2.422207621 0.020215672 0.233892564 -4.214561011 stable

ACBD4 -0.19053786 7.856513555 -2.422198106 0.02021613 0.233892564 -4.214581345 stable

CDH4 0.006997271 2.220508482 2.421806593 0.020234976 0.233995513 -4.21541794 stable

CNKSR2 -0.30114284 3.760879581 -2.42153297 0.020248157 0.233996576 -4.216002567 stable

HOXB4 0.159831687 6.321272607 2.421391716 0.020254965 0.233996576 -4.216304352 stable

BAALC 0.005400769 2.375311681 2.420939979 0.02027675 0.234133252 -4.217269392 stable

GGPS1 0.36296139 6.473940455 2.420454327 0.020300194 0.234218707 -4.218306737 stable

ZBTB33 0.091847364 5.526014427 2.420374162 0.020304066 0.234218707 -4.218477955 stable

MZB1 -0.483986339 8.644172314 -2.420131833 0.020315776 0.234224751 -4.218995495 stable

FOXR2 0.010451787 2.918514817 2.419719515 0.020335713 0.234224751 -4.219875993 stable

RBM34 -0.035632871 2.324241744 -2.419629331 0.020340076 0.234224751 -4.220068564 stable

TRMT5 -0.063125357 12.79295819 -2.419329245 0.0203546 0.234224751 -4.220709306 stable

P2RY11 /// PPAN-P2RY11 0.22454256 5.452089016 2.419186016 0.020361536 0.234224751 -4.221015108 stable

SSTR1 0.007319474 2.329505121 2.419128148 0.020364339 0.234224751 -4.221138655 stable

GRAP2 -0.323115939 10.56165709 -2.41841297 0.020399007 0.234345022 -4.222665369 stable

AP5S1 -0.151179368 7.647007485 -2.418391919 0.020400028 0.234345022 -4.222710301 stable

C15orf45 0.00657484 2.857408434 2.418250192 0.020406905 0.234345022 -4.223012811 stable

SGPP1 0.201869019 10.2595786 2.417962112 0.02042089 0.234345022 -4.223627661 stable

CHMP2B 0.190414387 10.31052351 2.417825771 0.020427511 0.234345022 -4.223918634 stable

CCDC71 -0.120623993 7.255576772 -2.417680364 0.020434575 0.234345022 -4.224228943 stable

IPO4 -0.148457532 7.995142143 -2.417245151 0.020455731 0.234450855 -4.225157639 stable

AL833181 -0.38250582 8.869735795 -2.417080531 0.020463739 0.234450855 -4.225508887 stable

PTOV1 -0.125281605 8.932686807 -2.415861512 0.020523124 0.234961234 -4.228109349 stable

LOC100507616 -0.456800884 4.931371654 -2.415756118 0.020528266 0.234961234 -4.228334136 stable

SPRN -0.063860163 3.416363491 -2.415220155 0.020554431 0.235146286 -4.229477133 stable

TNFRSF13B -0.323158055 7.292933968 -2.414686936 0.020580492 0.235246978 -4.230614092 stable

RRN3P3 0.034934094 2.484477801 2.41439442 0.020594801 0.235246978 -4.231237733 stable

RHOQ -0.264702465 8.829099797 -2.414356517 0.020596656 0.235246978 -4.231318539 stable

ELOF1 -0.102886057 9.129453185 -2.414179137 0.020605338 0.235246978 -4.23169668 stable

C17orf89 -0.034259098 3.563011118 -2.414017753 0.02061324 0.235246978 -4.232040702 stable

RPS3 0.06277838 15.66681327 2.412940351 0.020666065 0.23567184 -4.234336967 stable

DPH5 -0.146339667 10.03519076 -2.412759638 0.020674938 0.23567184 -4.234722049 stable

RBM39 0.110946245 12.91852762 2.412645832 0.020680527 0.23567184 -4.234964546 stable

NEGR1 -0.106241587 3.012323524 -2.409821191 0.020819691 0.237142828 -4.240980579 stable

KLHL36 -0.156813953 8.265609737 -2.40854814 0.020882688 0.237745253 -4.243690278 stable

PDHA1 -0.07368271 9.22867835 -2.408271261 0.020896412 0.237786406 -4.244279476 stable

PNO1 -0.118552218 9.267394321 -2.407678507 0.020925821 0.238005911 -4.245540691 stable

CRIM1 /// LOC101929500 0.238578891 5.430173493 2.406595546 0.020979647 0.238502794 -4.247844336 stable

ZNF816 /// ZNF816-ZNF321P 0.125815064 4.467620923 2.406091793 0.021004728 0.238672564 -4.248915647 stable

LOC101929747 -0.220921973 7.165122286 -2.405548425 0.021031812 0.238860948 -4.25007102 stable

PMF1 -0.114791496 9.685017793 -2.405184386 0.021049974 0.238860948 -4.250844975 stable

LBX1 0.006462103 2.602452043 2.404834645 0.021067437 0.238860948 -4.251588449 stable

UAP1 0.27704338 9.57670224 2.404795183 0.021069408 0.238860948 -4.251672332 stable

CLK1 0.222234727 11.88712114 2.404669648 0.02107568 0.238860948 -4.25193917 stable

OSBPL6 0.016538087 2.727649065 2.404352436 0.021091536 0.238860948 -4.252613394 stable

SH3BGRL2 -0.513324387 10.46113747 -2.404335282 0.021092394 0.238860948 -4.25264985 stable

JMY 0.163342861 9.42357871 2.403429857 0.021137712 0.239258958 -4.254573927 stable

SCP2 0.077529464 11.83129892 2.402291642 0.021194806 0.239777569 -4.256991938 stable

GATAD2A 0.160743053 10.3041502 2.402110259 0.021203917 0.239777569 -4.257377187 stable

FAM175A -0.132909831 9.225904385 -2.401428019 0.02123822 0.240050057 -4.258826043 stable

MSL3 -0.098064535 11.51605316 -2.401172102 0.0212511 0.240080271 -4.259369449 stable

MIR424 /// MIR503HG -0.035233417 3.089781354 -2.400118291 0.021304212 0.240564754 -4.261606628 stable

WBP2 -0.173815989 11.06726831 -2.399502122 0.021335323 0.24080045 -4.262914384 stable

HNRNPF 0.086535279 11.33961602 2.399165294 0.021352347 0.240877007 -4.263629162 stable

PRUNE -0.204476948 11.46614101 -2.398708598 0.021375449 0.241022025 -4.264598192 stable

LOC101928589 /// TMEM164 -0.140452988 10.62112951 -2.397944463 0.021414154 0.241342143 -4.266219248 stable

C2orf49 0.201549397 7.882398267 2.397569831 0.021433153 0.241342143 -4.267013863 stable

AOAH 0.237739361 10.86332804 2.397540873 0.021434622 0.241342143 -4.267075281 stable

VIT 0.573123962 2.691677672 2.397013723 0.021461383 0.241527837 -4.26819323 stable

PAFAH1B1 0.113248697 10.44453806 2.396663954 0.021479155 0.24161225 -4.268934899 stable

LOC692247 -0.176678741 5.726812157 -2.396382278 0.021493478 0.241657788 -4.269532124 stable

C1orf53 0.018318963 2.216100855 2.395717656 0.021527306 0.241834743 -4.270941082 stable

NAP1L5 0.326234553 6.510212218 2.395593586 0.021533627 0.241834743 -4.271204071 stable

CHST7 0.346189739 7.665404965 2.395467304 0.021540061 0.241834743 -4.271471738 stable

C17orf105 0.010622787 3.210499025 2.395052156 0.021561228 0.241900298 -4.272351612 stable

AK096159 0.0185424 2.879129818 2.394949407 0.021566469 0.241900298 -4.272569364 stable

IGHM -0.408827631 14.23559726 -2.3941854 0.02160548 0.242184467 -4.274188271 stable

IL17C -0.123734921 7.85892159 -2.394038129 0.021613007 0.242184467 -4.27450029 stable

RAMP1 0.236644123 7.149988281 2.393848671 0.021622694 0.242184467 -4.274901669 stable

ATP2A2 0.105847324 11.05867465 2.393195002 0.021656145 0.242388368 -4.276286326 stable

PPP2R1B 0.151059137 8.850457002 2.392950729 0.021668658 0.242388368 -4.276803695 stable

LAT2 -0.045808154 3.392990208 -2.392889134 0.021671814 0.242388368 -4.276934146 stable

RNPC3 -0.207495203 9.921135855 -2.392687474 0.02168215 0.242388714 -4.277361222 stable

PEF1 0.118375485 10.57396829 2.391399036 0.021748295 0.242950487 -4.280089255 stable

LYRM1 -0.112435163 10.14305346 -2.391196129 0.021758728 0.242950487 -4.280518775 stable

SOS1 0.021662612 2.955481483 2.3908321 0.021777457 0.242950487 -4.281289294 stable

ADCY7 0.253948588 8.391749529 2.390777119 0.021780287 0.242950487 -4.281405662 stable

ERP44 -0.138950271 8.104875032 -2.390512272 0.021793924 0.242950487 -4.281966186 stable

TMEM40 -0.338910329 7.108355636 -2.390476594 0.021795762 0.242950487 -4.282041691 stable

EIF5A2 0.204044884 5.194247627 2.390302996 0.021804705 0.242950487 -4.282409064 stable

GABPB1 0.142262067 9.438314777 2.389559938 0.021843024 0.243211091 -4.283981328 stable

TMED8 -0.125494564 10.71538445 -2.389448526 0.021848775 0.243211091 -4.284217036 stable

LZTS2 -0.15977215 6.771655114 -2.38902214 0.021870796 0.243334166 -4.285119046 stable

CREB3L1 -0.081907471 2.761924088 -2.388833941 0.021880522 0.243334166 -4.285517139 stable

ZNF354C 0.151228793 7.781703027 2.388494276 0.021898086 0.243414403 -4.286235566 stable

LOC100652930 /// NPEPL1 /// STX16-NPEPL1 -0.139586253 9.460184397 -2.387439784 0.021952694 0.243757807 -4.288465444 stable

ABT1 0.111774576 9.623357286 2.387366351 0.021956501 0.243757807 -4.288620701 stable

TRAPPC9 -0.189807834 6.464618717 -2.387297538 0.021960069 0.243757807 -4.288766187 stable

C14orf159 -0.123930567 6.576109191 -2.386856438 0.021982956 0.243896745 -4.289698702 stable

ATP13A3 0.118905869 8.753204323 2.3861218 0.02202112 0.244204977 -4.291251488 stable

EXOSC5 -0.143225252 8.495501859 -2.38532497 0.022062582 0.24446338 -4.292935329 stable

MCL1 0.241158148 12.08493149 2.385138977 0.022072271 0.24446338 -4.293328305 stable

APBB3 /// MIR6831 -0.189410197 7.809028671 -2.384996382 0.022079701 0.24446338 -4.293629572 stable

ANKFY1 0.008110469 2.029969158 2.384867755 0.022086405 0.24446338 -4.293901317 stable

SEC24B-AS1 -0.1266468 4.292517946 -2.38467629 0.022096388 0.24446338 -4.294305795 stable

TIGD2 0.284954696 7.351902081 2.382878044 0.022190348 0.245358817 -4.298103506 stable

TGFBR2 0.178739332 10.65258766 2.382728326 0.022198187 0.245358817 -4.298419598 stable

FZD3 0.244576407 3.644005718 2.382478409 0.022211278 0.245388201 -4.298947206 stable

KLRC3 0.614815384 9.559314403 2.381618862 0.022256356 0.245770778 -4.300761503 stable

ADAM10 0.097469939 12.73773777 2.380970764 0.0222904 0.246031202 -4.302129162 stable

CHST14 0.236440981 8.392552519 2.379985654 0.022342236 0.246487676 -4.304207477 stable

RNF141 -0.319474527 5.600997384 -2.379480252 0.022368872 0.246651286 -4.30527349 stable

KNTC1 -0.140950249 7.68293168 -2.379114202 0.022388182 0.246651286 -4.306045473 stable

ASS1 0.232579421 4.266286673 2.378892166 0.022399902 0.246651286 -4.306513694 stable

MIDN 0.284833745 9.10011455 2.378771021 0.022406299 0.246651286 -4.306769145 stable

ZMAT1 -0.238188374 8.840926764 -2.378710455 0.022409498 0.246651286 -4.306896853 stable

DMTN -0.447002473 9.102586619 -2.377445573 0.022476396 0.247271898 -4.309563414 stable

SNRNP48 0.17574724 8.330350239 2.377192889 0.022489782 0.24730349 -4.310095983 stable

RP11-97C16.1 -0.19035654 8.82492412 -2.375990356 0.022553585 0.247889188 -4.312629927 stable

SLC25A34 -0.175430872 4.871968241 -2.374228992 0.022647332 0.248754986 -4.316339703 stable

DLEU2 0.278641653 7.649639787 2.374113166 0.022653509 0.248754986 -4.316583583 stable

SOX12 -0.215280601 6.636536428 -2.373393476 0.022691924 0.249060547 -4.318098748 stable

LSM11 0.37525441 3.918355502 2.372463591 0.022741647 0.249290132 -4.320055929 stable

CBX8 0.255044307 4.999148839 2.372240376 0.022753597 0.249290132 -4.320525658 stable

C1QTNF5 /// MFRP -0.054042765 3.133093464 -2.372199146 0.022755805 0.249290132 -4.320612418 stable

NOL12 -0.126228307 9.491022568 -2.371928536 0.022770301 0.249290132 -4.321181832 stable

MORF4L2-AS1 -0.142122346 3.814039555 -2.371815246 0.022776373 0.249290132 -4.3214202 stable

WDFY4 -0.14886719 3.364993889 -2.371814112 0.022776434 0.249290132 -4.321422587 stable

NUDCD1 0.069304403 3.788367862 2.371173949 0.022810769 0.249549808 -4.322769369 stable

NDUFA4 -0.063596846 12.37291478 -2.369893552 0.022879582 0.250186266 -4.32546227 stable

MYL12A -0.303431256 8.266880576 -2.368723799 0.022942614 0.250680058 -4.327921523 stable

PLA2G12A -0.354608335 8.49101979 -2.368660013 0.022946055 0.250680058 -4.328055599 stable

LOC100288893 0.294333858 5.720603162 2.367770159 0.022994116 0.251006748 -4.329925758 stable

ARPC5L 0.164909644 11.10330116 2.367711219 0.022997302 0.251006748 -4.330049609 stable

OPTN 0.272826594 9.324483104 2.366833287 0.023044814 0.251334222 -4.331894167 stable

NPIPA1 -0.209499536 11.98440692 -2.366761981 0.023048676 0.251334222 -4.33204396 stable

C10orf88 0.185188765 6.6791523 2.366406477 0.023067943 0.251425552 -4.332790721 stable

PIK3CD 0.123373218 12.9330117 2.36584548 0.023098377 0.251425552 -4.333968963 stable

KBTBD7 0.408458422 7.862482204 2.365796008 0.023101063 0.251425552 -4.334072859 stable

CAMK1D -0.167718465 7.301426092 -2.365657853 0.023108564 0.251425552 -4.334362984 stable

LINC00341 /// SYNE3 -0.174174902 11.44715507 -2.365622219 0.023110499 0.251425552 -4.334437812 stable

DUSP14 0.297881542 5.612914744 2.365131516 0.023137162 0.251599249 -4.335468178 stable

AC007365.3 -0.187648852 6.137321655 -2.364669771 0.023162276 0.251755958 -4.336437591 stable

CSNK1E /// CSNK1E -0.095715664 7.70964411 -2.36315218 0.023244992 0.25245797 -4.339622713 stable

LA16c-380H5.4 -0.038867957 2.618942978 -2.363091035 0.02324833 0.25245797 -4.339751012 stable

RP13-30A9.2 0.005485001 2.555473334 2.362686883 0.023270406 0.252581084 -4.340598974 stable

RALGAPB 0.089045927 10.00854882 2.361805952 0.02331859 0.25298734 -4.342446903 stable

TBC1D30 0.096324891 2.974105093 2.361532696 0.023333554 0.25303298 -4.343020007 stable

RP11-119F7.5 0.145292733 3.643871151 2.359518202 0.023444144 0.25411507 -4.347243516 stable

TMPO 0.160669333 9.763789054 2.358759034 0.023485942 0.25445087 -4.348834459 stable

CSTF2T 0.208166268 8.824886109 2.357996718 0.023527981 0.254710106 -4.350431617 stable

ZNF621 0.136880665 8.4492301 2.357932463 0.023531528 0.254710106 -4.350566221 stable

SNX6 -0.088816021 10.68284635 -2.357155317 0.02357446 0.255036054 -4.35219402 stable

CRY1 0.192169378 9.877973335 2.356994988 0.023583326 0.255036054 -4.352529792 stable

RBM23 0.112232989 10.58552694 2.356529111 0.023609106 0.255117702 -4.35350537 stable

UHMK1 0.167207497 9.096437387 2.35646656 0.023612569 0.255117702 -4.353636344 stable

USF2 -0.173964244 10.28497833 -2.356246453 0.023624759 0.255132214 -4.354097203 stable

MICAL1 -0.121372785 10.78358771 -2.355764867 0.023651451 0.255214218 -4.355105435 stable

C16orf52 /// LOC101930115 /// VWA3A 0.08271034 7.27821187 2.355579201 0.023661748 0.255214218 -4.355494097 stable

AP4B1 0.102087451 9.817134716 2.355522315 0.023664904 0.255214218 -4.355613175 stable

SREBF2 -0.114788447 9.057893599 -2.354769517 0.023706703 0.255547829 -4.357188777 stable

CTDSPL -0.529980355 9.229334198 -2.354381597 0.023728268 0.255663123 -4.358000545 stable

ZNF397 0.180819165 8.491415649 2.353865852 0.023756967 0.255855136 -4.359079646 stable

C11orf84 0.16526413 6.08698764 2.35214844 0.023852758 0.256696532 -4.362671738 stable

ANXA6 0.166303845 12.09056711 2.35207395 0.02385692 0.256696532 -4.362827495 stable

SYTL3 0.523366943 4.926595205 2.351740653 0.023875553 0.256779557 -4.363524367 stable

ADIRF-AS1 -0.035223919 2.288849238 -2.351103315 0.023911221 0.257045624 -4.364856736 stable

NAT8 /// NAT8B -0.481211676 5.67873429 -2.350898198 0.02392271 0.257051651 -4.365285481 stable

GNAZ -0.442612453 9.494676073 -2.35052484 0.023943636 0.257159021 -4.366065817 stable

CFLAR 0.16167629 10.75439651 2.350139522 0.023965249 0.257273674 -4.366871052 stable

C6orf203 0.185921967 8.211911225 2.34954885 0.023998415 0.25751219 -4.368105245 stable

HDGFRP2 -0.134615982 7.96271621 -2.348053323 0.024082574 0.258297406 -4.371229069 stable

ELF4 0.175853586 9.58136572 2.347660592 0.024104718 0.258417078 -4.37204915 stable

EXOC3L2 -0.164332859 6.046818406 -2.34692113 0.024146463 0.258746673 -4.37359298 stable

LIMA1 0.243467801 7.335843021 2.344365936 0.024291212 0.259963915 -4.378924844 stable

SYNE1 0.230295976 11.13807856 2.344197273 0.024300795 0.259963915 -4.379276637 stable

LINS 0.220538183 9.005958062 2.344159079 0.024302965 0.259963915 -4.379356298 stable

LIMS1 /// LIMS3 /// LIMS3L -0.28360425 12.32844759 -2.344136177 0.024304266 0.259963915 -4.379404064 stable

DNPH1 -0.185960855 8.160997574 -2.343572501 0.024336319 0.260188433 -4.380579602 stable

ARMC6 -0.120289972 8.551949707 -2.34332677 0.024350304 0.260204951 -4.381092004 stable

ERN1 0.362918938 6.572362242 2.343055752 0.024365736 0.260204951 -4.381657088 stable

PTDSS1 -0.094821572 11.78411432 -2.342962437 0.024371052 0.260204951 -4.381851643 stable

AUH 0.133271726 9.305662707 2.342157806 0.02441693 0.260576505 -4.383528994 stable

HSH2D 0.277729511 9.264268863 2.341428045 0.024458607 0.260902902 -4.385049897 stable

RBM41 -0.208896206 6.549251513 -2.341095251 0.024477634 0.260987506 -4.385743358 stable

DENND5B -0.235090964 4.62361614 -2.340326417 0.024521643 0.261239579 -4.38734514 stable

CDK5RAP1 -0.084824902 9.347474238 -2.340294185 0.024523489 0.261239579 -4.387412282 stable

ZNF234 0.073616794 3.884026375 2.339590822 0.024563815 0.2615507 -4.3888773 stable

SLC35G1 -0.0943224 3.845827851 -2.338522099 0.024625203 0.261871444 -4.391102687 stable

PPP1R35 0.112625091 9.992423536 2.338354666 0.024634832 0.261871444 -4.391451262 stable

SIN3A 0.130571422 9.291189342 2.337973046 0.024656794 0.261871444 -4.392245674 stable

HRK -0.158513505 3.399508731 -2.337951701 0.024658023 0.261871444 -4.392290105 stable

METTL14 0.11483058 9.329270395 2.337950157 0.024658112 0.261871444 -4.39229332 stable

ZBTB1 0.164754349 4.230154182 2.337885333 0.024661844 0.261871444 -4.392428253 stable

YBX3 -0.250372614 12.37362319 -2.337711198 0.024671873 0.261871444 -4.392790705 stable

COG6 0.137135314 8.782114625 2.337430634 0.024688039 0.261874515 -4.393374641 stable

SHARPIN -0.115507642 8.351539943 -2.337319768 0.024694429 0.261874515 -4.393605372 stable

SH3GLB1 0.082204332 10.57131616 2.336895497 0.0247189 0.261939806 -4.394488273 stable

TXNDC9 0.129212705 4.454625343 2.336575143 0.024737391 0.261939806 -4.395154844 stable

C2CD5 0.107406065 11.16108951 2.336534479 0.024739739 0.261939806 -4.395239451 stable

DAP -0.223813661 9.741628722 -2.336441089 0.024745132 0.261939806 -4.395433754 stable

LGALSL -0.446889838 7.58963353 -2.335665876 0.024789942 0.262296098 -4.397046414 stable

LINC00294 0.194279718 8.145276196 2.33512474 0.024821265 0.262509428 -4.398171891 stable

BTN3A3 0.147823541 11.60973856 2.334692031 0.024846337 0.262539995 -4.399071715 stable

IGH /// IGHA1 /// IGHA2 /// IGHD /// IGHG1 /// IGHG2 /// IGHG3 /// IGHM /// IGHV4-31 -0.399096465 8.013876824 -2.334689586 0.024846479 0.262539995 -4.3990768 stable

LOC100287590 -0.142449904 4.82786835 -2.334440855 0.024860901 0.262574432 -4.399593983 stable

NHLRC2 0.106789427 7.070905286 2.333715227 0.024903019 0.262901221 -4.401102534 stable

TRA2B -0.074573728 12.11504177 -2.333204971 0.024932675 0.263040658 -4.402163124 stable

CTB-119C2.1 -0.095559327 3.114153929 -2.333100309 0.024938762 0.263040658 -4.402380647 stable

LAMB2P1 -0.194515488 4.812807117 -2.332720445 0.024960865 0.263040658 -4.403170072 stable

PRMT6 0.327780291 7.97893603 2.332372093 0.024981149 0.263040658 -4.403893924 stable

HTATSF1 0.126125587 7.561450597 2.331867579 0.025010554 0.263040658 -4.404942128 stable

LOC285628 /// MIR146A 0.303501443 6.400736969 2.331853671 0.025011365 0.263040658 -4.404971022 stable

DGCR6L 0.004938728 2.42079468 2.331804306 0.025014244 0.263040658 -4.405073575 stable

NMD3 -0.058056123 9.390262236 -2.331762064 0.025016707 0.263040658 -4.405161331 stable

SLC2A12 0.012958747 2.611717023 2.331759165 0.025016877 0.263040658 -4.405167352 stable

RP11-457I16.2 0.016359478 2.867727754 2.331232824 0.025047593 0.263245952 -4.406260685 stable

FAM24B -0.269445417 7.216991213 -2.330308662 0.025101608 0.263695817 -4.408179935 stable

LOC100130429 -0.203577487 6.477045221 -2.330041436 0.025117247 0.263735547 -4.408734788 stable

AXIN1 0.149966483 8.672389859 2.329860915 0.025127816 0.263735547 -4.409109587 stable

NSMCE4A -0.073254625 10.39270364 -2.329382374 0.025155853 0.263904308 -4.410103027 stable

MGC12488 -0.165158972 3.950835928 -2.329203592 0.025166335 0.263904308 -4.410474133 stable

RAP2A 0.258111371 2.604298759 2.328599384 0.025201788 0.264158315 -4.411728165 stable

FBXL4 0.105074082 8.543680756 2.328152412 0.025228044 0.264315735 -4.412655695 stable

PODNL1 -0.058630026 4.839267495 -2.327419566 0.025271146 0.264600563 -4.414176166 stable

ZNF57 0.505604518 5.068469652 2.327251337 0.02528105 0.264600563 -4.414525149 stable

SENP5 0.118919786 8.677427149 2.327116696 0.025288979 0.264600563 -4.41480444 stable

IL2RG 0.167043247 13.61346601 2.32634295 0.025334587 0.264855422 -4.416409219 stable

AIP 0.103351119 10.14207278 2.326321415 0.025335857 0.264855422 -4.416453879 stable

RFC1 0.144976562 8.709251473 2.326014438 0.025353973 0.264926314 -4.417090444 stable

ZNF689 0.258146836 8.675329382 2.325824883 0.025365166 0.264926314 -4.417483486 stable

CPD 0.239190795 9.639780208 2.325416144 0.025389315 0.265060838 -4.418330923 stable

HINFP 0.173017427 7.411268477 2.325059807 0.025410384 0.265163111 -4.419069623 stable

RAB29 0.179916488 11.66123847 2.324547952 0.025440677 0.265361497 -4.42013057 stable

MICB 0.261629745 10.0817749 2.324249001 0.025458385 0.265383195 -4.420750139 stable

FGF2 -0.050805403 3.175226331 -2.324131919 0.025465323 0.265383195 -4.420992771 stable

ZNF136 0.136491719 8.844642526 2.323536828 0.025500614 0.265633279 -4.422225857 stable

CT45A1 /// CT45A2 /// CT45A3 /// CT45A4 /// CT45A5 /// CT45A6 /// LOC101060211 /// LOC102723631 /// LOC102723680 /// LOC102723737 0.014923572 3.058090687 2.322607833 0.025555794 0.266090232 -4.424150351 stable

IGHA1 /// IGHA2 /// IGHD /// IGHG1 /// IGHG3 /// IGHG4 /// IGHM /// IGHV4-31 -0.58753616 8.901899249 -2.322111955 0.025585292 0.266279493 -4.425177367 stable

CROT 0.163051353 8.198407774 2.321697599 0.025609964 0.266418383 -4.426035417 stable

AKAP2 /// PALM2-AKAP2 -0.312179843 9.685329747 -2.321403257 0.025627503 0.266482978 -4.42664487 stable

OXCT2 -0.087458256 4.150867344 -2.320929041 0.025655783 0.266614618 -4.427626642 stable

DOCK11 0.09054396 12.34779866 2.320810889 0.025662833 0.266614618 -4.427871229 stable

ZNF598 -0.116349045 8.79281528 -2.320101987 0.025705171 0.266893796 -4.429338535 stable

RIOK3 -0.168125378 11.1088603 -2.319981064 0.025712399 0.266893796 -4.429588792 stable

IGHA1 /// IGHG1 /// IGHM -0.354108822 8.597738579 -2.319700632 0.025729169 0.26695006 -4.430169122 stable

FXYD2 /// FXYD6-FXYD2 -0.193558931 5.278968617 -2.318903604 0.025776885 0.267234557 -4.431818215 stable

RNASEH1 -0.148435051 8.850283804 -2.318863094 0.025779312 0.267234557 -4.431902022 stable

EMC2 0.179535665 9.040627656 2.317827849 0.025841414 0.267760308 -4.434043332 stable

TRBV27 -0.551849571 10.50568909 -2.31686264 0.025899435 0.268037654 -4.43603913 stable

ZKSCAN7 0.432865535 5.582747904 2.316827592 0.025901544 0.268037654 -4.436111589 stable

GLP1R -0.131065734 3.146896506 -2.316628835 0.025913507 0.268037654 -4.436522484 stable

ACKR1 /// CADM3 0.006355061 2.722488881 2.316372481 0.025928945 0.268037654 -4.43705241 stable

PREPL 0.142748512 10.40683593 2.316256812 0.025935913 0.268037654 -4.437291503 stable

TTLL12 -0.145111995 8.69653151 -2.316088551 0.025946052 0.268037654 -4.437639291 stable

DDX6 0.574485463 8.605946896 2.316057061 0.02594795 0.268037654 -4.437704375 stable

LOC102724364 /// SEC22B 0.111346144 9.582008855 2.315358199 0.025990105 0.268355245 -4.43914867 stable

PARS2 0.408107389 6.17137362 2.314841434 0.026021314 0.268484485 -4.440216422 stable

ZNF597 0.286721266 4.396279552 2.314631008 0.026034033 0.268484485 -4.440651159 stable

LYSMD2 0.196038121 11.94686903 2.314584155 0.026036865 0.268484485 -4.440747952 stable

LOC101929760 -0.465408455 5.866989014 -2.313211045 0.026120003 0.269115093 -4.443583997 stable

C15orf61 0.10158037 9.679561911 2.313110761 0.026126084 0.269115093 -4.443791075 stable

CAMK2D -0.151130258 9.692095077 -2.312909077 0.026138318 0.269115093 -4.444207516 stable

RAB3GAP1 -0.140255904 8.548580955 -2.312777104 0.026146326 0.269115093 -4.444480002 stable

NAA35 0.148988574 7.434926864 2.312630447 0.026155227 0.269115093 -4.444782791 stable

MST4 0.17008002 11.94137515 2.312296299 0.026175519 0.269144904 -4.445472624 stable

ATP6V1D 0.181817032 10.28572095 2.312205923 0.02618101 0.269144904 -4.445659187 stable

FAM81B -0.856748344 6.199635423 -2.310482613 0.026285907 0.270063985 -4.449215575 stable

IFIT5 0.368417558 9.345176869 2.310360132 0.026293377 0.270063985 -4.449468263 stable

VWA8 -0.199662526 9.30021723 -2.309632894 0.026337768 0.270178555 -4.450968401 stable

C16orf54 0.265930145 11.74555234 2.30957614 0.026341235 0.270178555 -4.451085457 stable

AK091028 /// GMDS-AS1 0.051309784 3.882473165 2.309465634 0.026347988 0.270178555 -4.451313372 stable

PLOD2 -0.379220658 4.58636973 -2.309424872 0.026350479 0.270178555 -4.451397441 stable

CD200R1 0.250412162 6.544110908 2.307826208 0.026448341 0.271063805 -4.454693666 stable

LOC101060321 /// LOC101060351 /// LOC101060376 /// LOC101060389 /// LOC102723859 /// LOC102724862 /// TBC1D3 /// TBC1D3C /// TBC1D3F /// TBC1D3H -0.161164084 9.810379922 -2.30723071 0.026484878 0.271203011 -4.45592106 stable

RHPN1 -0.146156873 7.235361339 -2.307209091 0.026486205 0.271203011 -4.455965615 stable

MORC2-AS1 -0.181232712 8.301830649 -2.306897327 0.026505353 0.271203011 -4.456608098 stable

BC042022 /// LOC100506331 0.023733296 3.112015519 2.306853506 0.026508045 0.271203011 -4.456698401 stable

IGHD3-16 /// IGHD3-16 /// IGHG1 /// SKAP2 -0.330733748 5.407841695 -2.306148122 0.026551416 0.271528637 -4.458151794 stable

MTMR14 -0.159781741 10.60941639 -2.305718712 0.02657785 0.271654872 -4.459036398 stable

ZNF630 0.190248947 2.8597243 2.305572457 0.026586859 0.271654872 -4.459337662 stable

PJA2 0.116621988 11.62515186 2.305261907 0.026605997 0.271732372 -4.4599773 stable

AHDC1 0.187107477 7.675297333 2.30499105 0.026622699 0.271784936 -4.46053513 stable

LOC439994 /// LOC642361 -0.158981051 9.279827493 -2.304096498 0.026677926 0.272230581 -4.462377108 stable

LSM8 -0.089403711 10.63364895 -2.303651559 0.026705433 0.272393103 -4.463293085 stable

SRSF1 0.118855509 6.624769447 2.303177349 0.026734778 0.272574219 -4.464269171 stable

HNRNPAB 0.121117804 11.86415456 2.302648631 0.026767531 0.2727899 -4.465357275 stable

HIST1H3C -0.008132382 2.575001948 -2.302387561 0.026783716 0.272798641 -4.465894489 stable

CXorf23 0.139241143 6.237305622 2.30214815 0.026798567 0.272798641 -4.466387093 stable

LINC01000 /// LINC01001 /// LINC01002 /// LOC100132062 /// LOC100133182 /// LOC100133331 /// LOC101929819 /// LOC729737 -0.273054231 11.92443995 -2.302073765 0.026803183 0.272798641 -4.466540138 stable

MALT1 -0.109400145 10.52963971 -2.30075644 0.026885041 0.27351343 -4.469249865 stable

RP11-337C18.10 /// RP11-337C18.8 0.044805675 2.648252443 2.299791973 0.026945115 0.273812845 -4.47123302 stable

LTBP4 -0.128960283 7.749025321 -2.299642599 0.02695443 0.273812845 -4.471540109 stable

NHLRC4 -0.123805452 5.557734536 -2.29963703 0.026954777 0.273812845 -4.471551559 stable

UQCC2 0.005162917 2.314631797 2.299536673 0.026961037 0.273812845 -4.471757867 stable

PSMB5 0.197919713 9.556946927 2.298672268 0.027015009 0.274242567 -4.473534589 stable

HYPK /// MIR1282 /// SERF2 -0.066274841 13.55991544 -2.297556893 0.027084795 0.274801172 -4.475826416 stable

ZBTB9 0.322598001 6.803290511 2.297247035 0.02710421 0.274801172 -4.476462949 stable

TNKS2 0.068792922 10.95821613 2.297183299 0.027108205 0.274801172 -4.476593872 stable

PAICS -0.123329556 9.498833252 -2.297046713 0.027116769 0.274801172 -4.47687443 stable

STOX2 -0.053294826 3.195967174 -2.296668785 0.027140476 0.274922974 -4.477650659 stable

IKZF3 0.316955014 6.674066298 2.296342141 0.027160982 0.275012248 -4.478321478 stable

PSMD5 0.122201527 9.841845924 2.296137564 0.027173831 0.275023962 -4.478741574 stable

SLC45A3 -0.506690807 7.746280287 -2.295727051 0.027199632 0.275166689 -4.47958447 stable

ECE1 -0.147609987 9.57360441 -2.29533635 0.027224209 0.275277246 -4.480386583 stable

ARFIP1 0.186638373 8.598611927 2.295181294 0.027233968 0.275277246 -4.480704886 stable

SFR1 -0.121934727 9.375423915 -2.293250919 0.027355725 0.276310368 -4.484666238 stable

ZNF680 0.157360428 8.150745496 2.293188473 0.027359672 0.276310368 -4.484794342 stable

TRIM58 -0.489774955 9.854704708 -2.292352851 0.027412537 0.276514249 -4.486508316 stable

MED26 0.098100697 9.836431389 2.292344413 0.027413071 0.276514249 -4.486525622 stable

GUCY2F 0.003733767 2.416494937 2.292311925 0.027415128 0.276514249 -4.48659225 stable

RP11-348N5.7 -0.164844758 5.067789673 -2.291644607 0.027457414 0.276665994 -4.48796065 stable

PNMA1 -0.216082154 10.71903729 -2.291525967 0.027464938 0.276665994 -4.488203901 stable

LOC646014 -0.137011144 7.678500427 -2.291517736 0.027465461 0.276665994 -4.488220778 stable

TRIM5 0.184287924 7.999546235 2.290941405 0.027502038 0.276915853 -4.489402308 stable

PRPF38A 0.096156578 10.07981121 2.29072978 0.02751548 0.276932648 -4.489836101 stable

FBXO7 -0.106992329 11.9020775 -2.290049892 0.027558704 0.277249053 -4.491229544 stable

AK091729 -0.136904511 4.271501707 -2.289267154 0.027608543 0.277498829 -4.492833393 stable

RP11-288H12.4 0.159263229 4.180009816 2.289221281 0.027611467 0.277498829 -4.492927374 stable

FAM206A -0.170258583 9.171062284 -2.289032562 0.027623496 0.277498829 -4.493313996 stable

LDHAL6CP /// LDHAL6CP 0.006426737 2.470518405 2.288919205 0.027630724 0.277498829 -4.493546214 stable

STK35 -0.061673083 2.548962582 -2.288340501 0.02766765 0.277693804 -4.494731586 stable

MAP6D1 -0.190506692 4.672326068 -2.288244965 0.02767375 0.277693804 -4.494927253 stable

NDE1 -0.130812343 9.964293834 -2.286814883 0.027765209 0.278492738 -4.497855455 stable

LINC01004 0.145670166 6.640238695 2.28643503 0.027789547 0.278618045 -4.498632998 stable

LOC102724814 -0.279991266 4.457750442 -2.286158228 0.027807295 0.278677194 -4.499199541 stable

TBC1D27 -0.418986139 5.954399834 -2.285428669 0.02785412 0.279027579 -4.500692507 stable

RPP14 0.13117974 9.07666659 2.285009345 0.027881066 0.279178604 -4.501550446 stable

LOC101927869 0.00455535 2.569742427 2.284575297 0.027908982 0.279339217 -4.502438383 stable

CNR1 -0.053352666 2.690662532 -2.284265087 0.027928949 0.279420161 -4.503072906 stable

C4orf46 0.292558051 6.107832652 2.283285217 0.027992102 0.279932926 -4.505076762 stable

PUS1 -0.109106355 8.431959076 -2.282403816 0.028049019 0.280027494 -4.506878688 stable

CDH17 0.022932465 2.222127024 2.282400341 0.028049244 0.280027494 -4.50688579 stable

TTC30B 0.338437632 4.308469387 2.282385019 0.028050234 0.280027494 -4.506917111 stable

RAPGEF1 0.155289259 8.835954087 2.282048258 0.028072009 0.280027494 -4.507605436 stable

CDC23 0.12565916 9.057966512 2.282019616 0.028073861 0.280027494 -4.507663976 stable

SERBP1 0.060463857 11.7005334 2.281888565 0.02808234 0.280027494 -4.507931816 stable

MLLT1 0.074194663 3.34789263 2.281517739 0.028106342 0.280027494 -4.50868964 stable

ZKSCAN5 0.232999939 7.549779101 2.281386272 0.028114856 0.280027494 -4.508958285 stable

PRSS3P2 -0.031936385 2.28118795 -2.281204554 0.028126628 0.280027494 -4.509329595 stable

BRD1 0.100743894 10.27990171 2.281137503 0.028130973 0.280027494 -4.509466598 stable

HIST1H2BO -0.372294217 3.36551941 -2.281030827 0.028137887 0.280027494 -4.509684558 stable

RNF5 /// RNF5P1 0.10360029 10.03187822 2.28092998 0.028144424 0.280027494 -4.5098906 stable

IRF6 0.03396201 2.483253016 2.280651142 0.028162506 0.280088927 -4.510460263 stable

FAM69A -0.5082312 5.370306541 -2.280425675 0.028177135 0.280115977 -4.51092085 stable

UBE2I -0.27758844 7.707024462 -2.28001131 0.028204038 0.280264972 -4.511767231 stable

OR1F1 -0.05421256 2.602174769 -2.27968164 0.028225459 0.280358555 -4.512440533 stable

ATAD3B -0.143491845 7.761208661 -2.279499576 0.028237295 0.280358555 -4.512812339 stable

IRF2 0.099554941 11.04909547 2.278393594 0.02830929 0.280954777 -4.515070458 stable

TTI2 0.316492006 6.664738518 2.278165552 0.028324156 0.280983747 -4.515535955 stable

RIC3 -0.328986806 4.785360688 -2.277693956 0.028354919 0.281170344 -4.516498501 stable

LSM14B 0.185941541 4.707057013 2.277350616 0.028377335 0.281274041 -4.517199177 stable

BROX 0.101037328 11.58329876 2.277128072 0.028391873 0.281299598 -4.517653292 stable

PPARD -0.088394835 7.899062398 -2.276862803 0.028409211 0.28135286 -4.518194548 stable

RBBP6 0.149084156 7.175719311 2.276240456 0.028449924 0.281520928 -4.519464199 stable

MRPS30 -0.112912416 10.05531372 -2.276237473 0.028450119 0.281520928 -4.519470284 stable

EPM2AIP1 -0.141523919 11.3359264 -2.275761832 0.02848127 0.28171066 -4.520440458 stable

BRD7 0.083114254 10.65957124 2.275419449 0.028503712 0.281814132 -4.521138729 stable

HK1 -0.095658943 12.40888098 -2.275174771 0.02851976 0.28185432 -4.521637687 stable

TBRG4 0.22023742 6.409645153 2.274864106 0.028540148 0.281937343 -4.522271149 stable

UTP23 -0.121895387 9.22711598 -2.27440105 0.02857056 0.282119288 -4.523215223 stable

RP11-803D5.4 0.058677162 3.502230409 2.2742132 0.028582906 0.282122756 -4.523598168 stable

MAPK3 -0.085775224 9.976643053 -2.273855276 0.028606442 0.282236632 -4.524327755 stable

REC8 -0.150096604 8.955804725 -2.273369417 0.028638419 0.282433652 -4.52531798 stable

AKAP1 -0.108212673 8.690186199 -2.272979168 0.028664127 0.282568704 -4.526113226 stable

CCDC65 0.146617664 4.575710138 2.272637068 0.02868668 0.282672556 -4.52681027 stable

DNAAF2 0.15931317 8.681251784 2.271891652 0.028735876 0.283038749 -4.528328811 stable

MIATNB -0.173198401 8.129289656 -2.270815322 0.028807045 0.28362097 -4.530520809 stable

ANXA7 -0.087332309 11.30462961 -2.27044771 0.028831388 0.283741873 -4.531269288 stable

TOP1P2 0.005629584 2.560747594 2.269806643 0.028873884 0.283893266 -4.532574315 stable

LIMS3 /// LIMS3L -0.292392653 6.261481261 -2.269732637 0.028878793 0.283893266 -4.532724951 stable

XYLT1 0.167615398 9.747312751 2.26966952 0.028882981 0.283893266 -4.532853422 stable

MSANTD3 -0.340159975 9.269116652 -2.269133166 0.028918588 0.284124522 -4.533945017 stable

MIS12 0.158090278 9.664800289 2.268712729 0.028946527 0.284152662 -4.534800557 stable

ERN2 -0.123985766 7.974314337 -2.268584337 0.028955064 0.284152662 -4.535061798 stable

HMGB1 -0.096787487 13.40687229 -2.268544782 0.028957694 0.284152662 -4.535142278 stable

BOLA1 0.230994798 6.406038212 2.268296844 0.028974188 0.284195942 -4.535646718 stable

FAM169A 0.433181935 6.660759573 2.267665684 0.029016211 0.284489499 -4.536930651 stable

LOC101929741 0.012812799 2.200784692 2.266654827 0.029083629 0.285031686 -4.53898641 stable

DHRS3 0.270247655 9.090782704 2.266133145 0.029118477 0.285254353 -4.540047072 stable

CACFD1 -0.150199021 6.126559887 -2.265759175 0.029143481 0.285279941 -4.540807296 stable

SCD5 0.386059435 8.816000942 2.265528203 0.029158933 0.285279941 -4.541276779 stable

NSFL1C -0.262521691 9.109832443 -2.265432927 0.02916531 0.285279941 -4.541470431 stable

CHD6 0.264478111 5.489905692 2.265368769 0.029169604 0.285279941 -4.54160083 stable

SIRT3 -0.158228649 7.740772567 -2.265064765 0.02918996 0.285360373 -4.542218673 stable

TARP /// TRGC2 /// TRGV9 0.426068229 11.9409594 2.264053533 0.029257764 0.28579698 -4.544273395 stable

TOMM5 0.140392392 10.81155573 2.264036272 0.029258923 0.28579698 -4.544308462 stable

DPY30 /// MEMO1 -0.113939101 10.09078323 -2.263663434 0.029283959 0.285868464 -4.545065849 stable

CDK2AP1 -0.237184612 12.15946923 -2.263565336 0.029290549 0.285868464 -4.54526511 stable

RAB5B 0.077749133 9.707809875 2.263219846 0.02931377 0.285976432 -4.545966836 stable

NUMB -0.294898856 7.599594998 -2.262305108 0.02937533 0.286353115 -4.547824365 stable

TBCC 0.166662975 10.68712257 2.262146053 0.029386046 0.286353115 -4.548147293 stable

C1RL-AS1 0.035483695 4.264694059 2.262103636 0.029388904 0.286353115 -4.548233409 stable

THNSL1 0.022356226 3.253890252 2.260970891 0.029465328 0.286708498 -4.550532687 stable

HAVCR1P1 0.003858181 2.216401748 2.26085435 0.029473201 0.286708498 -4.550769195 stable

CTNNAL1 -0.541370715 6.642392099 -2.260848385 0.029473604 0.286708498 -4.5507813 stable

MYOC -0.036556134 2.98223997 -2.260840511 0.029474136 0.286708498 -4.55079728 stable

CUL9 -0.148405139 9.325209958 -2.260629312 0.029488408 0.286728752 -4.551225859 stable

CEP152 0.076399537 4.485642409 2.260122301 0.029522697 0.286943531 -4.552254601 stable

ANXA9 0.007159418 2.667461386 2.259164556 0.029587564 0.287455227 -4.554197414 stable

ZBTB32 -0.038978248 1.990343096 -2.258018722 0.029665339 0.28809184 -4.556520944 stable

XK -0.467968038 8.692229668 -2.25717324 0.029722844 0.288510936 -4.558234839 stable

KLK8 /// KLK9 -0.050082064 3.538392115 -2.257023682 0.029733026 0.288510936 -4.558537959 stable

SLC38A9 0.128565035 8.793887851 2.256403926 0.029775255 0.288709706 -4.559793904 stable

GTF2E2 0.121150767 9.262401677 2.256362782 0.02977806 0.288709706 -4.559877274 stable

MRPL17 0.147408156 8.645612075 2.254669542 0.029893715 0.289711611 -4.563307259 stable

PPP2R5B -0.110478559 7.889615426 -2.253807162 0.029952774 0.290122908 -4.56505342 stable

CHD1L -0.139241045 9.808225733 -2.253689727 0.029960824 0.290122908 -4.565291164 stable

TMEM45A -0.780664549 5.417645794 -2.253315086 0.029986519 0.29025223 -4.566049553 stable

CFC1 /// CFC1B 0.009441019 2.899980055 2.252582688 0.030036808 0.290619403 -4.567531874 stable

AKAP17A -0.13570524 11.52754109 -2.251681346 0.030098801 0.291099466 -4.569355619 stable

LACTB -0.371939853 7.503720853 -2.251448779 0.030114815 0.291134635 -4.569826096 stable

DCAF4L2 0.004307836 2.614889093 2.25079242 0.030160052 0.291452168 -4.571153692 stable

PRICKLE4 /// TOMM6 -0.077023795 12.44068564 -2.250224368 0.030199251 0.291711124 -4.572302435 stable

PURA 0.104300373 11.29866745 2.249408441 0.030255635 0.292001795 -4.573952054 stable

FCRLB 0.387542581 6.152424869 2.249394794 0.030256579 0.292001795 -4.573979641 stable

PAPOLA -0.131175564 10.84805069 -2.249250126 0.030266586 0.292001795 -4.574272078 stable

MCPH1 0.186966293 7.645376118 2.248893854 0.030291244 0.292119861 -4.574992194 stable

MRPS24 /// URGCP-MRPS24 -0.103792386 11.04309626 -2.248350545 0.03032888 0.292192282 -4.576090196 stable

ZNF175 0.191729953 6.915652019 2.248348733 0.030329006 0.292192282 -4.576093856 stable

LOC100130856 /// PSPN 0.078957957 3.689093663 2.248247528 0.030336021 0.292192282 -4.576298365 stable

RBAK 0.137605919 5.928683932 2.247151081 0.030412118 0.292805335 -4.578513531 stable

THAP6 0.154548262 6.911059431 2.246909086 0.030428936 0.292847387 -4.579002324 stable

ASF1A 0.158649891 10.96162634 2.246365695 0.030466731 0.293091201 -4.580099746 stable

RAB31 -0.214794265 12.49274742 -2.245836062 0.03050361 0.293326002 -4.581169187 stable

ZCCHC8 0.098151215 10.38054801 2.245424337 0.030532305 0.293435948 -4.582000413 stable

IGHA1 /// IGHG1 /// IGHG3 /// IGHM /// IGHV4-31 -0.37100585 7.385561087 -2.24521271 0.030547065 0.293435948 -4.58242762 stable

WWC2 0.018935814 2.863741945 2.245135232 0.03055247 0.293435948 -4.582584014 stable

LNPEP 0.21013001 9.13736498 2.244379075 0.030605265 0.293720316 -4.584110153 stable

DMTF1 -0.143115017 10.58299962 -2.244353488 0.030607053 0.293720316 -4.58416179 stable

FAM193B -0.144172265 10.90889774 -2.243870973 0.030640787 0.293781059 -4.585135426 stable

AP1G2 -0.158627945 10.99441628 -2.24384908 0.030642318 0.293781059 -4.585179598 stable

RTBDN 0.009994635 2.63485167 2.24372708 0.030650853 0.293781059 -4.585425749 stable

CTHRC1 0.004337475 2.309572243 2.242966608 0.030704103 0.294065152 -4.586959858 stable

AGBL2 -0.179554161 3.330841593 -2.242946701 0.030705498 0.294065152 -4.58700001 stable

RPA1 -0.158796181 12.13547438 -2.242284249 0.030751953 0.294319455 -4.588336048 stable

SLC35B3 0.114796684 9.750935745 2.242211227 0.030757077 0.294319455 -4.5884833 stable

BEND2 -0.497339857 9.191527059 -2.241409438 0.030813396 0.294738465 -4.590099907 stable

PNP 0.220189787 10.08015822 2.240622095 0.030868789 0.29514829 -4.591686957 stable

SEC31A -0.072945952 12.20082357 -2.239849616 0.030923223 0.295548611 -4.593243627 stable

RACGAP1 0.143597557 8.078222234 2.238655497 0.031007537 0.296199356 -4.59564916 stable

GPD2 0.260238926 7.585342227 2.238528774 0.031016497 0.296199356 -4.595904384 stable

ARID4A 0.06821922 9.798800041 2.237263674 0.031106069 0.29693419 -4.59845172 stable

LOC101060604 /// SLC7A5P1 /// SLC7A5P2 /// SMG1P3 0.140387408 8.906923833 2.236848037 0.031135547 0.297095012 -4.599288382 stable

BC069776 0.0858635 3.363730761 2.235714617 0.03121606 0.297725549 -4.60156931 stable

CAPN5 0.128550926 3.432346161 2.235561608 0.031226943 0.297725549 -4.60187716 stable

HERC2 /// LOC101929047 /// LOC101929832 0.193432831 5.489619964 2.235342378 0.031242542 0.297753582 -4.602318219 stable

NFYA 0.108060876 9.195402077 2.234607276 0.031294899 0.298018475 -4.603796895 stable

LOC101060604 /// SLC7A5P1 /// SMG1P3 0.14616802 3.803760429 2.234535874 0.031299989 0.298018475 -4.603940503 stable

GP1BB /// SEPT5 /// SEPT5-GP1BB -0.440418269 12.8106816 -2.234261819 0.031319531 0.298018475 -4.604491662 stable

WDR31 0.007015809 2.903119224 2.23424097 0.031321018 0.298018475 -4.604533589 stable

LINC00106 /// LINC00106 -0.205292051 6.492026956 -2.233813623 0.031351514 0.298188017 -4.605392928 stable

ELK1 -0.122628757 8.777322541 -2.233164451 0.03139789 0.298441223 -4.606698085 stable

ABHD17C 0.252421028 3.313833716 2.232943611 0.031413681 0.298441223 -4.607142017 stable

GALK2 0.110880697 7.635382471 2.232907112 0.031416291 0.298441223 -4.607215384 stable

CTB-31O20.2 -0.292672202 9.827789089 -2.232731005 0.031428889 0.298441223 -4.607569365 stable

KIAA2022 0.010905164 2.446759226 2.232489459 0.031446176 0.29848487 -4.608054845 stable

LCORL 0.186650549 6.777624627 2.231865628 0.031490861 0.298788437 -4.609308488 stable

P2RY12 -0.411309954 9.112920438 -2.231628569 0.031507856 0.298829145 -4.609784807 stable

LOC284112 /// MIR195 /// MIR497 /// MIR497HG -0.036470633 3.787824375 -2.231253247 0.03153478 0.298895549 -4.610538858 stable

FAM136A /// LOC100287852 -0.067621812 9.926753556 -2.231176719 0.031540273 0.298895549 -4.610692596 stable

ANKRD20A1 /// ANKRD20A12P /// ANKRD20A2 /// ANKRD20A3 /// ANKRD20A4 /// ANKRD20A5P /// ANKRD20A8P 0.12756657 3.873393015 2.230559707 0.031584586 0.299186681 -4.611931977 stable

C18orf32 /// RPL17 /// RPL17-C18orf32 /// SNORD58A /// SNORD58B /// SNORD58C 0.04197664 15.27986701 2.23039488 0.031596434 0.299186681 -4.612263016 stable

SYT17 -0.17812317 5.942927391 -2.229823529 0.031637531 0.29945528 -4.613410376 stable

ABHD2 0.261535086 8.541513213 2.229257048 0.031678325 0.299604227 -4.614547734 stable

OSBPL3 0.161883806 8.889185839 2.229251253 0.031678743 0.299604227 -4.614559368 stable

IGHA1 /// IGHG1 /// IGHM /// IGHV3-23 /// IGHV4-31 -0.506125302 9.401332769 -2.22901713 0.031695617 0.29964333 -4.615029365 stable

PNCK -0.029389729 2.39346883 -2.228367428 0.031742485 0.299965842 -4.616333428 stable

PPIA 0.05235313 14.79424399 2.227848716 0.031779947 0.300199255 -4.617374362 stable

BHMT 0.005971064 2.556153687 2.227486798 0.03180611 0.300325773 -4.618100535 stable

LOC101928789 /// SP140 -0.245804132 9.445886428 -2.226697791 0.031863211 0.300744217 -4.619683331 stable

KLRG1 0.452863324 10.13412921 2.225378871 0.031958867 0.30152608 -4.622328199 stable

RP4-647C14.3 -0.16815147 3.492491185 -2.225074099 0.031981008 0.301611961 -4.622939196 stable

ZNF33A 0.185397706 3.193974386 2.224900621 0.031993616 0.301611961 -4.62328695 stable

ARPC4 /// ARPC4-TTLL3 /// TTLL3 -0.154631114 6.350587544 -2.222820403 0.032145152 0.302919123 -4.627455324 stable

SYNGR2 -0.142681513 12.05108635 -2.222609257 0.032160569 0.302943033 -4.627878255 stable

DNAJC10 -0.129397218 9.588622978 -2.222382608 0.032177126 0.30297765 -4.628332202 stable

FZR1 0.128897967 6.246023687 2.22220053 0.032190431 0.302981648 -4.628696855 stable

PPP6C -0.049088568 11.32258908 -2.221878112 0.032214005 0.303082246 -4.629342513 stable

ZSCAN26 0.260189987 7.271422934 2.220925421 0.032283751 0.303616997 -4.631249902 stable

CDKN2C 0.252645566 6.830085973 2.220733198 0.03229784 0.303628095 -4.631634677 stable

NEK7 0.103966759 11.9378983 2.219291715 0.032403667 0.304501257 -4.634519287 stable

MAL2 0.01027819 2.909089129 2.219108294 0.032417155 0.304506349 -4.634886236 stable

GNL3 -0.176113506 7.775673391 -2.218652803 0.032450671 0.304647441 -4.635797379 stable

IGKV1-37 /// IGKV1-37 /// IGKV1D-37 /// IGKV1D-37 -0.555326525 9.87291419 -2.218552183 0.032458079 0.304647441 -4.635998635 stable

ADNP 0.13523234 11.36520004 2.218190499 0.032484721 0.304667509 -4.636722003 stable

SEMA4F 0.096363026 3.293515841 2.218171461 0.032486124 0.304667509 -4.636760076 stable

SPIN1 0.135923614 8.666912731 2.217219348 0.032556351 0.305204436 -4.638663852 stable

RP11-245J9.5 0.02634713 2.996676112 2.216984893 0.032573665 0.305228045 -4.639132555 stable

LOC374443 0.286484492 8.54871224 2.216833857 0.032584823 0.305228045 -4.639434474 stable

KRAS -0.310337999 7.28685171 -2.215841504 0.032658219 0.305738714 -4.641417773 stable

C7orf25 /// PSMA2 0.285257735 6.502049211 2.215745376 0.032665337 0.305738714 -4.641609857 stable

MIR6805 /// RPL28 0.035865525 15.36155226 2.215438141 0.032688095 0.305745586 -4.642223731 stable

TP53BP2 0.104181152 9.702363333 2.215136805 0.032710429 0.305745586 -4.642825756 stable

FBXW9 -0.170553363 5.833297163 -2.215077115 0.032714855 0.305745586 -4.642945001 stable

LAIR2 0.792802542 8.972836585 2.214911438 0.032727142 0.305745586 -4.643275964 stable

SLC5A2 -0.046516288 3.244044576 -2.214858552 0.032731065 0.305745586 -4.643381608 stable

ATP2C1 -0.104510234 8.222209326 -2.213381981 0.032840765 0.306648521 -4.646330375 stable

GUSBP11 -0.275671647 12.1055412 -2.213163613 0.032857016 0.306678518 -4.646766335 stable

NDUFB2 0.094566162 12.1971464 2.212961639 0.032872053 0.306697168 -4.647169537 stable

TREML1 -0.50504557 10.85864883 -2.212567303 0.03290143 0.306849536 -4.647956666 stable

RP11-332H18.4 -0.0063855 2.509420662 -2.21237579 0.032915705 0.306861 -4.648338905 stable

ZKSCAN2 0.220115077 5.026204478 2.211775679 0.032960474 0.307085329 -4.649536493 stable

TDGF1 /// TDGF1P3 0.019173436 3.107396134 2.211568868 0.032975914 0.307085329 -4.649949149 stable

LOC101929988 -0.074356494 3.354082752 -2.211528412 0.032978935 0.307085329 -4.650029869 stable

PRB3 -0.202327834 6.328498211 -2.210652279 0.033044425 0.307518975 -4.651777687 stable

ZGPAT -0.08647179 9.461404259 -2.210555652 0.033051655 0.307518975 -4.65197042 stable

CCDC185 -0.062848704 3.827758699 -2.209094464 0.033161155 0.308331254 -4.654884087 stable

ZNF786 0.205936857 7.770492722 2.2090101 0.033167487 0.308331254 -4.655052267 stable

TTC13 0.097498413 9.809001822 2.208866282 0.033178283 0.308331254 -4.655338958 stable

ATAD1 0.112526853 10.17838368 2.206608154 0.033348218 0.309713521 -4.659838464 stable

MAP4K3 0.227172816 7.354247845 2.206539998 0.033353359 0.309713521 -4.659974216 stable

CASC14 -0.134972995 5.772040865 -2.205623053 0.033422593 0.310233941 -4.661800244 stable

CS -0.069150969 12.07511228 -2.205277865 0.033448689 0.310353698 -4.662487508 stable

HCG8 /// ZNRD1-AS1 -0.337570734 7.112776033 -2.204377147 0.03351687 0.310863684 -4.664280431 stable

BBS4 0.103608517 7.526801846 2.203854656 0.033556477 0.310961875 -4.665320215 stable

GMNN 0.289427206 7.014336252 2.203721262 0.033566596 0.310961875 -4.665585646 stable

THUMPD2 -0.093215007 7.643483811 -2.203627888 0.03357368 0.310961875 -4.665771435 stable

MTURN -0.425855236 9.971202542 -2.203408267 0.033590348 0.310961875 -4.666208401 stable

TMEM169 0.046805687 2.697258378 2.203365959 0.03359356 0.310961875 -4.666292574 stable

PIP5KL1 -0.102099367 4.321302377 -2.202799733 0.033636571 0.311153207 -4.667418983 stable

PLS1 0.234975656 3.107581513 2.202630707 0.03364942 0.311153207 -4.667755189 stable

MIR5193 /// UBA7 -0.126529514 9.923575021 -2.202565991 0.033654341 0.311153207 -4.667883907 stable

RP11-629E24.2 0.008953952 2.574458707 2.202397641 0.033667145 0.311153207 -4.668218741 stable

C7orf13 -0.11240822 4.235792328 -2.200034008 0.033847364 0.312695938 -4.672917694 stable

WDR82 0.120801716 12.31426669 2.199802264 0.03386508 0.31273677 -4.673378196 stable

UIMC1 -0.107651636 9.981396739 -2.19962592 0.033878567 0.312738525 -4.673728586 stable

ACVR1 -0.174707766 9.806159419 -2.198817525 0.033940451 0.313131579 -4.675334564 stable

PTER 0.147425598 9.312065613 2.198721991 0.033947771 0.313131579 -4.675524325 stable

CALM1 0.207542604 8.725270037 2.198469046 0.033967159 0.313154691 -4.67602672 stable

TGFB1I1 -0.519720685 7.530635874 -2.198341949 0.033976905 0.313154691 -4.676279141 stable

OSGIN2 0.146756132 9.19268261 2.197803171 0.034018244 0.313303619 -4.677349057 stable

C11orf65 0.016274806 2.856092152 2.197755834 0.034021879 0.313303619 -4.67744305 stable

LIG4 0.17520628 8.096143577 2.197498542 0.034041638 0.313303619 -4.677953906 stable

PIK3C2B -0.2256807 8.467864105 -2.19707395 0.034074268 0.313303619 -4.678796834 stable

PPAPDC2 0.25429802 7.934081192 2.196992745 0.034080512 0.313303619 -4.678958035 stable

ZNF501 0.16433607 3.5163071 2.196928048 0.034085487 0.313303619 -4.679086461 stable

KCTD1 0.186284761 3.964793451 2.196854221 0.034091165 0.313303619 -4.679233007 stable

STX6 0.172191374 9.198117163 2.196744253 0.034099624 0.313303619 -4.679451285 stable

LOC113230 /// MIR1199 -0.308543775 6.9717976 -2.196297746 0.034133991 0.313496917 -4.680337481 stable

C17orf70 -0.075564846 8.453362915 -2.195725094 0.034178112 0.313779615 -4.681473838 stable

ADCY9 0.331311045 8.453107571 2.194903241 0.034241521 0.313968057 -4.6831043 stable

NKX2-3 0.004032042 2.483058817 2.194829722 0.034247199 0.313968057 -4.68325013 stable

GAPDHP73 /// GAPDHP73 -0.15109203 7.371941966 -2.19476256 0.034252386 0.313968057 -4.683383347 stable

UPF2 -0.098445529 10.22374131 -2.194538079 0.034269729 0.313968057 -4.683828587 stable

RAP2A /// RAP2B 0.206451233 7.6954161 2.19444184 0.034277166 0.313968057 -4.684019458 stable

MTFR1L -0.115002606 9.45342176 -2.19442163 0.034278728 0.313968057 -4.684059541 stable

GSK3A -0.084061313 7.923171966 -2.192644571 0.034416326 0.315105644 -4.687582801 stable

AC018755.16 0.122347581 8.198415081 2.191990123 0.034467123 0.315233869 -4.688879775 stable

FAM73B -0.136437488 8.598692484 -2.191581268 0.034498891 0.315233869 -4.689689883 stable

ACOT1 /// ACOT2 0.207091644 6.075695418 2.191402121 0.03451282 0.315233869 -4.69004481 stable

ZNF625 /// ZNF625-ZNF20 0.207649773 5.139806184 2.191329129 0.034518496 0.315233869 -4.690189416 stable

ZNF253 0.118494978 6.241569972 2.191252942 0.034524421 0.315233869 -4.690340346 stable

SLC39A7 0.225065813 7.882267645 2.191240118 0.034525419 0.315233869 -4.69036575 stable

ARMCX4 -0.143144305 4.830213365 -2.191101788 0.034536181 0.315233869 -4.690639779 stable

GAS8 -0.149079071 6.140024983 -2.191058248 0.034539569 0.315233869 -4.690726029 stable

EID1 -0.059164131 13.02487649 -2.190911993 0.034550951 0.315233869 -4.691015737 stable

D21S2088E 0.017457452 3.154995653 2.18989012 0.034630572 0.315837795 -4.693039491 stable

ZNF414 -0.038581599 6.689778014 -2.189545247 0.03465748 0.315947638 -4.693722325 stable

SMIM15 0.182611732 10.73854574 2.189373778 0.034670865 0.315947638 -4.694061794 stable

CCDC88B -0.074605852 9.261633996 -2.189219489 0.034682913 0.315947638 -4.694367234 stable

PTGS1 -0.487285622 10.15203936 -2.18765379 0.034805387 0.316925807 -4.697465838 stable

ZBTB3 0.167372716 6.774017964 2.187502527 0.034817239 0.316925807 -4.697765105 stable

VWA3A -0.045660933 2.648171184 -2.187105967 0.03484833 0.317086097 -4.698549603 stable

TEX11 -0.017743778 3.246226686 -2.186830924 0.034869907 0.317159742 -4.699093645 stable

P2RX5 /// P2RX5-TAX1BP3 /// TAX1BP3 -0.1809946 9.384329762 -2.18607014 0.034929655 0.317554467 -4.700598216 stable

CDC42-IT1 -0.320883503 8.659957255 -2.185934621 0.034940307 0.317554467 -4.700866182 stable

NPM3 0.196547163 7.408546164 2.185537849 0.034971511 0.317706264 -4.701650662 stable

RP3-452M16.1 0.007457729 2.447494396 2.185378839 0.034984024 0.317706264 -4.701965019 stable

CCDC12 0.103608028 10.34332596 2.184109249 0.03508407 0.318469083 -4.704474309 stable

FUBP1 -0.0967274 9.425991945 -2.183945197 0.035097016 0.318469083 -4.704798468 stable

BC045789 -0.077279557 3.097634115 -2.183385596 0.035141208 0.318469083 -4.705904069 stable

NT5C1B-RDH14 /// RDH14 0.115463017 10.23269125 2.183285962 0.035149082 0.318469083 -4.706100893 stable

HERC6 0.009100884 2.380321838 2.183114372 0.035162645 0.318469083 -4.706439848 stable

SBNO1 -0.188126704 5.296242363 -2.18301607 0.035170417 0.318469083 -4.70663402 stable

ZNF271 -0.206098424 9.681726764 -2.182960159 0.035174839 0.318469083 -4.706744458 stable

C8orf33 -0.11122487 8.984072255 -2.182941182 0.035176339 0.318469083 -4.706781941 stable

CAMK4 -0.257019101 7.214700513 -2.18173361 0.035271956 0.318924625 -4.709166606 stable

MSRB3 -0.117618199 2.260606156 -2.181728263 0.03527238 0.318924625 -4.709177164 stable

TRIM68 0.149636554 8.544312793 2.18164609 0.035278895 0.318924625 -4.709339398 stable

EIF3J-AS1 -0.126056715 7.31750502 -2.181459708 0.035293676 0.318924625 -4.709707356 stable

RFNG -0.083014773 8.276640801 -2.181449924 0.035294452 0.318924625 -4.709726671 stable

RP11-199F11.2 -0.348825876 7.111336291 -2.180977542 0.035331939 0.31914076 -4.710659138 stable

MDN1 -0.422584192 6.290151459 -2.180694678 0.035354404 0.319166956 -4.711217429 stable

ZNF607 0.130356041 6.993520925 2.180599338 0.035361978 0.319166956 -4.711405588 stable

PMM1 -0.104046356 7.91675398 -2.180417501 0.035376429 0.319174906 -4.711764438 stable

EIF4G2 -0.142716348 2.707530966 -2.180102914 0.035401441 0.319278106 -4.712385212 stable

LARP4 0.193538037 6.961761465 2.179770832 0.035427862 0.31939392 -4.713040433 stable

ATAD3A /// ATAD3B -0.12520689 6.327160776 -2.179484904 0.035450624 0.319476681 -4.713604528 stable

ARHGAP18 -0.305778088 7.748077423 -2.178079542 0.035562693 0.320363883 -4.71637626 stable

RGS10 -0.199448803 3.600324742 -2.177446891 0.035613245 0.3205819 -4.717623555 stable

PEX11B 0.118072485 10.33176906 2.17743553 0.035614154 0.3205819 -4.717645951 stable

PPBP -0.133540252 15.16334323 -2.177097963 0.035641154 0.320702208 -4.718311356 stable

CTR9 0.105429355 11.12337048 2.176751431 0.03566889 0.320829045 -4.718994353 stable

GLS 0.149778588 9.228418335 2.176333934 0.035702331 0.320871458 -4.719817104 stable

KCNQ4 -0.028700885 2.411228289 -2.176195467 0.035713428 0.320871458 -4.72008995 stable

TRPM4 -0.324852099 3.41396546 -2.176181714 0.035714531 0.320871458 -4.72011705 stable

CRHR1-IT1 /// MGC57346 -0.101974516 5.977930208 -2.175908425 0.035736443 0.320945731 -4.720655517 stable

SNHG8 /// SNORA24 -0.200696824 11.02305802 -2.175384691 0.035778469 0.321200518 -4.721687296 stable

HEATR5A 0.210208911 8.094029756 2.174921994 0.035815633 0.321411485 -4.722598666 stable

COBLL1 -0.700307455 6.08456508 -2.174565573 0.035844284 0.321545924 -4.723300604 stable

APOBR 0.195460251 10.08579712 2.173256485 0.035949692 0.322249528 -4.725877956 stable

SLC25A53 0.173558711 3.589539811 2.173128646 0.03596 0.322249528 -4.726129584 stable

RELB -0.226432566 8.220139312 -2.172868035 0.035981023 0.322249528 -4.72664251 stable

TMEM219 -0.143734322 10.91356217 -2.17283178 0.035983948 0.322249528 -4.726713863 stable

HIST1H4H -0.925082775 8.32783233 -2.17270832 0.035993912 0.322249528 -4.726956834 stable

ZAK -0.264772779 5.188543519 -2.172571931 0.036004922 0.322249528 -4.727225236 stable

IGK /// IGKC -0.48115568 9.74388292 -2.172382131 0.036020248 0.322264073 -4.727598724 stable

UBN1 0.069867348 9.340890125 2.171766615 0.03606999 0.322586403 -4.728809763 stable

PTRH2 0.304649327 8.894061857 2.171471298 0.036093878 0.322677346 -4.72939071 stable

GTF2A1 0.119777767 5.774825449 2.170777455 0.036150056 0.323056788 -4.730755392 stable

FPGT 0.221758709 7.837142808 2.170362684 0.036183676 0.323234422 -4.731571021 stable

ABCB1 /// ABCB4 0.262131235 9.252400242 2.170146551 0.036201205 0.323268243 -4.731995987 stable

ZNF451 -0.203808503 10.68084723 -2.169567394 0.036248216 0.323520735 -4.733134581 stable

ST7-AS1 -0.038239535 2.339010441 -2.169459376 0.03625699 0.323520735 -4.733346912 stable

SLC30A5 0.127800632 9.069269825 2.16875756 0.036314041 0.323906928 -4.734726276 stable

MEA1 -0.087617499 10.61347768 -2.167981977 0.036377182 0.324245867 -4.736250217 stable

AK311120 /// LSAMP 0.015318508 3.381923174 2.16795216 0.036379612 0.324245867 -4.736308796 stable

MIR210HG -0.053881656 2.388297371 -2.167171257 0.036443287 0.324495596 -4.737842742 stable

CHST10 0.168621689 5.539243837 2.167068263 0.036451693 0.324495596 -4.738045023 stable

PNPLA2 -0.162943923 8.89249649 -2.167056577 0.036452647 0.324495596 -4.738067975 stable

DEPTOR -0.199930156 3.523326634 -2.166932021 0.036462815 0.324495596 -4.738312593 stable

MAN1A1 0.223118774 9.307280047 2.166461795 0.036501223 0.324714549 -4.739235982 stable

POMGNT2 0.180649785 7.533609299 2.16619058 0.036523393 0.324774693 -4.7397685 stable

DNAJC27-AS1 0.007465679 2.806643832 2.166041303 0.0365356 0.324774693 -4.740061574 stable

BBS10 0.182447445 6.493638357 2.165671403 0.036565864 0.32480662 -4.740787733 stable

PCDHA5 /// PCDHA6 0.008012111 2.691207144 2.165659848 0.03656681 0.32480662 -4.740810416 stable

LOC101930075 /// LOC102724993 /// NPIPA1 /// NPIPA2 /// NPIPA3 /// NPIPA5 /// NPIPA7 /// NPIPA8 /// PKD1P1 -0.204808811 11.91725785 -2.165388223 0.036589048 0.324881463 -4.741343583 stable

SIN3B -0.140227361 9.53712906 -2.164998417 0.036620983 0.3249201 -4.742108635 stable

PCDH7 -0.007776246 2.499093912 -2.164997872 0.036621027 0.3249201 -4.742109704 stable

SLC2A3 0.185186895 11.01306883 2.1645561 0.036657249 0.325118839 -4.742976617 stable

MAP1LC3C 0.030694642 2.916925628 2.164323101 0.036676366 0.325165777 -4.743433786 stable

USP1 0.076633054 9.562170777 2.163957175 0.036706407 0.325309496 -4.744151697 stable

IGH -0.182585807 7.363577439 -2.163293495 0.036760948 0.325625636 -4.745453529 stable

LOC101928826 /// TPT1 0.032148241 15.8021988 2.163186265 0.036769767 0.325625636 -4.745663836 stable

RNF11 -0.254653207 12.39647678 -2.163004007 0.036784761 0.325635812 -4.746021272 stable

MBNL2 /// MBNL2 -0.367984263 9.27656219 -2.162457008 0.036829792 0.325911793 -4.747093883 stable

HRK /// LOC283454 -0.232834628 3.147555952 -2.161821104 0.036882205 0.326144203 -4.748340563 stable

2-Mar -0.369303402 10.84777744 -2.161801906 0.036883788 0.326144203 -4.748378195 stable

ZSCAN32 0.172136055 8.122095497 2.161253677 0.036929029 0.326421527 -4.74945275 stable

ZNF25 0.164869108 7.843644664 2.160781991 0.036967992 0.32664318 -4.750377106 stable

CXCR6 0.57206175 5.429178724 2.160551361 0.036987057 0.326688907 -4.750829013 stable

MEIS1 -0.733608883 7.244890383 -2.159959072 0.037036056 0.326951098 -4.751989392 stable

STX4 0.105402694 9.609135233 2.159856569 0.037044542 0.326951098 -4.752190186 stable

MAP3K8 0.271018692 8.964246077 2.159247394 0.037095009 0.327160828 -4.753383342 stable

ZC3H6 0.122192401 8.274964902 2.159233945 0.037096124 0.327160828 -4.753409682 stable

DCTN5 0.131181903 7.301777463 2.158979057 0.037117258 0.327224527 -4.753908836 stable

GOSR1 -0.258572852 4.316696489 -2.158711152 0.037139484 0.327297791 -4.754433432 stable

CSNK2B /// LY6G5B -0.309194205 7.257303159 -2.158223097 0.037180004 0.327532158 -4.755388984 stable

MST1L 0.006238573 2.969341228 2.156690506 0.037307497 0.328410984 -4.758388507 stable

LAG3 0.318129973 3.911429832 2.156688205 0.037307689 0.328410984 -4.758393009 stable

FRMD3 -0.436235981 8.079680228 -2.156233765 0.037345567 0.328621433 -4.759282098 stable

GNA13 0.100303957 10.31190284 2.15554301 0.037403208 0.329005559 -4.760633246 stable

LOC100128644 -0.089441574 2.902247514 -2.15525972 0.037426871 0.329090626 -4.761187275 stable

SENP6 -0.077431664 10.88830432 -2.153871349 0.037543028 0.329988626 -4.763901683 stable

DNAL1 0.071577709 2.486095573 2.153433693 0.03757971 0.330138062 -4.76475706 stable

RIMBP3 /// RIMBP3B /// RIMBP3C 0.211555904 6.056468791 2.153326024 0.03758874 0.330138062 -4.764967475 stable

PNOC -0.44327123 8.350174223 -2.153166305 0.037602137 0.330138062 -4.765279591 stable

MAPK6 0.086869549 9.85307305 2.152421735 0.037664649 0.33053145 -4.766734364 stable

TUG1 -0.143826277 10.67307595 -2.152297978 0.037675049 0.33053145 -4.766976128 stable

FXYD5 -0.089368862 13.23156803 -2.15177146 0.03771932 0.330796467 -4.768004577 stable

PNMA3 -0.076723094 5.222403635 -2.15145687 0.037745794 0.330905259 -4.768618971 stable

KIAA0513 -0.27338393 4.854002924 -2.151024819 0.037782179 0.330966831 -4.769462654 stable

AGGF1 0.069671635 8.41685851 2.150853232 0.037796638 0.330966831 -4.769797682 stable

NDUFS4 -0.307326901 5.635550701 -2.150642263 0.037814422 0.330966831 -4.770209575 stable

DCTN2 -0.096479177 10.36476801 -2.150548741 0.037822308 0.330966831 -4.770392156 stable

LOC102724938 0.01276685 2.865274531 2.150457713 0.037829985 0.330966831 -4.770569861 stable

SMURF1 0.19559372 7.702600448 2.150371655 0.037837244 0.330966831 -4.770737859 stable

SOS2 0.153212579 9.126471733 2.149631313 0.037899744 0.33139029 -4.772182899 stable

TPST2 0.201020228 10.76831672 2.148940217 0.037958169 0.331777815 -4.773531467 stable

VDAC2 -0.072680365 11.62399011 -2.148518391 0.03799387 0.331857615 -4.774354428 stable

MDM2 0.00401355 2.539245846 2.148498933 0.037995517 0.331857615 -4.774392386 stable

ID3 0.564463026 7.694918496 2.147485228 0.038081434 0.332338946 -4.77636954 stable

SELP -0.406103133 8.534788388 -2.147402453 0.038088457 0.332338946 -4.776530953 stable

MLXIP -0.201801708 5.912515784 -2.147206179 0.038105115 0.332338946 -4.776913675 stable

FMO1 -0.009213472 2.455840163 -2.147182278 0.038107144 0.332338946 -4.776960278 stable

C6orf136 0.076118524 9.32206007 2.146873351 0.038133377 0.332444464 -4.777562603 stable

C5orf28 -0.315276663 9.333702014 -2.144429399 0.038341472 0.334134779 -4.782325265 stable

LOC730098 -0.0614968 2.909823709 -2.144203474 0.03836076 0.334179047 -4.782765324 stable

LOC101928731 0.004243816 2.469848774 2.143430431 0.03842682 0.334617286 -4.784270782 stable

ABHD10 0.173964582 9.441913992 2.143281966 0.038439518 0.334617286 -4.784559861 stable

VCX2 -0.051340073 2.828864093 -2.142988137 0.038464661 0.334712279 -4.785131935 stable

R3HCC1L 0.193052293 7.024461913 2.142570263 0.038500443 0.334899749 -4.785945412 stable

RUFY1 -0.331200304 11.88096534 -2.141030772 0.038632522 0.335924417 -4.788941271 stable

AP006547.3 -0.02194662 3.837565054 -2.14059886 0.038669649 0.335939053 -4.78978147 stable

LOC440434 -0.128509793 10.0677206 -2.140572596 0.038671908 0.335939053 -4.789832558 stable

LOC101929886 /// PGM5-AS1 -0.450578706 6.885367679 -2.140512776 0.038677053 0.335939053 -4.789948914 stable

SNHG1 /// SNORD22 /// SNORD25 /// SNORD26 /// SNORD27 /// SNORD28 /// SNORD29 /// SNORD30 /// SNORD31 -0.195797606 11.09327661 -2.140306572 0.038694792 0.335969067 -4.790349983 stable

B4GALT3 0.104110026 9.236807074 2.1393828 0.038774351 0.336535611 -4.792146358 stable

TANC2 0.15746825 3.526932525 2.139080874 0.038800385 0.336637352 -4.792733354 stable

PRDM2 0.123438851 9.082015285 2.138349656 0.0388635 0.337060616 -4.794154697 stable

LEMD3 0.081483624 10.9405297 2.13805385 0.038889059 0.337100149 -4.794729577 stable

LINC01082 -0.01957768 2.744201895 -2.137777544 0.038912945 0.337100149 -4.795266503 stable

IFT88 -0.150584777 8.583712246 -2.137507637 0.038936291 0.337100149 -4.795790941 stable

EIF3H -0.087642123 13.6129075 -2.137486213 0.038938145 0.337100149 -4.795832567 stable

HOXC8 -0.07916913 3.88783322 -2.137468036 0.038939718 0.337100149 -4.795867885 stable

C14orf2 -0.100138875 10.65408178 -2.135928364 0.039073141 0.338037284 -4.798858481 stable

PDIA3 0.111051092 13.21464893 2.135726667 0.03909065 0.338037284 -4.799250122 stable

SUCNR1 -0.659812321 5.12406976 -2.135721654 0.039091085 0.338037284 -4.799259857 stable

RBM3 -0.171342878 11.64812965 -2.135401591 0.039118883 0.33815334 -4.799881273 stable

CALD1 -0.369944527 5.502063162 -2.13482003 0.039169436 0.338451872 -4.801010213 stable

DYNLT1 0.152898743 12.48675906 2.13467334 0.039182197 0.338451872 -4.801294934 stable

BTAF1 -0.109673211 11.07211003 -2.134353804 0.039210005 0.338567746 -4.801915088 stable

POLR3C -0.122578433 8.565631678 -2.134113359 0.039230943 0.338597211 -4.802381695 stable

LYRM2 -0.112959355 9.645534598 -2.133984022 0.039242209 0.338597211 -4.802632669 stable

IL37 0.017155453 3.184404274 2.133608712 0.039274918 0.338755167 -4.803360876 stable

NEK8 -0.17483772 4.573627779 -2.133103615 0.039318975 0.338981567 -4.804340747 stable

ARHGAP11B /// LOC100288637 -0.106078712 2.830400185 -2.132977418 0.03932999 0.338981567 -4.804585538 stable

BARX1 -0.040883381 3.110140972 -2.132482954 0.039373173 0.339229454 -4.80554456 stable

BLOC1S2 -0.121940238 11.35645824 -2.132214856 0.039396605 0.339307044 -4.806064467 stable

BTN3A2 /// BTN3A3 0.148331462 13.19470877 2.131869265 0.039426827 0.339443044 -4.806734578 stable

TMEM106C -0.267889511 8.51162154 -2.131329727 0.039474051 0.339456909 -4.807780585 stable

DCAF12 0.235098296 10.48782446 2.131109648 0.039493328 0.339456909 -4.808207195 stable

BPHL -0.129643992 5.713701309 -2.130943295 0.039507905 0.339456909 -4.808529636 stable

BLOC1S6 -0.200694214 11.57585767 -2.130893908 0.039512233 0.339456909 -4.808625359 stable

CANT1 0.227078488 7.501478651 2.130689604 0.039530144 0.339456909 -4.809021328 stable

LOC613266 -0.067416414 2.598093576 -2.130439467 0.039552082 0.339456909 -4.809506085 stable

ARHGAP6 -0.427525157 9.255109716 -2.130432336 0.039552707 0.339456909 -4.809519903 stable

YIF1B -0.191598914 9.498650784 -2.130380171 0.039557284 0.339456909 -4.809620993 stable

ALOX5AP 0.145318339 12.20525545 2.130328828 0.039561789 0.339456909 -4.809720485 stable

PRR14L 0.020343888 2.996417674 2.130111687 0.039580846 0.339456909 -4.810141244 stable

TTC7B -0.433172424 8.256745654 -2.130039417 0.03958719 0.339456909 -4.810281276 stable

MAL -0.287905633 11.30457481 -2.129486486 0.039635761 0.33974207 -4.81135252 stable

CCDC147 0.010692568 2.673414659 2.129332075 0.039649334 0.33974207 -4.811651636 stable

NOL10 0.023599284 3.583006902 2.128927274 0.039684936 0.339923303 -4.81243571 stable

POLE2 0.241920974 4.453288292 2.128658735 0.03970857 0.339990302 -4.812955789 stable

SUPT5H -0.088088304 9.801840623 -2.128509974 0.039721668 0.339990302 -4.813243872 stable

DCAF8 0.212007609 5.048075076 2.127356284 0.039823373 0.340736836 -4.815477509 stable

PNN -0.155705994 7.563752222 -2.126884441 0.039865036 0.340969273 -4.816390761 stable

SLC18A2 -0.29403122 5.089667524 -2.126243394 0.0399217 0.340995424 -4.81763125 stable

CSTF3 0.125099256 8.833863753 2.126224729 0.03992335 0.340995424 -4.817667364 stable

FBXO16 /// ZNF395 -0.210384876 8.311443248 -2.126164803 0.039928651 0.340995424 -4.81778331 stable

PLEKHF2 -0.165933847 11.22760019 -2.12611236 0.039933291 0.340995424 -4.817884776 stable

ALS2CR12 -0.053692431 3.670246534 -2.126029969 0.03994058 0.340995424 -4.818044182 stable

HLTF 0.14162315 9.309704975 2.12575909 0.039964556 0.341068899 -4.818568229 stable

ZFAND5 0.142794689 13.07062505 2.125605132 0.039978188 0.341068899 -4.818866056 stable

GNE 0.088614773 8.498784961 2.125092434 0.040023614 0.341332643 -4.81985773 stable

ANAPC11 0.013017118 2.21431031 2.1244856 0.04007744 0.341512487 -4.821031241 stable

TMEM158 -0.591358544 9.357895124 -2.124126625 0.040109311 0.341512487 -4.821725311 stable

RND2 -0.010718646 2.81224219 -2.123904835 0.040129014 0.341512487 -4.822154091 stable

THNSL2 -0.022667356 2.594036289 -2.123894245 0.040129955 0.341512487 -4.822174562 stable

GPR116 0.006408609 2.332753495 2.123881963 0.040131046 0.341512487 -4.822198306 stable

MAST2 0.133223738 7.387477469 2.123764734 0.040141464 0.341512487 -4.822424924 stable

DHRS4-AS1 0.078672866 4.546852102 2.123709889 0.040146338 0.341512487 -4.822530944 stable

AGO2 /// CASC7 /// CASC7 /// DQ574852 0.143041108 6.843806849 2.123143078 0.040196748 0.341817684 -4.823626502 stable

IGH /// IGHA1 /// IGHA2 /// IGHG1 /// IGHG2 /// IGHG3 /// IGHM /// IGHV4-31 /// LOC102725526 -0.480613034 10.30894895 -2.122884294 0.040219782 0.34188995 -4.824126614 stable

SLBP 0.061847051 11.53466688 2.122431818 0.040260084 0.342091291 -4.825000931 stable

EIF5B -0.104936104 10.83812601 -2.122291871 0.040272556 0.342091291 -4.82527132 stable

AX746968 -0.031894563 2.888081532 -2.121921462 0.040305583 0.342248241 -4.825986913 stable

R3HDM4 -0.179965494 12.17279096 -2.121176156 0.040372111 0.34254865 -4.827426468 stable

SP4 0.400827043 5.027313968 2.120898669 0.040396905 0.34254865 -4.827962331 stable

LOC101929473 0.004637342 2.415230202 2.120895164 0.040397218 0.34254865 -4.8279691 stable

GCOM1 /// MYZAP /// POLR2M -0.099983895 9.372920509 -2.120872812 0.040399216 0.34254865 -4.828012262 stable

COX6CP2 /// COX6CP2 0.006497622 2.592462259 2.119746208 0.040500022 0.343157575 -4.83018729 stable

DCAF12L2 0.007234237 2.847051041 2.119744114 0.04050021 0.343157575 -4.830191331 stable

SETMAR -0.098127534 3.142167467 -2.119544687 0.040518077 0.343169374 -4.830576251 stable

GORASP1 -0.183410614 9.132541585 -2.119402929 0.040530782 0.343169374 -4.830849844 stable

RAD51-AS1 -0.179043074 7.720661091 -2.119076572 0.040560045 0.343293562 -4.83147966 stable

LINC00173 -0.14122376 2.677672241 -2.118638595 0.040599345 0.343502586 -4.832324762 stable

LOC100506319 -0.048700892 2.859173321 -2.118036534 0.040653423 0.343800863 -4.833486251 stable

MIR4723 /// TMEM199 0.141964645 8.629570088 2.117862618 0.040669057 0.343800863 -4.833821718 stable

NPPA -0.09804468 6.08958161 -2.117442947 0.040706803 0.343800863 -4.834631135 stable

MTERF1 0.149701704 6.98270211 2.117390988 0.040711478 0.343800863 -4.83473134 stable

SLC25A17 0.138796574 8.212068009 2.117341104 0.040715967 0.343800863 -4.83482754 stable

BAZ2A -0.092707046 9.955014317 -2.117270752 0.040722299 0.343800863 -4.834963211 stable

LOC100131510 -0.201997557 5.861682889 -2.117084525 0.040739065 0.343818994 -4.835322322 stable

ZNF396 0.018345371 3.279870951 2.11652179 0.040789762 0.344123382 -4.836407321 stable

LINC00216 -0.050998835 2.224458142 -2.116226681 0.040816371 0.344224401 -4.836976223 stable

LOC101929036 /// PAH 0.021429956 2.95542901 2.115838867 0.040851362 0.344291881 -4.837723747 stable

ATG4B 0.135867604 7.436339688 2.115813545 0.040853647 0.344291881 -4.837772552 stable

SOX13 0.009827445 2.333143092 2.115272202 0.040902537 0.344580437 -4.838815815 stable

CSTF2 0.184267586 6.867030108 2.115104309 0.04091771 0.344584844 -4.83913933 stable

TTLL11-IT1 0.008374502 2.367456792 2.114925239 0.040933899 0.344595919 -4.839484362 stable

SREK1 -0.21397802 5.328718995 -2.114765705 0.040948327 0.344595919 -4.839791731 stable

RP11-27I1.6 -0.145393603 3.514746897 -2.114559514 0.04096698 0.344619108 -4.840188966 stable

CMPK1 0.005230225 2.390006049 2.114183231 0.041001041 0.344619108 -4.840913813 stable

LOC101930288 /// LOC101930531 0.014614601 3.221124214 2.114147886 0.041004241 0.344619108 -4.840981893 stable

PGBD5 0.006132436 2.30824901 2.114056049 0.041012559 0.344619108 -4.841158784 stable

FAM222A 0.009807673 2.476061816 2.113859845 0.041030333 0.344619108 -4.841536678 stable

ARFGAP3 0.126714295 10.70442731 2.113764292 0.041038992 0.344619108 -4.841720708 stable

SLC6A4 -0.344053909 4.143572286 -2.113221567 0.041088202 0.344831888 -4.842765831 stable

NRG1-IT1 0.007443768 2.529125785 2.112928343 0.041114811 0.344831888 -4.843330405 stable

ATP11B 0.11482808 7.083588039 2.112888596 0.041118419 0.344831888 -4.843406928 stable

RAI2 0.008314145 2.929838355 2.112838436 0.041122973 0.344831888 -4.843503499 stable

ATP6AP1L 0.009898843 2.700744466 2.112478958 0.041155621 0.344937605 -4.844195528 stable

CETN3 0.155100306 9.841314585 2.112376717 0.04116491 0.344937605 -4.844392335 stable

TMEM186 0.177890504 8.159350728 2.111905954 0.041207708 0.345173255 -4.845298423 stable

ASCC2 -0.10345209 9.644481149 -2.109228073 0.041451904 0.346898338 -4.850449563 stable

AADAT -0.013713212 3.175181351 -2.109195726 0.041454861 0.346898338 -4.850511754 stable

LOC100996583 -0.269067254 5.698363389 -2.109162507 0.041457898 0.346898338 -4.85057562 stable

MAP4K1 -0.133932437 10.11036665 -2.1089597 0.041476446 0.346930119 -4.850965516 stable

ATG14 -0.0876028 9.429942382 -2.107256329 0.041632519 0.348111799 -4.854239078 stable

CTD-3092A11.2 -0.228996814 11.82943475 -2.105924909 0.041754871 0.348643214 -4.856796359 stable

MIR6789 /// PLEKHJ1 -0.116558668 8.721926596 -2.105804536 0.041765949 0.348643214 -4.8570275 stable

AC128677.4 /// CH17-132F21.1 /// IGKV1OR-1 /// IGKV1OR-1 /// IGKV1OR10-1 /// IGKV1OR10-1 /// IGKV1OR2-2 -0.433063787 9.150012513 -2.105789415 0.041767341 0.348643214 -4.857056533 stable

YAF2 0.125142182 8.134916981 2.105767683 0.041769341 0.348643214 -4.857098261 stable

CRYL1 -0.183680979 8.870633026 -2.105672004 0.041778148 0.348643214 -4.857281974 stable

SLC9A6 0.094077444 10.55958707 2.105476481 0.041796151 0.348643214 -4.857657374 stable

GPATCH8 0.092833507 10.34817578 2.10543651 0.041799833 0.348643214 -4.857734115 stable

LOC100132686 -0.046953228 2.615624809 -2.105019834 0.041838225 0.348839732 -4.858534022 stable

PIGO 0.087321383 8.2542988 2.104760835 0.041862104 0.34891515 -4.859031167 stable

GRIPAP1 -0.11117225 8.716150563 -2.104195004 0.041914315 0.349226569 -4.860117106 stable

CCT6A 0.111798216 9.689698394 2.103737839 0.041956541 0.349294354 -4.860994323 stable

TMEM192 -0.113181758 8.879028248 -2.103448685 0.041983268 0.349294354 -4.861549081 stable

SNX22 -0.221280565 4.355358096 -2.103424951 0.041985462 0.349294354 -4.861594612 stable

CIDECP 0.044459631 3.440237976 2.103290637 0.041997883 0.349294354 -4.861852277 stable

PCNXL4 0.298065978 4.666413794 2.103150952 0.042010803 0.349294354 -4.862120233 stable

NFKB1 -0.093734805 10.85307863 -2.103142852 0.042011552 0.349294354 -4.862135771 stable

LINC01019 0.004886004 2.452985887 2.102795419 0.042043705 0.349438156 -4.862802181 stable

KIR3DX1 0.109519952 3.93576994 2.10185227 0.042131096 0.350036351 -4.864610796 stable

NEFM 0.005142416 2.740761241 2.101657252 0.042149186 0.350036351 -4.864984689 stable

CENPA -0.046146253 2.488613915 -2.101537214 0.042160324 0.350036351 -4.865214815 stable

CHAC2 0.337437142 6.988431383 2.101331176 0.042179448 0.350071559 -4.865609788 stable

SLCO4A1 -0.112382415 2.874799342 -2.100734431 0.04223488 0.350407976 -4.866753567 stable

LOC100509303 0.012617851 2.990599592 2.10011035 0.04229292 0.350682739 -4.867949468 stable

TDRD1 0.005062422 2.609604056 2.099972156 0.042305781 0.350682739 -4.868214246 stable

RP4-714D9.5 0.147085558 4.429605458 2.099876757 0.042314662 0.350682739 -4.868397021 stable

NEURL3 -0.124820335 4.076532815 -2.099640486 0.042336663 0.350682739 -4.868849662 stable

TRPT1 -0.128559977 8.417707049 -2.09957735 0.042342544 0.350682739 -4.86897061 stable

FAM195A -0.170038615 8.017601145 -2.09838762 0.042453498 0.351358321 -4.871249199 stable

ZNF653 0.087836914 4.36752503 2.09812875 0.042477674 0.351358321 -4.871744856 stable

NT5DC1 -0.081534845 9.198122399 -2.098063557 0.042483764 0.351358321 -4.871869671 stable

ERCC4 0.291764192 5.068058885 2.097858398 0.042502935 0.351358321 -4.872262445 stable

AKAP2 /// PALM2 /// PALM2-AKAP2 0.00879992 2.891594092 2.097819105 0.042506607 0.351358321 -4.872337666 stable

ZNF236 0.116694903 5.865192019 2.097715715 0.042516272 0.351358321 -4.87253559 stable

RAB43 0.144390059 6.677069857 2.097582981 0.042528682 0.351358321 -4.872789674 stable

LOC102725016 /// PPP2R3B -0.036314378 3.281127622 -2.097043326 0.042579173 0.35159411 -4.873822578 stable

HCCS 0.314794583 8.860950799 2.096958452 0.042587119 0.35159411 -4.873985008 stable

SPIB -0.495495761 10.06980531 -2.096489347 0.042631058 0.351833378 -4.874882678 stable

RACGAP1P 0.010244429 2.878682449 2.096175142 0.042660511 0.35195296 -4.875483844 stable

IQCB1 -0.134067395 8.738661414 -2.095563521 0.042717895 0.352302808 -4.876653851 stable

PALB2 0.189560513 7.977043079 2.095313728 0.04274135 0.352343015 -4.877131617 stable

GLI1 -0.077325338 3.06505821 -2.095192583 0.04275273 0.352343015 -4.877363308 stable

FBXO31 0.135177754 5.710749484 2.094706402 0.042798426 0.352596066 -4.878293029 stable

POLR3D 0.15022198 7.52380566 2.094071695 0.042858145 0.352893483 -4.879506517 stable

MTOR -0.055260136 7.974097756 -2.094003854 0.042864533 0.352893483 -4.879636203 stable

KCNMB3 -0.043228 2.762234005 -2.093607849 0.042901835 0.353077 -4.880393153 stable

CDH10 0.007397277 3.068222508 2.092813277 0.042976767 0.353569968 -4.881911607 stable

CTD-2555O16.4 /// MTHFD1 -0.360214884 5.903072917 -2.092526145 0.043003873 0.353669266 -4.882460211 stable

CCL1 0.007119936 2.622151748 2.092174036 0.043037134 0.353773901 -4.883132883 stable

PIK3IP1 -0.388839454 9.29414071 -2.092040837 0.043049722 0.353773901 -4.883387324 stable

FBXW7 -0.097262147 9.822079477 -2.091913928 0.043061718 0.353773901 -4.883629737 stable

ZNF331 -0.221500464 8.902773726 -2.091659348 0.043085792 0.353787847 -4.884115985 stable

CYP2B7P 0.021807783 3.004654744 2.091577883 0.043093499 0.353787847 -4.884271573 stable

ZNF75D 0.114640445 8.288876308 2.09056489 0.043189423 0.354451649 -4.886205863 stable

ZMYM1 0.129848833 7.063798262 2.090289425 0.043215541 0.354542286 -4.886731729 stable

KIR2DL4 1.178971197 4.493361667 3.42052055 0.001487971 0.047430832 -1.798120612 up

C16orf74 -0.296374207 7.084143101 -2.089076441 0.043330711 0.35512351 -4.889046673 stable

JUND -0.140969251 9.69535412 -2.089066237 0.043331681 0.35512351 -4.889066143 stable

SPTA1 0.006950594 2.529203945 2.088788749 0.043358067 0.355215982 -4.88959557 stable

NPAS1 -0.076758412 5.539952206 -2.088011872 0.043432012 0.355521613 -4.891077492 stable

PARN -0.080075047 9.551815943 -2.08780792 0.043451443 0.355521613 -4.891466467 stable

MISP -0.060173839 3.77834673 -2.087780522 0.043454054 0.355521613 -4.891518718 stable

WFDC21P -0.178933255 3.353598546 -2.087673707 0.043464234 0.355521613 -4.891722419 stable

RFK 0.170059362 10.18850074 2.087603275 0.043470948 0.355521613 -4.891856732 stable

KCNK17 -0.055061797 2.61629223 -2.086946533 0.043533594 0.355910205 -4.893108955 stable

MRPS15 -0.085804158 9.548282135 -2.086784669 0.043549046 0.355912825 -4.893417537 stable

FOXP1-IT1 -0.227869381 8.090018433 -2.086507764 0.043575492 0.356005259 -4.893945391 stable

VAC14 -0.13195969 6.265434783 -2.086208993 0.043604042 0.356114814 -4.894514867 stable

FLJ31713 0.009014531 2.709030359 2.085374026 0.043683916 0.356643316 -4.896106016 stable

KIAA1586 0.118484965 5.635591587 2.084312811 0.043785619 0.357349601 -4.898127582 stable

C17orf85 0.104923669 7.525540262 2.084022195 0.043813507 0.357392558 -4.898681048 stable

SYAP1 0.116646382 10.07734889 2.083941306 0.043821272 0.357392558 -4.898835086 stable

EHD3 -0.41818683 9.703129996 -2.083723962 0.043842142 0.357438829 -4.899248958 stable

TMED6 -0.084671153 2.421261758 -2.083372271 0.043875931 0.357514035 -4.899918582 stable

INCENP 0.062590695 2.506005508 2.083277086 0.04388508 0.357514035 -4.900099799 stable

CMIP -0.188216274 11.59146927 -2.083092241 0.043902851 0.357514035 -4.900451697 stable

IKBIP 0.284488636 8.560142537 2.082987454 0.043912928 0.357514035 -4.900651174 stable

LTBP1 -0.432700938 7.732190011 -2.08283737 0.043927365 0.357514035 -4.900936866 stable

SF1 -0.316420914 6.322798496 -2.082372895 0.043972071 0.357754092 -4.901820915 stable

LINGO2 0.212027403 2.756879654 2.081628011 0.044043849 0.358175337 -4.903238343 stable

LRIG2 -0.123344583 7.931399111 -2.081201245 0.044085019 0.358175337 -4.904050247 stable

KCTD11 0.212604985 7.309897805 2.081149846 0.04408998 0.358175337 -4.904148023 stable

POLB 0.140704403 10.19248242 2.081117322 0.044093119 0.358175337 -4.904209892 stable

PSMD11 -0.162286104 8.576957704 -2.081041542 0.044100434 0.358175337 -4.904354042 stable

CEP57L1 0.059855906 4.217531754 2.080888467 0.044115214 0.358175337 -4.90464521 stable

SP110 -0.106969224 11.25856653 -2.080551741 0.044147741 0.358315744 -4.905285648 stable

NRROS 0.337532205 9.815248809 2.079988006 0.044202244 0.358634354 -4.906357658 stable

RABEP1 0.160015219 8.017174514 2.078780586 0.044319179 0.359459107 -4.90865294 stable

COL6A2 0.363285655 8.830020351 2.078018896 0.044393086 0.359874735 -4.91010035 stable

CCDC28A 0.122255018 10.65121803 2.07793715 0.044401024 0.359874735 -4.910255665 stable

SEC23B 0.191595969 8.061981135 2.077634815 0.044430394 0.359988733 -4.910830046 stable

VDAC3 0.185440855 3.679417368 2.077378636 0.044455293 0.360066445 -4.911316686 stable

KPNA2 0.142914853 10.605806 2.077084652 0.044483882 0.360173976 -4.911875082 stable

SNRPN /// SNURF -0.369072066 9.140615313 -2.076647605 0.044526414 0.360378868 -4.912705097 stable

PBDC1 0.106637583 9.595389244 2.076509806 0.044539831 0.360378868 -4.912966768 stable

BEND5 -0.169039586 2.786143302 -2.076092736 0.044580463 0.360583583 -4.913758673 stable

LMNA -0.394434709 7.033079097 -2.075873385 0.044601845 0.360591343 -4.914175111 stable

FOSL1 -0.079054151 2.375635417 -2.075728069 0.044616015 0.360591343 -4.914450972 stable

LAX1 0.183781851 9.272732421 2.075611208 0.044627414 0.360591343 -4.914672808 stable

LGALS4 -0.039741924 2.311997136 -2.075453285 0.044642821 0.360591965 -4.914972572 stable

EXOC1 0.095489087 10.85075287 2.074304063 0.044755085 0.361374651 -4.917153444 stable

THBD 0.568156436 5.881737183 2.073896193 0.044794988 0.361572722 -4.917927224 stable

S100A13 -0.209936651 6.271360467 -2.073146422 0.044868421 0.361889252 -4.919349318 stable

ULK2 0.323284755 6.769119807 2.073070573 0.044875856 0.361889252 -4.919493159 stable

RP11-218C14.8 0.010456381 2.460131771 2.072933138 0.04488933 0.361889252 -4.91975378 stable

BC039681 /// RP5-1157M23.2 -0.173058268 4.771781586 -2.072867706 0.044895746 0.361889252 -4.919877854 stable

PLEKHO1 -0.124614078 12.27118709 -2.071907084 0.044990034 0.362495157 -4.921699074 stable

BIRC6 0.12037902 11.40823065 2.07178798 0.045001737 0.362495157 -4.921924835 stable

FANCL 0.192570529 7.989879347 2.071573439 0.045022824 0.362540853 -4.922331466 stable

KIAA1143 0.107690149 10.60702164 2.071320819 0.045047664 0.362616052 -4.92281023 stable

TMEM19 0.232727499 3.746762464 2.071164959 0.045062996 0.362616052 -4.923105591 stable

HARS2 0.127625551 8.890456038 2.070927075 0.045086405 0.362634241 -4.923556355 stable

C10orf82 -0.171902559 3.447010712 -2.070772013 0.045101669 0.362634241 -4.92385016 stable

COLQ 0.496362415 6.781486752 2.070595311 0.04511907 0.362634241 -4.924184945 stable

C3orf58 0.238667933 11.0565284 2.070515555 0.045126926 0.362634241 -4.924336045 stable

NASP /// NASP 0.267009116 3.096842376 2.070284714 0.04514967 0.362693098 -4.924773356 stable

EARS2 -0.072135139 3.754345714 -2.07001038 0.045176713 0.362775593 -4.92529301 stable

SCN3A -0.854017114 5.552302335 -2.069867663 0.045190786 0.362775593 -4.925563328 stable

GP9 -0.383016398 8.669332915 -2.069508867 0.045226186 0.362827573 -4.926242854 stable

EGLN2 /// RAB4B-EGLN2 0.092463069 10.8176131 2.069341773 0.04524268 0.362827573 -4.926559282 stable

3-Mar -0.370380051 6.457802171 -2.069294258 0.045247371 0.362827573 -4.926649258 stable

LOC653160 0.142861213 5.539600625 2.069091697 0.045267375 0.362827573 -4.927032815 stable

MED13 0.068389222 9.844076306 2.068873074 0.045288974 0.362827573 -4.927446754 stable

FNDC3A 0.093848458 10.11896323 2.068864557 0.045289816 0.362827573 -4.927462879 stable

CSNK2A1 /// NFE2L2 0.087597602 7.968299581 2.068447877 0.045331007 0.363033918 -4.928251716 stable

BAG2 0.216181679 7.312855639 2.067752537 0.045399819 0.363258349 -4.929567815 stable

NOP9 0.18493935 5.551670719 2.067610495 0.045413887 0.363258349 -4.92983662 stable

ABHD6 0.399566666 7.186216678 2.06754564 0.045420312 0.363258349 -4.929959351 stable

SFPQ -0.082280906 10.19377863 -2.067540637 0.045420807 0.363258349 -4.929968818 stable

CYTIP 0.17048436 12.33805919 2.066218005 0.045552002 0.364183769 -4.932471039 stable

LOC100506098 -0.087545484 2.462380569 -2.06603061 0.045570617 0.364208798 -4.932825458 stable

TAF10 -0.098010904 12.53475775 -2.065648983 0.045608547 0.364383067 -4.933547149 stable

AGPAT6 -0.112713657 9.561847815 -2.06540961 0.045632352 0.364383067 -4.93399977 stable

SCG3 0.008910306 2.985771736 2.065039002 0.04566923 0.364383067 -4.934700455 stable

GPATCH2 -0.118046777 7.096898072 -2.064964121 0.045676685 0.364383067 -4.934842016 stable

PSPC1 0.102679446 9.333350961 2.064739811 0.045699021 0.364383067 -4.935266044 stable

FAM46C -0.353578259 9.076397318 -2.06472805 0.045700192 0.364383067 -4.935288277 stable

SCAND1 -0.152668784 10.56726886 -2.064721294 0.045700865 0.364383067 -4.935301048 stable

VAX2 -0.026457795 3.280276178 -2.064528532 0.045720068 0.364412647 -4.935665406 stable

IGKV1OR2-108 /// IGKV1OR2-108 -0.456790103 10.39925385 -2.064249757 0.045747852 0.36451058 -4.936192299 stable

TRH 0.0075504 2.489245758 2.063614126 0.045811258 0.364865146 -4.937393446 stable

FAM124A -0.087763745 3.284112146 -2.063492723 0.045823377 0.364865146 -4.937622825 stable

PTGES3 0.063644121 13.58479836 2.062893083 0.045883276 0.365218457 -4.938755636 stable

SCARNA15 -0.106774494 7.979374025 -2.062154749 0.045957125 0.365598847 -4.9401501 stable

LOC101926996 0.015557345 2.741700253 2.062053497 0.04596726 0.365598847 -4.940341299 stable

GSTA1 -0.114588354 3.646348869 -2.06194926 0.045977696 0.365598847 -4.940538127 stable

UBLCP1 0.11275155 10.40422053 2.061785859 0.04599406 0.365605369 -4.940846659 stable

NHP2P2 /// NHP2P2 -0.048400125 8.369765144 -2.061377534 0.046034974 0.365806969 -4.941617564 stable

HLA-B 0.050049225 15.35180469 2.060661879 0.04610676 0.366058567 -4.942968405 stable

ZNF141 0.14961074 6.040452001 2.060653483 0.046107602 0.366058567 -4.942984252 stable

PADI1 -0.061524719 5.55408276 -2.060596468 0.046113326 0.366058567 -4.943091854 stable

PRTFDC1 -0.37424952 6.141026392 -2.060362738 0.046136795 0.366081811 -4.94353294 stable

IFI16 0.111157914 12.47591636 2.060257332 0.046147382 0.366081811 -4.943731845 stable

BNIP3L -0.103350269 12.43204213 -2.059617108 0.046211734 0.366468708 -4.944939798 stable

NDUFV2 -0.096625493 10.69519132 -2.059376522 0.046235936 0.36653706 -4.945393651 stable

SCNN1B 0.004412225 2.206710017 2.058979376 0.046275913 0.366730373 -4.94614275 stable

KSR2 0.009070338 2.965544237 2.057242862 0.046451065 0.367994445 -4.94941682 stable

EIF4ENIF1 0.09167993 9.704558901 2.057029852 0.04647259 0.368041009 -4.949818282 stable

MIR31HG 0.005469787 2.440435075 2.056294354 0.046546981 0.368363247 -4.951204227 stable

CSAG2 /// CSAG3 -0.069934631 2.870209519 -2.056099167 0.04656674 0.368363247 -4.951571964 stable

IFNL2 /// IFNL3 0.006786375 2.78217551 2.056075583 0.046569128 0.368363247 -4.951616395 stable

SLC25A32 -0.105493975 9.267580875 -2.055917723 0.046585114 0.368363247 -4.951913783 stable

LL22NC03-N27C7.1 /// ZNF70 0.130658865 3.925071277 2.055853848 0.046591584 0.368363247 -4.952034111 stable

CYSLTR2 0.351905046 6.020407441 2.055673134 0.046609894 0.368384176 -4.952374523 stable

VSX1 0.01314649 2.659018153 2.055163375 0.046661574 0.368668755 -4.953334628 stable

NME1-NME2 /// NME2 -0.071802782 12.50994068 -2.054951087 0.046683111 0.368715063 -4.953734407 stable

OIP5-AS1 0.08543565 12.20634309 2.054397545 0.04673931 0.368847347 -4.954776671 stable

TVP23B 0.154594095 8.856849907 2.054174683 0.046761953 0.368847347 -4.955196237 stable

LOC647070 -0.102526679 5.216488221 -2.053997112 0.046780001 0.368847347 -4.955530508 stable

RP11-548M13.1 -0.103322156 4.04795751 -2.053784756 0.046801593 0.368847347 -4.955930232 stable

SLC5A6 -0.116469009 7.441383584 -2.053702457 0.046809963 0.368847347 -4.956085137 stable

PATE2 0.005889732 2.459779618 2.053633378 0.04681699 0.368847347 -4.956215155 stable

TNFAIP8L1 -0.09990961 8.317955377 -2.05356798 0.046823643 0.368847347 -4.956338242 stable

TASP1 0.149099288 8.056039857 2.053551568 0.046825313 0.368847347 -4.95636913 stable

HNRNPA1 -0.108919953 12.53587948 -2.053321444 0.046848731 0.36890827 -4.956802222 stable

CABP5 -0.526482088 7.992004248 -2.052957609 0.046885778 0.369012483 -4.95748688 stable

MCTP2 0.009911435 2.044065462 2.052713907 0.046910606 0.369012483 -4.957945418 stable

ZNF284 0.134070177 7.311834915 2.052603192 0.04692189 0.369012483 -4.958153719 stable

MPPE1 -0.084585252 8.787697064 -2.052449979 0.046937508 0.369012483 -4.958441963 stable

OVGP1 -0.370170752 6.031129567 -2.052172148 0.046965842 0.369012483 -4.958964609 stable

HIST1H2AD /// HIST1H3A /// HIST1H3B /// HIST1H3C /// HIST1H3D /// HIST1H3E /// HIST1H3F /// HIST1H3G /// HIST1H3H /// HIST1H3I /// HIST1H3J -0.404633041 4.68655478 -2.052156997 0.046967388 0.369012483 -4.958993109 stable

SEC23A 0.091651656 8.969312216 2.05200499 0.046982897 0.369012483 -4.959279034 stable

MKRN1 -0.073941128 12.12546805 -2.051960129 0.046987475 0.369012483 -4.959363414 stable

R3HDM2 -0.070628181 10.42964838 -2.051739025 0.047010043 0.369066496 -4.959779274 stable

DDR1 /// MIR4640 -0.168333699 6.786745739 -2.051428977 0.047041706 0.369191849 -4.960362361 stable

UBA1 0.017937236 2.217419372 2.05105058 0.047080375 0.369372078 -4.96107389 stable

FAM150B 0.259230129 3.378979017 2.05053448 0.04713316 0.369662903 -4.962044184 stable

LOC399491 -0.187133816 11.38123014 -2.050315717 0.04715555 0.369715228 -4.962455411 stable

SGCE -0.28720252 3.696963294 -2.049843909 0.04720387 0.369907524 -4.963342187 stable

CSDC2 0.009128037 2.405169196 2.049718341 0.047216737 0.369907524 -4.963578168 stable

LOC101927020 -0.042288939 3.680410385 -2.049496601 0.047239467 0.369907524 -4.963994857 stable

GATA1 -0.142719077 4.28761942 -2.049395366 0.047249848 0.369907524 -4.964185084 stable

HECTD1 0.146962204 8.603625359 2.048985195 0.047291926 0.369907524 -4.964955745 stable

ZRANB2 -0.140629458 11.14545268 -2.04896873 0.047293616 0.369907524 -4.964986677 stable

MAGEH1 0.157818207 9.274581303 2.048787533 0.047312216 0.369907524 -4.965327081 stable

AP1M2 -0.009912313 2.046751624 -2.048572631 0.047334284 0.369907524 -4.965730775 stable

RRAS2 0.270270106 8.920777735 2.048545783 0.047337041 0.369907524 -4.965781206 stable

RCSD1 -0.097791339 12.8741657 -2.048542844 0.047337343 0.369907524 -4.965786727 stable

LINC00899 -0.072273097 2.891405332 -2.0481753 0.047375108 0.370079681 -4.96647707 stable

TSNAXIP1 -0.017239278 2.446206288 -2.047882621 0.0474052 0.370171808 -4.967026727 stable

TRAPPC3L -0.331078318 5.119358953 -2.047751701 0.047418666 0.370171808 -4.967272577 stable

SAMD9L 0.178486212 11.19568466 2.047600703 0.047434201 0.370171808 -4.967556115 stable

HPS6 0.221094947 8.6914624 2.047448607 0.047449853 0.370171808 -4.967841697 stable

HCG18 0.160528795 5.020441961 2.046329563 0.047565156 0.370827426 -4.969942344 stable

CAP1 0.053892781 14.11948221 2.046326962 0.047565424 0.370827426 -4.969947226 stable

TTF1 -0.124382339 9.592725134 -2.045931762 0.047606203 0.371022365 -4.970688866 stable

TRIM28 -0.088905804 12.03407027 -2.04533351 0.047667992 0.371380864 -4.971811338 stable

TTBK1 -0.082728053 4.259169909 -2.044944943 0.047708161 0.37142786 -4.972540248 stable

PTPN2 0.113602957 4.109349765 2.044841583 0.047718851 0.37142786 -4.972734122 stable

PDCD4-AS1 -0.259202436 8.334780262 -2.044752062 0.047728112 0.37142786 -4.972902032 stable

PRKACB 0.155628097 12.92617056 2.044574147 0.047746521 0.37142786 -4.973235718 stable

PRO2949 0.012634516 3.458057366 2.044511737 0.04775298 0.37142786 -4.973352766 stable

CCDC107 0.111965891 8.720237198 2.044282551 0.047776706 0.371489558 -4.973782568 stable

HDAC8 0.090284593 7.431803185 2.043970869 0.04780899 0.371617728 -4.974367017 stable

DDHD1 0.168161383 7.837477185 2.043284065 0.047880194 0.371994667 -4.975654621 stable

MIR600 /// MIR600HG -0.231154409 3.30582697 -2.042979573 0.047911792 0.371994667 -4.976225365 stable

WDHD1 0.037517685 3.262356372 2.042851151 0.047925124 0.371994667 -4.97646606 stable

C17orf67 -0.118303871 5.89609885 -2.042757584 0.047934839 0.371994667 -4.97664142 stable

FBXW11 0.086916013 9.46621559 2.042657269 0.047945258 0.371994667 -4.976829421 stable

RP1-118J21.25 -0.10404237 6.059577113 -2.042588741 0.047952376 0.371994667 -4.976957845 stable

REXO1 -0.079270674 6.932542229 -2.041820131 0.048032276 0.372491648 -4.978398012 stable

ASB16-AS1 -0.098944351 7.68853342 -2.04135174 0.048081024 0.372746795 -4.979275436 stable

LOC100291666 -0.101939004 3.258947487 -2.040874557 0.048130732 0.373009209 -4.980169164 stable

CACNG8 -0.110508721 5.882881786 -2.04044483 0.048175535 0.373074166 -4.980973866 stable

HEXIM2 -0.376810605 7.939605592 -2.040365883 0.04818377 0.373074166 -4.981121686 stable

CDC73 0.106918919 8.798925965 2.040337813 0.048186698 0.373074166 -4.981174242 stable

SLC43A1 0.119789512 6.864638546 2.039037527 0.048322513 0.373946831 -4.983608213 stable

FRG1B -0.263301494 7.762123945 -2.038905644 0.048336307 0.373946831 -4.983855012 stable

LOC389906 /// LOC441528 -0.291151982 6.970318418 -2.0388024 0.048347107 0.373946831 -4.984048208 stable

LOC340184 0.047364333 3.426170258 2.038384722 0.048390824 0.373999327 -4.98482971 stable

ZBTB8A 0.020614558 2.991824843 2.03813851 0.04841661 0.373999327 -4.985290328 stable

BAIAP3 -0.138782769 5.676320355 -2.038092513 0.048421429 0.373999327 -4.985376375 stable

POLR1C 0.169580234 7.760583273 2.037919339 0.048439575 0.373999327 -4.98570032 stable

DPY19L2P4 0.003828246 2.379215776 2.037893735 0.048442258 0.373999327 -4.985748214 stable

NPSA 0.0120495 2.632102311 2.037753147 0.048456994 0.373999327 -4.986011183 stable

RSPH6A -0.11973674 4.011314542 -2.037658083 0.048466961 0.373999327 -4.986188991 stable

RNF208 -0.204824002 3.442287362 -2.037523252 0.0484811 0.373999327 -4.98644117 stable

LRRC38 0.033620825 2.952147528 2.037285039 0.048506089 0.374069413 -4.986886672 stable

KIF2A -0.24967492 10.18853972 -2.036732016 0.048564145 0.374394379 -4.987920768 stable

DDX43 0.248676378 3.535021883 2.036183358 0.048621803 0.374716062 -4.988946477 stable

ECSIT -0.114688671 8.762709422 -2.035670489 0.048675754 0.375008974 -4.989905078 stable

CYB5RL -0.139019457 6.248264382 -2.034938141 0.048752883 0.375276512 -4.99127357 stable

TESK1 0.123644396 8.629486869 2.034915408 0.048755279 0.375276512 -4.991316043 stable

ACAD11 /// NPHP3 /// NPHP3-ACAD11 -0.138090621 8.624991546 -2.03488632 0.048758344 0.375276512 -4.991370389 stable

SSX3 /// SSX5 /// SSX7 -0.010774719 2.346381146 -2.034720873 0.048775785 0.375287946 -4.991679488 stable

EFNB2 0.032321828 2.526159768 2.034433559 0.048806086 0.375398285 -4.992216219 stable

WDR44 0.12514752 9.053516633 2.033981882 0.048853754 0.37539862 -4.993059873 stable

C21orf15 0.169076178 4.69209803 2.033821655 0.048870674 0.37539862 -4.993359113 stable

IGLVIVOR22-2 /// IGLVIVOR22-2 0.012489258 3.107058434 2.033564144 0.048897877 0.37539862 -4.99384 stable

THAP5 0.199460609 9.214544173 2.033531635 0.048901312 0.37539862 -4.993900707 stable

GCG 0.005469374 2.37590264 2.033466005 0.048908248 0.37539862 -4.994023257 stable

TMEM177 0.180500291 6.258876177 2.033369877 0.048918408 0.37539862 -4.994202751 stable

RAB27B -0.286246225 8.02816439 -2.033367344 0.048918675 0.37539862 -4.994207481 stable

LINC01000 -0.239155377 12.00024072 -2.033224172 0.048933811 0.37539862 -4.994474804 stable

CRB3 -0.080902674 7.019531649 -2.032815015 0.048977089 0.375568954 -4.99523868 stable

GYG2 0.005101483 2.617920939 2.032712386 0.048987949 0.375568954 -4.995430263 stable

DDI2 /// RSC1A1 0.211951899 6.974967758 2.032353814 0.049025911 0.375737521 -4.996099569 stable

CMTM5 -0.462523983 9.690122644 -2.032146868 0.049047832 0.375783079 -4.996485808 stable

LOC101930324 /// NSF 0.1176336 10.58613299 2.031687212 0.049096552 0.375941139 -4.997343587 stable

INTS7 0.017354208 2.940400084 2.031650654 0.049100428 0.375941139 -4.997411802 stable

DOK2 0.125527258 11.88283042 2.031459629 0.049120689 0.375946254 -4.997768229 stable

ZDHHC11 -0.086335032 5.979398645 -2.031342998 0.049133063 0.375946254 -4.997985833 stable

PINX1 -0.090931903 8.10960054 -2.030234366 0.04925082 0.376724727 -5.000053763 stable

MRPS26 -0.105004281 9.353031816 -2.029621805 0.04931599 0.377100586 -5.00119598 stable

KERA 0.014636407 3.373727569 2.029366389 0.049343186 0.377185921 -5.001672162 stable

KLHL3 0.023297892 2.971647464 2.028569897 0.049428078 0.377560805 -5.003156784 stable

METTL13 0.137840351 8.374160912 2.028517904 0.049433624 0.377560805 -5.003253679 stable

ADARB1 -0.257824051 9.043955084 -2.028454533 0.049440384 0.377560805 -5.003371778 stable

AK021933 0.008584274 2.932878776 2.02815737 0.049472096 0.377603187 -5.003925528 stable

PRSS3 -0.012558453 2.268462472 -2.028095649 0.049478685 0.377603187 -5.004040535 stable

NDUFAF3 -0.12627972 10.2241804 -2.027951314 0.049494096 0.377603187 -5.004309468 stable

LSM7 -0.115536402 11.90984935 -2.027662935 0.049524899 0.377715677 -5.004846743 stable

MED25 -0.074478794 7.544670222 -2.026918592 0.049604483 0.37816864 -5.006233237 stable

ZNF384 -0.078825354 9.91517531 -2.026740087 0.049623586 0.37816864 -5.006565679 stable

ZBTB7A 0.125672066 7.090980238 2.02664522 0.04963374 0.37816864 -5.006742346 stable

EIF3B -0.08039973 11.0411609 -2.02644257 0.049655438 0.37816864 -5.007119712 stable

PANK3 0.188090285 6.327342007 2.026262585 0.049674715 0.37816864 -5.007454847 stable

UBR3 0.156869436 9.805883099 2.026206186 0.049680758 0.37816864 -5.007559857 stable

RPS16P5 -0.298325992 8.66741575 -2.025950327 0.049708176 0.378191036 -5.008036217 stable

LENG8 -0.261965428 10.58221252 -2.025732782 0.049731499 0.378191036 -5.008441207 stable

LOC101928139 0.006562531 2.517241092 2.025593219 0.049746467 0.378191036 -5.008701004 stable

HIST1H3G -0.105751684 7.104489455 -2.025578784 0.049748015 0.378191036 -5.008727874 stable

AARS2 -0.102657893 6.950148418 -2.025000329 0.049810096 0.378455606 -5.00980451 stable

PRKX /// PRKY 0.116552151 10.27546096 2.024954686 0.049814997 0.378455606 -5.00988945 stable

LOC100131564 -0.350614687 10.91901527 -2.024651085 0.04984761 0.378581092 -5.010454411 stable

NCOA5 0.148821926 9.691791283 2.024099096 0.049906953 0.378909435 -5.011481412 stable

DNAJC9 -0.099866795 10.19757795 -2.022851368 0.050041318 0.379806983 -5.013802037 stable

ERRFI1 0.110099231 2.656667406 2.022638477 0.050064275 0.379858651 -5.014197874 stable

HAUS1 -0.10982021 10.09589517 -2.022059707 0.050126734 0.379877652 -5.015273832 stable

ELOVL7 -0.462727652 9.89243148 -2.022056589 0.05012707 0.379877652 -5.015279628 stable

MMS19 -0.091072553 10.09092867 -2.02187007 0.050147213 0.379877652 -5.015626323 stable

METTL8 -0.159702602 8.996538792 -2.02180861 0.050153852 0.379877652 -5.015740556 stable

YIPF6 0.264795898 5.807337922 2.021787438 0.050156139 0.379877652 -5.015779907 stable

ESRRG 0.006580095 2.85618458 2.021623868 0.050173813 0.379877652 -5.016083914 stable

RP11-96K19.4 0.018403284 3.157793872 2.021568153 0.050179834 0.379877652 -5.01618746 stable

TMEM198B -0.160929532 7.617476091 -2.021352668 0.050203127 0.379931706 -5.016587913 stable

NARS2 -0.06271144 9.310905835 -2.020902383 0.050251831 0.380177973 -5.017424606 stable

C12orf61 0.009161071 2.800473962 2.020553929 0.050289549 0.380340993 -5.018071978 stable

DKFZp434L192 /// LOC100653233 -0.101361153 4.030689293 -2.020374004 0.050309035 0.380366058 -5.018406214 stable

ADAT3 /// SCAMP4 0.24479689 7.10965733 2.020168299 0.05033132 0.38041227 -5.018788311 stable

C2orf68 -0.093379903 9.094854288 -2.018981532 0.050460059 0.381262786 -5.020992121 stable

TRPM6 -0.062957422 3.055544037 -2.018818974 0.050477716 0.381273716 -5.021293908 stable

CCNE1 -0.145351558 6.265694055 -2.018439453 0.050518959 0.381462737 -5.021998407 stable

RP11-173M1.8 0.303785969 7.688492316 2.018175629 0.050547646 0.381556862 -5.022488077 stable

ZFP41 -0.078370344 5.651721099 -2.017976519 0.050569306 0.381597897 -5.022857599 stable

SH3GLB2 -0.120205654 9.118467528 -2.017257992 0.050647537 0.381951362 -5.024190849 stable

ZBTB38 0.167709821 9.434247145 2.017018831 0.0506736 0.381951362 -5.024634536 stable

MRPS36 0.11386145 10.212884 2.016926031 0.050683716 0.381951362 -5.024806684 stable

CGGBP1 -0.066204266 10.96457328 -2.0168691 0.050689923 0.381951362 -5.02491229 stable

SMC6 -0.148396501 9.716933581 -2.016801068 0.050697341 0.381951362 -5.025038487 stable

MYO1C -0.180148254 9.143927136 -2.01600851 0.050783828 0.382480444 -5.026508389 stable

TAF4B -0.203729316 5.882213105 -2.015290561 0.050862285 0.382948723 -5.027839517 stable

ANKS3 -0.118919598 5.58611201 -2.014869822 0.050908312 0.383155409 -5.028619419 stable

ETAA1 0.105744067 7.163733178 2.014603673 0.050937446 0.383155409 -5.029112697 stable

MIRLET7D -0.16382415 8.129169451 -2.014593079 0.050938606 0.383155409 -5.029132331 stable

C2orf42 0.175720619 8.230633556 2.014256507 0.050975471 0.38318776 -5.029756052 stable

EID2 0.118568641 9.931069015 2.014256335 0.05097549 0.38318776 -5.029756372 stable

CHAMP1 0.171706191 9.741389998 2.013795835 0.051025966 0.383211851 -5.030609615 stable

CASP7 0.143138618 9.108761216 2.013702362 0.051036217 0.383211851 -5.030782789 stable

SART1 -0.089510847 9.449388322 -2.013662573 0.051040581 0.383211851 -5.030856502 stable

FAM156A /// FAM156B -0.150325457 10.20034956 -2.013495827 0.051058874 0.383211851 -5.031165403 stable

TXNDC15 0.134821116 10.299424 2.013484136 0.051060156 0.383211851 -5.03118706 stable

YWHAQ 0.078596543 12.83519595 2.012605409 0.051156652 0.383593014 -5.032814579 stable

CDK13 0.123604385 10.69434482 2.012501169 0.051168109 0.383593014 -5.033007608 stable

KAT6A 0.027220257 3.116738884 2.01249454 0.051168838 0.383593014 -5.033019884 stable

AK022030 /// TROVE2 0.096019074 9.937387278 2.012427775 0.051176178 0.383593014 -5.033143512 stable

FAM181A 0.002930478 1.837852859 2.011996154 0.051223649 0.383766911 -5.033942664 stable

LOC100507217 -0.146307474 13.17530429 -2.01181171 0.051243947 0.383766911 -5.034284123 stable

UBXN7 0.124874843 9.848010897 2.011559606 0.051271701 0.383766911 -5.034750798 stable

CCDC9 -0.091841605 7.441426271 -2.011279348 0.051302571 0.383766911 -5.035269534 stable

WWC3 -0.149208761 9.279488624 -2.011187274 0.051312716 0.383766911 -5.035439942 stable

MRPS28 0.122181965 9.201999733 2.011080617 0.05132447 0.383766911 -5.035637335 stable

ARMC5 -0.105273239 7.813767679 -2.011044218 0.051328482 0.383766911 -5.035704696 stable

NF2 0.047945322 4.038055466 2.010834537 0.051351598 0.383766911 -5.036092724 stable

AKR1C4 0.009803437 2.78616928 2.010810402 0.05135426 0.383766911 -5.036137386 stable

BC043400 /// RP11-875O11.1 0.317515833 3.965119978 2.010735345 0.051362537 0.383766911 -5.036276275 stable

PYGO2 0.122004608 9.027420355 2.010516182 0.051386713 0.383825619 -5.036681798 stable

PTGDS 0.434008081 8.333229814 2.010316949 0.051408699 0.383867939 -5.037050415 stable

CEBPB 0.220190217 14.28237246 2.009909873 0.051453647 0.384035061 -5.037803486 stable

SOX14 0.004912015 2.767700728 2.009818547 0.051463735 0.384035061 -5.037972417 stable

PTPLAD1 0.11877251 7.903667853 2.009429852 0.051506692 0.384233718 -5.038691343 stable

DDX27 /// SS18 -0.082831075 10.04506798 -2.009079671 0.05154542 0.384400705 -5.039338938 stable

CD63 0.139417135 12.19645038 2.008628373 0.051595368 0.384579365 -5.040173392 stable

MB21D1 0.399618575 7.986232621 2.008567771 0.051602078 0.384579365 -5.040285435 stable

NUDT11 0.226624 3.047242005 2.008129961 0.051650579 0.384818897 -5.041094787 stable

CSRNP1 -0.27834235 8.414194956 -2.007862913 0.051680182 0.384825579 -5.041588392 stable

MXRA7 0.515065546 9.275407075 2.007826699 0.051684197 0.384825579 -5.041655325 stable

INPP1 0.219311509 8.878982105 2.006896263 0.051787461 0.385472427 -5.043374679 stable

DYRK1B 0.164132975 5.737271151 2.006066194 0.051879737 0.385718764 -5.04490802 stable

ZSCAN20 0.062773236 2.626992715 2.006053242 0.051881178 0.385718764 -5.044931941 stable

B3GALTL 0.35883841 4.348058197 2.005780913 0.051911483 0.385718764 -5.045434885 stable

SDCCAG3 -0.102697111 8.633369244 -2.005466879 0.051946449 0.385718764 -5.046014782 stable

LOC158402 /// RP11-4O1.2 0.218006368 6.128756155 2.005448649 0.05194848 0.385718764 -5.046048444 stable

LMBR1L -0.082369957 9.028679693 -2.0054191 0.051951771 0.385718764 -5.046103005 stable

C7orf26 -0.066617787 9.792279118 -2.005279303 0.051967345 0.385718764 -5.046361126 stable

SLC13A4 0.004574922 2.595344147 2.00527781 0.051967511 0.385718764 -5.046363882 stable

FHL1 -0.361495055 8.530716012 -2.005161145 0.051980511 0.385718764 -5.046579281 stable

SMARCA4 -0.155431476 6.735872577 -2.005124946 0.051984545 0.385718764 -5.046646114 stable

CLMN -0.259232533 7.992657923 -2.004902577 0.052009333 0.385780988 -5.047056641 stable

FER1L4 0.030431493 2.678058536 2.004660911 0.052036283 0.38585921 -5.04750275 stable

KRT8P12 0.108047052 6.136180748 2.003854295 0.052126324 0.386389041 -5.04899143 stable

PLP2 -0.173277685 12.23959889 -2.003726608 0.05214059 0.386389041 -5.049227043 stable

AC004538.3 /// BC040327 0.00884769 2.566411528 2.003313667 0.052186749 0.386609299 -5.049988934 stable

CA5B 0.244777104 7.052851208 2.003045011 0.052216799 0.386710116 -5.050484546 stable

PBX1 -0.348162153 7.166906194 -2.002761662 0.052248509 0.386746012 -5.051007205 stable

IGLJ3 -0.421326049 9.056014531 -2.002667226 0.052259081 0.386746012 -5.051181387 stable

MUC3A /// MUC3B -0.043922302 2.933529772 -2.002508678 0.052276834 0.386746012 -5.051473804 stable

AHSA1 0.094285675 10.36633254 2.002414196 0.052287416 0.386746012 -5.051648051 stable

RABGGTB -0.107583407 10.41543662 -2.001730481 0.052364049 0.387154566 -5.052908791 stable

ACP1 0.165899582 3.844017373 2.001488961 0.052391143 0.387154566 -5.053354062 stable

ZNF775 0.177878773 6.16879392 2.001481037 0.052392032 0.387154566 -5.053368668 stable

GSE1 0.120369603 10.09293557 2.001310332 0.052411189 0.387170055 -5.053683355 stable

PIH1D3 0.00552452 2.752222363 2.001169049 0.052427049 0.387170055 -5.053943786 stable

PPP1R13L -0.138946185 2.572674632 -2.000989342 0.052447229 0.38719751 -5.054275026 stable

ADM5 -0.064116006 2.727602995 -2.000350143 0.052519059 0.387491034 -5.055453012 stable

GH2 -0.018793569 2.560870437 -2.000342346 0.052519936 0.387491034 -5.055467379 stable

C2orf47 0.174803952 9.045073472 2.00016516 0.052539863 0.387516499 -5.055793861 stable

LOC285902 /// ZNF273 /// ZNF479 /// ZNF733P 0.003421564 2.276000256 1.99979207 0.052581843 0.387567274 -5.056481239 stable

SOGA1 -0.167778214 4.286992193 -1.999734427 0.052588332 0.387567274 -5.056587432 stable

TTC22 0.031147532 2.720753911 1.99966472 0.052596179 0.387567274 -5.056715845 stable

ERI1 0.136753759 8.077533166 1.999314676 0.052635602 0.3877363 -5.057360635 stable

FAM50B 0.317045543 7.103908409 1.99789298 0.052795982 0.388537359 -5.059978498 stable

STAM2 0.157932076 5.978065704 1.997840554 0.052801904 0.388537359 -5.060075005 stable

PNPLA3 -0.028052943 2.857061729 -1.997600849 0.052828989 0.388537359 -5.060516231 stable

USP49 0.018347356 2.748707993 1.997533766 0.052836571 0.388537359 -5.060639703 stable

TTI1 0.089737541 8.507067208 1.997506949 0.052839603 0.388537359 -5.060689061 stable

LINC00623 /// LINC00869 0.155506588 11.84287242 1.99747283 0.05284346 0.388537359 -5.060751858 stable

ORC2 0.092485583 8.810576245 1.997004808 0.05289639 0.388791682 -5.06161318 stable

SCTR -0.012058975 3.110652184 -1.996874745 0.052911108 0.388791682 -5.061852513 stable

RP11-554J4.1 -0.231644245 8.15365237 -1.996600475 0.052942156 0.388898326 -5.062357161 stable

SMARCD2 0.107102363 9.821701344 1.996254699 0.05298132 0.388923509 -5.062993299 stable

KIF3B -0.111324111 8.91543421 -1.996146632 0.052993565 0.388923509 -5.063192096 stable

WFDC8 0.00490889 2.394200212 1.996132303 0.052995189 0.388923509 -5.063218454 stable

LOC102606465 -0.189953638 9.194125437 -1.995745467 0.053039044 0.388993829 -5.063929989 stable

TACC1 0.136973868 7.807175292 1.995737894 0.053039903 0.388993829 -5.063943918 stable

PLA2G16 0.382829103 7.994375909 1.995610209 0.053054386 0.388993829 -5.064178754 stable

LOC101928269 0.003421223 2.305846157 1.994908346 0.053134055 0.389330396 -5.065469385 stable

LINC00424 0.040745069 3.467478099 1.994851749 0.053140484 0.389330396 -5.065573443 stable

UBN2 0.099020566 8.347469 1.994768452 0.053149947 0.389330396 -5.065726587 stable

PSMC2 -0.091353426 9.225336737 -1.994545937 0.053175233 0.389394352 -5.066135662 stable

CATIP-AS1 -0.209964924 3.468544121 -1.994162375 0.053218845 0.389437679 -5.066840721 stable

SPAG8 0.085397166 5.088058749 1.994151008 0.053220138 0.389437679 -5.066861614 stable

ZNHIT2 0.215065214 3.893077757 1.994057096 0.053230821 0.389437679 -5.067034224 stable

NAT8B -0.570747816 6.24254666 -1.993769363 0.053263564 0.389556057 -5.067563037 stable

USP32 /// USP6 -0.135144389 3.761750178 -1.993545245 0.053289079 0.389621521 -5.067974892 stable

DKFZP434F142 0.008951953 2.715563145 1.993331421 0.053313433 0.38967845 -5.068367793 stable

HLA-F 0.080902206 14.09602463 1.993119323 0.053337599 0.389733977 -5.06875749 stable

HM13 0.004311102 2.428474618 1.992814169 0.053372385 0.389867043 -5.069318105 stable

PRIM1 -0.12783978 8.325587573 -1.992203242 0.053442087 0.390254993 -5.070440258 stable

AGPS 0.140032527 9.582135866 1.99188862 0.053478014 0.390343649 -5.071018049 stable

MTO1 0.08134894 10.21267534 1.991723689 0.053496856 0.390343649 -5.071320909 stable

PHF19 0.113544912 8.368536815 1.991656088 0.05350458 0.390343649 -5.071445039 stable

PPIL4 0.220984677 9.330373861 1.991515828 0.05352061 0.390343649 -5.071702573 stable

UNC93A 0.007335008 2.395552497 1.991181763 0.053558806 0.390501141 -5.072315894 stable

AGPAT5 -0.119096882 9.629214007 -1.991006998 0.053578798 0.390525845 -5.072636719 stable

TNIP1 -0.071811089 11.03300497 -1.990583424 0.053627278 0.390672401 -5.073414199 stable

HMGB1P4 /// HMGB1P4 -0.102033893 10.12995142 -1.990541103 0.053632124 0.390672401 -5.073491872 stable

ANKRD53 -0.019471942 3.088693891 -1.99037772 0.053650835 0.390687709 -5.073791726 stable

TRAP1 -0.14398373 8.976037307 -1.99012364 0.053679945 0.390691639 -5.074257991 stable

ZNF624 0.165271219 4.973624347 1.990083061 0.053684596 0.390691639 -5.074332454 stable

TMEM115 0.144428953 8.286281523 1.989744931 0.05372336 0.390852812 -5.074952876 stable

CSRP2 -0.113543336 3.262588232 -1.988511871 0.053864926 0.391761569 -5.077214649 stable

JADE2 0.106649897 7.529014557 1.988100298 0.05391225 0.391769053 -5.077969332 stable

RP11-769O8.3 0.462571902 6.23835604 1.987972812 0.053926916 0.391769053 -5.078203072 stable

PCSK5 0.21636317 8.882001338 1.987906416 0.053934555 0.391769053 -5.078324801 stable

NFYB 0.095996355 9.293539523 1.987895784 0.053935779 0.391769053 -5.078344293 stable

COPS8 0.081450137 10.44944228 1.987778856 0.053949235 0.391769053 -5.078558659 stable

JAZF1 0.164404552 10.75802909 1.986798006 0.054062229 0.392269482 -5.080356454 stable

CRADD 0.234754089 7.053689953 1.986776495 0.05406471 0.392269482 -5.080395874 stable

C2orf81 -0.190324122 5.272200601 -1.986746401 0.05406818 0.392269482 -5.080451021 stable

WASF3 -0.660945529 7.437847563 -1.986482415 0.054098629 0.39229048 -5.080934746 stable

MFSD1 -0.134286346 12.92465561 -1.986203672 0.054130797 0.39229048 -5.081445456 stable

TNFRSF10A -0.170585653 8.122989193 -1.986146733 0.054137369 0.39229048 -5.081549771 stable

HTRA3 -0.073958804 7.016708646 -1.986143112 0.054137787 0.39229048 -5.081556405 stable

CCDC91 -0.399323947 9.260869488 -1.985834362 0.054173441 0.392427936 -5.08212201 stable

C12orf60 0.124324522 3.691132848 1.985121597 0.054255827 0.392903728 -5.083427467 stable

SERHL /// SERHL2 -0.03622201 2.506205189 -1.984153016 0.054367956 0.393222729 -5.085200848 stable

MGC27345 /// RBM28 0.069682877 8.420979991 1.98413144 0.054370456 0.393222729 -5.085240345 stable

KLHL8 0.184074286 5.309615905 1.984058057 0.05437896 0.393222729 -5.085374672 stable

NXN -0.091066668 3.290895472 -1.984035061 0.054381625 0.393222729 -5.085416765 stable

PYCARDOS -0.148660195 5.916256607 -1.984019163 0.054383467 0.393222729 -5.085445866 stable

PCYT2 0.118684186 7.701820041 1.983499866 0.054443682 0.393309842 -5.086396307 stable

TAP1 0.201775609 11.435353 1.983448809 0.054449605 0.393309842 -5.086489744 stable

CHMP4B 0.184016376 9.586672462 1.983441049 0.054450506 0.393309842 -5.086503945 stable

FAM21C /// LOC101930591 -0.089212864 11.64588742 -1.983337224 0.054462553 0.393309842 -5.086693941 stable

LOC100293704 /// MIR8072 -0.040922382 3.903533471 -1.983149688 0.054484319 0.393309842 -5.087037106 stable

RTCA 0.099492211 10.88787991 1.983050408 0.054495845 0.393309842 -5.087218765 stable

RP11-109E10.1 -0.004969747 2.550511036 -1.982857392 0.05451826 0.393350915 -5.087571915 stable

NBN 0.096336114 9.6380791 1.98265218 0.054542099 0.393369338 -5.087947349 stable

MIXL1 -0.040762826 3.110183858 -1.982547514 0.054554261 0.393369338 -5.088138823 stable

FAM122B -0.186747278 10.30984054 -1.982273439 0.054586121 0.393425349 -5.08864017 stable

FMO5 0.0078137 2.790153977 1.982192931 0.054595482 0.393425349 -5.088787427 stable

CTC-471J1.2 0.058433065 3.476641383 1.981642324 0.054659545 0.393766357 -5.089794413 stable

HNRNPC -0.092034506 3.378576491 -1.98086528 0.054750064 0.394297691 -5.091215135 stable

RAD21L1 0.067696972 3.545786716 1.980718305 0.0547672 0.394300371 -5.091483808 stable

LMAN2 0.111691517 10.26806278 1.980401407 0.054804163 0.394364465 -5.092063049 stable

ZNF385D -0.159656021 3.986187791 -1.980254414 0.054821316 0.394364465 -5.092331704 stable

ZNF81 -0.081452903 3.841884494 -1.980210837 0.054826402 0.394364465 -5.092411344 stable

MOCS3 0.391331058 5.764200471 1.979928154 0.054859404 0.394428248 -5.09292794 stable

DAPP1 -0.175275749 10.9121352 -1.979847635 0.054868808 0.394428248 -5.093075077 stable

CTD-2619J13.13 0.035327196 2.944043184 1.979469456 0.054912992 0.394625265 -5.093766074 stable

AK021804 0.006124495 2.617310202 1.979159443 0.054949236 0.394765111 -5.094332439 stable

TMEM245 0.135187959 9.807188372 1.978791145 0.05499232 0.394954003 -5.095005192 stable

LAMP1 0.117754139 10.59099279 1.978606351 0.055013949 0.394988732 -5.095342708 stable

LYST 0.23250807 7.136884382 1.978061448 0.055077768 0.395174404 -5.096337796 stable

A2ML1 0.0038736 2.317678279 1.978051045 0.055078987 0.395174404 -5.096356791 stable

ANP32B -0.079621632 12.53549843 -1.977955265 0.055090212 0.395174404 -5.096531678 stable

AC012065.7 -0.306296389 5.709042223 -1.977565593 0.055135899 0.395381547 -5.097243118 stable

hsa-let-7a-3 /// hsa-let-7b /// hsa-mir-4763 /// RP4-695O20__B.10 0.202175559 2.974442681 1.976703052 0.055237144 0.395767435 -5.098817488 stable

PWWP2B 0.199183826 8.477960084 1.976686891 0.055239042 0.395767435 -5.098846982 stable

ZNF235 -0.057208098 2.79635443 -1.976677127 0.055240189 0.395767435 -5.098864799 stable

CMTM4 0.054807607 2.345773638 1.97626874 0.055288184 0.395976406 -5.099610014 stable

ZDHHC6 -0.05306765 10.5090915 -1.976142504 0.055303027 0.395976406 -5.099840339 stable

OMA1 -0.106265776 9.656213883 -1.975173597 0.055417067 0.396672192 -5.10160778 stable

DIRAS1 -0.151747615 4.003867844 -1.974901734 0.055449101 0.396780746 -5.102103575 stable

TBX6 -0.071660034 2.681796386 -1.974272309 0.05552333 0.397191075 -5.103251239 stable

PLAA 0.22533355 5.150786664 1.97371157 0.055589532 0.397340457 -5.104273413 stable

ATF2 0.026892253 3.380737746 1.973698256 0.055591104 0.397340457 -5.10429768 stable

USP10 0.129862561 9.134657132 1.973666196 0.055594892 0.397340457 -5.104356115 stable

TTC27 -0.114782141 8.487310643 -1.973241664 0.055645062 0.397543554 -5.105129825 stable

LMBRD2 0.110573468 10.06269169 1.973139749 0.055657112 0.397543554 -5.105315545 stable

AKR1C2 /// LOC101930400 0.09745098 2.680752199 1.972725479 0.055706116 0.397772786 -5.106070389 stable

DCUN1D3 0.128035597 6.780999436 1.972414385 0.05574294 0.397798857 -5.106637152 stable

SCAMP1-AS1 -0.144687648 8.760368115 -1.972117965 0.055778047 0.397798857 -5.107177111 stable

USP48 -0.205393088 8.133296567 -1.972063209 0.055784534 0.397798857 -5.107276849 stable

IL1B 0.991678711 8.575658278 1.971858686 0.055808771 0.397798857 -5.107649361 stable

FKBP6 -0.044854701 2.668560895 -1.971688134 0.055828989 0.397798857 -5.107959976 stable

CLEC1B -0.503524131 9.977580864 -1.971665325 0.055831693 0.397798857 -5.108001515 stable

RAD1 0.120301846 7.955245242 1.97155485 0.055844793 0.397798857 -5.108202703 stable

C9orf41 0.167102669 8.814789773 1.971552538 0.055845067 0.397798857 -5.108206913 stable

NRM -0.179178855 9.241133162 -1.971274334 0.055878068 0.397913425 -5.108713513 stable

HN1L -0.127971019 7.564410592 -1.970716877 0.055944246 0.398251305 -5.109728445 stable

GPT2 -0.115445086 3.875016859 -1.970589475 0.055959379 0.398251305 -5.109960367 stable

NKAPL -0.124050578 3.518109321 -1.970284909 0.055995572 0.398365719 -5.110514745 stable

C12orf29 0.140431999 9.107076077 1.97016919 0.056009329 0.398365719 -5.110725363 stable

KIZ -0.101952498 8.502581957 -1.969798461 0.056053421 0.398496559 -5.111400047 stable

C15orf37 -0.192631878 6.979168527 -1.96963096 0.056073353 0.398496559 -5.111704846 stable

DLGAP1-AS1 0.207934873 7.047579868 1.969561982 0.056081563 0.398496559 -5.111830359 stable

ABCD2 0.224174062 5.871353499 1.969444951 0.056095494 0.398496559 -5.1120433 stable

MYL6B -0.251224858 6.041885615 -1.968999961 0.056148492 0.39864765 -5.112852874 stable

CYP1A1 0.00321606 2.260779643 1.968981769 0.05615066 0.39864765 -5.112885968 stable

SETD5 -0.045833716 2.627605996 -1.968826018 0.056169221 0.398659093 -5.11316929 stable

ANKRD37 0.218483201 6.331007917 1.968335991 0.056227652 0.398953422 -5.114060566 stable

BMPR1A 0.181075891 4.05408944 1.966941019 0.056394277 0.39993547 -5.116596788 stable

LINC00692 0.028946872 3.135003325 1.966892616 0.056400066 0.39993547 -5.116684764 stable

AC144652.1 -0.262317281 6.640486263 -1.966050913 0.05650082 0.400480855 -5.118214338 stable

C7orf49 /// LOC653739 0.211805304 4.838345716 1.965834954 0.056526696 0.400480855 -5.118606701 stable

BCL11A -0.289399306 9.881578659 -1.965823598 0.056528057 0.400480855 -5.118627332 stable

SLC6A15 0.010293804 3.006325067 1.964644152 0.056669561 0.401362466 -5.120769563 stable

CD274 0.139487457 4.568046244 1.964139634 0.056730184 0.40167088 -5.121685601 stable

VAV3 0.147908324 8.972969541 1.963656225 0.056788323 0.401777686 -5.12256313 stable

KIFC3 0.063069274 2.826207409 1.963597256 0.056795419 0.401777686 -5.122670164 stable

RGS6 -0.271073981 3.458089997 -1.963588156 0.056796514 0.401777686 -5.12268668 stable

LIG1 -0.172635896 8.206173387 -1.963163793 0.056847601 0.40190427 -5.123456858 stable

BMS1P20 /// LOC101929959 0.175260989 5.280500945 1.96315564 0.056848582 0.40190427 -5.123471652 stable

ATF7 0.256211648 3.923382167 1.962951048 0.056873227 0.401957681 -5.123842916 stable

MTHFD2 0.200816387 10.91102219 1.962758997 0.056896369 0.402000446 -5.124191394 stable

SARNP 0.090450885 9.850768481 1.9625843 0.056917426 0.402028464 -5.124508359 stable

IL26 0.017274302 3.374353584 1.962400561 0.056939582 0.402064214 -5.124841705 stable

PLA2G2A -0.035288456 2.746142257 -1.961969505 0.056991587 0.402251683 -5.12562364 stable

PHACTR2 0.197101768 6.938976986 1.961897037 0.057000334 0.402251683 -5.125755084 stable

VPS37B 0.123278653 10.15790498 1.961570473 0.057039766 0.402409216 -5.12634736 stable

STXBP3 -0.071847996 3.632237685 -1.961113737 0.057094954 0.402565634 -5.127175588 stable

RP11-263K4.3 0.007446373 2.741887753 1.96052431 0.057166245 0.402565634 -5.128244201 stable

SPZ1 0.006371915 2.639285779 1.960370822 0.057184822 0.402565634 -5.128522426 stable

DUS2 -0.156570862 9.12014044 -1.960287012 0.057194968 0.402565634 -5.128674339 stable

MUTYH -0.086513145 8.69689346 -1.960152418 0.057211264 0.402565634 -5.128918294 stable

SUMO3 -0.109012993 10.5867978 -1.960132566 0.057213668 0.402565634 -5.128954274 stable

MFSD8 -0.149614376 9.064769949 -1.960064889 0.057221865 0.402565634 -5.129076934 stable

GAS6-AS1 -0.37435282 5.781573132 -1.960030047 0.057226085 0.402565634 -5.129140081 stable

FRRS1L 0.004356618 2.339543575 1.959626214 0.057275016 0.402565634 -5.129871915 stable

PIK3C3 0.007679027 2.376175745 1.959394657 0.05730309 0.402565634 -5.130291492 stable

RP11-753A21.1 0.009804361 3.263505284 1.959374179 0.057305573 0.402565634 -5.130328594 stable

HTR7 /// HTR7P1 0.007177489 2.524996965 1.959338252 0.05730993 0.402565634 -5.130393689 stable

FARSB -0.075020389 9.416090253 -1.959336595 0.057310131 0.402565634 -5.130396691 stable

CLP1 0.187801474 9.229901855 1.959290398 0.057315734 0.402565634 -5.130480393 stable

RP11-382F24.1 /// RP11-382F24.2 0.007338573 3.063685281 1.959266239 0.057318665 0.402565634 -5.130524165 stable

LDHAL6A 0.004928593 1.976713429 1.959118124 0.057336632 0.40257162 -5.130792511 stable

ZMAT4 0.217273344 2.690087244 1.958837968 0.057370631 0.402690126 -5.131300034 stable

LOC100130458 -0.393866137 4.244103195 -1.958459272 0.057416616 0.402892669 -5.131985977 stable

PCAT6 -0.028920832 2.587466975 -1.957930878 0.057480832 0.403222979 -5.132942889 stable

ITFG2 /// LOC100507424 -0.168025014 7.569992559 -1.957760051 0.057501606 0.403245562 -5.133252208 stable

RAD51D 0.286235753 5.322803516 1.957622489 0.057518339 0.403245562 -5.133501279 stable

AC092192.1 0.047659384 2.34682129 1.957409254 0.057544286 0.403307257 -5.133887335 stable

ADAMTSL5 -0.055998412 2.920970186 -1.95701268 0.057592569 0.403524774 -5.13460523 stable

LOC100287166 /// LOC101930064 -0.131678594 4.57578722 -1.956872585 0.057609634 0.403524774 -5.134858805 stable

EIF2A -0.106491878 11.63426591 -1.95665896 0.057635663 0.403586913 -5.135245447 stable

SRBD1 0.153199657 7.647806638 1.956285917 0.057681142 0.403772617 -5.135920534 stable

KLRAP1 0.249604938 4.286296607 1.956159868 0.057696517 0.403772617 -5.13614862 stable

ORM1 -0.365331537 4.185868797 -1.955179003 0.057816273 0.404490349 -5.137923075 stable

ARHGEF16 -0.033312703 3.015322491 -1.954376978 0.057914354 0.405005877 -5.139373456 stable

TET2 0.31499654 6.050045635 1.954269193 0.057927546 0.405005877 -5.139568336 stable

MAP7D1 0.08968536 10.84098005 1.954154247 0.057941617 0.405005877 -5.139776156 stable

HTR7P1 0.007095967 2.911279575 1.953701837 0.057997029 0.405272761 -5.140594001 stable

LAMTOR3 0.166874891 10.79645307 1.953470646 0.058025363 0.405350331 -5.141011878 stable

MICA /// MICB 0.094754631 8.869244256 1.95332006 0.058043825 0.40535891 -5.141284041 stable

RP11-338N10.1 0.036711747 2.609418347 1.95311346 0.058069162 0.405415485 -5.14165741 stable

CASP16 0.004135275 2.337260639 1.952966636 0.058087175 0.405420901 -5.141922733 stable

PDILT 0.006958245 2.39898435 1.952824762 0.058104584 0.405422109 -5.142179096 stable

FYN 0.116866085 12.93964379 1.952568056 0.058136097 0.405472505 -5.142642918 stable

JAKMIP1 0.360757626 6.545019479 1.95248509 0.058146285 0.405472505 -5.142792813 stable

GGA1 -0.196842448 8.330199317 -1.952179012 0.058183883 0.405614435 -5.143345756 stable

OXR1 0.113907935 9.515936913 1.951929024 0.058214606 0.405708371 -5.143797318 stable

LOC101926913 0.004836635 2.782611165 1.951543398 0.058262027 0.405918586 -5.144493795 stable

PVALB -0.787625555 7.049272376 -1.951378478 0.058282318 0.40593971 -5.144791621 stable

TBC1D22A -0.08595341 7.896638008 -1.950964098 0.058333328 0.406078901 -5.145539853 stable

LOC101928152 -0.45366333 3.838019447 -1.950935647 0.058336831 0.406078901 -5.145591221 stable

CDIP1 0.152874067 7.461684974 1.950757384 0.058358788 0.406111554 -5.14591306 stable

GAFA2 0.00879225 2.944862982 1.9504823 0.058392684 0.40622725 -5.146409655 stable

PTAR1 0.097546149 10.72066448 1.950120673 0.058437271 0.406363645 -5.147062394 stable

POLD2 -0.114351819 8.991220403 -1.949622313 0.058498764 0.406363645 -5.147961774 stable

ACTL7B 0.004049958 2.552477889 1.949536155 0.0585094 0.406363645 -5.148117242 stable

CRH -0.136402449 4.170011928 -1.949495903 0.05851437 0.406363645 -5.148189874 stable

TESK2 -0.111958045 7.947023825 -1.949492198 0.058514828 0.406363645 -5.148196559 stable

LINC00567 0.017941878 3.410590509 1.949385988 0.058527943 0.406363645 -5.148388198 stable

NSUN5 -0.143830362 10.86776604 -1.949343207 0.058533227 0.406363645 -5.148465388 stable

RP11-171N4.1 0.0432598 3.263539044 1.948914003 0.058586258 0.406611796 -5.149239722 stable

RASSF8 0.008002153 2.918449726 1.948689895 0.058613965 0.406654094 -5.149643983 stable

PIGH -0.130485291 8.185851305 -1.948398413 0.058650018 0.406654094 -5.150169722 stable

CAND1.11 0.007760992 2.825438738 1.948358314 0.058654979 0.406654094 -5.150242043 stable

AX748339 -0.115481551 4.935581463 -1.948133771 0.058682768 0.406654094 -5.150646993 stable

TXK 0.300698791 10.77000351 1.948091202 0.058688037 0.406654094 -5.15072376 stable

KIF3A 0.216760371 7.393572061 1.948026181 0.058696086 0.406654094 -5.150841013 stable

RP11-673E11.2 0.005639182 2.840043322 1.947556299 0.058754285 0.406919554 -5.151688258 stable

PNPLA7 -0.136271795 3.623553497 -1.947437533 0.058769003 0.406919554 -5.151902378 stable

ADCY8 0.007928552 2.814098208 1.946840007 0.0588431 0.407312699 -5.152979483 stable

PLGLB2 0.391325979 5.065315549 1.946675804 0.058863476 0.407333868 -5.153275428 stable

UTP15 0.166393928 6.237782062 1.94613014 0.058931232 0.407661918 -5.154258742 stable

ITSN1 -0.032281326 2.610282167 -1.94598141 0.058949712 0.407661918 -5.15452672 stable

CXCL5 -0.088343365 3.715595655 -1.945875471 0.058962878 0.407661918 -5.15471759 stable

NDUFS6 0.10710248 10.29855556 1.945434354 0.059017726 0.407757907 -5.155512253 stable

NBPF10 /// NBPF14 /// NBPF26 -0.098367369 13.22041154 -1.945380937 0.059024371 0.407757907 -5.155608472 stable

IGLL3P -0.283182813 9.950085599 -1.945345587 0.059028769 0.407757907 -5.155672147 stable

ANKRD36BP2 -0.469714455 5.928857656 -1.944587817 0.059123108 0.408246303 -5.157036858 stable

MAP3K19 0.006331105 2.524920847 1.944335614 0.059154536 0.408246303 -5.157490969 stable

LOC101928323 -0.029083165 3.543519447 -1.944161301 0.059176265 0.408246303 -5.157804805 stable

LINC00847 0.256928132 8.326322154 1.944084214 0.059185877 0.408246303 -5.157943585 stable

DCAF11 0.090959185 9.137657739 1.944081186 0.059186255 0.408246303 -5.157949036 stable

IL17A 0.009348546 2.947828763 1.943920591 0.059206283 0.408264728 -5.158238144 stable

MCEE -0.126571749 8.483918923 -1.943305903 0.059282998 0.408611949 -5.159344539 stable

LOC101928927 /// SNHG15 /// SNORA9 -0.126091602 8.882033521 -1.943238771 0.059291381 0.408611949 -5.159465353 stable

PI16 -0.045457777 6.080953145 -1.94287243 0.059337148 0.408685832 -5.160124587 stable

RPA3OS 0.12823848 6.820731097 1.942865716 0.059337987 0.408685832 -5.160136668 stable

MEF2C-AS1 -0.029604415 2.54344341 -1.942735774 0.059354228 0.408685832 -5.160370474 stable

GUSBP1 /// GUSBP4 -0.066979262 2.632144027 -1.94196683 0.059450415 0.40914474 -5.161753782 stable

TMEM17 -0.36029609 3.304987292 -1.94192488 0.059455666 0.40914474 -5.161829235 stable

NFKB2 -0.153678034 9.755504994 -1.941017051 0.059569407 0.40972663 -5.163461785 stable

RSAD1 -0.088315337 9.535648297 -1.940906321 0.059583293 0.40972663 -5.163660869 stable

SNRPB2 -0.262090692 4.072942017 -1.940833049 0.059592483 0.40972663 -5.1637926 stable

MIR7110 /// PDIA5 -0.232059027 8.038470096 -1.940406317 0.059646031 0.409889403 -5.164559717 stable

ZXDC -0.072356823 9.222197722 -1.940366647 0.059651011 0.409889403 -5.164631022 stable

ACSBG1 -0.385874022 4.212813115 -1.940118608 0.059682157 0.409983649 -5.16507684 stable

PIGN 0.137184249 7.454686849 1.939562301 0.059752063 0.410344018 -5.166076557 stable

DQX1 0.014181525 2.839736597 1.939367612 0.059776544 0.41039232 -5.166426369 stable

C1orf198 -0.281969192 8.817190956 -1.939147604 0.059804219 0.410462516 -5.16682164 stable

RABIF 0.157281218 7.218071442 1.938895442 0.059835953 0.410555847 -5.167274633 stable

APAF1 0.182113643 10.42224147 1.938681492 0.05986289 0.410555847 -5.167658944 stable

RFX7 0.116421511 8.616321198 1.938623582 0.059870182 0.410555847 -5.167762959 stable

BTNL3 -0.077622992 5.663237418 -1.938317291 0.059908767 0.410590644 -5.168313064 stable

GSTZ1 -0.231618686 6.996720921 -1.93830616 0.059910169 0.410590644 -5.168333054 stable

HES6 0.145846818 4.142756393 1.937764017 0.059978518 0.410939333 -5.169306574 stable

GZF1 0.153097485 8.146131885 1.937240166 0.060044625 0.41127246 -5.170247033 stable

SEC22C 0.314193558 4.290972694 1.936936137 0.060083021 0.411415642 -5.170792756 stable

RAB4A /// SPHAR -0.171041486 9.502460179 -1.936215084 0.060174166 0.411919837 -5.172086741 stable

CLDN16 0.008628589 2.433879497 1.934442277 0.060398766 0.413235061 -5.175266496 stable

KRT83 -0.065297976 2.886897528 -1.934367218 0.060408291 0.413235061 -5.17540107 stable

ZEB2 0.305213899 9.669586319 1.93428282 0.060419003 0.413235061 -5.175552385 stable

ZNF417 /// ZNF587 0.087893198 3.920917304 1.933051993 0.060575408 0.414066154 -5.177758472 stable

ALAD -0.085871751 9.687880846 -1.933049501 0.060575725 0.414066154 -5.177762938 stable

MIR3656 /// TRAPPC4 0.152433162 10.1613929 1.932349621 0.060664817 0.414174625 -5.179016859 stable

LA16c-395F10.2 -0.017096197 3.099374358 -1.932291931 0.060672166 0.414174625 -5.179120202 stable

PRR24 -0.109026368 8.16537404 -1.932283612 0.060673225 0.414174625 -5.179135102 stable

HDGFRP3 0.128209752 3.109420191 1.932278584 0.060673866 0.414174625 -5.17914411 stable

LEFTY1 -0.290983879 3.547031968 -1.93197323 0.060712777 0.414174625 -5.179691056 stable

ABHD13 -0.085002804 8.511154092 -1.931968883 0.060713331 0.414174625 -5.179698842 stable

DARS2 0.216774813 4.870968802 1.931821896 0.060732069 0.414174625 -5.179962097 stable

TOR1B 0.189449796 9.818646704 1.931818796 0.060732464 0.414174625 -5.179967649 stable

LOC101928161 0.004245588 2.394047249 1.930765098 0.060866937 0.414966566 -5.181854349 stable

TMEM237 0.048976989 3.10658497 1.930623357 0.060885045 0.414966566 -5.182108079 stable

SNX4 0.09589795 9.158287859 1.930494459 0.060901517 0.414966566 -5.182338806 stable

HMG20A 0.10862161 10.4389968 1.929896186 0.06097802 0.415341654 -5.183409543 stable

FER1L5 0.004762501 2.649621117 1.929785766 0.060992149 0.415341654 -5.183607134 stable

CYP4F22 0.299326584 3.280136457 1.929649872 0.061009541 0.415341654 -5.183850296 stable

BBIP1 -0.075820708 10.57670332 -1.929336957 0.061049605 0.415480202 -5.184410157 stable

FAM53B-AS1 0.035278882 2.482975447 1.929215042 0.061065221 0.415480202 -5.184628265 stable

EIF2D -0.11723032 10.14307766 -1.928861918 0.06111047 0.415667834 -5.185259944 stable

PWP1 -0.090543235 10.57015151 -1.928467978 0.061160984 0.415891155 -5.185964524 stable

POU1F1 0.026026502 3.63941718 1.928198642 0.061195541 0.415905655 -5.186446175 stable

ACAD10 -0.111682238 7.313522091 -1.928175731 0.061198481 0.415905655 -5.186487145 stable

PCYOX1 0.213562581 6.895288541 1.927762747 0.061251503 0.416145757 -5.187225568 stable

EPDR1 0.28213348 3.750178458 1.927421811 0.061295305 0.416323093 -5.187835067 stable

POLG 0.085299539 9.762017008 1.926674633 0.061391393 0.416855359 -5.189170509 stable

MEPCE -0.135390773 10.92508548 -1.925676697 0.061519932 0.417607593 -5.190953468 stable

CCDC158 0.003956006 2.53929186 1.925471853 0.061546345 0.417640836 -5.191319357 stable

RAX -0.017765884 2.66249522 -1.925331086 0.061564502 0.417640836 -5.191570775 stable

LRRC2 0.024513287 3.527066992 1.925225317 0.061578147 0.417640836 -5.191759674 stable

NCALD 0.003038296 2.55988971 1.924950084 0.061613668 0.417640836 -5.19225119 stable

LOC100131662 -0.374316677 6.183355999 -1.924909736 0.061618876 0.417640836 -5.19232324 stable

ACTR3BP2 /// ACTR3BP5 -0.036641549 3.184430983 -1.924725287 0.061642692 0.417640836 -5.192652594 stable

CCDC176 -0.290584136 5.492670532 -1.924675507 0.061649121 0.417640836 -5.192741475 stable

IGLC1 -0.326517949 14.15273102 -1.923606011 0.061787383 0.418456962 -5.194650625 stable

RRAGA 0.117142553 10.68164124 1.923202679 0.061839594 0.418610429 -5.195370382 stable

C4orf27 0.029947184 3.439206095 1.923156008 0.061845638 0.418610429 -5.19545366 stable

SNX16 0.248383383 4.896353263 1.922806067 0.061890972 0.418796761 -5.196078026 stable

MAVS -0.077047884 9.476292313 -1.922605522 0.061916965 0.418852149 -5.196435798 stable

LOC100506368 -0.023306 2.474330332 -1.922089564 0.061983882 0.419184269 -5.197356123 stable

SMAP2 -0.168952545 12.23141751 -1.921872025 0.062012114 0.419254654 -5.197744092 stable

RGS12 0.070820722 3.759082251 1.921463961 0.062065103 0.419447372 -5.198471751 stable

ABL1 0.091075382 9.180324653 1.921377888 0.062076285 0.419447372 -5.198625222 stable

SF3B1 -0.114587242 11.54614548 -1.92105945 0.062117669 0.419606459 -5.199192951 stable

FAM120B 0.089263277 8.042899088 1.920760709 0.062156515 0.419651655 -5.199725493 stable

ATF6B -0.151295214 6.001958235 -1.920733583 0.062160043 0.419651655 -5.199773845 stable

LOC728743 /// ZNF775 -0.157703422 8.796725511 -1.920579548 0.062180081 0.419666484 -5.200048402 stable

HDX 0.086424311 3.104461063 1.920279437 0.062219139 0.419777173 -5.200583275 stable

SOX7 0.005793013 2.583559647 1.920179302 0.062232176 0.419777173 -5.200761726 stable

LOC101927592 0.011746828 2.758953341 1.919861269 0.062273596 0.419879355 -5.201328443 stable

TMEM14B -0.097390599 12.34572489 -1.919788885 0.062283027 0.419879355 -5.201457416 stable

RP11-63D14.1 0.005899879 2.879984538 1.918879854 0.062401565 0.420557937 -5.20307678 stable

FOS 0.491329 13.23532054 1.918675598 0.062428226 0.420580898 -5.203440557 stable

SLC9A1 0.017905085 3.796415557 1.918476144 0.062454271 0.420580898 -5.203795753 stable

AC092620.2 -0.312198132 8.024191513 -1.918189772 0.062491681 0.420580898 -5.20430568 stable

NSUN6 -0.254341529 7.955105172 -1.9181378 0.062498472 0.420580898 -5.204398219 stable

ZSCAN9 0.235735257 6.489399861 1.918019587 0.062513922 0.420580898 -5.204608691 stable

EGLN2 /// RAB4B /// RAB4B-EGLN2 0.133839595 7.76165863 1.917838123 0.062537645 0.420580898 -5.204931759 stable

ITGAE -0.101013835 11.06083732 -1.917672027 0.062559365 0.420580898 -5.205227443 stable

GTF2H4 0.137021026 7.139106705 1.917555536 0.062574603 0.420580898 -5.20543481 stable

PLK1 -0.025106074 2.417254601 -1.917481373 0.062584305 0.420580898 -5.205566821 stable

TMEM140 -0.293217392 9.689917398 -1.917444273 0.06258916 0.420580898 -5.20563286 stable

FOXA3 0.002942783 2.222966439 1.917324626 0.062604816 0.420580898 -5.205845822 stable

QRICH2 -0.058104969 3.328441146 -1.917103626 0.062633745 0.420580898 -5.206239156 stable

HES4 0.334261782 4.871989074 1.917075513 0.062637425 0.420580898 -5.206289188 stable

RP11-203B7.1 0.015417463 2.766201348 1.916671949 0.062690284 0.420697738 -5.207007345 stable

LOC100129129 -0.153112875 7.037269204 -1.916669543 0.062690599 0.420697738 -5.207011625 stable

LINC00152 /// LOC101930489 /// MIR4435-1HG -0.132353694 11.16639678 -1.915553241 0.06283701 0.421495363 -5.20899747 stable

LINC01023 -0.072453407 2.934222074 -1.915490123 0.062845297 0.421495363 -5.209109726 stable

HINT3 0.232319056 4.155793056 1.915323824 0.062867136 0.42152164 -5.209405473 stable

PARM1 -0.207872915 2.70544586 -1.914089865 0.063029388 0.422489094 -5.211599296 stable

WNT7A -0.071410172 2.487489803 -1.913749065 0.063074262 0.422669437 -5.212204989 stable

ASAP2 -0.296630925 8.273864535 -1.913232568 0.063142324 0.42272427 -5.213122773 stable

IGKV1-17 /// IGKV1-17 -0.467081093 10.15495348 -1.913157253 0.063152254 0.42272427 -5.213256587 stable

FNDC3B /// LOC101928615 0.216182776 9.747090397 1.912836318 0.063194583 0.42272427 -5.213826748 stable

FAM71A /// FAM71A /// RP11-338C15.5 -0.023787752 2.789017229 -1.912669812 0.063216553 0.42272427 -5.214122525 stable

RP11-1109M24.16 0.007393095 2.860034334 1.912574819 0.063229091 0.42272427 -5.214291259 stable

TBCCD1 0.161639704 7.747500558 1.912496421 0.063239439 0.42272427 -5.21443051 stable

THOC7 -0.083163676 11.02199725 -1.912463634 0.063243768 0.42272427 -5.214488745 stable

POLR3A 0.084518015 3.158040086 1.912373442 0.063255676 0.42272427 -5.214648937 stable

RP11-298H24.1 0.007478636 2.705956286 1.91234303 0.063259691 0.42272427 -5.21470295 stable

LOC100130987 -0.168645657 3.809681938 -1.91214554 0.063285774 0.42272427 -5.215053691 stable

FGFR1OP2 0.094930783 4.120768274 1.912126568 0.06328828 0.42272427 -5.215087383 stable

IGLC1 /// IGLJ3 /// IGLV2-14 /// IGLV@ -0.472804336 10.97606913 -1.911970529 0.063308895 0.42272427 -5.215364482 stable

SIRT1 0.157543003 9.130917366 1.911796844 0.063331849 0.42272427 -5.215672896 stable

5-Mar 0.08405564 9.893954318 1.911748377 0.063338255 0.42272427 -5.215758955 stable

CCDC169 0.00826572 2.557192315 1.911573148 0.063361422 0.42272427 -5.21607008 stable

HEIH -0.120373049 10.31538316 -1.911508279 0.06337 0.42272427 -5.216185253 stable

OBSL1 -0.106988977 2.72732354 -1.91131628 0.063395395 0.422773772 -5.216526116 stable

HOXA10-AS /// MIR196B 0.003774862 2.545262567 1.911074373 0.063427404 0.42286734 -5.216955546 stable

EML6 -0.667049858 5.8149989 -1.910705474 0.063476243 0.422940219 -5.217610322 stable

HPS1 -0.100369571 9.38700042 -1.910585267 0.063492165 0.422940219 -5.217823659 stable

TINF2 0.085207838 10.76031733 1.910517448 0.063501149 0.422940219 -5.217944018 stable

CEP250 0.195674002 7.146062629 1.910448668 0.063510261 0.422940219 -5.218066077 stable

LOC101929450 0.033725512 3.495396136 1.910113302 0.063554709 0.42297838 -5.218661177 stable

RP11-804H8.6 -0.152649027 4.635000769 -1.910091267 0.063557631 0.42297838 -5.218700274 stable

C12orf5 0.193436656 8.96084895 1.909998425 0.063569941 0.42297838 -5.218865004 stable

PSMA5 0.118608865 10.54998986 1.909438543 0.06364422 0.423269444 -5.219858261 stable

CCL11 0.004794118 2.626362713 1.90939744 0.063649676 0.423269444 -5.219931171 stable

GGT7 -0.122602673 7.752889579 -1.909056282 0.063694977 0.423450975 -5.220536272 stable

LRP8 0.157884419 6.495364766 1.908885826 0.063717621 0.423481824 -5.220838572 stable

MTERF2 0.247813399 7.100241223 1.90841151 0.063780669 0.423757378 -5.221679642 stable

PLEKHA1 0.179485856 9.318204837 1.908180981 0.063811331 0.423757378 -5.222088359 stable

MIER1 0.174717695 8.078013987 1.90816746 0.06381313 0.423757378 -5.222112331 stable

ZNF112 0.078613319 3.360368983 1.907259947 0.063933962 0.424439943 -5.223720905 stable

CCNB1IP1 -0.101162709 9.667505333 -1.906923624 0.063978792 0.424525295 -5.224316879 stable

DERA -0.081921503 10.29313369 -1.906862882 0.063986891 0.424525295 -5.224424508 stable

ACTG1P4 /// AMY2B /// RNPC3 -0.189120597 10.33304781 -1.906717234 0.064006316 0.424525295 -5.224682566 stable

FBXO11 -0.167725385 9.705283144 -1.906495805 0.064035857 0.424525295 -5.225074863 stable

LOC100996696 /// PABPC4 -0.061597603 11.71436166 -1.90644385 0.06404279 0.424525295 -5.225166904 stable

TSG101 0.124349263 10.33127259 1.906351525 0.064055111 0.424525295 -5.225330457 stable

ATP13A1 -0.063056096 9.781962822 -1.906129135 0.0640848 0.424602416 -5.225724394 stable

SNAPC3 0.095980906 7.776212843 1.905552541 0.064161828 0.424993063 -5.226745582 stable

FSHB -0.043857345 2.683229593 -1.904400907 0.064315916 0.425810487 -5.228784442 stable

DUSP22 /// LOC100653247 -0.131558468 11.19285475 -1.904359645 0.064321443 0.425810487 -5.228857475 stable

DEFB107A /// DEFB107B 0.010601472 2.765909106 1.904221155 0.064339995 0.425813459 -5.229102584 stable

RP11-559M23.1 -0.13615494 5.850367183 -1.903817065 0.064394155 0.425899693 -5.229817692 stable

PRRC2B 0.121645562 10.08814486 1.903645912 0.064417106 0.425899693 -5.230120538 stable

BCL7B -0.074282816 9.769415557 -1.903610033 0.064421918 0.425899693 -5.23018402 stable

HRASLS -0.602226704 6.844092965 -1.90358367 0.064425454 0.425899693 -5.230230666 stable

GPR45 -0.012146122 3.331467676 -1.903230495 0.064472841 0.425934243 -5.230855503 stable

GLI3 0.007347237 2.769485827 1.903145958 0.064484189 0.425934243 -5.231005052 stable

MRPL37 -0.094327148 9.726923332 -1.903094405 0.064491109 0.425934243 -5.231096248 stable

TGS1 0.117990098 8.01865906 1.903004982 0.064503115 0.425934243 -5.23125443 stable

RASA2 0.172979259 9.307789795 1.902744229 0.064538135 0.42597771 -5.231715648 stable

ZNF641 0.215473296 7.173195578 1.902568189 0.064561787 0.42597771 -5.232026997 stable

TRMT10C 0.184876919 9.07679025 1.9025515 0.064564029 0.42597771 -5.232056511 stable

GEMIN6 0.254551535 7.848857991 1.902183043 0.064613559 0.426176343 -5.232708091 stable

LOC101928728 -0.102049607 2.494853806 -1.902058012 0.064630374 0.426176343 -5.232929173 stable

FRAT1 0.240680222 9.601457736 1.901581065 0.064694549 0.426479959 -5.233772405 stable

THAP9 0.040619359 3.592446766 1.901223227 0.064742734 0.42667802 -5.23440494 stable

TTLL4 -0.098106788 4.979764057 -1.901054232 0.064765501 0.426708501 -5.234703631 stable

SLC8A3 -0.368927403 4.263907285 -1.900292379 0.064868222 0.4272656 -5.2360499 stable

ISG20 -0.14630892 11.5533357 -1.899940214 0.064915752 0.427420422 -5.236672058 stable

FAM229A -0.178656265 4.319972503 -1.89983453 0.064930022 0.427420422 -5.236858749 stable

IGLC1 /// IGLV3-25 /// IGLV3-25 -0.411701177 9.489164639 -1.899714412 0.064946243 0.427420422 -5.237070925 stable

ZNF154 -0.345946046 5.982828806 -1.899298638 0.065002419 0.427602639 -5.237805266 stable

LINC01187 0.015764451 2.822003906 1.899205959 0.065014947 0.427602639 -5.237968936 stable

PLEKHM3 0.2761553 6.310350036 1.899010849 0.065041327 0.427602639 -5.238313481 stable

KAT7 0.094869691 9.449345864 1.898786549 0.065071666 0.427602639 -5.238709534 stable

CNN3 -0.148396233 2.947203053 -1.898779044 0.065072681 0.427602639 -5.238722786 stable

PRSS54 0.003381099 2.440706461 1.89870271 0.065083009 0.427602639 -5.238857562 stable

OSBP2 0.035317376 3.305866392 1.898529994 0.065106382 0.42763675 -5.239162493 stable

NGDN -0.06350966 10.33295823 -1.898277224 0.065140601 0.427645565 -5.23960872 stable

FKBP1B /// MFSD2B -0.186039537 5.840407818 -1.898151972 0.065157563 0.427645565 -5.239829816 stable

LINC01278 0.189290932 8.040792376 1.897835414 0.065200449 0.427645565 -5.240388553 stable

RBP2 -0.037731843 3.27473071 -1.897818562 0.065202733 0.427645565 -5.240418295 stable

LOC100131860 0.011969102 2.599566416 1.897758751 0.065210839 0.427645565 -5.240523854 stable

NOX3 0.00520078 2.368708902 1.897714678 0.065216812 0.427645565 -5.240601635 stable

LINC01365 0.006211267 2.719697084 1.897157426 0.065292382 0.42802177 -5.241584965 stable

CHCHD7 -0.099495797 9.744467703 -1.896526851 0.065377985 0.428463524 -5.242697392 stable

EMD -0.108857227 8.165206086 -1.895765263 0.065481502 0.428915042 -5.244040538 stable

ANO6 -0.17711096 11.23601752 -1.895751666 0.065483352 0.428915042 -5.244064514 stable

CRTC3 -0.134269566 11.590951 -1.895413663 0.06552934 0.429083302 -5.244660473 stable

MAU2 -0.08899487 9.340680428 -1.895071503 0.065575922 0.429083302 -5.245263672 stable

TMC4 0.089041567 3.464896408 1.89467742 0.065629608 0.429083302 -5.245958296 stable

RABGEF1 -0.083423887 5.681755099 -1.894612365 0.065638474 0.429083302 -5.246072951 stable

MFSD10 0.07999486 9.204292174 1.894217095 0.065692366 0.429083302 -5.246769526 stable

SST 0.006718675 2.609362284 1.894105891 0.065707535 0.429083302 -5.246965478 stable

IGHG1 /// IGHM /// LOC102725426 /// MIR8071-1 /// MIR8071-2 -0.577084098 9.388617271 -1.894091756 0.065709463 0.429083302 -5.246990385 stable

MVD 0.157664673 5.126467973 1.894083268 0.065710621 0.429083302 -5.24700534 stable

C12orf65 0.206068158 6.618550138 1.894065489 0.065713047 0.429083302 -5.247036667 stable

ZBP1 0.198302773 6.603622015 1.893828569 0.065745376 0.429083302 -5.247454101 stable

TUSC2 0.123269626 9.004124961 1.89354311 0.065784346 0.429083302 -5.247956998 stable

MNX1-AS1 0.003506897 2.371767004 1.893503045 0.065789817 0.429083302 -5.248027575 stable

PFKFB3 0.190690771 9.426474562 1.89340139 0.065803701 0.429083302 -5.248206645 stable

HDGF -0.066908516 11.51367132 -1.893348907 0.06581087 0.429083302 -5.248299092 stable

ZFP2 0.009271707 3.139042244 1.893334471 0.065812842 0.429083302 -5.24832452 stable

CHTF18 -0.111969987 6.421361696 -1.893326324 0.065813955 0.429083302 -5.248338872 stable

M6PR /// PHC1 0.085711707 11.46983064 1.893288193 0.065819164 0.429083302 -5.248406036 stable

RAP2B 0.067361372 11.84941768 1.892940291 0.065866707 0.429161847 -5.249018788 stable

RPL22P22 /// RPL22P22 -0.060564146 8.736453184 -1.892754202 0.06589215 0.429161847 -5.249346503 stable

FHL2 -0.538096107 6.424071512 -1.892599836 0.065913262 0.429161847 -5.249618332 stable

IGLJ3 /// IGLV3-19 /// IGLV3-19 -0.425305189 8.774306902 -1.892470875 0.065930903 0.429161847 -5.24984541 stable

REEP4 0.216174835 8.738373907 1.892327288 0.06595055 0.429161847 -5.250098227 stable

SNX18 0.113767652 2.379672223 1.892275489 0.065957639 0.429161847 -5.250189426 stable

SEC62 0.130125555 7.863661994 1.892266033 0.065958934 0.429161847 -5.250206075 stable

RCC2 0.071556405 11.44094112 1.891894876 0.066009748 0.429373697 -5.250859488 stable

NAPB 0.156549515 5.495305765 1.891538108 0.066058624 0.429572824 -5.251487469 stable

RSPO2 0.006164775 2.744378517 1.891325561 0.066087758 0.42964349 -5.251861548 stable

DOCK2 0.086926696 2.647539415 1.890925757 0.066142587 0.429881126 -5.2525651 stable

KIAA1715 0.108497399 7.801742898 1.890663574 0.066178564 0.429996137 -5.253026406 stable

IGSF11 0.005474221 2.288662159 1.890407863 0.06621367 0.430105421 -5.253476276 stable

ELL 0.17408038 6.52814953 1.88991548 0.066281311 0.430277758 -5.254342375 stable

GBP3 0.304193539 10.49002102 1.889817524 0.066294775 0.430277758 -5.254514656 stable

PRKCI 0.126313274 4.644972556 1.889815301 0.066295081 0.430277758 -5.254518566 stable

TMEM52B 0.035048187 3.287477006 1.889501655 0.066338207 0.430438887 -5.255070147 stable

NHP2 -0.101576689 11.49795058 -1.889157983 0.066385489 0.430626885 -5.255674445 stable

PET112 -0.132025062 6.759375938 -1.888886492 0.066422861 0.430667631 -5.256151758 stable

KRT18 -0.223489892 5.533394529 -1.888846335 0.06642839 0.430667631 -5.256222353 stable

IGLL5 -0.414431883 10.31099279 -1.888650505 0.06645536 0.430688288 -5.256566604 stable

ATL1 -0.288480794 5.969327856 -1.888215892 0.066515249 0.430688288 -5.257330508 stable

GOLPH3L 0.110711096 9.453956629 1.888085952 0.066533163 0.430688288 -5.25755887 stable

NIPAL4 -0.149258093 2.924469466 -1.888075483 0.066534607 0.430688288 -5.257577267 stable

FAM212B-AS1 -0.353812462 7.049458218 -1.888027276 0.066541254 0.430688288 -5.257661986 stable

WNT6 0.004867729 2.31061658 1.888014079 0.066543074 0.430688288 -5.257685178 stable

EFHC1 -0.199546719 6.856495228 -1.887893151 0.066559752 0.430688288 -5.257897684 stable

ZSCAN16 0.127819525 7.159856357 1.887750835 0.066579384 0.430696835 -5.258147759 stable

RNF139-AS1 -0.275571319 2.522906323 -1.887537514 0.06660882 0.430768784 -5.258522575 stable

OSBPL2 0.064469273 8.721638082 1.887017856 0.066680575 0.431114296 -5.259435494 stable

ANAPC15 -0.152369272 9.218297991 -1.886487289 0.066753905 0.431469797 -5.260367364 stable

SYCE2 -0.007299522 2.432910452 -1.886209792 0.066792285 0.431599267 -5.260854664 stable

ADAMTSL4 0.087927829 4.492516802 1.885953688 0.066827723 0.431709659 -5.261304342 stable

TMCC3 0.35227111 7.782399952 1.885796465 0.066849486 0.431731677 -5.261580376 stable

MSANTD3-TMEFF1 /// TMEFF1 -0.189840133 3.885975118 -1.885149585 0.066939094 0.432052385 -5.262715894 stable

MGAT4B -0.179405106 10.13958936 -1.885111719 0.066944343 0.432052385 -5.262782354 stable

IRF8 -0.196393315 11.81877602 -1.884910904 0.066972183 0.432052385 -5.263134788 stable

GPR171 0.256741485 10.55248584 1.884907752 0.06697262 0.432052385 -5.263140319 stable

ZZZ3 0.077612076 9.273915014 1.88469192 0.067002553 0.43212697 -5.263519074 stable

HMGCS1 0.235950239 7.106741399 1.884137056 0.067079559 0.432505019 -5.264492614 stable

CCDC14 0.003972652 2.400397544 1.883933198 0.06710787 0.432568982 -5.264850235 stable

RP4-781L3.1 0.076533739 9.618669289 1.883522646 0.067164917 0.432742581 -5.265570354 stable

ITPR3 -0.083402164 4.487955854 -1.883474583 0.067171598 0.432742581 -5.26565465 stable

PIH1D1 -0.056384309 10.24463463 -1.882708888 0.067278113 0.433194261 -5.266997321 stable

FGF6 -0.139716284 6.110107449 -1.88270579 0.067278544 0.433194261 -5.267002754 stable

GOLT1B -0.068472979 9.578463515 -1.882292557 0.067336089 0.433446124 -5.267727183 stable

LOC101927204 0.161890301 8.485641965 1.882136545 0.067357825 0.433467414 -5.268000649 stable

PPIF -0.151609071 10.1323517 -1.881973136 0.067380598 0.433495363 -5.268287062 stable

C19orf33 -0.517178485 8.908289596 -1.88163301 0.06742802 0.433681835 -5.268883147 stable

MTMR12 -0.113041855 7.514826031 -1.881364991 0.067465409 0.433781682 -5.269352797 stable

INO80D -0.054055823 3.124492432 -1.881160002 0.067494017 0.433781682 -5.269711961 stable

C7orf73 -0.177224762 8.303678 -1.88100158 0.067516134 0.433781682 -5.269989514 stable

DHODH -0.064651704 3.333509942 -1.880993128 0.067517314 0.433781682 -5.270004321 stable

ANKRD13A -0.067640067 12.28785083 -1.880773484 0.067547988 0.433860246 -5.270389099 stable

LOC101927603 0.107310718 3.463577076 1.880485249 0.067588259 0.433966119 -5.27089398 stable

MKNK1 0.152458185 6.597297737 1.880391426 0.067601372 0.433966119 -5.27105831 stable

NAV2-AS5 0.004234321 2.268585791 1.880142942 0.067636111 0.434070658 -5.271493491 stable

CTD-2251F13.1 0.008140731 2.793086233 1.879537944 0.067720756 0.434373334 -5.272552855 stable

ASXL2 0.110360961 9.008490949 1.879388394 0.067741694 0.434373334 -5.272814676 stable

ITGB3BP -0.294293172 9.797826475 -1.879291264 0.067755295 0.434373334 -5.272984715 stable

LOC101928054 0.155163926 9.375232752 1.87927807 0.067757143 0.434373334 -5.273007811 stable

EGFEM1P 0.011441719 2.806209861 1.878624214 0.067848768 0.434667744 -5.274152275 stable

LOC100507459 0.013456319 2.850573003 1.878592796 0.067853174 0.434667744 -5.27420726 stable

IL34 -0.082934759 6.149728933 -1.878408092 0.067879077 0.434667744 -5.274530488 stable

RP3-384D21.2 -0.056938337 2.756311194 -1.878397993 0.067880493 0.434667744 -5.274548161 stable

DAG1 0.094833639 9.360184662 1.878264952 0.067899157 0.434667744 -5.274780963 stable

LCP2 0.122406426 12.28752152 1.87815954 0.067913948 0.434667744 -5.274965408 stable

RAB13 -0.247325498 8.193121992 -1.877374328 0.06802421 0.43516904 -5.276339069 stable

TNPO2 0.071376429 4.321539083 1.877338289 0.068029274 0.43516904 -5.276402104 stable

SH3BP5-AS1 -0.236881094 7.486615009 -1.877094091 0.068063599 0.435188446 -5.276829204 stable

RHOBTB1 -0.395023542 8.225641367 -1.876993185 0.068077787 0.435188446 -5.277005673 stable

FAM27B /// FAM27C -0.01488804 2.520113264 -1.87690832 0.068089721 0.435188446 -5.277154083 stable

MIR636 /// SRSF2 -0.094434044 11.22234563 -1.876712361 0.068117285 0.435188446 -5.277496752 stable

TSC22D2 0.089907325 10.24359972 1.876314736 0.068173244 0.435188446 -5.278191976 stable

BRWD1-IT2 -0.10457102 3.113958666 -1.876258132 0.068181214 0.435188446 -5.278290936 stable

LOC100129884 -0.093503956 2.95159135 -1.876215083 0.068187275 0.435188446 -5.278366195 stable

TMED9 0.096678564 11.5740914 1.875982461 0.068220037 0.435188446 -5.278772845 stable

LPAR5 0.277695032 9.051839934 1.875923293 0.068228372 0.435188446 -5.278876273 stable

ANKRD30BP2 0.008490374 2.939645546 1.87588504 0.068233762 0.435188446 -5.278943137 stable

PGM1 0.152106426 10.26046912 1.875838036 0.068240384 0.435188446 -5.279025297 stable

DGKZ 0.110876191 11.96620311 1.875736242 0.068254729 0.435188446 -5.279203221 stable

AZIN1 0.077753178 11.24907663 1.875522947 0.068284793 0.435188446 -5.279576009 stable

THEM4 -0.294190137 9.488143122 -1.875476529 0.068291338 0.435188446 -5.279657132 stable

ZNF691 0.140305452 2.623300584 1.875260838 0.068321754 0.435264351 -5.280034064 stable

GPN3 0.201052751 8.884141226 1.875037802 0.068353219 0.435346888 -5.280423793 stable

MED12L 0.003970533 2.278477796 1.874395 0.06844397 0.435806884 -5.2815468 stable

DHFR -0.151461201 8.307086476 -1.874059196 0.068491421 0.43599099 -5.282133339 stable

BOD1L1 0.066005922 10.52975789 1.873694139 0.068543036 0.436201503 -5.282770874 stable

PIGW -0.12451637 6.432280104 -1.873426209 0.06858094 0.436206971 -5.283238722 stable

USH1C -0.124587382 2.858609985 -1.873425884 0.068580986 0.436206971 -5.28323929 stable

SLCO2B1 0.005863688 2.1416955 1.873221945 0.068609849 0.436272578 -5.28359536 stable

MAPK8IP3 -0.185832805 7.865323219 -1.873052023 0.068633906 0.436307596 -5.283892014 stable

LPCAT3 /// U47924.30 -0.193108464 6.11913284 -1.872871982 0.068659403 0.436351749 -5.284206309 stable

FBXO33 -0.155420729 9.929565955 -1.872373171 0.068730087 0.436569072 -5.285076948 stable

PPP1R17 0.491452366 3.951085043 1.8723687 0.06873072 0.436569072 -5.285084751 stable

AP001347.6 /// BC024173 /// BC048201 0.008391339 2.745338602 1.872185297 0.068756725 0.436616341 -5.285404818 stable

RP11-486G15.2 -0.208784924 3.661017276 -1.871474221 0.068857628 0.437118244 -5.286645511 stable

ZNF516 0.19517483 8.510971815 1.871366452 0.068872931 0.437118244 -5.286833514 stable

LOC646626 -0.075963828 2.279262744 -1.871142845 0.068904693 0.43713621 -5.287223566 stable

CHST8 -0.204506981 3.883637636 -1.87102296 0.068921728 0.43713621 -5.287432673 stable

TRUB2 -0.074608915 7.750436621 -1.870846349 0.068946828 0.43713621 -5.287740703 stable

PPP1R3E -0.242263388 6.801994847 -1.870816232 0.06895111 0.43713621 -5.287793229 stable

MRPL40 -0.114372111 9.641859116 -1.870692599 0.068968687 0.43713621 -5.288008841 stable

KLHL31 0.004147682 2.301305271 1.870380878 0.069013021 0.437299372 -5.288552423 stable

NPTX2 0.005786834 2.541526541 1.870088817 0.069054581 0.437444871 -5.289061652 stable

GNAS-AS1 -0.007327312 2.368730545 -1.869906489 0.069080537 0.437463986 -5.289379521 stable

MTL5 0.015863652 2.192307849 1.86973401 0.069105099 0.437463986 -5.289680196 stable

CACUL1 0.140117398 8.961149463 1.869537062 0.069133155 0.437463986 -5.290023498 stable

IGHA1 /// IGHD /// IGHG1 /// IGHG3 /// IGHM /// IGHV4-31 -0.400082033 9.426482517 -1.86952555 0.069134795 0.437463986 -5.290043562 stable

ALKBH4 -0.111493185 7.098860876 -1.869358342 0.069158622 0.437463986 -5.290335 stable

COPB1 0.070986753 12.36438582 1.86928418 0.069169192 0.437463986 -5.290464254 stable

RGL2 -0.069277373 9.540010419 -1.868879945 0.069226831 0.437710833 -5.291168705 stable

LOC101930566 /// LOC102800447 0.015413545 3.247910683 1.868310099 0.069308155 0.438049078 -5.292161547 stable

PANK2 0.105615596 10.66057126 1.86824413 0.069317574 0.438049078 -5.292276469 stable

COL15A1 0.025333049 2.547123032 1.867749141 0.069388289 0.438340749 -5.293138659 stable

CCL26 0.005841395 2.448567288 1.867354628 0.069444694 0.438340749 -5.293825699 stable

NDC1 0.121049887 8.298010217 1.867235162 0.069461782 0.438340749 -5.294033725 stable

DNASE2 -0.12918462 8.476745887 -1.867212569 0.069465014 0.438340749 -5.294073064 stable

FCHO2 0.083430295 3.603436977 1.867202116 0.06946651 0.438340749 -5.294091264 stable

TARP 0.374801553 6.204287009 1.867138958 0.069475546 0.438340749 -5.294201235 stable

BCL3 /// MIR8085 0.244854878 6.42200998 1.86694932 0.069502683 0.438386383 -5.294531413 stable

C8G 0.043254617 2.620936027 1.86670681 0.0695374 0.438386383 -5.294953606 stable

SLC6A10P /// SLC6A8 -0.033816179 2.919464676 -1.866697778 0.069538693 0.438386383 -5.294969328 stable

ZNF543 0.028013724 3.061222416 1.866406644 0.06958039 0.438433498 -5.295476109 stable

COA6 0.154683092 9.146296457 1.866385311 0.069583447 0.438433498 -5.295513241 stable

MICU1 -0.147203322 9.668485288 -1.866044873 0.069632235 0.438623404 -5.296105758 stable

TIMM44 -0.102849439 7.132029633 -1.865425129 0.069721125 0.439065748 -5.29718416 stable

LOC101927534 -0.032656741 2.733089687 -1.865169572 0.069757807 0.439130255 -5.297628763 stable

MNAT1 0.133554046 6.844128868 1.864959902 0.069787916 0.439130255 -5.297993495 stable

MYO18A /// TIAF1 -0.116891462 9.93590706 -1.864891537 0.069797735 0.439130255 -5.298112412 stable

PCGF1 0.165902244 8.346080558 1.86477355 0.069814685 0.439130255 -5.298317637 stable

HLA-DMB -0.178633035 12.38532631 -1.864703733 0.069824716 0.439130255 -5.298439069 stable

NFE2L1 0.141333224 9.931483991 1.864357547 0.069874475 0.439303208 -5.299041137 stable

CRIPT 0.268751641 7.427935891 1.86425256 0.069889571 0.439303208 -5.299223705 stable

PDHB 0.093997829 10.09973579 1.863622939 0.069980163 0.439755121 -5.300318415 stable

TECR -0.395742091 6.264882078 -1.862925482 0.070080633 0.440268845 -5.301530711 stable

NDUFA6 -0.160918296 9.606949453 -1.861947098 0.070221777 0.44103776 -5.303230664 stable

LOC101929315 0.005590051 2.533442642 1.861285539 0.070317353 0.441441529 -5.304379711 stable

AURKAPS1 /// RAB3GAP2 0.084330378 9.741832946 1.861242318 0.070323601 0.441441529 -5.304454768 stable

APP -0.188522065 9.844931868 -1.860661111 0.070407665 0.441721459 -5.305463951 stable

PPP2CB 0.032747679 4.282439374 1.860561232 0.070422121 0.441721459 -5.305637351 stable

UBIAD1 -0.105856554 8.354056751 -1.860157204 0.070480619 0.441721459 -5.306338701 stable

SLC14A1 0.25944861 5.482365596 1.860144572 0.070482449 0.441721459 -5.306360627 stable

ANO9 -0.254243585 8.951594532 -1.860115539 0.070486654 0.441721459 -5.306411021 stable

NCL 0.061001575 13.76231373 1.860110603 0.070487369 0.441721459 -5.306419588 stable

DEK 0.067506477 12.98425582 1.860025802 0.070499654 0.441721459 -5.306566775 stable

RCAN3 -0.233758793 8.752429873 -1.859612949 0.070559487 0.441978612 -5.30728327 stable

SBF1 0.156574385 6.920728259 1.85908305 0.070636346 0.442342252 -5.308202703 stable

NOL8 -0.063410225 10.29084921 -1.858390912 0.070736845 0.442743065 -5.309403309 stable

XCL1 0.058779387 2.874920997 1.85835989 0.070741352 0.442743065 -5.309457113 stable

ZBTB20-AS1 0.064576419 2.937566877 1.85825344 0.070756821 0.442743065 -5.309641729 stable

NDUFA2 0.046760294 2.551705352 1.858052028 0.070786096 0.442808447 -5.309991014 stable

GFM2 0.063891511 9.152590213 1.85768493 0.07083948 0.442915163 -5.310627545 stable

PI4K2B /// SEPSECS-AS1 0.130577934 8.691458092 1.857675742 0.070840816 0.442915163 -5.310643476 stable

TMBIM4 -0.143369975 5.767512181 -1.857451067 0.070873506 0.443001792 -5.311032998 stable

TNF -0.464231598 5.635481516 -1.857126352 0.070920775 0.443179476 -5.311595894 stable

RP11-108K3.2 -0.00303448 2.182572099 -1.856846215 0.070961576 0.44331666 -5.312081448 stable

RP11-5C23.1 0.097382553 8.113944133 1.856355601 0.07103308 0.443645533 -5.312931666 stable

FLJ37453 -0.126500152 8.717399159 -1.856054249 0.071077031 0.443802189 -5.313453808 stable

PTPRH -0.041915498 2.886990258 -1.855671918 0.071132825 0.444017232 -5.314116155 stable

WNT2B 0.008323892 2.998458081 1.855559581 0.071149226 0.444017232 -5.314310745 stable

MCM3 0.101056946 10.06285381 1.855188379 0.071203443 0.444154229 -5.314953672 stable

GDPD5 -0.186648146 7.825028134 -1.855150721 0.071208945 0.444154229 -5.315018891 stable

TCF4 -0.31342549 9.915045535 -1.854792196 0.071261347 0.444363241 -5.315639748 stable

RPAIN -0.184610997 7.740019244 -1.85463591 0.0712842 0.444387933 -5.315910356 stable

RAB5C 0.170240769 10.57739963 1.854233404 0.071343086 0.444637181 -5.316607208 stable

EHD4 -0.192176981 5.029415757 -1.85403264 0.071372472 0.444702496 -5.316954739 stable

DNAJC5 0.074536728 9.741367077 1.853888784 0.071393536 0.44471593 -5.317203742 stable

TUBB2B 0.009258673 3.516280238 1.853608985 0.071434519 0.444853405 -5.317688003 stable

ANXA2R 0.178406187 10.01394334 1.853026197 0.071519945 0.445267505 -5.318696464 stable

PCDHA2 /// PCDHA3 0.006554852 3.003490725 1.852739253 0.071562038 0.445387137 -5.319192898 stable

GDAP2 -0.241109548 8.748049501 -1.852611593 0.071580772 0.445387137 -5.319413738 stable

PARVB -0.339679794 9.391629766 -1.852486403 0.071599148 0.445387137 -5.319630294 stable

LINC00630 0.008452853 2.864091017 1.852372527 0.071615866 0.445387137 -5.319827267 stable

LOC101060363 /// PPIA 0.042816761 14.82105959 1.852250124 0.07163384 0.445387137 -5.320038978 stable

PCDHGA1 /// PCDHGA10 /// PCDHGA11 /// PCDHGA12 /// PCDHGA2 /// PCDHGA3 /// PCDHGA4 /// PCDHGA5 /// PCDHGA6 /// PCDHGA7 /// PCDHGA8 /// PCDHGA9 /// PCDHGB1 /// PCDHGB2 /// PCDHGB3 /// PCDHGB4 /// PCDHGB5 /// PCDHGB6 /// PCDHGB7 /// PCDHGC3 /// PCDHGC4 /// PCDHGC5 0.146400458 2.428555682 1.851665155 0.07171979 0.445734376 -5.321050594 stable

IL22RA2 0.021979516 3.254299907 1.851612106 0.071727589 0.445734376 -5.32114232 stable

PSMA3 0.079064907 11.6781422 1.851334541 0.071768406 0.445870228 -5.321622223 stable

LOC101929084 -0.015462378 2.847670006 -1.851044944 0.071811014 0.446017128 -5.322122863 stable

GATC 0.141663687 9.151805305 1.850728537 0.071857591 0.446097625 -5.322669776 stable

POLM -0.145333614 8.792897555 -1.850699231 0.071861907 0.446097625 -5.322720428 stable

SEL1L2 0.003710935 2.597936463 1.850175662 0.07193904 0.446458614 -5.32362524 stable

CCRL2 0.491948196 4.534320684 1.849975709 0.071968516 0.446523728 -5.323970735 stable

TGIF1 -0.43861652 6.446289032 -1.84965608 0.072015655 0.446698369 -5.32452295 stable

RP11-182J23.1 0.007659342 2.94286409 1.849023789 0.072108983 0.447159346 -5.325615111 stable

FAHD2CP -0.145694592 4.049465444 -1.848819663 0.072139135 0.447184277 -5.325967631 stable

SHOC2 -0.049768434 11.95558143 -1.848739168 0.072151028 0.447184277 -5.326106635 stable

C21orf49 -0.178548252 3.764650079 -1.848533029 0.072181492 0.447224232 -5.326462588 stable

PRPS2 -0.092589715 9.082728048 -1.848438254 0.072195502 0.447224232 -5.32662623 stable

C15orf52 -0.190804447 6.056942792 -1.848202302 0.072230392 0.447286061 -5.327033603 stable

PRKAR1B -0.16505426 7.66415245 -1.848113567 0.072243516 0.447286061 -5.327186794 stable

CRHR1 0.003212716 2.206632625 1.847767732 0.072294687 0.44748509 -5.327783779 stable

LOC100130987 /// RAD9A 0.153601429 7.268533983 1.84728132 0.072366711 0.44762161 -5.328623271 stable

AJ011981 /// DPY19L1 0.127376761 3.007294171 1.847091654 0.072394812 0.44762161 -5.328950563 stable

SLC35D2 -0.098337448 8.981834825 -1.847088356 0.0723953 0.44762161 -5.328956253 stable

METTL18 0.27325787 8.621885268 1.847002974 0.072407954 0.44762161 -5.329103582 stable

TPRXL 0.017056474 2.288715963 1.846769517 0.07244256 0.44762161 -5.329506386 stable

PIK3C2A 0.138376471 8.935775627 1.846758212 0.072444237 0.44762161 -5.329525891 stable

LMTK3 -0.056193818 2.6327382 -1.846613087 0.072465757 0.44762161 -5.329776264 stable

SLC22A1 0.013345192 3.079464863 1.846484361 0.07248485 0.44762161 -5.329998333 stable

RP13-39P12.3 0.057984164 3.32705425 1.846462849 0.072488041 0.44762161 -5.330035442 stable

VARS -0.167677369 7.594686498 -1.846176017 0.072530602 0.44762161 -5.330530212 stable

TIMM50 -0.102599455 4.664030171 -1.846119833 0.072538941 0.44762161 -5.330627119 stable

RIN3 0.271160459 6.094678098 1.846078261 0.072545112 0.44762161 -5.330698821 stable

AXIN2 0.091222771 2.309754297 1.845850224 0.07257897 0.447681852 -5.33109211 stable

PEX11G -0.169297182 4.51972088 -1.845531689 0.072626286 0.447681852 -5.331641408 stable

HSPA1L 0.168329246 8.711370083 1.845451096 0.072638262 0.447681852 -5.331780375 stable

RBM19 0.185368959 6.580751138 1.845353243 0.072652805 0.447681852 -5.331949096 stable

FAM120A 0.129470843 5.036955215 1.845207686 0.072674442 0.447681852 -5.332200056 stable

PLOD1 0.23756576 9.034513917 1.845124221 0.072686852 0.447681852 -5.332343954 stable

WASIR1 /// WASIR1 /// WASIR2 /// WASIR2 -0.154955359 2.841448003 -1.845043624 0.072698837 0.447681852 -5.332482901 stable

DNAJC12 0.005281948 2.767628282 1.84494116 0.072714076 0.447681852 -5.33265954 stable

EPN1 -0.142613978 7.230002703 -1.844739925 0.072744012 0.447681852 -5.333006425 stable

LOC728099 -0.024896248 3.252724572 -1.844706922 0.072748923 0.447681852 -5.333063314 stable

SHB 0.054036469 3.170282863 1.844603985 0.072764241 0.447681852 -5.33324074 stable

SLU7 0.127134378 10.37573345 1.844460852 0.072785546 0.447695822 -5.333487436 stable

SLC25A33 -0.119396116 8.090462022 -1.843837797 0.072878346 0.448081069 -5.334561116 stable

ZNF770 0.114907746 10.72588481 1.843784565 0.072886279 0.448081069 -5.334652834 stable

CCDC92 -0.181994508 11.29939987 -1.843437806 0.072937975 0.448281711 -5.33525024 stable

CNNM3 0.133557425 9.446285442 1.843181622 0.072976188 0.448313661 -5.33569154 stable

LYPD6B -0.05729309 2.432202916 -1.8431474 0.072981294 0.448313661 -5.335750487 stable

HSD17B14 -0.018713166 2.114624846 -1.842806687 0.073032144 0.448324686 -5.336337308 stable

LOC102723927 -0.166128342 2.359269715 -1.84273027 0.073043553 0.448324686 -5.336468911 stable

SLC4A1AP 0.084235631 9.703186622 1.842723997 0.07304449 0.448324686 -5.336479715 stable

GTPBP4 -0.153549337 6.818881205 -1.842582478 0.073065623 0.448324686 -5.336723421 stable

CBFB 0.059257274 12.298961 1.842383685 0.073095318 0.448324686 -5.337065731 stable

AK026714 /// PELI1 -0.392336126 10.90311998 -1.842362737 0.073098448 0.448324686 -5.337101801 stable

SUGT1P1 0.017039159 2.724810644 1.842241838 0.073116513 0.448324686 -5.337309966 stable

FMR1 0.073378578 10.3979376 1.841684206 0.073199885 0.448568284 -5.338269952 stable

LGI4 -0.030075137 4.179837132 -1.841610861 0.073210857 0.448568284 -5.3383962 stable

GPR25 0.139624857 3.981881031 1.841593502 0.073213454 0.448568284 -5.338426079 stable

CLC -0.527516268 11.16085601 -1.840919309 0.073314376 0.449069648 -5.339586355 stable

LRMP -0.090005193 11.49625757 -1.84030849 0.073405915 0.449384614 -5.34063726 stable

TNNI3K 0.127907383 3.729610698 1.840266094 0.073412272 0.449384614 -5.34071019 stable

EFTUD2 0.034699345 2.691398944 1.840113338 0.073435181 0.449384614 -5.340972953 stable

ARL6IP5 0.047381457 13.69249721 1.840032385 0.073447324 0.449384614 -5.341112197 stable

PCIF1 0.095427904 8.881284706 1.839816479 0.073479719 0.449384614 -5.341483544 stable

SHANK2-AS3 -0.017025418 3.60767663 -1.83981173 0.073480431 0.449384614 -5.341491711 stable

STIP1 0.153201323 9.634779924 1.839599058 0.073512353 0.449461918 -5.341857458 stable

FOXN3-AS1 -0.028801066 3.069383721 -1.839472935 0.073531289 0.449461918 -5.342074345 stable

NME5 0.003641654 2.513199556 1.839085298 0.073589516 0.449562708 -5.342740862 stable

MTAP 0.115088988 4.833268215 1.838991193 0.073603657 0.449562708 -5.342902653 stable

KLHDC8B -0.252784044 7.762613632 -1.838932096 0.073612539 0.449562708 -5.343004251 stable

CHRNB3 0.002761417 2.185871085 1.838854307 0.073624232 0.449562708 -5.343137981 stable

ZNF35 0.227297599 5.866467136 1.838670526 0.073651862 0.449604931 -5.343453907 stable

PEAR1 -0.407187118 7.328702116 -1.838549724 0.073670028 0.449604931 -5.343661555 stable

BTG2 0.274452362 10.1645252 1.838426976 0.073688492 0.449604931 -5.343872537 stable

C14orf166 -0.05332661 12.62974422 -1.838257661 0.073713966 0.44964372 -5.344163539 stable

TBC1D8B -0.324614231 3.459557641 -1.83798809 0.073754539 0.449710341 -5.344626806 stable

SLC39A3 -0.103534013 8.974125837 -1.837741104 0.07379173 0.449710341 -5.345051209 stable

KRR1 0.081235159 9.852777139 1.837693223 0.073798941 0.449710341 -5.345133479 stable

KCTD21 0.2847022 7.226219028 1.837677129 0.073801365 0.449710341 -5.345161132 stable

LINC01138 0.177864694 8.948892467 1.837471884 0.073832286 0.449782234 -5.345513763 stable

ZNF160 0.195427526 7.580492158 1.837024559 0.073899716 0.45007644 -5.3462822 stable

MYCBP2 -0.102403545 11.15598665 -1.836845143 0.073926775 0.450096087 -5.346590365 stable

ACTL9 0.004492942 2.433921929 1.836749436 0.073941213 0.450096087 -5.346754741 stable

GCN1L1 -0.111933527 9.598787798 -1.836595721 0.073964407 0.450120783 -5.347018731 stable

ATF1 0.127446787 9.240716419 1.836208863 0.074022807 0.45034997 -5.347683037 stable

CHMP4C 0.007545477 2.76652637 1.836092633 0.074040361 0.45034997 -5.347882603 stable

TEAD3 0.058677754 3.438160339 1.835842483 0.074078152 0.450441128 -5.348312071 stable

TTC33 0.125595821 8.821170673 1.835679512 0.074102782 0.450441128 -5.34859184 stable

MYL9 -0.569818348 10.36494377 -1.835613236 0.0741128 0.450441128 -5.348705608 stable

ATXN7L1 -0.021989313 2.673564502 -1.835444412 0.074138324 0.450479856 -5.348995395 stable

DIP2A 0.325290466 8.278175383 1.835034405 0.074200344 0.450740259 -5.349699078 stable

ABCD3 0.131281799 8.526845057 1.834720431 0.074247867 0.450802595 -5.350237855 stable

CINP 0.288644975 4.25547258 1.834448504 0.074289046 0.450802595 -5.350704416 stable

LINC00463 -0.059185233 3.428724067 -1.834371495 0.074300712 0.450802595 -5.350836535 stable

MYO15A 0.045907645 2.960359249 1.83429197 0.07431276 0.450802595 -5.350972965 stable

C19orf45 -0.00753425 2.682039085 -1.834232402 0.074321786 0.450802595 -5.351075153 stable

CA10 0.008757549 2.732472331 1.834202502 0.074326316 0.450802595 -5.351126446 stable

PKIB 0.114327572 3.315459998 1.834080758 0.074344767 0.450802595 -5.351335287 stable

SEC24B 0.082918363 11.21775628 1.833551497 0.074425023 0.451064038 -5.352243053 stable

C11orf31 -0.114399013 6.866776904 -1.833543492 0.074426237 0.451064038 -5.352256781 stable

GDF5 -0.05198029 3.001743737 -1.833116907 0.074490978 0.4512117 -5.352988278 stable

PRRC1 0.124768173 8.659262886 1.832719378 0.074551352 0.4512117 -5.353669823 stable

GALNS -0.095904152 9.349301405 -1.832715267 0.074551977 0.4512117 -5.353676871 stable

PTPRM 0.274856305 5.381123816 1.832683763 0.074556763 0.4512117 -5.353730877 stable

RP11-305O4.3 0.017683549 3.18575591 1.832364227 0.074605326 0.4512117 -5.354278608 stable

ZNF813 0.084287194 6.964966892 1.832294729 0.074615891 0.4512117 -5.354397727 stable

C22orf39 -0.110932822 9.43522524 -1.831917591 0.074673249 0.4512117 -5.355044072 stable

ACTL6A 0.096656375 9.841154622 1.831873231 0.074679998 0.4512117 -5.35512009 stable

BC062753 0.113188159 9.251090446 1.83185516 0.074682747 0.4512117 -5.355151055 stable

PTGS2 1.120905619 6.664545797 1.831794375 0.074691997 0.4512117 -5.355255217 stable

CRLF2 -0.021344643 2.641703284 -1.831419426 0.074749071 0.4512117 -5.355897664 stable

SIGMAR1 -0.10047931 8.099361522 -1.831357331 0.074758526 0.4512117 -5.356004048 stable

EID3 0.233709019 7.146865851 1.831310302 0.074765688 0.4512117 -5.356084618 stable

CHRNB2 -0.150540753 8.56762468 -1.831302837 0.074766825 0.4512117 -5.356097407 stable

ZC3HAV1 -0.145137833 12.16323573 -1.831041087 0.074806698 0.4512117 -5.356545807 stable

APCDD1L -0.015287539 3.398268301 -1.831038965 0.074807022 0.4512117 -5.356549441 stable

OXER1 0.077821764 4.712352719 1.830990425 0.074814418 0.4512117 -5.356632589 stable

SNORD116-17 /// SNORD116-19 /// SNORD116-20 /// SNORD116-21 /// SNORD116@ -0.089419019 3.601205608 -1.830922958 0.074824699 0.4512117 -5.356748153 stable

PTS -0.087387934 8.872146683 -1.830875383 0.07483195 0.4512117 -5.356829644 stable

LINC00599 /// MIR124-1 -0.037771459 2.269913368 -1.830655608 0.074865452 0.4512117 -5.357206066 stable

LOC101928068 -0.154163994 3.480094486 -1.830639188 0.074867956 0.4512117 -5.357234187 stable

LOC101927257 0.050458839 2.743711461 1.830608497 0.074872636 0.4512117 -5.357286751 stable

REG1A 0.027526099 2.665266772 1.830318322 0.074916893 0.451362766 -5.357783682 stable

LOC101060747 /// PDPK1 -0.133964488 9.314193318 -1.830165404 0.074940225 0.451387716 -5.358045532 stable

PRKCSH -0.087944317 11.00028851 -1.830014637 0.074963235 0.451410712 -5.35830368 stable

UBE2R2 -0.084543369 11.73091127 -1.829714121 0.075009117 0.451571393 -5.358818181 stable

NOL6 -0.0797873 8.625851528 -1.829388474 0.075058863 0.451678131 -5.359375626 stable

RPS6KL1 -0.080805435 2.552283048 -1.829245041 0.075080782 0.451678131 -5.35962113 stable

AC083843.1 0.067223421 2.80080005 1.829221004 0.075084456 0.451678131 -5.359662271 stable

COL9A2 -0.308338141 5.082701111 -1.828772948 0.075152967 0.451888866 -5.360429062 stable

AEN -0.108896599 7.261216756 -1.828740621 0.075157912 0.451888866 -5.36048438 stable

CD38 0.433256745 8.490688558 1.828521972 0.075191366 0.451974474 -5.360858508 stable

CYBA 0.111567932 13.81876841 1.828293339 0.075226361 0.452069297 -5.361249677 stable

ICE1 0.070169229 10.73661068 1.828022147 0.075267888 0.452203319 -5.361713611 stable

C5orf42 0.11724407 3.967826709 1.82768653 0.075319307 0.452319391 -5.362287676 stable

ARL16 -0.150274856 8.991978452 -1.827586213 0.075334682 0.452319391 -5.362459249 stable

FXYD3 -0.128257471 3.201147066 -1.827372158 0.075367499 0.452319391 -5.362825321 stable

FHDC1 0.098002761 2.486903861 1.82715574 0.075400689 0.452319391 -5.363195398 stable

PTPRA 0.100384554 9.377884112 1.827118444 0.075406411 0.452319391 -5.363259172 stable

GSPT2 0.216448095 8.846941185 1.826819369 0.075452301 0.452319391 -5.363770524 stable

RP11-135A1.2 -0.052555454 2.939228283 -1.826599225 0.075486096 0.452319391 -5.364146878 stable

RAB11FIP1 0.16166385 10.77965332 1.826599123 0.075486112 0.452319391 -5.364147051 stable

ZBTB43 0.193367766 5.74690912 1.826481391 0.07550419 0.452319391 -5.364348309 stable

MAGED4 /// MAGED4B /// SNORA11D /// SNORA11E -0.333391968 3.298086337 -1.826418577 0.075513837 0.452319391 -5.36445568 stable

MBP 0.309469256 10.48790678 1.826389445 0.075518312 0.452319391 -5.364505477 stable

CLGN 0.012232037 3.090289344 1.826375558 0.075520445 0.452319391 -5.364529214 stable

GPR160 -0.191318264 10.12657801 -1.826266459 0.075537204 0.452319391 -5.364715694 stable

SLC2A14 /// SLC2A3 0.180487165 10.23104265 1.825903818 0.075592933 0.452331301 -5.365335479 stable

REEP2 -0.07396391 3.598881277 -1.825886552 0.075595587 0.452331301 -5.365364985 stable

FAM102B 0.113183235 9.267515751 1.825861337 0.075599464 0.452331301 -5.365408076 stable

PDE5A -0.345202886 7.338026068 -1.825614455 0.075637427 0.452331301 -5.365829952 stable

ARL14 0.008679513 2.881584644 1.82541561 0.075668015 0.452331301 -5.366169706 stable

RP3-507I15.1 -0.068344216 11.72812101 -1.825389351 0.075672055 0.452331301 -5.36621457 stable

CREB3L3 0.013702663 2.975330488 1.825377947 0.075673809 0.452331301 -5.366234055 stable

FAM65B -0.133274423 12.30057466 -1.824867464 0.075752389 0.45268596 -5.367106128 stable

PKDCC -0.020127292 2.281742744 -1.824714104 0.075776009 0.452699059 -5.367368078 stable

RP11-533E19.7 -0.133868093 4.561737714 -1.824520182 0.075805886 0.452699059 -5.367699283 stable

HMGN2 -0.045780207 13.98428562 -1.824478427 0.075812321 0.452699059 -5.367770594 stable

NNMT 0.025711125 2.715355938 1.824116664 0.075868087 0.452917073 -5.36838837 stable

SOCS6 0.128903511 3.928403447 1.823719808 0.075929302 0.453167501 -5.369065955 stable

ZNF30 0.224952829 7.559595032 1.823571064 0.075952257 0.453189508 -5.369319886 stable

CEP70 0.009007746 2.989139812 1.823429998 0.075974033 0.453204469 -5.369560694 stable

ARHGEF26 -0.01670952 3.43707835 -1.823201716 0.076009282 0.453296845 -5.369950351 stable

PIM3 -0.150532346 10.71084843 -1.823039069 0.076034405 0.453296845 -5.370227949 stable

TADA2A 0.14718446 5.675793921 1.822955383 0.076047334 0.453296845 -5.370370772 stable

AX747730 0.241051199 5.513371263 1.822782708 0.076074018 0.453341013 -5.370665453 stable

MYZAP -0.542511963 7.237975571 -1.822303667 0.076148086 0.45366746 -5.37148284 stable

CKAP2 /// IGLC1 /// IGLJ2 /// IGLJ2 /// IGLJ3 /// IGLJ3 /// IGLJ3 /// IGLV1-44 /// IGLV2-14 /// IGLV@ -0.389825068 10.69414921 -1.822128488 0.076175187 0.453713997 -5.371781703 stable

SNRPA1 -0.141567183 10.80120774 -1.821687429 0.076243457 0.453848286 -5.372534062 stable

LINC00290 0.032201156 3.31186126 1.821626021 0.076252966 0.453848286 -5.3726388 stable

C1orf177 0.096688689 2.531606156 1.821608889 0.07625562 0.453848286 -5.37266802 stable

GALNT11 0.107575157 10.05362142 1.821467879 0.07627746 0.45385277 -5.372908514 stable

LOC728353 -0.028583653 2.83871972 -1.821354889 0.076294964 0.45385277 -5.373101209 stable

EFNA1 0.286162214 2.837791293 1.820890357 0.076366965 0.454067964 -5.373893321 stable

ZNF19 0.18569008 3.438479355 1.820872401 0.076369749 0.454067964 -5.373923935 stable

TRAK2 -0.130034239 9.977359157 -1.820438929 0.07643699 0.454141681 -5.374662925 stable

USB1 0.139871559 9.537121051 1.820390573 0.076444494 0.454141681 -5.374745354 stable

MIR4296 0.10806356 3.073857027 1.820333834 0.0764533 0.454141681 -5.374842071 stable

TXNDC11 -0.130748016 9.37068899 -1.820294664 0.076459379 0.454141681 -5.374908838 stable

C5orf34 0.160892051 4.181714644 1.81997268 0.07650937 0.454323883 -5.375457629 stable

JAG1 -0.027448687 3.653172791 -1.819175456 0.076633266 0.454778871 -5.37681607 stable

CRAT -0.124999212 7.218592943 -1.819119895 0.076641908 0.454778871 -5.376910726 stable

RAB10 0.102484128 12.43005926 1.818979381 0.076663764 0.454778871 -5.377150098 stable

CTNNBIP1 -0.126789298 8.459880411 -1.818919573 0.076673069 0.454778871 -5.37725198 stable

RP11-255A11.4 0.032519518 2.970734674 1.818857889 0.076682667 0.454778871 -5.377357053 stable

ACSL5 0.060354783 9.981427175 1.818414621 0.076751666 0.455073339 -5.37811204 stable

BC036261 /// GS1-18A18.2 -0.008289403 2.959521272 -1.817701578 0.076862769 0.455489255 -5.379326187 stable

TWISTNB 0.140802752 5.441459044 1.817509475 0.076892724 0.455489255 -5.379653226 stable

DLEC1 0.093477015 3.110688623 1.817479455 0.076897406 0.455489255 -5.37970433 stable

EVX1 -0.04687663 4.96571251 -1.817467479 0.076899274 0.455489255 -5.379724716 stable

DGCR5 -0.009145404 2.528149762 -1.817308412 0.076924088 0.455521518 -5.379995487 stable

NSUN5P1 -0.195479673 10.75580284 -1.817159928 0.076947257 0.455544029 -5.380248225 stable

ARHGAP10 -0.101021008 6.296112795 -1.816934536 0.076982437 0.455545837 -5.380631836 stable

HIST1H2BG /// HIST1H2BJ -0.440915832 8.344303569 -1.81690981 0.076986297 0.455545837 -5.380673918 stable

KCNJ2 0.856898553 5.363888802 1.816675738 0.077022849 0.455647491 -5.381072255 stable

RP11-334J6.6 -0.089096864 3.117005299 -1.816373634 0.077070045 0.455718625 -5.381586303 stable

LOC401913 -0.067678647 3.453432655 -1.816148145 0.077105288 0.455718625 -5.381969939 stable

KCNAB2 0.15027075 11.04044879 1.815967466 0.077133538 0.455718625 -5.382277309 stable

ARFGEF2 0.015783632 2.021558756 1.815928798 0.077139585 0.455718625 -5.382343088 stable

RBM33 0.170382328 5.416840733 1.81585872 0.077150544 0.455718625 -5.382462295 stable

ENOX1 0.009695663 2.663006435 1.815855021 0.077151123 0.455718625 -5.382468588 stable

SRPX 0.027982984 2.640858982 1.815709314 0.077173915 0.455738805 -5.382716431 stable

IFITM3 0.163050935 13.26106844 1.815253671 0.077245226 0.456045422 -5.383491361 stable

RRBP1 0.136901631 7.664998976 1.81510495 0.077268514 0.456065274 -5.383744261 stable

DUSP5 0.278788926 9.402771177 1.814865489 0.077306023 0.456065274 -5.38415143 stable

ARHGEF18 -0.106948799 12.50590111 -1.814860799 0.077306758 0.456065274 -5.384159404 stable

DTNA 0.003234824 2.437471023 1.814591155 0.077349013 0.456200135 -5.384617838 stable

NUDC -0.071395149 9.782471417 -1.814185442 0.077412629 0.456301187 -5.385307499 stable

LINC01118 -0.061915087 2.955616466 -1.814098084 0.077426332 0.456301187 -5.38545598 stable

FIG4 -0.127485022 9.852427234 -1.813998079 0.077442022 0.456301187 -5.385625949 stable

MAPKAPK5-AS1 -0.120254803 9.844409371 -1.81387393 0.077461504 0.456301187 -5.385836944 stable

ZIC5 0.007962469 2.751938296 1.813863471 0.077463145 0.456301187 -5.385854718 stable

C21orf91-OT1 0.147947898 5.018179806 1.813724022 0.077485033 0.45631584 -5.386091699 stable

IGHA1 /// IGHG1 /// IGHG3 /// IGHM /// IGHV4-31 /// LOC100293211 -0.241323539 5.14663621 -1.813240035 0.07756104 0.456649118 -5.386914072 stable

WDR59 -0.111613352 10.06955284 -1.813078916 0.077586357 0.456683859 -5.387187799 stable

FAM192A -0.083281227 9.251082138 -1.812901956 0.077614171 0.456733278 -5.387488415 stable

ABCG1 0.219032948 8.574294048 1.812668348 0.077650901 0.456792029 -5.387885224 stable

ACSF2 -0.120068711 7.557953688 -1.812591446 0.077662996 0.456792029 -5.388015841 stable

LYPLA1 -0.061011897 11.55533783 -1.812389878 0.077694705 0.456864286 -5.38835818 stable

SLITRK2 -0.023576498 2.915096892 -1.811971117 0.077760615 0.45707474 -5.389069294 stable

RAMP3 -0.05967784 1.8298546 -1.811813542 0.077785429 0.45707474 -5.389336841 stable

SNRNP70 -0.138580174 11.21115513 -1.811765657 0.077792971 0.45707474 -5.389418142 stable

CLPP -0.085995818 8.974143652 -1.811352586 0.077858055 0.45707474 -5.390119391 stable

TYK2 -0.093863498 10.66807669 -1.811349325 0.077858569 0.45707474 -5.390124925 stable

PCDHGB6 0.016541918 2.75133419 1.811316723 0.077863708 0.45707474 -5.390180266 stable

LOC100506999 0.014939076 3.495381162 1.81124517 0.077874987 0.45707474 -5.390301723 stable

CNPPD1 -0.070741678 10.40315136 -1.810794259 0.077946099 0.45707474 -5.391067018 stable

BOLA2 /// LOC101060386 /// LOC101060596 /// LOC101060604 /// LOC102723773 /// LOC613037 /// NPIPA5 /// NPIPB4 /// NPIPB5 /// SLC7A5P1 /// SMG1 /// SMG1P1 /// SMG1P2 /// SMG1P3 /// SMG1P5 /// SMG1P7 0.197561297 8.758201471 1.810777645 0.077948721 0.45707474 -5.391095213 stable

C14orf142 0.149583336 9.112905091 1.810727863 0.077956575 0.45707474 -5.391179692 stable

ARPC5 0.09548028 13.23128474 1.810621606 0.077973343 0.45707474 -5.391360006 stable

YIPF4 0.109875954 6.808343764 1.810450247 0.07800039 0.45707474 -5.391650774 stable

INTS2 0.150315342 7.652422841 1.810428998 0.078003744 0.45707474 -5.391686828 stable

SERINC1 0.106874905 13.02331976 1.81023713 0.078034039 0.45707474 -5.392012367 stable

PIAS1 -0.073365233 11.12906806 -1.810213215 0.078037815 0.45707474 -5.392052941 stable

CSF3 -0.012794704 3.414240754 -1.810190414 0.078041416 0.45707474 -5.392091625 stable

ERMARD -0.093100547 9.229446706 -1.809856449 0.078094175 0.45726104 -5.392658174 stable

NBPF1 -0.103155465 12.2758149 -1.809742823 0.078112132 0.45726104 -5.392850912 stable

LOC100652770 0.012232731 2.314023602 1.809619998 0.078131547 0.45726104 -5.393059244 stable

POLH 0.006412759 1.854345136 1.809168508 0.078202949 0.457425488 -5.393824943 stable

MICA 0.150478416 7.899627088 1.809143719 0.078206871 0.457425488 -5.393866977 stable

ZNF462 -0.093688171 2.837145344 -1.808730978 0.078272196 0.457425488 -5.394566812 stable

C10orf113 0.020600754 3.031413013 1.808586216 0.078295118 0.457425488 -5.394812235 stable

IFT122 /// LOC101927266 -0.140616556 6.886778572 -1.808531929 0.078303716 0.457425488 -5.394904266 stable

RASGRF1 -0.057908497 3.0468738 -1.808509313 0.078307298 0.457425488 -5.394942607 stable

EXOSC2 -0.141674676 7.946124554 -1.808353683 0.078331951 0.457425488 -5.395206427 stable

XPO4 0.117525859 7.021879418 1.808170303 0.078361008 0.457425488 -5.395517263 stable

GCK 0.009720781 2.240156991 1.808117309 0.078369407 0.457425488 -5.395607086 stable

LINC00868 -0.06813093 2.840798344 -1.807956739 0.078394859 0.457425488 -5.39587923 stable

LINC00857 -0.015690469 2.836247154 -1.807924444 0.07839998 0.457425488 -5.395933963 stable

ZNF316 -0.107089975 7.986549755 -1.807837945 0.078413694 0.457425488 -5.396080556 stable

OR5K1 0.006578522 2.549979659 1.807820374 0.078416481 0.457425488 -5.396110334 stable

IGH /// IGHA1 /// IGHD /// IGHG1 /// IGHG3 /// IGHM /// IGHV3-23 /// IGHV4-31 -0.397116299 10.26697192 -1.807723072 0.078431912 0.457425488 -5.396275228 stable

AP1S3 0.018805317 3.282905657 1.807527789 0.078462889 0.457492713 -5.396606143 stable

HIST1H3E 0.013497594 1.950673698 1.807286349 0.078501202 0.457602668 -5.397015231 stable

B4GALT7 -0.108376155 7.461145424 -1.806817824 0.078575595 0.45783059 -5.397808953 stable

PVRL3 0.02604899 2.676447225 1.806794934 0.078579231 0.45783059 -5.397847726 stable

CTAGE15 /// CTAGE4 /// CTAGE8 /// CTAGE9 -0.125862995 7.860519913 -1.806066123 0.078695076 0.45819506 -5.399082036 stable

FAM99B -0.049510232 3.420076257 -1.806042296 0.078698866 0.45819506 -5.399122382 stable

UBR7 0.082683462 9.504215081 1.805923999 0.078717683 0.45819506 -5.399322688 stable

ELFN2 0.004605017 2.324464211 1.805793842 0.078738392 0.45819506 -5.399543063 stable

UVRAG -0.080253513 10.29975701 -1.805788843 0.078739188 0.45819506 -5.399551527 stable

ANKRD20A1 /// ANKRD20A2 /// ANKRD20A3 /// ANKRD20A4 0.036564986 3.235869293 1.805595792 0.078769912 0.458260475 -5.399878364 stable

RP4-758J24.5 -0.097270735 4.944486236 -1.805423086 0.078797407 0.458307074 -5.400170732 stable

RP5-1074L1.4 -0.235478228 7.585753484 -1.805002829 0.078864346 0.458583013 -5.400882074 stable

RP11-250B2.3 0.006684407 3.087234436 1.803644646 0.079081009 0.459559731 -5.403180024 stable

SLC24A1 0.159865026 5.090091168 1.803537903 0.079098058 0.459559731 -5.403360564 stable

SPATA9 0.01777027 2.622369927 1.803464667 0.079109757 0.459559731 -5.403484427 stable

ZBTB18 0.137569885 8.93747597 1.803460208 0.079110469 0.459559731 -5.403491967 stable

CPNE4 -0.037225152 2.840019099 -1.803312163 0.079134124 0.459583637 -5.40374234 stable

HBQ1 -0.079294067 2.141927709 -1.802960856 0.079190279 0.459796239 -5.404336396 stable

MTFR1 0.155114686 7.360790797 1.802672841 0.079236343 0.459950154 -5.404823355 stable

UBE2Q2L 0.053548884 6.141402414 1.802396539 0.079280554 0.459993487 -5.405290447 stable

SLC25A15 0.194156513 7.713413871 1.802381749 0.079282922 0.459993487 -5.405315448 stable

MDFI -0.036944551 3.821144688 -1.801632308 0.079402949 0.460576266 -5.406582077 stable

BMS1P20 -0.442517007 8.592965168 -1.801292205 0.079457469 0.460778876 -5.407156737 stable

STON1 -0.216648154 3.63593903 -1.800574224 0.079572668 0.461333183 -5.408369584 stable

SMIM10 0.010688716 2.988487916 1.800255519 0.079623849 0.46143394 -5.408907825 stable

BC043266 /// RP4-568F9.6 -0.01174214 3.447409507 -1.800221693 0.079629283 0.46143394 -5.408964947 stable

LOC652993 -0.02741334 2.490547694 -1.799814636 0.079694697 0.461656366 -5.409652273 stable

SRSF7 0.136170993 9.715815389 1.79966941 0.079718045 0.461656366 -5.409897457 stable

CNIH3 0.010396085 2.907482588 1.799616528 0.079726549 0.461656366 -5.409986735 stable

CC2D1B 0.129977845 8.717565403 1.799295743 0.079778148 0.461841453 -5.410528244 stable

PTTG3P -0.093025476 3.445330414 -1.798730482 0.079869141 0.462254446 -5.411482251 stable

RBM48 0.131641801 4.065489174 1.798458789 0.079912908 0.462393975 -5.411940704 stable

HTRA2 -0.085535347 8.933577645 -1.796888002 0.080166342 0.463746321 -5.414590098 stable

DHX33 -0.090323663 8.907402249 -1.796600748 0.080212762 0.463757269 -5.415074388 stable

MED29 -0.079261035 9.78863776 -1.796437421 0.080239165 0.463757269 -5.415349717 stable

SPATA33 0.130582481 3.31024101 1.796404333 0.080244515 0.463757269 -5.415405493 stable

KCNK12 0.004493463 2.379149929 1.796081703 0.080296696 0.463757269 -5.415949296 stable

RNF170 0.059013076 2.746317343 1.795995151 0.080310699 0.463757269 -5.416095167 stable

AGER -0.16532956 7.468246555 -1.795947133 0.080318469 0.463757269 -5.416176093 stable

C7orf55-LUC7L2 /// LUC7L2 0.074509784 8.896014645 1.795941072 0.08031945 0.463757269 -5.416186308 stable

LINC00304 0.023909642 2.310769717 1.795786239 0.080344508 0.463757269 -5.416447237 stable

MIR6845 /// NRBP2 -0.157496695 8.651801173 -1.795614354 0.080372334 0.463757269 -5.416736881 stable

DBH 0.022600024 2.576647289 1.795561957 0.080380818 0.463757269 -5.416825172 stable

RPL6 0.031939476 15.39471658 1.795535392 0.080385119 0.463757269 -5.416869932 stable

SLC46A3 -0.039805761 9.167433367 -1.795039271 0.08046549 0.464107109 -5.417705785 stable

LOC101929460 0.028697631 3.492412767 1.794828633 0.080499634 0.464190214 -5.418060603 stable

BRPF1 0.089723662 9.041911254 1.79405682 0.080624846 0.464798284 -5.419360418 stable

LINC00882 0.005550978 2.59461754 1.79361134 0.080697191 0.465083782 -5.420110438 stable

PAIP2 -0.055441276 2.349699645 -1.793302947 0.080747306 0.465083782 -5.420629563 stable

EPHX4 0.054753038 2.946463682 1.793288252 0.080749695 0.465083782 -5.420654298 stable

AP3B2 -0.006919471 3.165330836 -1.79326508 0.080753461 0.465083782 -5.4206933 stable

UBE2T 0.204174685 6.182099124 1.792944665 0.080805561 0.465269915 -5.421232574 stable

RP11-573N10.1 0.004214842 2.409208539 1.792752336 0.080836847 0.465336145 -5.421556232 stable

TMOD2 0.183970347 3.696701808 1.792416151 0.08089156 0.46553716 -5.422121908 stable

IRF9 0.101400317 11.95404804 1.79213979 0.080936559 0.465682194 -5.422586855 stable

MOBP -0.004065941 2.352351172 -1.791881237 0.080978678 0.465810587 -5.423021786 stable

TMA16 0.062386086 3.804611806 1.791730713 0.081003207 0.465837761 -5.423274969 stable

HAUS6 0.134106481 6.624520052 1.791460452 0.081047265 0.46588827 -5.423729508 stable

CCDC77 0.109182717 6.698217079 1.79140518 0.081056277 0.46588827 -5.423822459 stable

NOX1 0.007073209 2.553454986 1.791222921 0.081086003 0.46588827 -5.42412895 stable

ACTR1A 0.15458793 9.935440914 1.791090241 0.081107648 0.46588827 -5.424352052 stable

GSAP 0.136682934 11.81977498 1.791069534 0.081111027 0.46588827 -5.424386869 stable

AC016999.2 -0.006949843 2.700750343 -1.790140044 0.081262805 0.466646103 -5.425949398 stable

HN1 0.131645412 10.40217673 1.78961848 0.081348077 0.467021752 -5.426825878 stable

SENP8 0.009465791 2.785539597 1.789258887 0.081406912 0.46724548 -5.427430044 stable

AC068039.4 -0.115934985 3.848764905 -1.789013034 0.081447158 0.467362429 -5.42784305 stable

MRPS21 -0.071574571 10.81929426 -1.788732441 0.081493111 0.467512065 -5.428314358 stable

RP3-508I15.21 -0.112050182 6.196996415 -1.788495834 0.081531878 0.467620408 -5.428711734 stable

LRRN1 0.128983996 3.401030482 1.788161922 0.081586614 0.467820268 -5.429272458 stable

BMPR2 0.146142507 7.127853389 1.788029261 0.081608368 0.467830961 -5.429495205 stable

ETFDH 0.167165726 6.852382029 1.787689446 0.081664116 0.467865148 -5.430065714 stable

MGAM 0.633741492 5.921337638 1.787642143 0.081671879 0.467865148 -5.430145122 stable

SLC35A5 0.192408436 9.465251398 1.787629182 0.081674006 0.467865148 -5.430166879 stable

FLJ38717 -0.230126265 8.118161779 -1.786965071 0.08178306 0.468195134 -5.431281546 stable

ANKH -0.025236933 2.6548291 -1.786965008 0.08178307 0.468195134 -5.431281652 stable

FASTK -0.048811012 8.825799034 -1.786914755 0.081791327 0.468195134 -5.431365984 stable

DGCR6 /// DGCR6L 0.110441342 9.459542306 1.786445215 0.081868511 0.468522929 -5.432153845 stable

MEG3 0.005903515 2.932353016 1.785698009 0.081991464 0.469112437 -5.433407253 stable

LOC101927933 /// LRRC8C 0.247651641 8.757647334 1.785571463 0.082012303 0.469117552 -5.433619485 stable

SCPEP1 -0.174027212 5.279845137 -1.785421206 0.082037052 0.469145027 -5.433871467 stable

TRIM59 0.156648463 8.29344741 1.785044409 0.082099142 0.469385981 -5.434503278 stable

ZHX1 0.117150605 8.415458771 1.784866714 0.082128437 0.469439362 -5.434801197 stable

DNAJB5 0.00458446 2.506662056 1.784719654 0.082152689 0.469463894 -5.435047734 stable

ETFA -0.091149917 11.84685468 -1.783737077 0.082314878 0.470276472 -5.436694528 stable

NHLH1 -0.03119941 2.6628126 -1.783407567 0.082369328 0.470473282 -5.437246612 stable

CGB1 /// LHB -0.055292512 3.933147581 -1.783221749 0.082400048 0.470474146 -5.437557906 stable

CDKN2AIP 0.127395354 10.01891508 1.783164681 0.082409484 0.470474146 -5.437653506 stable

AX747826 -0.627498116 7.893514583 -1.782754963 0.08247726 0.470746815 -5.438339778 stable

GYPE 0.011094627 2.983205251 1.782542088 0.082512492 0.470833653 -5.438696287 stable

NEK2 0.08514685 3.243610144 1.782160408 0.082575694 0.471080014 -5.439335412 stable

UGT8 -0.204935445 3.500158268 -1.782013419 0.082600045 0.471104668 -5.439581514 stable

NID2 -0.063501129 2.783304523 -1.78166533 0.082657734 0.471319412 -5.440164248 stable

IGLV1-44 -0.274237543 12.89294705 -1.780962676 0.082774291 0.471752529 -5.441340263 stable

RP11-524D16__A.3 0.004844849 2.662830437 1.780949187 0.08277653 0.471752529 -5.441362836 stable

LOC100506446 -0.043597638 2.554959987 -1.780752307 0.082809214 0.471752529 -5.441692276 stable

ATRAID -0.073696055 11.16435192 -1.780723967 0.082813919 0.471752529 -5.441739695 stable

C1QBP -0.076650582 11.91450811 -1.780179484 0.08290437 0.472003608 -5.44265061 stable

ATP5J -0.0611278 12.4474233 -1.780059738 0.082924273 0.472003608 -5.442850912 stable

LOC101928100 0.028647881 3.532252578 1.780040354 0.082927495 0.472003608 -5.442883335 stable

SOCS2 0.185914024 7.710956821 1.779975577 0.082938264 0.472003608 -5.442991683 stable

TULP3 0.212298747 6.97399674 1.779658468 0.082990999 0.472189476 -5.443522043 stable

EDEM3 0.089323262 9.23105459 1.779430317 0.083028958 0.472291203 -5.443903573 stable

DQ570096 0.224455299 7.146261255 1.779211607 0.08306536 0.472384026 -5.444269275 stable

CORO1A 0.060122877 13.86525462 1.779006993 0.083099428 0.472463535 -5.444611374 stable

CD81 0.107354503 12.39857904 1.778881994 0.083120246 0.47246769 -5.444820346 stable

BGN -0.058705141 3.898903681 -1.77859008 0.08316888 0.472629915 -5.445308316 stable

CKAP2 /// IGLC1 /// IGLV1-40 /// IGLV1-40 /// IGLV1-50 /// IGLV1-50 -0.349548057 4.305692048 -1.77846004 0.083190552 0.472638885 -5.445525672 stable

CRYBB3 -0.078274698 2.371195296 -1.778309434 0.083215659 0.472667353 -5.445777386 stable

CD53 0.039723641 14.09107675 1.778126348 0.083246188 0.472702881 -5.446083363 stable

PHB2 -0.076774406 13.07284749 -1.7780309 0.083262108 0.472702881 -5.446242865 stable

SARS 0.083392531 11.00714488 1.777727249 0.08331277 0.472876367 -5.446750248 stable

PDZD11 0.13743537 9.022519692 1.777516861 0.083347887 0.472885829 -5.447101751 stable

DNAJB7 0.019332221 3.102321058 1.777340134 0.083377396 0.472885829 -5.447396988 stable

UCN -0.070136877 2.604908657 -1.777301702 0.083383814 0.472885829 -5.447461189 stable

KDM4A 0.157513764 7.563495893 1.777235585 0.083394856 0.472885829 -5.447571635 stable

LOC101928464 -0.090275487 6.916612683 -1.776854654 0.083458502 0.473132664 -5.448207895 stable

C7orf71 0.00771496 3.024516339 1.776673459 0.08348879 0.473190321 -5.4485105 stable

WSB1 0.188617669 9.581047508 1.776526161 0.083513419 0.473215882 -5.448756476 stable

ATXN7L3B 0.137766391 7.760914864 1.775991988 0.083602786 0.473597241 -5.449648359 stable

LOC100507600 -0.030014607 3.294686645 -1.775883229 0.083620991 0.473597241 -5.44982992 stable

ZNF121 0.095854385 10.36076562 1.775672239 0.083656319 0.473683263 -5.450182119 stable

CLUH -0.096667201 8.084730374 -1.775348034 0.083710627 0.473876692 -5.450723234 stable

VRTN -0.037291407 6.212950338 -1.775112467 0.083750106 0.473886018 -5.451116356 stable

LOC100288778 /// LOC101930154 /// MIR6859-1 /// MIR6859-2 /// WASH1 /// WASH2P /// WASH3P /// WASH7P -0.124975286 11.77294993 -1.775097771 0.083752569 0.473886018 -5.451140879 stable

RP11-63A1.2 0.005511269 2.822131458 1.77461367 0.083833751 0.474231279 -5.451948617 stable

WIBG -0.049175167 2.903973376 -1.774011939 0.083934751 0.474688455 -5.452952364 stable

NSUN5P1 /// NSUN5P2 -0.198063249 10.44865848 -1.773807989 0.083969007 0.474768034 -5.453292507 stable

TIMP3 -0.023133297 2.707462887 -1.773447925 0.084029514 0.474928783 -5.453892934 stable

RNF144A 0.010479516 2.695928212 1.77337753 0.084041347 0.474928783 -5.454010309 stable

HMBS -0.125528814 7.906035987 -1.773278406 0.084058013 0.474928783 -5.45417558 stable

CTD-2012K14.6 -0.046823732 3.211143268 -1.772490963 0.084190503 0.475483867 -5.455488216 stable

IGHA1 /// IGHV4-31 -0.130567694 3.024781667 -1.772449716 0.084197448 0.475483867 -5.45555696 stable

PKIA -0.310477069 9.104205306 -1.772232237 0.084234073 0.475483867 -5.455919395 stable

LOC100507073 0.013955694 2.695023309 1.772214152 0.084237119 0.475483867 -5.455949532 stable

SPHKAP 0.003274272 2.259099151 1.771990754 0.084274756 0.475582181 -5.456321788 stable

MIR646HG 0.350955384 4.499444265 1.771558082 0.08434769 0.475705135 -5.457042652 stable

MRPL27 0.139595286 9.762078666 1.771519832 0.084354141 0.475705135 -5.457106373 stable

DUX1 -0.091633976 3.627222533 -1.771112806 0.084422805 0.475705135 -5.457784359 stable

DHRS13 0.154979827 8.407990027 1.77097213 0.084446548 0.475705135 -5.458018653 stable

TMEM117 0.09003687 3.721945673 1.770963347 0.08444803 0.475705135 -5.45803328 stable

CCL20 0.004076936 2.441778394 1.770903513 0.08445813 0.475705135 -5.458132928 stable

TNIP3 0.01998316 3.050836441 1.770830694 0.084470424 0.475705135 -5.458254198 stable

CNTNAP3P2 0.004755375 2.695345706 1.770787096 0.084477785 0.475705135 -5.458326802 stable

ZNF268 0.184476282 6.878583256 1.770782473 0.084478566 0.475705135 -5.4583345 stable

EIF4A3 0.102752269 10.73192019 1.770560167 0.084516109 0.475802633 -5.458704683 stable

OLFM2 0.12300704 4.716197793 1.770358708 0.084550144 0.475880337 -5.459040117 stable

MPL -0.532016109 8.332700863 -1.770177441 0.084580777 0.475938864 -5.459341903 stable

GBP5 0.307723794 10.26939785 1.769909335 0.084626103 0.476080019 -5.459788217 stable

ZFR2 0.005896604 2.425334173 1.769442154 0.084705132 0.476182552 -5.460565794 stable

RNF185 0.134678318 7.978289767 1.7693147 0.084726704 0.476182552 -5.460777898 stable

DLL4 -0.084069086 4.902452686 -1.769171475 0.08475095 0.476182552 -5.461016232 stable

RAB9B 0.029411301 2.892134105 1.769155127 0.084753718 0.476182552 -5.461043434 stable

ATG13 0.059800108 9.151215039 1.769140902 0.084756126 0.476182552 -5.461067105 stable

BFSP1 0.259183565 3.325651747 1.769083776 0.084765799 0.476182552 -5.46116216 stable

CMAHP 0.005146328 2.53571041 1.768816999 0.084810983 0.476182552 -5.461606028 stable

MAGOH /// MAGOHB 0.068900879 10.22478421 1.768753874 0.084821677 0.476182552 -5.461711049 stable

FAM66B /// FAM66C /// FAM66D /// LOC101928910 /// LOC102725108 0.05393572 4.210190407 1.768725211 0.084826533 0.476182552 -5.461758734 stable

PDE1A 0.009354117 2.47319238 1.768535214 0.08485873 0.47624963 -5.462074807 stable

RNF126P1 -0.0631195 7.317710077 -1.768283997 0.084901318 0.476374974 -5.462492679 stable

DDX11L2 -0.562433872 10.14105198 -1.767893173 0.084967607 0.476633218 -5.46314267 stable

LOC101927256 0.035589924 2.700032539 1.767439795 0.085044561 0.476951149 -5.463896545 stable

ARHGAP17 -0.108121801 10.70539508 -1.767253635 0.085076176 0.477014716 -5.464206044 stable

ADAM22 0.006154948 2.911447208 1.767109799 0.08510061 0.477037998 -5.464445158 stable

CEP192 0.114065668 8.330938811 1.766686295 0.085172586 0.477327707 -5.465149098 stable

HOXA7 -0.009936864 3.017361252 -1.765990421 0.085290963 0.477837963 -5.466305455 stable

FOXA2 0.003150495 2.333520871 1.765784816 0.085325966 0.477837963 -5.466647043 stable

HOXB7 0.098368625 2.87904482 1.765776536 0.085327376 0.477837963 -5.466660798 stable

NT5DC3 -0.11333724 2.556033391 -1.765673449 0.08534493 0.477837963 -5.466832051 stable

SLC25A44 0.136060669 8.388913058 1.765554376 0.085365211 0.477837963 -5.46702985 stable

DHX40 -0.065961896 10.16064219 -1.765156645 0.085432982 0.478064633 -5.467690461 stable

WDPCP -0.153356971 5.847137813 -1.765045983 0.085451846 0.478064633 -5.467874241 stable

TRPS1 0.190683147 8.380404238 1.764880551 0.085480053 0.478064633 -5.468148964 stable

ZFP82 0.249330223 7.052336716 1.764839783 0.085487005 0.478064633 -5.468216661 stable

SLCO1B3 0.013576709 3.104833676 1.764107985 0.085611883 0.478395652 -5.469431623 stable

B3GALT2 0.047209483 3.200408478 1.763948518 0.085639116 0.478395652 -5.469696321 stable

HHAT -0.217754751 4.612261016 -1.763860022 0.085654232 0.478395652 -5.469843206 stable

ITGB2 0.140316962 14.55927903 1.763837024 0.085658161 0.478395652 -5.469881377 stable

KCNN2 0.008500494 2.68468034 1.763664138 0.085687699 0.478395652 -5.47016831 stable

MIEF1 0.082747195 9.323498339 1.763643183 0.085691279 0.478395652 -5.470203087 stable

LYVE1 0.058315126 2.810558109 1.763279333 0.085753474 0.478395652 -5.470806871 stable

SORCS2 0.003796766 2.326616697 1.763188087 0.085769077 0.478395652 -5.470958271 stable

ACE2 0.013372987 3.263238799 1.763168742 0.085772386 0.478395652 -5.470990369 stable

TMEM44 0.050349444 3.311479303 1.763165313 0.085772972 0.478395652 -5.470996058 stable

DPP10-AS3 0.003176527 2.194530098 1.763119408 0.085780823 0.478395652 -5.471072222 stable

LOC101930075 /// NPIPA1 /// NPIPA2 /// NPIPA3 /// NPIPA5 /// NPIPA7 /// NPIPA8 /// PKD1P1 -0.157551672 12.40814074 -1.762917976 0.08581528 0.478395652 -5.471406414 stable

PIH1D2 0.013893011 3.156273762 1.762788001 0.08583752 0.478395652 -5.471622036 stable

LOC643201 0.022742497 3.107933962 1.762678875 0.085856197 0.478395652 -5.47180306 stable

DPEP2 -0.151995512 10.94944127 -1.762570153 0.085874807 0.478395652 -5.471983404 stable

EXT2 0.18095373 8.465734333 1.762569708 0.085874883 0.478395652 -5.471984143 stable

WBSCR22 -0.07814114 10.31509527 -1.762469952 0.085891962 0.478395652 -5.472149606 stable

NUPL1 0.085600484 7.911947115 1.762251705 0.085929337 0.478463219 -5.47251158 stable

LOC101926987 -0.087127018 2.565768209 -1.762142548 0.085948035 0.478463219 -5.472692609 stable

LINC01159 -0.07636001 3.014969916 -1.761951403 0.085980786 0.478463219 -5.473009585 stable

TMEM65 0.106211419 3.964922106 1.761924124 0.085985461 0.478463219 -5.47305482 stable

ACMSD 0.036500954 3.087174503 1.761721842 0.086020133 0.478511419 -5.473390228 stable

CCNJL 0.180709165 6.323678851 1.761636229 0.086034811 0.478511419 -5.473532175 stable

CBLC 0.003460865 2.318924295 1.761281124 0.086095715 0.478625556 -5.474120879 stable

ID2B 0.014706577 3.093817496 1.761213251 0.08610736 0.478625556 -5.47423339 stable

ENTHD2 -0.135159875 8.523668857 -1.761160687 0.086116379 0.478625556 -5.474320521 stable

ZBED8 0.029559742 2.976157823 1.760875968 0.086165247 0.478672136 -5.474792437 stable

MORF4L2 0.071549735 12.12231668 1.760874718 0.086165462 0.478672136 -5.47479451 stable

TOP1 -0.105854598 8.608391318 -1.760543387 0.086222359 0.478757545 -5.475343602 stable

LOC389641 -0.171433599 4.595331232 -1.760484977 0.086232393 0.478757545 -5.475440392 stable

HMGCR 0.159076263 9.4907798 1.760168074 0.086286848 0.478757545 -5.475965477 stable

PBX4 -0.184627198 7.467532301 -1.759972533 0.086320463 0.478757545 -5.476289433 stable

LOC283737 0.0051739 2.522380854 1.759936532 0.086326653 0.478757545 -5.476349073 stable

GOT1 0.142634229 8.27092733 1.759857254 0.086340285 0.478757545 -5.476480405 stable

RNF214 -0.059310821 7.432637045 -1.759834956 0.08634412 0.478757545 -5.476517342 stable

IGHA1 /// IGHA2 /// IGHD /// IGHG1 /// IGHG3 /// IGHM /// IGHV4-31 -0.348471939 9.639292307 -1.759779368 0.08635368 0.478757545 -5.476609424 stable

UBE2J1 -0.162525411 10.40360502 -1.759719215 0.086364026 0.478757545 -5.476709065 stable

KLF11 -0.247673531 7.542524601 -1.759279889 0.086439622 0.479063702 -5.477436706 stable

ABCC8 -0.022009448 3.143550212 -1.759160738 0.086460134 0.479064503 -5.477634023 stable

BCCIP -0.109340478 8.096155748 -1.758862806 0.086511442 0.479138 -5.478127362 stable

CEACAM5 0.048936952 2.657877726 1.758656083 0.086547057 0.479138 -5.478469627 stable

LOC286370 0.01139473 2.923346496 1.758646809 0.086548655 0.479138 -5.478484981 stable

DBNL /// MIR6837 -0.111145538 11.27464709 -1.758351657 0.086599527 0.479138 -5.478973595 stable

PEBP4 -0.068012056 2.730751392 -1.758327874 0.086603628 0.479138 -5.479012964 stable

POFUT1 0.072012859 3.296319377 1.758094636 0.086643848 0.479138 -5.479399026 stable

LOC101930405 -0.238630988 5.396309778 -1.757957964 0.086667423 0.479138 -5.479625231 stable

RP11-355B11.2 -0.198870494 6.080765955 -1.75792168 0.086673683 0.479138 -5.47968528 stable

PRKCQ 0.203642516 10.54589225 1.757902404 0.086677009 0.479138 -5.479717183 stable

CNTROB -0.114044514 7.543825966 -1.757901845 0.086677105 0.479138 -5.479718108 stable

C17orf75 0.151788455 5.022200057 1.757598068 0.08672953 0.479315147 -5.480220817 stable

PRO2012 0.00451751 2.50778224 1.757123888 0.086811415 0.479364998 -5.481005372 stable

TLL2 0.006752037 2.828561227 1.756986778 0.086835104 0.479364998 -5.481232194 stable

RP4-581F12.1 0.076104887 6.788388198 1.756868952 0.086855466 0.479364998 -5.481427103 stable

C20orf57 /// DUSP15 0.012350393 2.794198142 1.756848967 0.08685892 0.479364998 -5.481460161 stable

OR2B6 -0.020994413 3.027502303 -1.756740064 0.086877744 0.479364998 -5.481640297 stable

LOC100505812 0.269264454 7.539619469 1.756612827 0.086899741 0.479364998 -5.481850748 stable

ZFHX2 -0.056054005 6.263455448 -1.756588723 0.086903909 0.479364998 -5.481890615 stable

MAFG -0.124633085 8.447146099 -1.756504759 0.086918428 0.479364998 -5.482029482 stable

TMX4 0.156897602 9.752803513 1.756484264 0.086921973 0.479364998 -5.482063378 stable

KLHDC4 /// LOC100129637 0.26209512 8.312215724 1.756131763 0.086982952 0.479588847 -5.482646312 stable

SEC31B -0.242680319 8.022179896 -1.755752267 0.087048642 0.479709264 -5.483273776 stable

SOX17 0.003073497 2.539720645 1.755481679 0.087095505 0.479709264 -5.4837211 stable

SLC6A13 -0.021187318 2.65157298 -1.755393027 0.087110864 0.479709264 -5.483867642 stable

ARV1 -0.090760521 9.327498399 -1.755360304 0.087116533 0.479709264 -5.483921732 stable

LOC100506236 0.009368521 2.940943735 1.755345112 0.087119166 0.479709264 -5.483946844 stable

AX746690 /// RP4-564F22.5 0.009768262 2.333844392 1.755237574 0.0871378 0.479709264 -5.484124594 stable

LOC145474 -0.418215052 5.328777175 -1.755181278 0.087147557 0.479709264 -5.484217641 stable

SLC1A2 0.013755587 3.211074025 1.754618002 0.087245228 0.480018219 -5.4851485 stable

DTX3L 0.110545144 12.22813258 1.754526904 0.087261033 0.480018219 -5.485299021 stable

GALE -0.196684777 6.193923323 -1.75450457 0.087264908 0.480018219 -5.485335924 stable

ARIH2OS -0.245674693 4.572465761 -1.754324111 0.087296225 0.48007821 -5.485634077 stable

LOC102724873 /// ZNF75A 0.135144428 5.470337019 1.754101307 0.087334903 0.480099443 -5.486002155 stable

RP4-561L24.3 0.008197219 2.499907963 1.754066717 0.087340909 0.480099443 -5.486059295 stable

CTA-280A3__B.2 -0.016335287 3.412609988 -1.753773779 0.087391787 0.480266873 -5.486543168 stable

SLC16A8 -0.037538999 3.732930975 -1.75356345 0.087428332 0.480282212 -5.486890546 stable

E2F5 -0.283724153 8.421164811 -1.753236407 0.087485183 0.480282212 -5.487430618 stable

SH3GL3 0.003580031 2.346415345 1.753103726 0.087508256 0.480282212 -5.4876497 stable

LRRC48 0.00358201 2.222965995 1.752945527 0.087535773 0.480282212 -5.487910898 stable

TBK1 0.144788308 9.834382356 1.752787651 0.087563242 0.480282212 -5.488171544 stable

LINC00623 0.136243857 12.09149798 1.752776899 0.087565113 0.480282212 -5.488189293 stable

KIAA1211L 0.007263333 2.450360318 1.752736925 0.087572069 0.480282212 -5.488255286 stable

H1F0 -0.239536024 9.014689181 -1.752669208 0.087583854 0.480282212 -5.488367073 stable

MRPL50 0.162502063 7.862151114 1.752550064 0.087604593 0.480282212 -5.48856375 stable

C20orf194 -0.116331923 8.106957875 -1.752270377 0.087653291 0.480282212 -5.489025397 stable

PACS2 -0.075887153 5.033533379 -1.752262928 0.087654589 0.480282212 -5.489037691 stable

FNBP1 0.096903519 12.69522807 1.75220042 0.087665476 0.480282212 -5.489140857 stable

TDRP -0.28026606 6.965612667 -1.752087769 0.087685099 0.480282212 -5.489326772 stable

MTPN -0.091850245 8.229512204 -1.751953535 0.087708487 0.480282212 -5.489548296 stable

SLC4A11 -0.007146442 2.667860193 -1.751945713 0.08770985 0.480282212 -5.489561203 stable

NOP14 -0.082248021 8.418446417 -1.751880085 0.087721287 0.480282212 -5.489669501 stable

CPSF4 -0.064727488 9.287468959 -1.751719271 0.087749316 0.480323868 -5.48993486 stable

COMMD5 /// LOC101928879 /// ZNF250 0.192234024 9.300543395 1.751160933 0.08784669 0.480744999 -5.490856009 stable

ATP5E -0.060868736 14.07043977 -1.750782299 0.087912776 0.480994744 -5.491480539 stable

RPS29 0.028242616 15.68521088 1.750537258 0.087955567 0.481092823 -5.491884655 stable

GCSAML -0.440528075 5.066635822 -1.75044384 0.087971885 0.481092823 -5.492038706 stable

NCAPD3 0.073467245 8.517251265 1.750328339 0.087992064 0.481092823 -5.492229162 stable

LOC284898 -0.080156788 2.660322401 -1.749913484 0.088064574 0.481377373 -5.492913153 stable

LSM14A -0.097651002 12.28186769 -1.749567121 0.088125151 0.481596577 -5.493484112 stable

GSG2 -0.016592293 3.147507747 -1.749370463 0.088159561 0.481672712 -5.493808249 stable

LOC102724030 0.003248817 2.485049471 1.748855917 0.088249646 0.482031738 -5.494656189 stable

TRIM52 -0.108366304 8.520389212 -1.748761071 0.08826626 0.482031738 -5.494812466 stable

CEBPA-AS1 -0.128284623 7.56170955 -1.748519245 0.088308631 0.482151187 -5.495210888 stable

ADAMTS19 0.007644744 2.527458012 1.748366104 0.088335473 0.482185809 -5.495463172 stable

HIST3H3 -0.028027539 2.473084693 -1.748105817 0.088381109 0.482244354 -5.495891926 stable

MRPL39 0.121674097 9.260566012 1.748071069 0.088387203 0.482244354 -5.49594916 stable

RHO -0.031983402 2.925773254 -1.747661583 0.088459044 0.482389212 -5.496623557 stable

MFSD9 0.141610158 5.927181553 1.747599395 0.088469958 0.482389212 -5.496725965 stable

C12orf4 -0.119063511 8.253626957 -1.747532168 0.088481759 0.482389212 -5.496836668 stable

CFL2 0.197599404 7.135741604 1.747406226 0.088503868 0.482389212 -5.497044046 stable

AK094644 0.013703496 3.221073762 1.747017655 0.088572114 0.482389212 -5.497683793 stable

HOTAIRM1 0.308142578 5.583252056 1.747001425 0.088574965 0.482389212 -5.497710513 stable

COASY -0.102385366 9.219923522 -1.746951801 0.088583684 0.482389212 -5.497792205 stable

FRMPD3 0.151427537 4.738205968 1.746806105 0.088609287 0.482389212 -5.498032042 stable

FAM160B1 0.006600276 2.902011239 1.746685689 0.088630452 0.482389212 -5.498230251 stable

SYNC 0.271843908 5.101205119 1.74654547 0.088655103 0.482389212 -5.498461041 stable

GTF2H1 0.139121987 9.00090548 1.746441713 0.088673348 0.482389212 -5.498631808 stable

LINC00623 /// LINC00869 /// LINC01138 /// LOC103091866 0.171366383 11.55158083 1.746319974 0.088694759 0.482389212 -5.49883216 stable

LRRC20 0.091402724 4.460783641 1.746287514 0.088700468 0.482389212 -5.498885577 stable

ATP1B3 0.124411989 11.73704453 1.746285189 0.088700878 0.482389212 -5.498889405 stable

LOC100507472 /// PCSK6 -0.49007612 8.29810973 -1.74565414 0.088811938 0.48277541 -5.49992774 stable

LOC101559451 0.009554816 3.098679993 1.745580857 0.088824843 0.48277541 -5.500048301 stable

DNAJC5B -0.026918153 2.246030517 -1.745514624 0.088836508 0.48277541 -5.500157258 stable

TMED2 0.070208412 12.5965102 1.745415362 0.088853992 0.48277541 -5.500320546 stable

USP30-AS1 0.172522415 6.410010416 1.745247513 0.088883564 0.482824551 -5.500596641 stable

CPEB2 0.111110166 9.017394793 1.745028281 0.088922201 0.482922901 -5.500957221 stable

AC008753.4 -0.067313499 4.485305143 -1.744910832 0.088942905 0.48292384 -5.501150378 stable

MBD1 0.081614236 9.340441364 1.744364676 0.089039238 0.483335316 -5.502048447 stable

ATL2 0.221026667 6.084972547 1.743785063 0.089141568 0.48367624 -5.503001267 stable

WDYHV1 0.157072003 7.724414473 1.743534183 0.089185891 0.48367624 -5.503413603 stable

LOC101929662 0.005982314 2.830849954 1.743505481 0.089190963 0.48367624 -5.503460772 stable

SSPO -0.064355407 7.236939921 -1.743448393 0.089201052 0.48367624 -5.503554592 stable

LOC101928371 0.009121946 2.633399415 1.743418845 0.089206274 0.48367624 -5.503603149 stable

B4GALT5 0.269894559 10.13464612 1.743206065 0.089243887 0.48367624 -5.503952807 stable

TMBIM6 0.06670645 13.50805132 1.743194184 0.089245988 0.48367624 -5.503972329 stable

SH3RF3 -0.17663771 6.601494724 -1.742911193 0.089296034 0.483741177 -5.504437303 stable

LOC728485 -0.065371369 5.949625724 -1.742893848 0.089299102 0.483741177 -5.5044658 stable

NADK2 0.089199305 8.366308301 1.742345023 0.089396231 0.484155823 -5.505367365 stable

MAP7D3 -0.064680771 5.772844693 -1.742040431 0.089450174 0.484317597 -5.50586762 stable

KIAA1841 0.178274284 4.945544092 1.741943857 0.089467283 0.484317597 -5.506026216 stable

C16orf58 -0.12315336 9.190704128 -1.741703798 0.089509823 0.48443639 -5.506420412 stable

STRN4 -0.154978609 9.438174509 -1.741328121 0.08957643 0.484543829 -5.507037211 stable

DLEU1 -0.127104016 8.177715458 -1.741288986 0.089583371 0.484543829 -5.507101457 stable

EIF1AD 0.164794662 6.288281218 1.741243292 0.089591476 0.484543829 -5.50717647 stable

FOXO3 /// FOXO3B -0.092784519 10.55911597 -1.740997736 0.089635041 0.484571855 -5.507579554 stable

LONP1 -0.063944727 8.79379164 -1.740981844 0.089637862 0.484571855 -5.50760564 stable

CDKN1B 0.200335362 12.45435251 1.740655541 0.089695781 0.484773547 -5.508141191 stable

ZMPSTE24 0.116667491 10.47621283 1.740510938 0.089721459 0.484800928 -5.508378497 stable

CDKN2D -0.160074018 10.26100572 -1.740367187 0.089746991 0.484827512 -5.508614387 stable

HTT 0.110467341 8.850456822 1.739209711 0.089952798 0.485827732 -5.51051316 stable

KLHL5 -0.154145283 10.42190716 -1.739004302 0.089989363 0.485913636 -5.510850009 stable

ZNF90 0.113428747 4.367911245 1.738664956 0.090049796 0.486128357 -5.511406426 stable

GAFA3 0.011740048 2.960279212 1.73816884 0.090138209 0.486493992 -5.512219729 stable

FAM174A 0.131710496 8.655513565 1.737922105 0.090182207 0.486567862 -5.512624137 stable

LOC101060264 0.007919518 2.451257448 1.737744191 0.090213944 0.486567862 -5.512915714 stable

SDR39U1 -0.108355416 10.02371228 -1.737744126 0.090213955 0.486567862 -5.512915822 stable

HIST1H3B -0.146442425 3.124110906 -1.737463763 0.090263986 0.486686988 -5.513375248 stable

TMEM263 0.170449528 10.21654692 1.737276145 0.09029748 0.486686988 -5.513682658 stable

EXOSC1 0.121522361 4.047728756 1.737272575 0.090298117 0.486686988 -5.513688508 stable

THAP10 0.002848318 2.341582663 1.736315727 0.090469096 0.487496816 -5.515255856 stable

LRP10 0.059256522 12.27031398 1.735782032 0.090564579 0.487833228 -5.516129745 stable

AC002059.10 -0.030708837 3.020644824 -1.735531331 0.090609461 0.487833228 -5.516540172 stable

ATP13A5 0.004035352 2.053876763 1.735463199 0.090621662 0.487833228 -5.516651703 stable

SRGAP2 -0.362116998 7.514585254 -1.735449137 0.09062418 0.487833228 -5.516674721 stable

COL11A2 0.055969667 2.640747027 1.735387446 0.090635228 0.487833228 -5.516775705 stable

CDX2 -0.032220872 2.101002073 -1.735085532 0.090689316 0.488012676 -5.517269871 stable

ANAPC16 -0.07540672 12.56187464 -1.734830296 0.090735063 0.488147166 -5.517687577 stable

RP3-329A5.8 -0.053897429 6.106538362 -1.734680445 0.09076193 0.488180049 -5.517932793 stable

LINC00514 0.008652805 3.099580075 1.734323765 0.090825907 0.488202429 -5.518516387 stable

LRRC56 -0.130765271 5.150459997 -1.734299321 0.090830292 0.488202429 -5.518556378 stable

TTYH1 0.003228907 2.384033918 1.734149048 0.090857259 0.488202429 -5.518802219 stable

ENTPD7 0.171675027 3.974852542 1.734139481 0.090858976 0.488202429 -5.51881787 stable

MECP2 0.119840547 9.118632707 1.734007611 0.090882646 0.488202429 -5.519033588 stable

AK055981 0.1546939 6.459609349 1.733963153 0.090890627 0.488202429 -5.51910631 stable

BTN2A1 0.086170376 10.27651394 1.733720489 0.0909342 0.488248736 -5.519503225 stable

GNAO1 -0.017373066 2.070733855 -1.733565759 0.090961993 0.488248736 -5.519756285 stable

CMTM7 -0.211178992 9.47456186 -1.733274541 0.091014322 0.488248736 -5.520232518 stable

FABP5 -0.191982907 9.385616981 -1.733168716 0.091033343 0.488248736 -5.520405558 stable

SNRNP35 0.09607115 8.702269384 1.73309424 0.091046732 0.488248736 -5.520527332 stable

VILL -0.095148991 8.373839155 -1.733053643 0.091054031 0.488248736 -5.52059371 stable

ZCCHC7 -0.152923388 8.772108867 -1.733022141 0.091059695 0.488248736 -5.520645215 stable

RNF139 -0.093851998 11.36957635 -1.732990903 0.091065312 0.488248736 -5.520696288 stable

ATG4C 0.195036486 8.27560354 1.732844173 0.091091699 0.488278912 -5.520936178 stable

MAMDC2 0.005007305 2.660037545 1.732647239 0.091127126 0.488357512 -5.52125812 stable

GABARAPL1 0.160499152 8.588546448 1.732253232 0.091198037 0.488613601 -5.521902137 stable

SFTA2 -0.010883434 2.870034824 -1.732114015 0.091223104 0.488613601 -5.522129661 stable

LOC388692 0.271195675 9.04554247 1.731944239 0.091253681 0.488613601 -5.522407109 stable

HIST1H2AB -0.037234345 2.470957617 -1.731920229 0.091258006 0.488613601 -5.522446344 stable

DEGS2 0.083221594 3.183126702 1.731444955 0.091343652 0.488909136 -5.5232229 stable

KRCC1 0.089227881 11.36028419 1.731308273 0.091368295 0.488909136 -5.523446192 stable

INPP5E -0.113618989 9.081374112 -1.731267978 0.091375561 0.488909136 -5.523512017 stable

CCBL2 -0.087474454 10.57510176 -1.731119949 0.091402258 0.488940755 -5.523753826 stable

GNB5 -0.074659327 2.458961646 -1.730959882 0.091431133 0.48898401 -5.524015279 stable

BC012193 -0.069871933 3.731172886 -1.730771341 0.091465154 0.489011186 -5.524323214 stable

SERPINC1 0.004374236 2.676573943 1.730701302 0.091477795 0.489011186 -5.524437597 stable

C9orf85 0.082293799 5.860587703 1.73034667 0.091541823 0.489180285 -5.525016703 stable

FTH1P5 -0.051401702 14.51848449 -1.730288066 0.091552408 0.489180285 -5.525112392 stable

CD2 0.177388486 12.72929781 1.730180595 0.091571821 0.489180285 -5.525287866 stable

PTPRN -0.069736308 3.669705452 -1.729947194 0.091613993 0.489294443 -5.525668918 stable

RSL1D1 -0.134699586 11.85970842 -1.729509293 0.091693159 0.489606081 -5.526383719 stable

SLC44A3 0.005588799 2.405649193 1.729279677 0.091734692 0.489686348 -5.526758467 stable

MORC3 0.097003484 10.52701475 1.729023904 0.091780976 0.489686348 -5.527175856 stable

LINC01097 0.011973542 2.68795718 1.728827054 0.091816611 0.489686348 -5.527497054 stable

HORMAD2 0.005806831 2.699837781 1.728805129 0.091820581 0.489686348 -5.527532827 stable

SLC23A3 0.021042683 3.059882341 1.728709664 0.091837867 0.489686348 -5.527688583 stable

ATP1A1 0.067964834 11.27137025 1.72860095 0.091857555 0.489686348 -5.527865946 stable

PIGU 0.111795623 8.033042981 1.728598418 0.091858014 0.489686348 -5.527870076 stable

ZFYVE1 0.089150335 8.946468471 1.72847458 0.091880446 0.489686348 -5.528072103 stable

ZCWPW1 -0.081963031 5.426193299 -1.728391139 0.091895563 0.489686348 -5.52820822 stable

COPG2IT1 -0.065507867 3.0576445 -1.727852528 0.091993194 0.490095563 -5.529086715 stable

ADH4 0.096240967 6.283600034 1.727488962 0.092059144 0.490335852 -5.529679573 stable

LOC101929857 /// LOC101929864 /// SLX1A /// SLX1A-SULT1A3 /// SLX1B /// SULT1A3 /// SULT1A4 0.180418879 5.833751855 1.727310514 0.092091528 0.490397292 -5.529970524 stable

CDIPT 0.05275192 11.36694089 1.726462188 0.092245612 0.491037117 -5.531353331 stable

INTS6-AS1 -0.199136912 6.336020498 -1.72631502 0.092272364 0.491037117 -5.531593161 stable

CFLAR /// LOC102724614 0.120189516 9.046486706 1.726304315 0.09227431 0.491037117 -5.531610605 stable

PRSS22 -0.059195158 5.487006435 -1.725381006 0.092442301 0.491740947 -5.533114859 stable

ERCC6 /// ERCC6-PGBD3 /// PGBD3 0.204846586 4.95182811 1.725347595 0.092448385 0.491740947 -5.53316928 stable

EML3 -0.060165956 10.15833399 -1.725139384 0.092486305 0.491750169 -5.533508396 stable

H2AFY2 -0.105629461 6.885094969 -1.724928294 0.092524763 0.491750169 -5.533852166 stable

ENDOD1 -0.312629993 8.167169211 -1.724888022 0.092532101 0.491750169 -5.533917745 stable

ANKRD16 -0.125144866 7.551095714 -1.724807447 0.092546786 0.491750169 -5.534048954 stable

LOC286190 0.009910105 3.14310592 1.724758573 0.092555694 0.491750169 -5.534128538 stable

HMGB4 0.021471521 2.785553643 1.724649587 0.09257556 0.491750169 -5.534305996 stable

PDGFB 0.182722004 4.496075448 1.723956543 0.092701975 0.492214451 -5.535434241 stable

LOC101929528 -0.014857778 2.993153277 -1.723940967 0.092704818 0.492214451 -5.535459594 stable

UCN2 -0.026746538 3.748064315 -1.723452271 0.092794047 0.492465927 -5.536254929 stable

IL1RAP 0.099770624 7.494551187 1.723452224 0.092794056 0.492465927 -5.536255005 stable

WDR83 0.096890067 7.05674928 1.723328072 0.092816736 0.492475173 -5.536457027 stable

C6orf58 -0.006842219 2.389612912 -1.722977265 0.092880846 0.492659295 -5.5370278 stable

MRPL3 -0.06379509 11.27505071 -1.722908986 0.092893328 0.492659295 -5.537138879 stable

NIFK-AS1 0.056119602 2.841586171 1.722296387 0.093005382 0.493068069 -5.53813532 stable

KIAA0232 0.089568285 10.50197434 1.72225842 0.09301233 0.493068069 -5.538197068 stable

AP1B1 -0.105172792 10.07383978 -1.722127485 0.093036296 0.493083985 -5.538409999 stable

DZANK1 0.013401442 3.071554475 1.721891815 0.093079446 0.493129543 -5.538793223 stable

MRPS9 0.102961496 9.17085049 1.721782434 0.093099479 0.493129543 -5.538971072 stable

PYY2 -0.14548051 3.855035725 -1.721737065 0.093107789 0.493129543 -5.539044837 stable

GNAL 0.30750865 3.201059766 1.721113216 0.093222121 0.493623933 -5.540058984 stable

IQSEC3 0.003064293 2.141247486 1.720922409 0.093257113 0.493698078 -5.540369101 stable

IGKC /// IGKV2-28 /// IGKV2-28 /// IGKV2D-28 /// IGKV2D-28 -0.471962234 9.460399351 -1.720504343 0.093333821 0.493826804 -5.541048481 stable

FCGR2B -0.400112065 9.28509614 -1.720501717 0.093334303 0.493826804 -5.541052748 stable

CENPQ 0.147160938 5.296143721 1.720446632 0.093344414 0.493826804 -5.541142253 stable

LOC101929243 -0.169570555 7.85925488 -1.720249188 0.093380664 0.493907487 -5.541463051 stable

GAS2L3 -0.042453571 2.901256919 -1.719561887 0.093506939 0.49446419 -5.542579503 stable

ZCRB1 -0.081429258 10.14604741 -1.719341104 0.093547533 0.49456766 -5.542938061 stable

SPATA7 0.155506518 4.001404216 1.719026647 0.093605375 0.494740032 -5.543448681 stable

ZFAND6 0.062047648 11.385286 1.718855071 0.093636948 0.494740032 -5.543727254 stable

VEPH1 -0.249305857 5.579883715 -1.718632268 0.093677961 0.494740032 -5.544088967 stable

SKAP1 0.20265929 10.34437757 1.718488855 0.093704368 0.494740032 -5.54432177 stable

HYI -0.11347605 5.84313883 -1.718485371 0.093705009 0.494740032 -5.544327425 stable
[truncated: 1,722,377 more chars]
